# Telescoped Flow Synthesis of Azacyclic Scaffolds Exploiting the Chromoselective Photolysis of Vinyl Azides and Azirines

**DOI:** 10.1002/chem.202401491

**Published:** 2024-06-21

**Authors:** Ruairi Crawford, Marcus Baumann

**Affiliations:** ^1^ University College Dublin School of Chemistry Science Centre South D04 N2E5 Dublin Ireland

## Abstract

An efficient chromoselective photochemical process is presented for the synthesis of 2*H*‐azirines and 1,3‐diazabicylo[3.1.0]hex‐3‐enes from readily available vinyl azides. The method exploits continuous flow photochemistry to enable the safe consumption of the hazardous azide group and provides uniform irradiation using high‐power LEDs at 365–450 nm. Additionally, a scaled telescoped process has been developed providing access to drug‐like 1,6‐dihydropyrimidines and pyrimidines *via* integrated ring‐expansion and oxidation reactions. Given the prevalence of various azacyclic targets in pharmaceutical, agrochemical and materials applications it is anticipated that this methodology will enable further exploitations of these important scaffolds.

## Graphical abstract

“This study reports an efficient and readily scalable photochemical route into azirines and related photo‐dimers that can be accessed from simple vinyl azides in a chromoselective manner. The value of these entities is demonstrated through flow‐based conversions to highly substituted dihydropyrimidines and pyrimidines that are privileged scaffolds in medicinal and materials chemistry.”

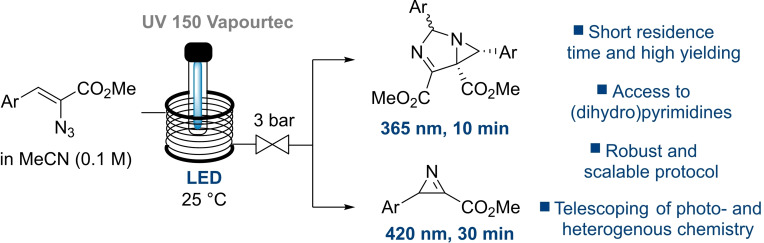

## Introduction

Azides are highly versatile handles frequently used as precursors for a variety of nitrogen‐based heterocycles, however, multiple hazards are associated with these entities such as their short life‐time, toxicity and explosive nature.^[1]^ In recent years flow reactor technology is widely being appreciated in both academia and the fine chemical industry to enable the safe use of hazardous reagents.^[2]^ Reactor miniaturisation and spatiotemporal processing thereby enable more precise control over reaction conditions, and a high surface area to volume ratio facilitates the safe use of light‐ and heat‐sensitive reactive intermediates.[^3^, ^4^, ^5^] Moreover, a paradigm shift can be seen whereby modern chemical reactions are triggered by more sustainable approaches.^[6]^ Photons being tuneable and traceless reagent equivalents are ideal species in this context. Thus, photochemical reactions in flow mode have numerous benefits over traditional batch set‐ups due to short pathlengths, uniform irradiation and unrestricted scalability.[^7^, ^8^]

The last decade witnessed a number of attractive examples for the synthesis of 2*H*‐azirines from vinyl azides.[^3^, ^9^, ^10^, ^11^, ^12^] 2*H*‐Azirines are valuable synthetic building blocks which are amongst the smallest nitrogen containing heterocycles. In addition to acting as a nucleophile on nitrogen and being an electrophile on the imine type carbon, azirines can react as dienophiles and dipolarophiles after ring opening to an azomethine ylide, thus displaying very diverse reactivity.[^13^, ^14^] Relevant studies underpinning this intriguing reactivity include works from Kirschning and co‐workers who in 2013 demonstrated the safe consumption of vinyl azides both thermally and photochemically for the synthesis of 2*H*‐azirines, with applications in trapping of the azomethine ylide intermediate.^[9]^ In 2021, the Luisi group developed a continuous flow approach for the generation of 2*H*‐azirines *via* thermolysis of vinyl azides followed by quenching with alkyl lithium nucleophiles.^[11]^

**Scheme 1:**
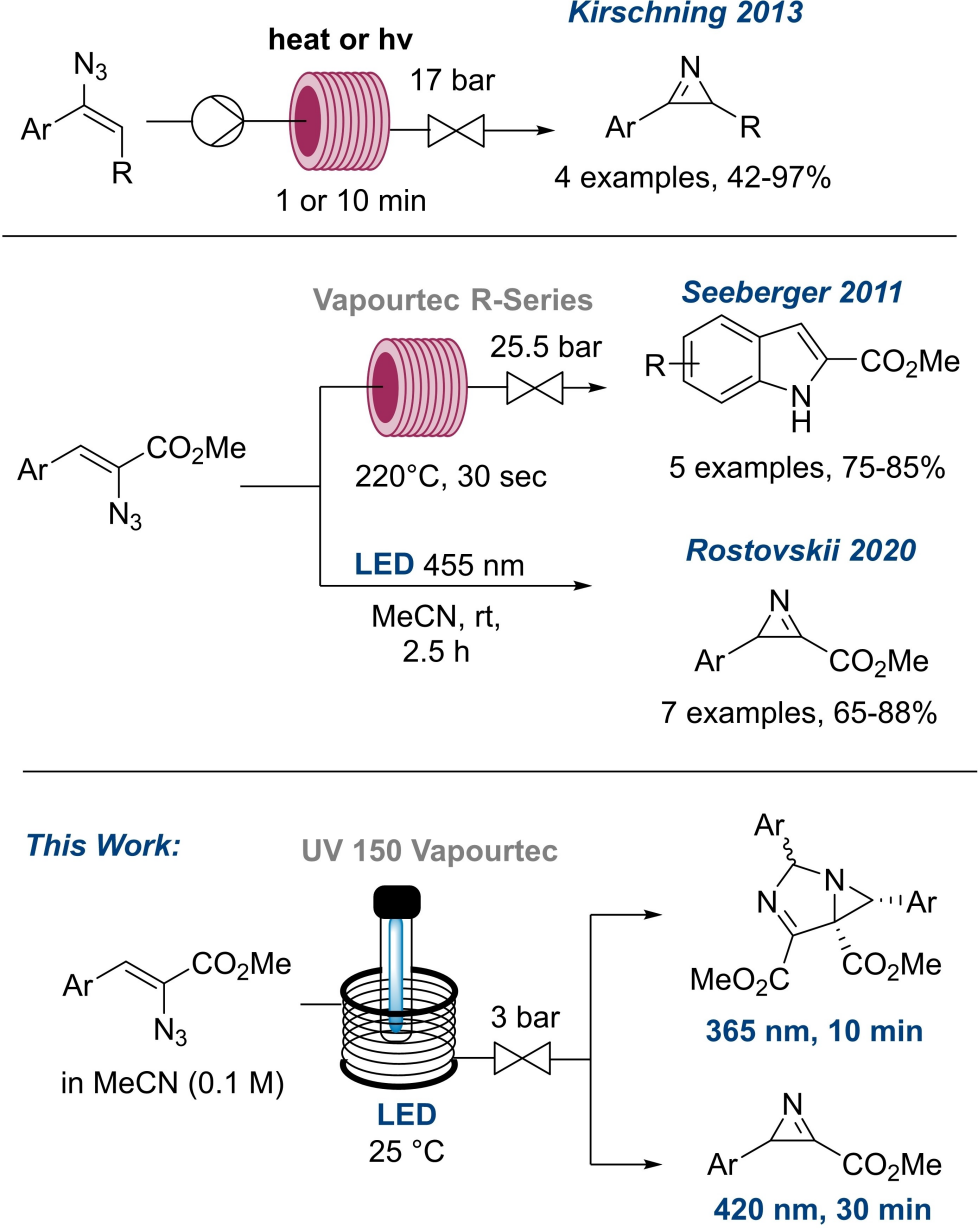
Examples of reported applications of vinyl azides.[^9^, ^10^, ^18^]

To date, there are several publications which have explored a specific type of vinyl azides of interest.[^15^, ^16^, ^17^] Moreover, in 2011 Seeberger and co‐workers developed a continuous flow platform for the generation of indoles from vinyl azides achieving yields between 75–85 % in 30 seconds *via* a superheating approach (Scheme 1).^[18]^ The Rostovskii group synthesised 2*H*‐azirines under batch photochemical conditions on small scale (2.5 hours, 455 nm, Scheme 1).^[10]^ However, under batch conditions handling the azide presents some risks due to the possibility of pressure spikes due to sudden nitrogen gas release. Consequently, the scalability of batch photochemical routes remains challenging.

Cognisant of the significant synthetic utility of 2*H*‐azirines we wished to develop a continuous flow platform for the scalable and safe consumption of different vinyl azides under photochemical conditions giving rise to 2*H*‐azirines and related 1,3‐diazabicylo[3.1.0]hex‐3‐enes. Ultimately, in this study we also report a telescoped flow approach merging the initial photochemical reactions with heterogenous downstream transformations for the synthesis of drug‐like 1,6‐dihydropyrimidines and pyrimidines.

## Results and Discussion

We initiated our study by preparing a library of vinyl azides from commercially available methyl bromoacetate and benzaldehydes (see ESI for details).[^15^, ^16^] Using vinyl azide **1a** as the model substrate the formation of 2*H*‐azirines was studied under photochemical conditions. This concerned the effect of different light sources and wavelengths, as well as variations of both residence time and concentration on the yield of product **2a**. Suitable conditions were identified when using LEDs at 420 nm (24 W input power), 30 min residence time and a substrate concentration of 0.1 M (in MeCN).

Firstly, irradiating **1a** with a UV−A LED at 365 nm results in the exclusive formation of diastereomeric dimers (**3a**, **3a’**, Table 1, entry 1). This can be explained by the concomitant ring opening of the 2*H*‐azirine followed by 1,3‐dipolar cycloaddition with **2a**. A switch to 450 nm light prevented this process in favour of selectively producing compound **2a**. Initially, this gave moderate conversions between 9–56 % possibly indicating a weak absorbance of **1a** in the wavelength region (entries 2–4). Next, it was decided to change the light source and irradiate **1a** with light of 420 nm for 20 min (entry 5) resulting in a high yield of 85 %. Increasing the residence time to 30 min improved the yield further (91 %) while minimising dimer formation (<2 %, entry 6). Increasing or reducing the concentration of the substrate had no beneficial effect on the outcome (entries 7 and 8) while increasing the scale to 200 mg showed that the excellent yield can be retained for this flow process (entry 9). Crucially, in flow the reaction time has been reduced significantly to 30 minutes compared to a previous batch method (150 minutes).^[10]^

**Table 1:** Optimisation table for the synthesis of azirine product **2a**.

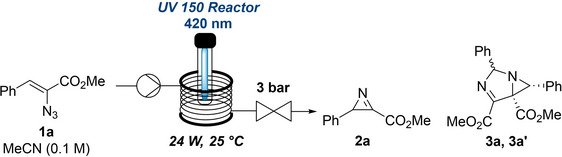				
Entry	t_Res_ (min)	Yield of **2a**	Conversion of **1a** to **2a**	Yield of **3a**/**3a’**
1^a^	10	0	0	99
2^b^	10	7	9	0
3^b^	30	39	40	0
4^b^	50	48	56	0
5	20	85	86	0
6	30	91	95	<2
7^c^	30	81	95	<2
8^d^	30	82	92	<2
9^e^	30	92	94	<2

Yields are based on ^1^H‐NMR using 1,3,5‐trimethoxybenzene as internal standard. Reactions were performed on 25 mg scale in MeCN (0.1 M). ^a^ Reaction was performed at 365 nm (70 W). ^b^ Reaction was performed at 450 nm (24 W). ^c^ At 200 mM concentration. ^d^ At 50 mM concentration. ^e^ Reaction was performed on 200 mg scale.

**Scheme 2:**
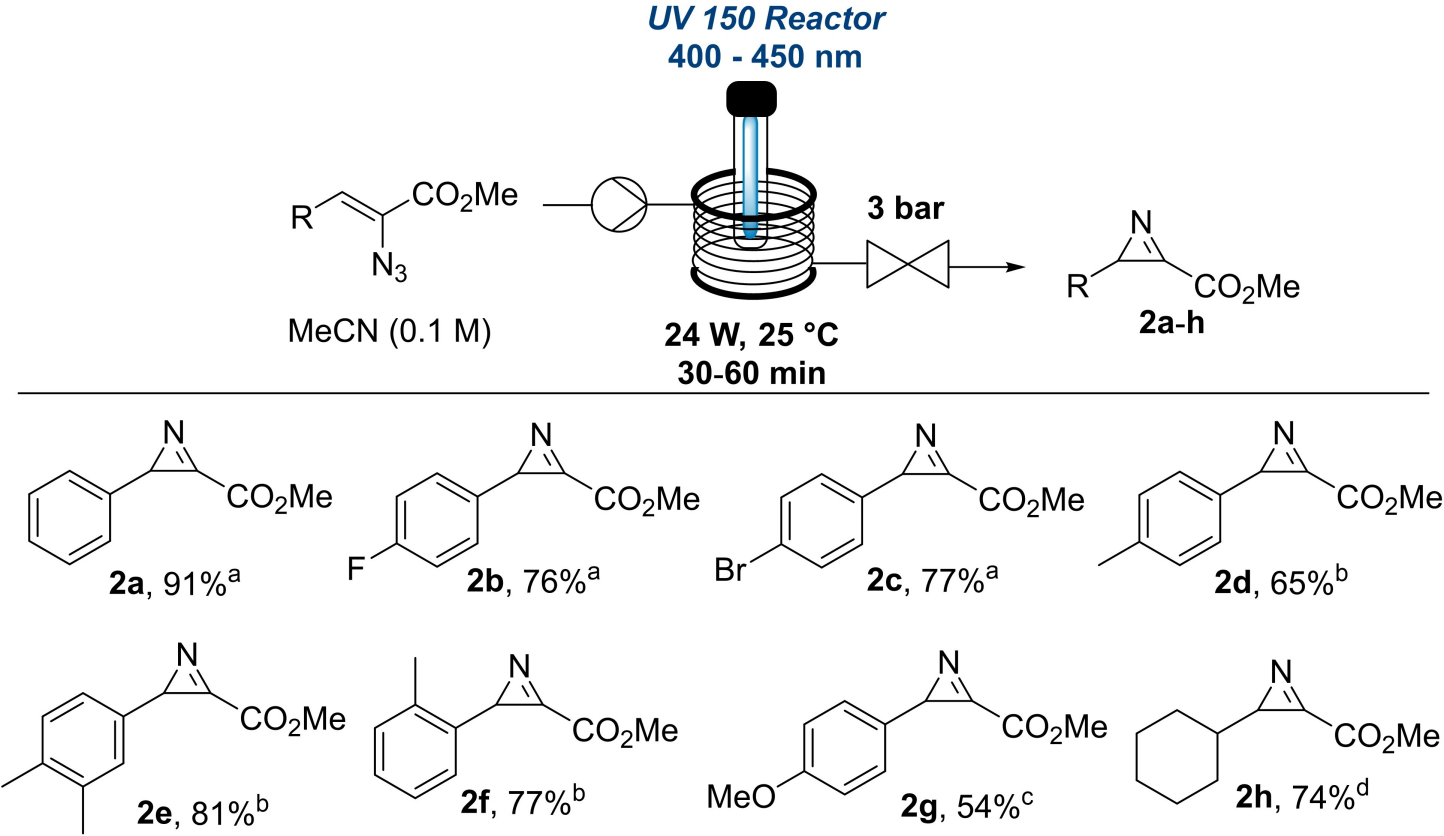
Synthesis of 2*H*‐azirines (**2a‐2h**) from vinyl azides under flow conditions. Yields reported are ^1^H‐NMR yields (internal standard: 1,3,5‐trimethoxybenzene). ^a^ 420 nm LED, 24 W, 30 min. ^b^ 450 nm LED, 24 W, 60 min. ^c^ 450 nm LED, 24 W, 20 min. ^d^ 400 nm LED, 40 W and 45 min.

Using the highest yielding conditions different vinyl azides were evaluated next. This showed that electron withdrawing groups on the aryl portion provided the product in high yields of ca. 76 % (**2b** and **2c**, Scheme 2). Conversely, using an LED emitting at 450 nm and adjusting the residence time to 60 mins was necessary for more electron‐rich systems to selectively generate the desired azirine products **2d**–**g** in good yields of 65–81 %. To minimise competing side‐reactions further adjustment was needed for the most electron‐rich system giving product **2g** in a yield of 54 % along with 27 % remaining starting material when reducing the residence time to 20 minutes. Product **2h** bearing a cyclohexyl ring necessitated a lower wavelength in combination with a longer residence time (400 nm, 45 min) to give a comparable yield.

### Synthesis of 1,3‐Diazabicylo[3.1.0]hex‐3‐enes

As shown above, variation of the wavelength used to convert the vinyl azide substrates allowed for tuning the yields and selectivities towards the azirine targets. Amongst the side products observed in this process the photodimerisation towards 1,3‐diazabicylo[3.1.0]hex‐3‐enes stood out. As photoflow chemistry has been exploited in the past by us and others to generate more complex scaffolds^[19]^ on demand, we decided to investigate whether these underexplored 1,3‐diazobicylco[3.1.0]hex‐3‐enes can be accessed selectively. This was an appealing target as only two prior studies on their synthesis and functionalisation are reported which suffer from limited scalability due to using batch operations.[^10^, ^20^]

**Scheme 3:**
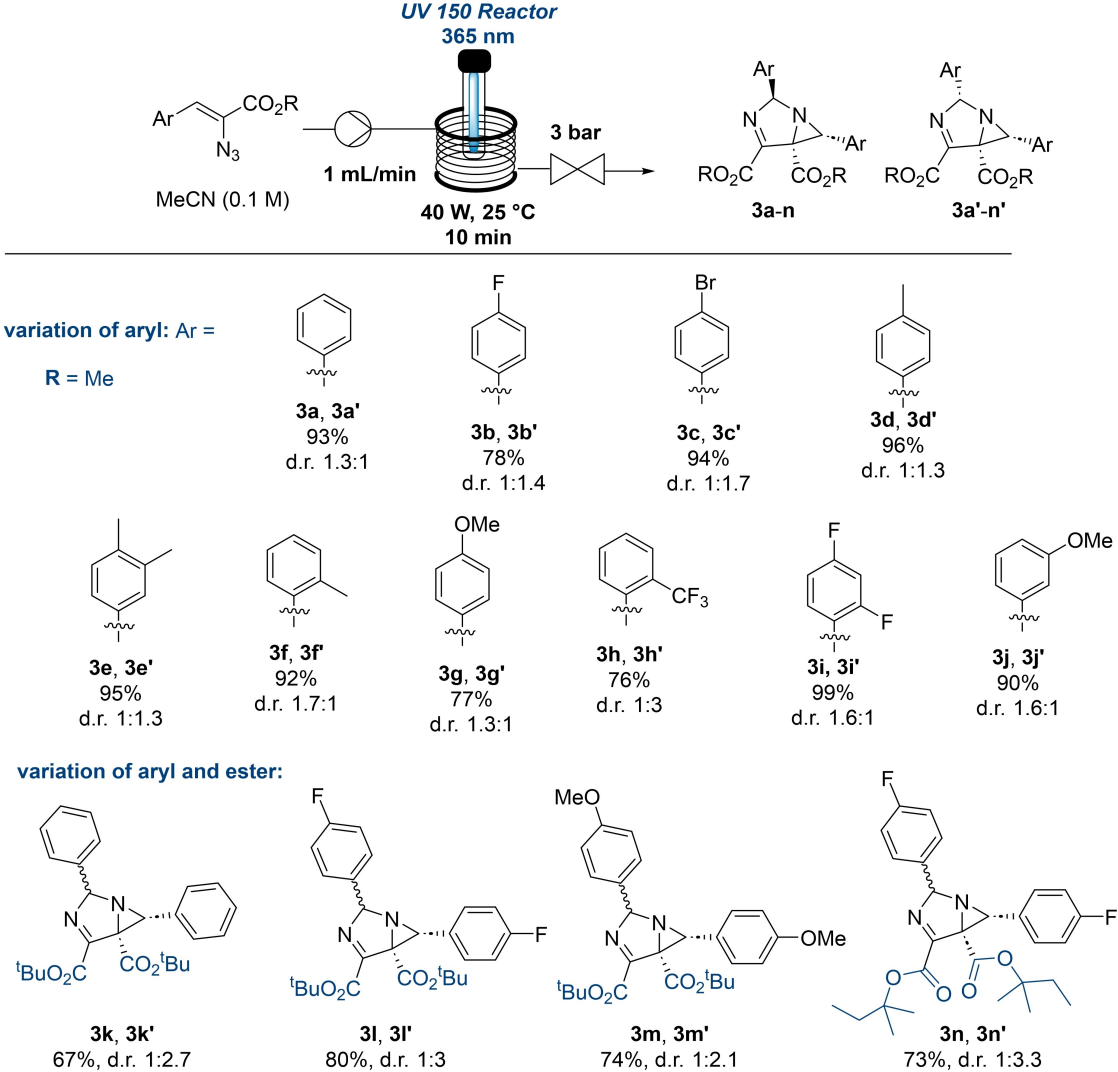
Synthesis of 1,3‐diazobicylco[3.1.0]hex‐3‐ene library (**3a**‐**n**). Reported yields after column chromatography.

As shown in Scheme 3 this objective was quickly met with success when utilising a high‐power LED emitting at 365 nm in combination with a shorter residence time of 10 minutes. The desired products (**3a**–**n**) were obtained as mixtures of diastereoisomers which could be separated *via* column chromatography, except for compounds possessing ortho‐substituted aryl rings. This approach tolerated different substitution patterns on the aryl ring for electron‐donation and electron‐withdrawing groups in either the ortho, meta or para positions. In addition, variations on the ester moiety were well tolerated giving the desired products in high yields when using tert. butyl and tert. pentyl esters which also showed a slight change in diastereoselectivity compared to the smaller methyl ester exploited previously. A marked improvement in productivity was observed for this flow method due to its short residence time (10 min) allowing to access larger quantities of these unusual scaffolds compared to the initial batch report.^[10]^

**Scheme 4:**
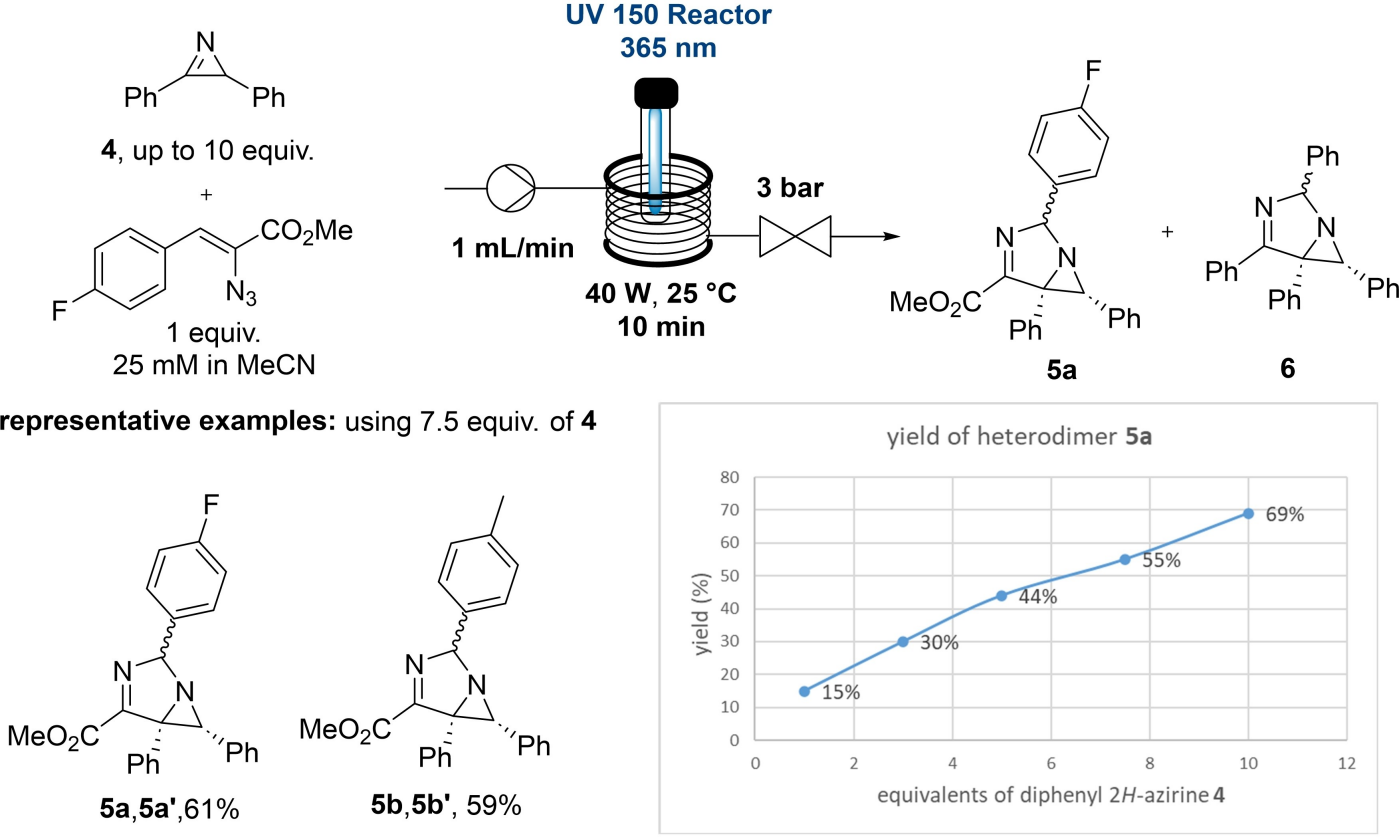
Synthesis of heterodimeric 1,3‐diazabicylco[3.1.0]hex‐3‐enes **5a**,**5a’** and **5b**,**5b’**.

The possibility of forming heterodimeric 1,3‐diazabicylco[3.1.0]hex‐3‐enes *via* 1,3‐dipolar cycloaddition between two different 2*H*‐azirines is an attractive prospect in view of using these compounds as building blocks towards other heterocyclic structures. Reviewing the literature identified 2,3‐diphenyl 2*H*‐azirine (**4**) as a potentially suitable dipolarophile.[^21^, ^22^] Test reactions indicated that a reduction of the input power from 70 to 40 W was possible. Using these conditions (10 min, 365 nm, 40 W) mixtures of 2,3‐diphenyl 2*H*‐azirine and vinyl azide **1b** were irradiated in the flow set‐up showing quickly that 2,3‐diphenyl 2*H*‐azirine needs to be used in excess to control the formation of the desired heterodimer **5** and recover unreacted 2,3‐diphenyl 2*H*‐azirine afterwards. Using this approach the desired heterodimer could be obtained as the major product when using at least 7.5 equivalents of the dipolarophile. On preparatory scale a set of heterodimeric products bearing either a fluoride or methyl group in the para position gave the desired products **5a**–**b** in good yields, whereas a para‐methoxy group gave various unknown side products in this case (details not shown). Despite these limitations, this method shows for the first time that heterodimeric 1,3‐diazabicylco[3.1.0]hex‐3‐enes can be accessed in short residence times *via* the direct photolysis of vinyl azides with an excess of 2,3‐diphenyl 2*H*‐azirine (Scheme 4).

### Synthesis of 1,6‐Dihydropyridimines and Related Pyrimidines:

Base‐mediated ring expansion of 1,3‐diazobicyclo[3.1.0]hex‐3‐enes is known to generate 1,6‐dihydropyridimines in yields of ranging from 42–97 % when using DBU in MeCN.^[10]^ Such aryl dihydropyrimidines are valuable entities that have been studied for the treatment of chronic infections with hepatitis B virus and can be seen within a drug candidate synthesised by Roche.[^23^, ^24^] We therefore wished to investigate whether flow approaches could further streamline the access to these heterocycles starting from vinyl azides. Specifically, as prior batch reports use solution phase bases (e. g., DBU) in small scale reactions a potential for straightforward reaction scale‐up in flow was recognised especially when using heterogeneous bases in a packed‐bed reactor.

**Scheme 5:**
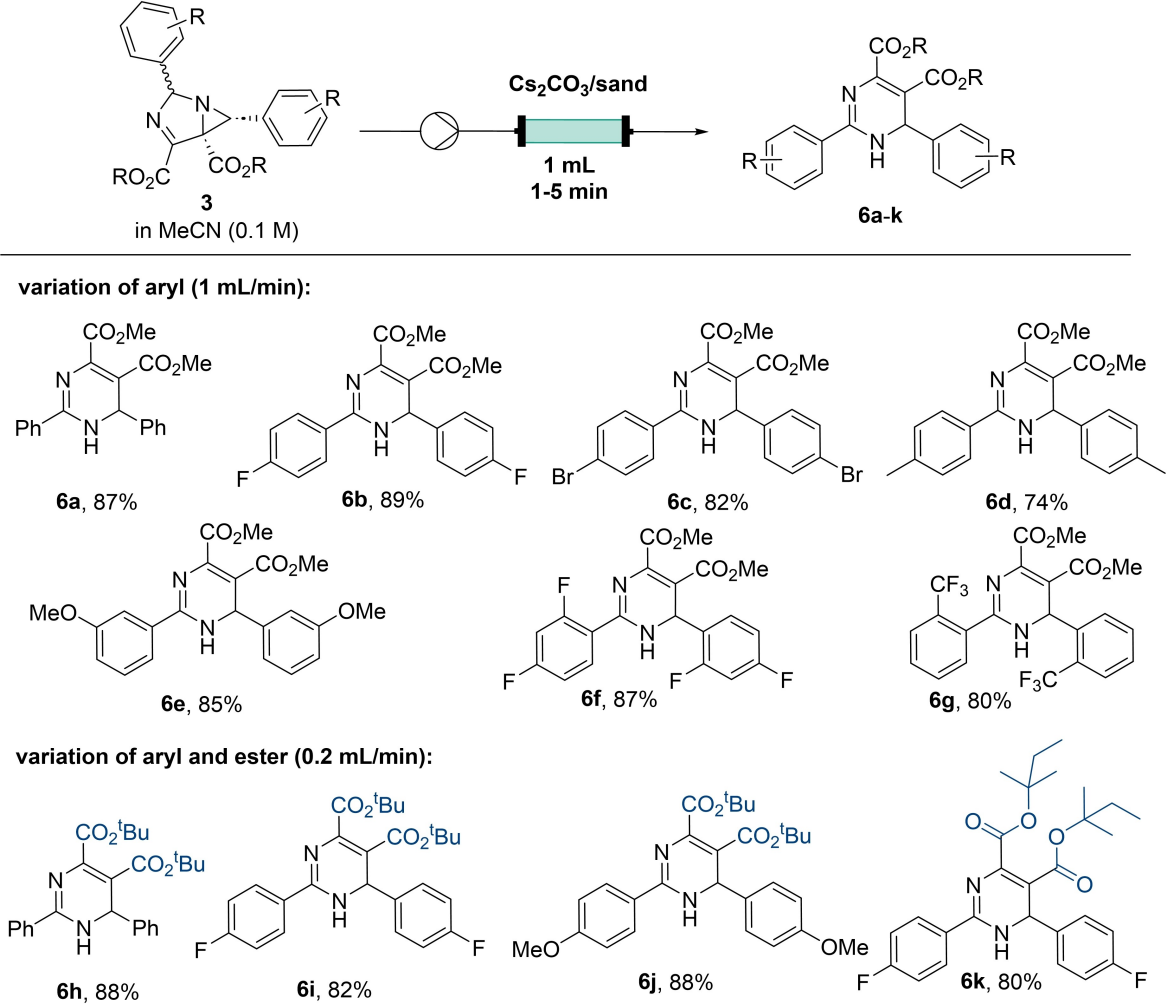
Synthesis of 1,6‐dihydropyrimidines (**6a**‐**k**) *via* flow isomerisation approach. Packed bed reactor was filled with 1 g of Cs_2_CO_3._

Cs_2_CO_3_ was quickly identified as a suitable insoluble base that could be filled into Omnifit glass columns to affect the facile ring expansion of 1,3‐diazabicylco[3.1.0]hex‐3‐enes in flow mode (Scheme 5). This approach would not only eliminate the need for separating the spent base during purification, but moreover it would also benefit from better mass transfer thus accelerating the rate of this reaction.^[25]^

When directing a solution of 1,3‐diazabicylco[3.1.0]hex‐3‐enes in MeCN through a packed column filled with a mixture Cs_2_CO_3_ and sand (used as an inert additive) high yields of the desired 1,6‐dihydropyrimidines were obtained within a short residence time of ca. 1 minute. Overall, this transformation is characterised by a very good scope allowing the use of electron‐donating substituents (**6d**–**e**) as well as electron‐withdrawing groups (**6b**–**c**, **6f**–**g**). Replacing the methyl ester for bulkier esters was tolerated when the residence time was increased to 5 min (**6h**–**k**).

**Scheme 6:**
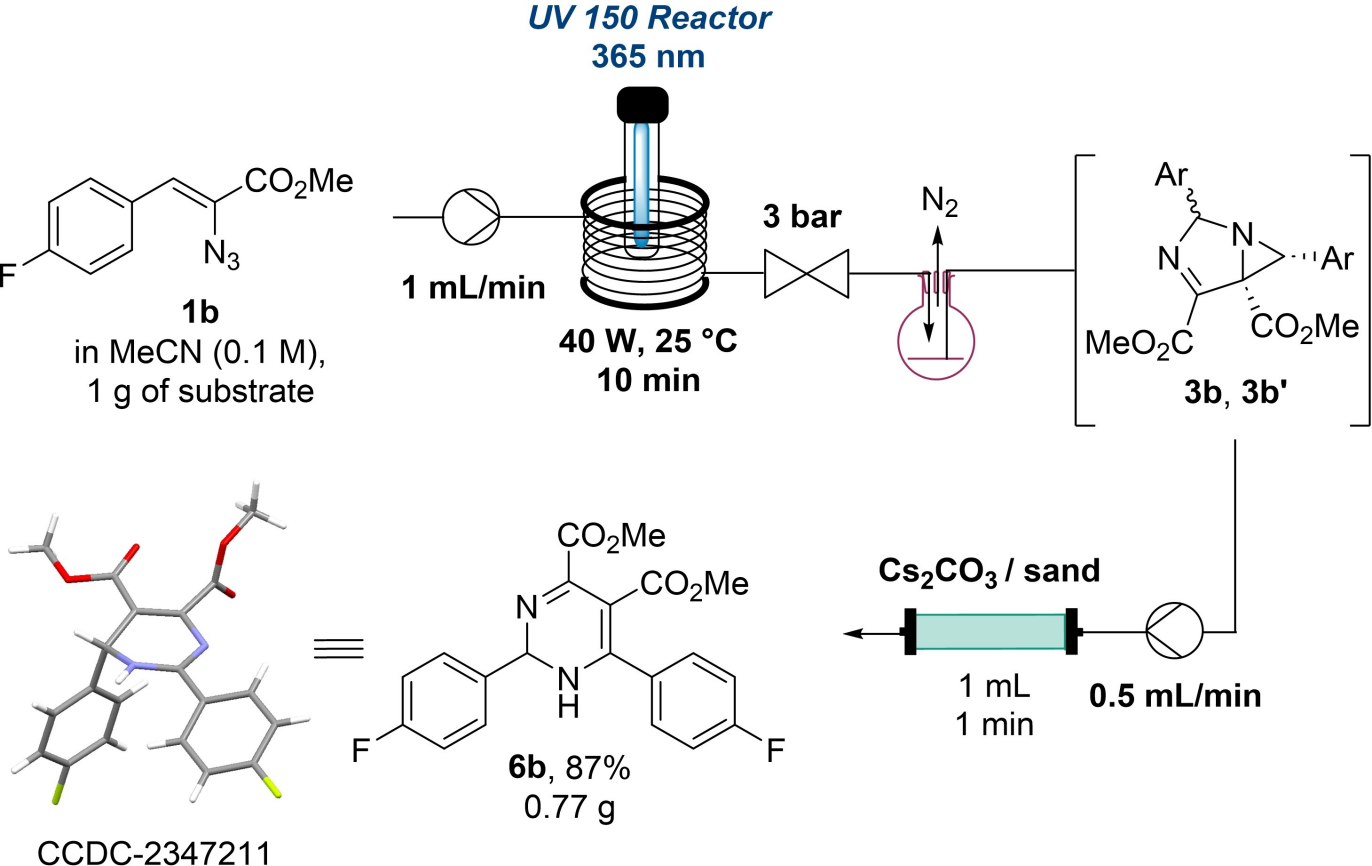
Continuous flow synthesis of dihydropyrimidine **6b** by telescoping photochemical process and heterogeneous transformation. Packed bed reactor was filled with 1.8 g of Cs_2_CO_3._

To further evaluate the robustness of this ring expansion approach, it was merged with the initial photochemical stage *via* reaction telescoping^[27]^ for the synthesis of **6b** in a gram‐scale reaction (Scheme 6). The diastereomeric mixture of 1,3‐diazabicylco[3.1.0]hex‐3‐enes (**3b**/**3b’**) was thereby collected in a reservoir from where it was directly pumped into the pack‐bed column to facilitate removal of N_2_ gas. To our delight, this approach worked very well and increased the product yield over the two steps (i. e., 87 % compared to 69 % for the non‐telescoped version). In addition, single crystals of product **6b** could be obtained allowing confirmation of the anticipated connectivity of the 1,6‐dihydropyrimidine product.

**Scheme 7:**
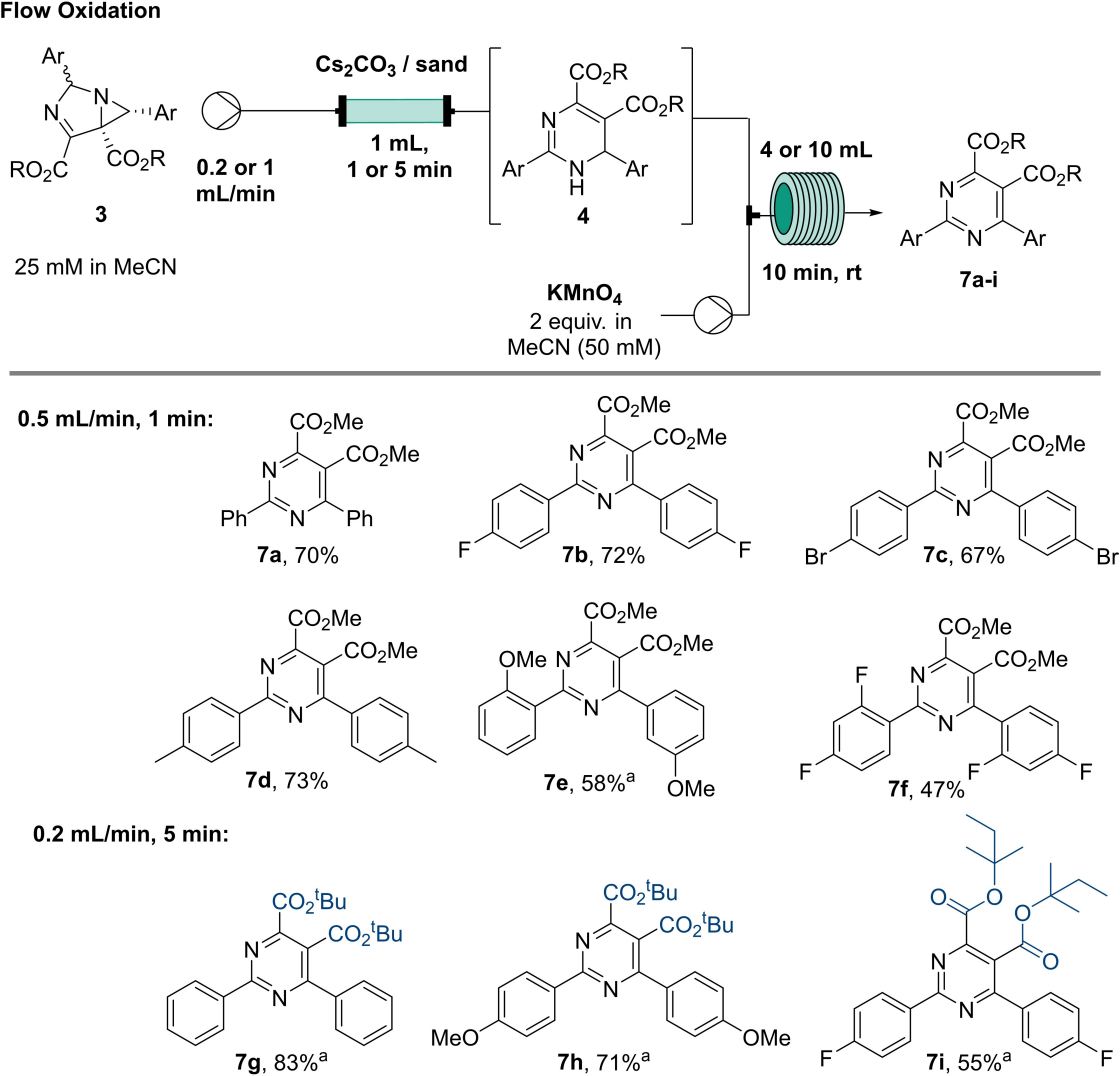
Synthesis of pyrimidine library. ^a^ Reactions were performed at a higher concentration of 1,3‐diazabicylo[3.1.0]hex‐3‐ene (0.1 M, MeCN) and KMnO_4_ stream (0.2 M, MeCN/H_2_O (1 : 1)). Packed bed reactor was filled with 1 g of Cs_2_CO_3._ Reported yields are isolated yields.

Being able to effectively generate 1,6‐dihydropyrimidines from 1,3‐diazabicylco[3.1.0]hex‐3‐enes we wished to couple their oxidation to the corresponding pyrimidine products in a telescoped manner as such densely functionalised scaffolds are gaining momentum in the pharmaceutical and materials fields.[^28^, ^29^, ^30^, ^31^, ^32^] Moreover, we targeted the replacement of previously used oxidants such as DDQ,^[10]^ a hazardous and non‐environmentally friendly agent. Initial efforts considered the use of MnO_2_ as a solid oxidant that could be packed into flow cartridges, however, it was found that a stronger oxidant was needed to obtain the desired pyrimidines in a fast and clean process. Therefore, a switch to KMnO_4_ (50 mM, MeCN) was favoured which showed promise as full conversion of dihydropyrimidines was observed within 10 minutes enabling reaction telescoping as a feasible process (Scheme 7). The resulting flow sequence used tubular reactor coils (at r.t., 10 mL) and syringe pumps in combination with a slight excess of the oxidant rendering the pyrimidine products in high yields.

Overall, telescoping these two steps was successful giving products **7a**–**i** in good yields between 47–83 % in an overall residence time of 11 minutes. For pyrimidines **7e** and **7f** a slight drop in yield was noticed as some of the 1,6‐dihydropyrimidine intermediate remained in the column (indicated by distinct yellow colour). Importantly, the throughput of the flow process can be increased fourfold by increasing the concentrations of the respective substrate and oxidant streams without compromising the yields (see **7g**–**i**). Moreover, this flow approach was successfully applied to accommodate different ester groups (**7g**–**i**) delivering the corresponding products in good to excellent yields. Though the analogous batch mode oxidation is a viable alternative giving good yields of the desired products (e. g., **7j** and **7k**), this approach requires the removal of the base and thus leads to longer process times.

**Scheme 8:**
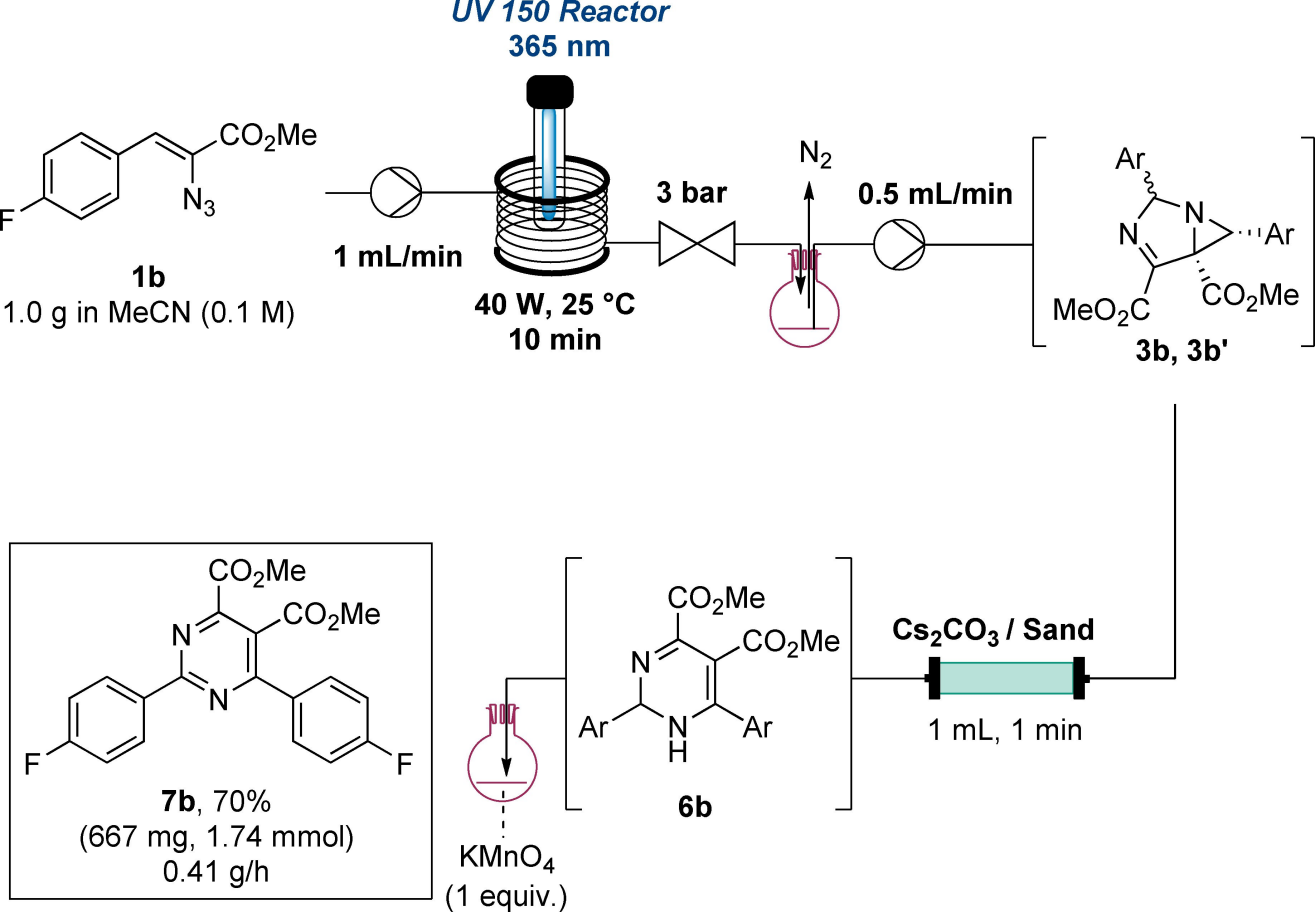
Telescoped 3‐step synthesis of pyrimidine **7b**. Packed bed reactor was filled with 1.8 g of Cs_2_CO_3._

The final objective of this study concerned the integration of all synthetic steps to achieve the synthesis of a pyrimidine product (e. g., **7b**, Scheme 8) directly from the vinyl azide substrate without isolation of any intermediate. As shown in Scheme 8 this was achieved by telescoping the photochemical formation of the 1,3‐diazabicylo[3.1.0]hex‐3‐ene followed by its ring expansion using a packed‐bed reactor as discussed above. The outflow from the packed‐bed reactor was subsequently collected in a flask containing KMnO_4_ (1 equiv.) where upon stirring at ambient temperature the desired pyrimidine was generated. This approach exploited continuous flow processing for the key transformations of the vinyl azide and the resulting 1,3‐diazabicylo[3.1.0]hex‐3‐ene and was found to be the most straightforward and robust means to generate the pyrimidine target with an overall yield of 70 % on gram‐scale from **1b**.

## Conclusions

In conclusion, robust flow protocols have been developed for the safe and effective conversion of vinyl azides under photochemical conditions to give various nitrogen containing heterocycles including 2*H*‐azirines and 1,3‐diazabicylco[3.1.0]hex‐3‐enes. The use of different high‐power LEDs emitting between 365 and 450 nm enabled the selective transformation of the vinyl azide substrates to give either target in short residence times and with good productivities. In addition, this flow approach was amenable to generate heterodimeric 1,3‐diazabicylco[3.1.0]hex‐3‐enes when using 2,3‐diphenyl 2*H*‐azirine as a premade dipolarophile. The flow‐based conversion of 1,3‐diazabicylco[3.1.0]hex‐3‐enes into 1,6‐dihydropyrimdines and pyrimidines as potential drug precursors was achieved exploiting Cs_2_CO_3_ as a heterogeneous reagent placed in a packed‐bed reactor, and KMnO_4_ as a solution‐phase oxidant. Several telescoped approaches to these azacyclic targets were developed showcasing how this methodology can be employed for generating both small libraries of drug‐like compounds as well as demonstrating the accessibility of selected targets on gram scale.

## Conflict of interests

The authors declare no conflict of interest.

1

## Supporting information

As a service to our authors and readers, this journal provides supporting information supplied by the authors. Such materials are peer reviewed and may be re‐organized for online delivery, but are not copy‐edited or typeset. Technical support issues arising from supporting information (other than missing files) should be addressed to the authors.

Supporting Information

## Data Availability

The data that support the findings of this study are available in the supplementary material of this article.
