# Supplementary material for: Telescoped Flow Synthesis of Azacyclic Scaffolds Exploiting the Chromoselective Photolysis of Vinyl Azides and Azirines
Source: Chemistry. 2024 Jun 21;30(38):e202401491. doi: 10.1002/chem.202401491 (PMC11497334; doi:10.1002/chem.202401491)

Chemistry–A European Journal

Supporting Information

Telescoped Flow Synthesis of Azacyclic Scaffolds Exploiting the Chromoselective Photolysis of Vinyl Azides and Azirines

Ruairi Crawford and Marcus Baumann*

Telescoped Flow Synthesis of Azacyclic Scaffolds Exploiting the Chromoselective Photolysis of Vinyl Azides and Azirines

Ruairi Crawford and Marcus Baumann*

Email: marcus.baumann@ucd.ie

University College Dublin, School of Chemistry, Science Centre South, D04N2E5, Dublin, Ireland

Contents

General materials and methods 3

Experimental procedures 4

Characterisation data of starting materials 8

Characterisation data of vinyl azides 8

Characterisation of 2*H*-azirines 13

Characterisation of 1,3-diazabicyclo[3.1.0]hex-3-ene-4,5-dicarboxylates 15

Characterisation of 1,6-dihydropyrimidine-4,5-dicarboxylate 29

Characterisation of pyrimidine-4,5-dicarboxylates 34

X-Ray data 39

Pictures of the flow equipment 40

References 41

Copies of NMR Data 42

General materials and methods

Substrates, reagents, and solvents were used as purchased without further purification. ^{1}H-NMR spectra were recorded at 25 ˚C using a Varian VNMRS 400, 500 MHz spectrometers. Deuterated solvents acquired from Sigma-Aldrich were used as supplied. Spectra recorded in ppm using the chosen solvent peak as a reference ($CHCl_3$ δ_H = 7.27 ppm) or DMSO-d_6 (δ_H = 2.50 ppm). Data for ^{1}H-NMR are reported as follows: chemical shift (δ/ppm) (multiplicity, coupling constant (Hz), integration).^{13}C[^{1}H] NMR spectra were recorded on the same instruments (100 and 125 MHz) and are reported relative to $CHCl_3$ (δ 77.0 ppm) or DMSO-d_6 (δ 39.52 ppm). ^{19}F NMR were recorded at 376 MHz. Multiplicities are reported as follows: s = singlet, d = doublet, t = triplet, q = quartet, m = multiplicity (C, CH, CH_2, CH_3). Experiments were used in the structural assignment.

IR spectra were obtained by use of a Platinum spectrometer (near, ATR sampling, Bruker, Billercia, MA, USA) with intensities of the characteristic signals as reported as weak (w, <20% of tallest signal), medium (m, 21-70% of tallest signal) or strong (s, >71% of tallest signal).

High-resolution mass spectrometry was performed using the indicated techniques on a micromass LCT orthogonal time of flight mass spectrometer and quadropole time-of-flight mass spectrometer with leucine-enkephalin (Tyr-Gly-Phe-Leu) as internal lock mass.

Melting points were recorded on Stuart SMP10 melting point apparatus.

Flow reactions were performed using a Vapourtec E-Series UV-150 photoflow reactor, containing a high-power LED (365 nm, 400 nm, 420 and 450 nm) and a reactor coil of 10 mL volume (FEP tubing). Reactions were also performed using The Easy Scholar Flow reactor equipped with Omnifit® column.

TLC was performed on Merck pre-coated Silica gel 60 F254 aluminium plates with realisation by UV irradiation at 254nm, $KMnO_4$. Flash chromatography was performed using Macherey-Nagel silica gel 60 M, with a particle range of 0.04 - 0.063 mm.

Experimental procedures

Note: Several azirines and azabicyclo[3.1.0]hex-2-ene-2-carboxylates reported in this study were found to be prone to degradation when evaporation of residual solvents takes places in rotavap water baths (40 ^{o}C) for prolonged periods of time. Evaporation time therefore were reduced and NMR spectra of some of these compounds consequently show small amounts of residual solvents such as EtOAc.

General procedure A for the synthesis of Methyl 2-azidoacetate

Following a procedure previously published.[1] Methyl 2-bromoacetate (1 equiv.) was dissolved in MeOH (13.6 M) and a slurry of sodium azide (1.2 equiv.) in deionised H_2O (22.3 M) was added at room temperature. The flask was submerged into a water bath and stirred for 20 minutes at room temperature. The flask was heated to 80 °C for 2 hours. The reaction mixture was poured onto deionised water (50 mL) and extracted with Et_2O (3 x 80 mL). The combined organic phases were dried with Na_2SO_4 and the solvent was evaporated in *vacuo* to afford the product.

General procedure B for the synthesis of substituted 2-azido-3-acrylates (1a-j)

Following a procedure previously published. [1] A suspension of sodium methoxide (1.5 equiv.) in methanol (1.76 M) was cooled to -20 °C. The appropriate aldehyde (1 equiv.) and methyl 2-azidoacetate (2.5 equiv.) mixture was added over a period of 20 minutes. The mixture was stirred for 1 hr at -20 °C. The mixture was then allowed to warm to room temperature and stirred overnight. The reaction mixture was diluted with deionised water (20 mL) and allowed to stir for a further 5 minutes. The organic layer was extracted with Et_2O (3 x 20 mL). The combined organic phases were washed with H_2O (2 x 20 mL) and brine (20 mL) and dried with Na_2SO_4. The solvent was evaporated in vacuo to afford the crude product. The crude material was purified by SiO_2 column chromatography using EtOAc/pentane (2-20%).

General procedure C for the synthesis of substituted 2-azido-3-acrylates (k-n)

Following a procedure previously published.[2] In an inert RBF dry THF (0.28 M) was added, alongside the appropriate lithium alkoxide (1.3 equiv.) added slowly at -10 °C. After 20 minutes the appropriate vinyl azide dissolved in THF (2 mL) was added slowly. The reaction mixture was allowed to warm and stir room temperature for 2 hours. The reaction mixture was quenched with H_2O and extracted with CH_2Cl_2 (3 x 30 mL) and washed with brine (20 mL) and dried with Na_2SO_4. The solvent was evaporated in vacuo to afford the crude product. The crude material was purified by SiO_2 column chromatography using EtOAc/pentane (2-20%).

General procedure D for the synthesis of 2*H*-azirines (2a-c)

A solution of vinyl azide in MeCN (100 mM) was prepared and passed through the UV-150 Vapourtec photoreactor equipped with 420 nm LED lamp (residence time 30, approx. 25 °C, 24 W and 3 bar). The reaction mixture was collected, the solvent evaporated in vacuo to afford the crude product.

General procedure E for the synthesis of 2*H*-azirines (2d-g)

A solution of vinyl azide in MeCN (100 mM) was prepared and passed through the UV-150 Vapourtec photoreactor equipped with 450 nm LED lamp (residence time 60 min, approx. 25 °C, 24 W and 3 bar). The reaction mixture was collected, the solvent evaporated in vacuo to afford the crude product.

General procedure F for the synthesis of 2*H*-azirines (2h)

A solution of vinyl azide in MeCN (100 mM) was prepared and passed through the UV-150 Vapourtec photoreactor equipped with 400 nm LED lamp (residence time 45 min, approx. 25 °C, 70 W and 3 bar). The reaction mixture was collected, the solvent evaporated in vacuo to afford the crude product.

General procedure G for the synthesis of 1,3-diazabicyclo[3.1.0]hex-3-ene-4,5-dicarboxylate (3a-n)

A solution of vinyl azide in MeCN (100 mM) was prepared and passed through the UV-150 Vapourtec photoreactor equipped with 365 nm LED lamp (10 min residence time, approx. 25 °C, 40 W input power and 3 bar). The reaction mixture was collected, the solvent evaporated in vacuo to afford the crude product. The crude material was purified by SiO_2 column chromatography using EtOAc/pentane (2-20%).

Procedure for the synthesis of 2,3-diphenyl-2H-azirine (4)

Following a procedure previously published.[3]

General procedure for the synthesis of heterodimeric azabicyclo[3.1.0]hex-2-ene-2-carboxylate (5a-5b)

A solution of the appropriate vinyl azide (1b or 1d, 1 equiv.) in MeCN in MeCN (25mM) and the desired equiv. of dipolarophile (**5**, 1-10 equiv.) in MeCN (25 mM) was prepared and pass through the UV-150 Vapourtec photoreactor equipped with 365 nm LED lamp (10 min residence time, approx. 25 °C, 40 W input power and 3 bar). The reaction mixture was collected, the solvent evaporated in vacuo to afford the crude product. The crude material was purified by SiO_2 column chromatography using EtOAc/pentane (2-20%).

General procedure H for the synthesis of 1,6-dihydropyrimidine-4,5-dicarboxylate in Flow (6a-k)

A solution of the desired 1,3-diazabicyclo[3.1.0]hex-3-ene-4,5-dicarboxylate was dissolved in MeCN (25 mM or 0.1 M). The solution is pumped through the Vapourtec Easy Scholar Flow reactor equipped with packed column of Cs_2CO_3/sand. The crude product is collected, the solvent is evaporated in *vacuo* to afford the crude product The crude material was purified by SiO_2 column chromatography using EtOAc/ pentane (20-30%).

<u>Prepping packed column and residence time of packed column calculation</u>

An Omnifit® glass column was filled with 2 cm of sand. A blend of sand (1 cm) Cs_2CO_3 (1 g) was made up and 4 cm of this was added into the column and the remaining space was filled with sand. The weight of the dry mass column was taken three 3 times to get an average. MeCN was passed through the glass column for 20 minutes and the wet mass was taken three times to get an average.

Calculations for 8 cm (height) x 1 cm (outer diameter) Omnifit® column

Dry Mass = 33.68 g, Wet Mass = 34.46 g

Difference = 0.78 g

Density = Mass/ Volume, density of MeCN = 0.786 g/cm^3

0.786 g/cm ^{3} = 0.78 g/ Volume

Volume of packed column = **1 mL**

Gram Scale Flow Synthesis of 1,6-dihydropyrimidine-4,5-dicarboxylate (6b)

Vinyl azide (**1b**) was dissolved in MeCN (100 mM) was prepared passed through the UV-150 Vapourtec photoreactor equipped with 365 nm LED lamp (10 min residence time, approx. 25 °C, 40 W input power and 3 bar). The reaction mixture was collected into a RBF to allow release of N_2. The solution of the desired 1,3-diazabicyclo[3.1.0]hex-3-ene-4,5-dicarboxylate was pumped through the Vapourtec Easy Scholar Flow Reactor at 0.5 mL/min equipped with an Omnifit packed column of Cs_2CO_3/sand. The crude product is collected, the solvent is evaporated in *vacuo* to afford the crude product The crude material was purified by SiO_2 column chromatography using EtOAc/ pentane (20-30%).

General Procedure I for the synthesis of pyrimidine-4,5-dicarboxylate (7a-d,f)

A solution of desired 1,3-diazabicyclo[3.1.0]hex-3-ene-4,5-dicarboxylate in MeCN (25 mM) was pumped through the Vapourtec Easy Scholar Flow Reactor at 0.5 mL/min equipped with an Omnifit packed column of Cs_2CO_3/sand. The crude product was met with a stream of $KMnO_4$ (2 equiv. in MeCN, 50 mM) pumping at 0.5 mL/min and passed through a PFA coil (10 mL) with a residence time of 10 minutes. The reaction mixture is collected and quenched with $NaHSO_3$ solution (1 equiv. in minimal amount of H_2O) extracted with Et_2O (3 x 20 mL) and brine (1 x 20 mL). The combined organic phases were dried with Na_2SO_4 and the solvent was evaporated in *vacuo* to afford the crude product. The crude material was purified by SiO_2 column chromatography using EtOAc/ Pentane (10-20%)

General Procedure J Flow Synthesis of pyrimidine-4,5-dicarboxylate (7e,g-i)

A solution of desired 1,3-diazabicyclo[3.1.0]hex-3-ene-4,5-dicarboxylate in MeCN (0.1 M) was pumped through the Vapourtec Easy Scholar Flow Reactor equipped at flow rate of 0.5 mL/min with a Omnifit packed column of Cs_2CO_3/sand. The crude product was met with a

stream of $KMnO_4$ (2 equiv. in MeCN:H_2O (1:1, 0.2 M) pumping at 0.5 mL/min and passed through a PFA coil (10 mL) with a residence time of 10 minutes.

The reaction mixture is collected and quenched with $NaHSO_3$ solution (1 equiv. in minimal amount of H_2O) extracted with Et_2O (3 x 20 mL) and brine (1 x 20 mL). The combined organic phases were dried with Na_2SO_4 and the solvent was evaporated in *vacuo* to afford the crude product. The crude material was purified by SiO_2 column chromatography using EtOAc/ Pentane (10-20%)

Gram Scale Flow Synthesis of pyrimidine-4,5-dicarboxylate (7b)

Vinyl azide (**1b**) was dissolved in MeCN (100 mM) was prepared passed through the UV-150 Vapourtec photoreactor equipped with 365 nm LED lamp (10 min residence time, approx. 25 °C, 40 W input power and 3 bar). The reaction mixture was collected into a RBF to allow release of N_2. The solution of the desired 1,3-diazabicyclo[3.1.0]hex-3-ene-4,5-dicarboxylate was pumped through the Vapourtec Easy Scholar Flow Reactor at 0.5 mL/min equipped with an Omnifit packed column of Cs_2CO_3/sand. The crude product is collected and solid $KMnO_4$ (1 equiv.) was added, from the last drop of the 1,6-dihydroypyrimidine 10 minutes is timed. The reaction mixture is collected and quenched with $NaHSO_3$ solution (1 equiv. in minimal amount of H_2O) extracted with Et_2O (3 x 20 mL) and brine (1 x 20 mL). The combined organic phases were dried with Na_2SO_4 and the solvent was evaporated in *vacuo* to afford the crude product. The crude material was purified by SiO_2 column chromatography using EtOAc/ Pentane (10-20%)

Batch Synthesis of pyrimidine-4,5-dicarboxylate (7j-k)

The desired 1,6-dihydropyrimidine-4,5-dicarboxylate in MeCN (25 mM) was prepared. $KMnO_4$ (1 equiv.) was added to the reaction mixture. The reaction progress was monitored by TLC. Upon completion of the reaction ~15 minutes the reaction mixture is extracted with Et_2O (3 x 20 mL) and brine (1 x 20 mL). The combined organic phases were dried with Na_2SO_4 and the solvent was evaporated in *vacuo* to afford the crude product. The crude material was purified by SiO_2 column chromatography using EtOAc/ Pentane (10-20%).

Characterisation data of starting material

Methyl 2-azidoacetate

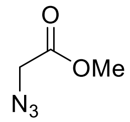

Chemical Formula: $C_3H_5N_3O_2$
Molecular Weight: 115.09

Yield: 90% (5.7 g, 48.9 mmol)

Appearance: Colourless oil

^{1}H-NMR (400 MHz, $CDCl_3$) δ/ppm 3.88 (s, 2H), 3.80 (s, 2H). **^{13}C-NMR (100 MHz, $CDCl_3$)** δ/ppm 168.9 (C), 52.7 (CH_3), 50.4 (CH_2). In agreement with data previously reported.[1]

Characterisation data of Vinyl Azides

Methyl (*Z*)-2-azido-3-phenylacrylate (1a)

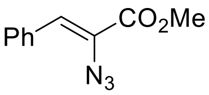

Chemical Formula: $C_{10}H_9N_3O_2$
Molecular Weight: 203.20

Yield: 85 % (1.16 g, 5.71 mmol)

Appearance: Yellow oil

^{1}H-NMR (500 MHz, $CDCl_3$) δ/ppm 7.62 (d, *J* = 7.3 Hz, 2H), 7.39-7.34 (m, 3H), 6.92 (s, 1H), 3.92 (s, 3H). **^{13}C-NMR (125 MHz, $CDCl_3$)** δ/ppm 164.4 (C), 133.5 (C), 131.0 (2CH), 129.9 (CH), 128.9 (2CH), 126.0 (CH), 125.7 (C), 55.3 (CH_3). **IR (neat)** v/cm^{-1}: 2113 (s), 1711 (s), 1615 (m), 1435 (m), 1375 (m), 1253 (s), 1210 (s), 961 (w), 812 (s), 687 (s), 657 (m), 451 (w). **HR-MS (TOF ES+):** calcd for $C_{10}H_9N_3O_2Na$ 226.0588 found 226.0587. In agreement with data previously reported.[1]

Methyl (*Z*)-2-azido-3-(4-fluorophenyl)acrylate (1b)

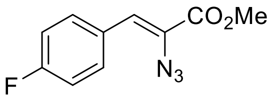

Chemical Formula: $C_{10}H_8FN_3O_2$
Molecular Weight: 221.19

Yield: 62% (920 mg, 2.81 mmol)

Appearance: Light-yellow solid

Melting point: 54-56 °C

^{1}H-NMR (400 MHz, $CDCl_3$) δ/ppm 7.62 (m, 2H), 7.07 (m, 2H), 6.88 (s, 1H), 3.91 (s, CH_3). **^{13}C-NMR (100 MHz, $CDCl_3$)** δ/ppm 164.7 (d, *J* = 250 Hz, CF), 162.4 (C), 133.1 (d, *J* = 9 Hz, 2CH), 129.9 (d, *J* = 3 Hz, C), 125.4 (d, J = 3 Hz, C), 124.8 (CH), 116.2 (d, J = 21 Hz, 2CH), 53.4 (CH_3). **^{19}F-NMR (376 MHz, $CDCl_3$) δ/ppm** -109.7 (m). **IR (neat)** v/cm^{-1}: 2120 (s), 1707 (s), 1504 (m), 1317 (w), 1225 (s), 1156 (s), 880 (w), 822 (s), 650 (m), 481 (w). **HR-MS (TOF ES+):** calcd for $C_{10}H_9FN_3O_2$ 222.0634 found 222.0634. In agreement with data previously reported. [1]

Methyl (*Z*)-2-azido-3-(4-bromophenyl)acrylate (1c)

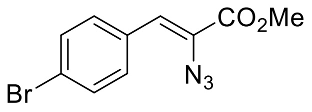

Chemical Formula: $C_{10}H_8BrN_3O_2$
Molecular Weight: 282.10

Yield: 76% (844 mg, 2.98 mmol)

Appearance: Yellow Solid

Melting point: 53-54 °C

^{1}H-NMR (400 MHz, CDCl$_3$) δ/ppm 7.68 (m, 2H), 7.51 (m, 2H), 6.81 (s, 1H), 3.91 (s, 3H), **^{13}C-NMR (100 MHz, CDCl$_3$)** δ/ppm 164.2 (C), 132.5 (C), 132.4 (2CH), 132.1 (2CH), 126.4 (C), 124.4 (CH), 124.0 (CH), 53.4 (CH_3). **IR (neat)** v/cm^{-1}: 2113 (s), 1703 (s), 1613 (m), 1582 (m), 1484 (m), 1379 (s), 1241 (s), 1207 (s), 1186 (s), 1007 (m), 702 (w)m 463 (w). **HR-MS (TOF ES+):** calcd for $C_{10}H_8BrN_3O_2Na$ 303.9692 found 303.0692. In agreement with data previously reported. [1]

Methyl (*Z*)-2-azido-3-(p-tolyl)acrylate (1d)

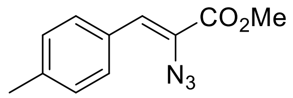

Chemical Formula: $C_{11}H_{11}N_3O_2$
Molecular Weight: 217.23

Yield: 58% (271 mg, 1.25 mmol)

Appearance: Yellow solid

Melting point: 66-68 °C

^{1}H-NMR (400 MHz, CDCl$_3$) δ/ppm 7.72 (d, *J* = 8.3 Hz, 2H), 7,21 (d, *J* = 8.3 Hz, 2H), 6.91 (s, 1H), 3.91 (s, 3H), 2.38 (s, 3H). **^{13}C-NMR (100 MHz, CDCl$_3$)** δ/ppm 164.6 (C), 140.4 (C), 131.1 (2CH), 130.9 (C), 129.7 (2CH), 126.3 (CH), 124.9 (C), 53.3 (CH_3), 22.0 (CH_3). **IR (neat)** v/cm^{-1}: 2181 (s), 1707 (s), 1616 (m), 1436 (m), 1399 (m), 1321 (m), 1247 (s), 1208 (s), 1076 (m), 816 (s), 755 (m), 631 (w),465 (s). **HR-MS (TOF ES+):** calcd for $C_{11}H_{12}N_3O_2$ 218.0885 found 218.0885. In agreement with data previously reported. [2]

Methyl (*Z*)-2-azido-3-(3,4-dimethylphenyl)acrylate (1e)

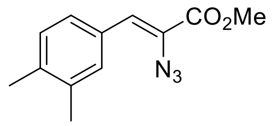

Chemical Formula: $C_{12}H_{13}N_3O_2$
Molecular Weight: 231.26

Yield: 68% (1.14 g, 2.92 mmol)

Appearance: Yellow solid

Melting point: 80 °C

^{1}H-NMR (400 MHz, CDCl$_3$) δ/ppm 7.58 (d, *J* = 6.6 Hz, 2H), 7.16 (d, *J* = 2.4 Hz, 1H), 6.89 (s, 1H), 3.93 (s, 3H), 2.29 (s, 6H). **^{13}C-NMR (100 MHz, CDCl$_3$)** δ/ppm 164.1 (C), 138.6 (C), 136.6 (C), 131.7 (CH), 130.7 (C), 129.7 (CH), 128.1 (CH), 126.0 (CH), 124.1 (C), 52.7 (CH_3), 19.7 (2CH_3). **IR (neat)** v/cm^{-1}: 2117 (s), 1699 (s), 1615 (m), 1432 (m), 1369 (m), 1253 (s), 1235 (s), 1080 (s), 925 (m), 711 (m), 647 (w), 444 (w). **HR-MS (TOF ES+):** calcd for $C_{12}H_{14}N_3O_2$ 232.1069 found 232.1081.

Methyl (*Z*)-2-azido-3-(o-tolyl)acrylate (1f)

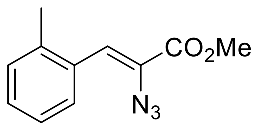

Chemical Formula: $C_{11}H_{11}N_3O_2$
Molecular Weight: 217.23

Yield: 72% (1.06 g, 4.88 mmol)

Appearance: Yellow solid

Melting point: 57-58 °C

^{1}H-NMR (400 MHz, CDCl$_3$) δ/ppm 7.98 (m, 1H), 7.24 (m, 3H), 7.14 (s, 1H), 3.93 (s, 3H), 2.37 (s, 3H).). **^{13}C-NMR (100 MHz, CDCl$_3$)** δ/ppm 164.5(C), 138.1 (C), 132.4 (C), 130.8 (CH), 130.1 (CH), 129.7 (CH), 126.6 (C), 126.3 (CH), 124.0 (CH), 53.4 (CH_3), 20.6 (CH_3). **IR (neat)** v/cm^{-1}: 2112 (s), 1709 (s)m 1435 (m), 1377 (m), 1245 (s), 1076 (m), 958 (w), 817 (m), 779 (m), 650 (w). **HR-MS (TOF ES+):** calcd for $C_{11}H_{12}N_3O_2$ 218.0885 found 218.0885. In agreement with data previously reported. [1]

Methyl (*Z*)-2-azido-3-(4-methoxyphenyl)acrylate (1g)

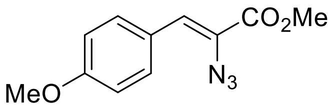

Chemical Formula: $C_{11}H_{11}N_3O_3$
Molecular Weight: 233.23

Yield: 86% (890 mg, 3.67 mmol)

Appearance: Light-yellow solid

Melting point: 80 °C

^{1}H-NMR (400 MHz, CDCl$_3$) δ/ppm 7.80 (m, 2H), 6.92 (m, 2H), 6.89 (s, 1H), 3.89 (s, 3H), 3.84 (s, 3H). **^{13}C-NMR (100 MHz, CDCl$_3$)** δ/ppm 164.4 (C), 160.6 (C), 132.8 (2CH), 126.1 (C), 125.8 (CH), 123.2 (C), 114.0 (2CH), 55.4 (CH_3), 52.9 (CH_3). **IR (neat)** v/cm^{-1}: 2117 (m), 1698 (m), 1595 (m), 1505 (m), 1244 (s), 1170 (s), 1080 (s), 817 (m), 650 (w), 547 (m). **HR-MS (TOF ES+):** calcd for $C_{11}H_{12}N_3O_3$ 234.0843 found 234.0843. In agreement with data previously reported. [1]

Methyl (*Z*)-2-azido-3-cyclohexylacrylate (1h)

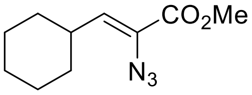

Chemical Formula: $C_{10}H_{15}N_3O_2$
Molecular Weight: 209.25

Yield: 22% (235 mg, 1.12 mmol)

Appearance: Colourless oil

^{1}H-NMR (500 MHz, CDCl$_3$) δ/ppm 6.04 (d, J = 9.6 Hz, 1H), 3.81 (s, 3H), 2.53 (m ,1H), 1.70-1.64 (m, 5H), 1.32 (m, 2H), 1,20 (tt, J = 12.3, 2.9 Hz , 1H), 1.09 (m, 2H). **^{13}C-NMR (125 MHz, CDCl$_3$)** δ/ppm 163.9 (C), 137.2 (CH), 126.3 (C), 52.9 (CH_3), 36.9 (CH), 32.1 (2CH_2), 26.2 (CH_2), 25.8 (2CH_2). **IR (neat)** v/cm^{-1}: 2926 (m), 2851 (w), 2116 (s), 1717 (s), 1631 (w) 1437 (m), 1303 (m), 1256 (s), 1223 (s), 1066 (m), 971 (w), 736 (s), 665 (w). **HR-MS (TOF ES+):** calcd for $C_{10}H_{16}N_3O_2$ 210.1198 found 210.1198.

Methyl (Z)-2-azido-3-(3-methoxyphenyl)acrylate (1i)

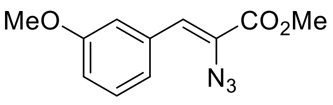

Chemical Formula: $C_{11}H_{11}N_3O_3$
Molecular Weight: 233.23

Yield: 69% (1.10g, 4.71 mmol)

Appearance: Yellow solid

Melting point: 53 °C

^{1}H-NMR (500 MHz, $CDCl_3$) δ/ppm 7.44 (m, 1H), 7.33 (m, 1H), 7.30 (t, *J* = 7.8 Hz, 1H), 6.90 (m, 1H), 6.89 (s, 1H), 3.91 (s, 3H), 3.84 (s, 3H). **^{13}C-NMR (125 MHz, $CDCl_3$)** δ/ppm 164.1 (C), 159.6 (C), 134.5 (C), 129.5 (CH), 125.7 (C), 125.6 (CH), 123.5 (CH), 115.6 (CH), 115.5 (CH), 55.4 (CH_3), 53.1 (CH_3). **IR (neat)** v/cm^{-1}: 2951 (w), 2120 (m), 1714 (s), 1619 (m), 1572 (m), 1477 (w), 1433 (m), 1297 (s), 1081 (s), 970 (w), 784 (w), 653 (w). **HR-MS (TOF ES+):** calcd for $C_{11}H_{12}N_3O_3$ 234.0873 found 234.0870. In agreement with data previously reported.[4]

Methyl (*Z*)-2-azido-3-(2,4-difluorophenyl)acrylate (1j)

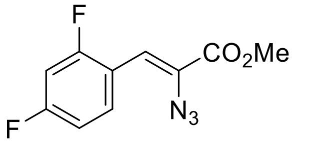

Chemical Formula: $C_{10}H_7F_2N_3O_2$
Molecular Weight: 239.1818

Yield: 73% (1.2g, 5.02 mmol)

Appearance: Light-yellow solid

Melting point: 86 °C

^{1}H-NMR (400 MHz, $CDCl_3$) δ/ppm 8.30 (m, 1H), 7.06 (s, 1H), 6.89 (m, 1H), 6.71 (m, 1H), 3.91 (s, 3H). **^{13}C-NMR (100 MHz, $CDCl_3$)** δ/ppm 163.2 (dd, *J* = 250, 12 Hz, CF), 163.8 (C), 161.1 (dd, J = 250, 12 Hz, CF), 131.9 (dd, *J* = 10, 3 Hz, CH), 126.5 (t, *J* = 2.3 Hz, C), 117.8 (dd, *J* = 10, 3Hz, CH), 115.0 (dd, *J* = 8, 2 Hz, CH), 115.8 (dd, *J* = 21, 3 Hz, CH), 103.9 (t, *J* = 26 Hz, C), 53.2 (CH_3). **^{19}F-NMR (376 MHz, $CDCl_3$) δ/ppm -**106.2 (m), -110.6 (m). **IR (neat)** v/cm^{-1}: 3092 (w), 2122 (m), 1715 (m), 1606 (m), 1585 (m), 1431 (m), 1385 (m), 1269 (s), 1238 (s), 1187 (m), 966 (m), 880 (s), 866 (s), 757 (m), 647 (w). **HR-MS (TOF ES+):** calcd for $C_{10}H_7F_2N_3O_2Na$ 262.0399 found 262.0399.

Tert-butyl (Z)-2-azido-3-phenylacrylate (1k)

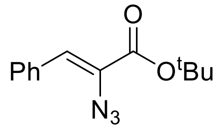

Chemical Formula: $C_{13}H_{15}N_3O_2$
Molecular Weight: 245.28

Yield: 64% (573 mg, 2.17 mmol)

Appearance: Yellow oil

^{1}H-NMR (500 MHz, $CDCl_3$) δ/ppm 7.83 (m, 2H), 7.41 (m, 2H), 7.24 (m, 1H), 6.86 (s, 1H), 1.61 (s, 3CH_3). **^{13}C-NMR (125 MHz, $CDCl_3$)** δ/ppm 164.2 (C), 133.4 (C), 130.5 (2CH), 129.1 (CH), 128.4 (2CH), 126.7 (C), 124.7 (CH), 83.4 (C), 28.1 (3CH_3). **IR (neat)** v/cm^{-1}: 2970 (w), 2111 (s), 1700 (s), 1611 (w), 1337 (m), 1268

(m), 1150 (s), 1062 (m), 878 (w), 767 (w). **HR-MS (TOF ES+):** calcd for $C_{13}H_{15}FN_3O_2Na$ 263.1503 found 263.1495.

Tert-butyl (Z)-2-azido-3-(4-fluorophenyl)acrylate (1l)

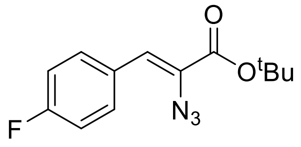

Chemical Formula: $C_{13}H_{14}FN_3O_2$
Molecular Weight: 263.27

Yield: 67% (240 mg, 1.02 mmol)

Appearance: Yellow solid

Melting point: 69 °C

^{1}H-NMR (400 MHz, CDCl$_3$) δ/ppm 7.82 (m, 2H), 7.08 (m, 2H), 6.79 (s, 1H), 1.59 (s, 9H). **^{13}C-NMR (100 MHz, CDCl$_3$)** δ/ppm 162.9 (d, *J* = 250 Hz, CF), 162.5 (C), 132.5 (d, *J* = 8 Hz, 2CH), 129.8 (d, *J* = 3 Hz, C) 126.4 (d, *J* = 3 Hz, C), 123.6 (CH), 115.6 (d, *J* = 22 Hz, 2CH), 83.7 (C), 28.2 (3CH$_3$). **^{19}F-NMR (376 MHz, CDCl$_3$) δ/ppm** -110.50 (m). **IR (neat)** v/cm^{-1}: 3009 (w), 2101 (m), 1702 (m), 1600 (w), 1583 (w), 1507 (m), 1371 (m), 1318 (w), 1149 (s), 885 (w), 761 (m). **HR-MS (TOF ES+):** calcd for $C_{13}H_{14}FN_3O_2Na$ 286.0962 found 286.0963.

Tert-butyl (Z)-2-azido-3-(4-methoxyphenyl)acrylate (1m)

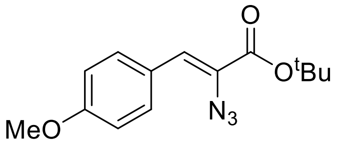

Chemical Formula: $C_{14}H_{17}N_3O_3$
Molecular Weight: 275.31

Yield: 60% (424 mg, 1.54 mmol)

Appearance: Yellow oil

^{1}H-NMR (500 MHz, CDCl$_3$) δ/ppm 7.79 (m, 2H), 6.92 (m, 2H), 6.81 (s, 1H), 3.82 (s, 3H), 1.59 (s, 9H). **^{13}C-NMR (125 MHz, CDCl$_3$)** δ/ppm 162.8 (C), 160.4 (C), 132.3 (2CH), 126.3 (C), 124.9 (CH), 124.6 (C), 114.0 (2CH), 83.2 (C), 55.4 (CH$_3$), 28.2 (3CH$_3$). **IR (neat)** v/cm^{-1}: 2976 (w), 2111 (s), 1699 (s), 1611 (w), 1447 (w), 1369 (m), 1268 (m), 1150 (s), 1082 (m), 878 (m), 639 (m). **HR-MS (TOF ES+):** calcd for $C_{14}H_{18}N_3O_3$ 276.1343 found 276.1336.

Tert-pentyl (Z)-2-azido-3-(4-fluorophenyl)acrylate (1n)

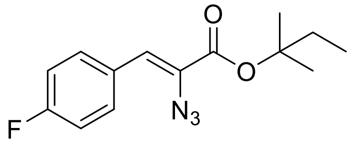

Chemical Formula: $C_{14}H_{16}FN_3O_2$
Molecular Weight: 277.30

Yield: 67% (264 mg, 1.00 mmol)

Appearance: Orange oil

^{1}H-NMR (400 MHz, CDCl$_3$) δ/ppm 7.62 (m, 2H), 7.08 (m, 2H), 6.79 (s, 1H), 1.92 (q, *J* = 7.6 Hz, 2H), 1.56 (s, 6H), 0.97 (t, *J* = 7.6 Hz, 3H). **^{13}C-NMR (125 MHz, CDCl$_3$)** δ/ppm 162.9 (d, *J* = 250 Hz, CF), 162.4 (C), 132.5 (d, *J* = 8.0 Hz, 2CH), 129.8 (d, *J* = 3 Hz, C), 126.4 (d, *J* = 3 Hz, C), 123.4 (CH), 115.6 (d, *J* = 21 Hz, 2CH), 86.3 (C), 33.6 (CH$_2$), 25.7 (2CH$_3$), 8.5 (CH$_3$). **^{19}F-NMR (376 MHz, CDCl$_3$) δ/ppm** -110.4 (m). **IR**

(neat) v/cm^{-1}: 2976 (w), 2105 (s), 1704 (s), 1620 (w), 1600 (m), 1506 (m), 1460 (w), 1368 (m), 1264 (m), 1077 (m), 829 (s), 640 (w). **HR-MS (TOF ES+):** calcd for $C_{13}H_{16}FN_3O_2$ 278.1299 found 278.1292.

Characterisation of 2*H*-Azirines

Reported Yields are ^{1}H-NMR yields due to unstable to column chromatography.

Methyl 2-phenyl-2H-azirine-3-carboxylate (2a)

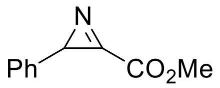

Chemical Formula: $C_{10}H_9NO_2$
Molecular Weight: 175.19

Synthesised by Procedure **D**

NMR Yield: 95% (43 mg, 0.25 mmol)

Appearance: Yellow oil

^{1}H-NMR (400 MHz, CDCl$_3$) δ/ppm 7.34-7.32 (m, 3H), 7.15-7.13 (m, 2H), 4.02 (s, 3H), 3.48 (s, 1H). **^{13}C-NMR (100 MHz, CDCl$_3$)** δ/ppm 163.5 (C), 159.5 (C), 138.6 (C), 128.9 (2CH), 128.6 (CH), 126.9 (2CH), 54.1 (CH$_3$), 39.3 (CH). **HR-MS (TOF ES+):** calcd for found $C_{10}H_{10}NO_2$ (M+H$^+$) calcd for 176.0706 found 176.0705 (M+H$^+$).

Methyl 2-(4-fluorophenyl)-2H-azirine-3-carboxylate (2b)

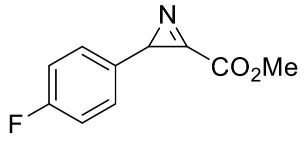

Chemical Formula: $C_{10}H_8FNO_2$
Molecular Weight: 193.18

Synthesised by Procedure **D**

NMR Yield: 76% (23 mg, 0.12 mmol)

Appearance: Yellow oil

^{1}H-NMR (400 MHz, CDCl$_3$) δ/ppm 7.12-7.09 (m, 2H), 7.05-6.99 (m, 2H), 4.03 (s, 3H), 3.46 (s, 3H). **^{13}C-NMR (100 MHz, CDCl$_3$)** δ/ppm 164.1 (d, *J* = 245 Hz, CF), 163.4 (C), 159.1 (C), 134.1 (d, *J* = 3 Hz, C), 128.3 (d, *J* = 9 Hz, 2CH), 115.8 (d, *J* = 22 Hz, 2CH), 53.9 (CH$_3$), 38.3 (CH). **^{19}F-NMR (376 MHz, CDCl$_3$)** δ/ppm -113.6 (m). **IR (neat)** v/cm^{-1}: 1754 (s), 1719 (s), 1509 (s), 1328 (m), 1306 (m), 1215 (s), 1098 (w), 964 (w), 836 (s), 775 (w), 536 (m). **HR-MS (TOF ES+):** calcd for $C_{10}H_9FNO_2$ 194.0573 found 194.0570.

Methyl 2-(4-bromophenyl)-2H-azirine-3-carboxylate (2c)

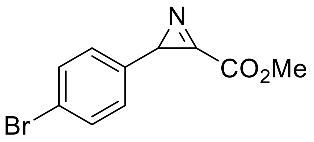

Chemical Formula: $C_{10}H_8BrNO_2$
Molecular Weight: 254.08

Synthesised by Procedure **D**

NMR Yield: 77% (23 mg, 0.09 mmol)

Appearance: Yellow oil

^{1}H-NMR (400 MHz, CDCl$_3$) δ/ppm 7.46 (m, 2H), 7.01 (m, 2H), 4.03 (s, 3H), 3.43 (s, 1H). **^{13}C-NMR (100 MHz, CDCl$_3$)** δ/ppm 163.0 (C), 158.9 (C), 137.4 (C), 131.8 (2CH), 128.2 (2CH), 122.3 (C), 53.9 (CH$_3$), 38.4 (CH). **IR (neat)** v/cm^{-1}: 1753 (s), 1717 (s),

1488 (m), 1434 (m), 1295 (m), 1268 (m), 1220 (s), 1166 (s), 1070 (s), 1008 (s), 829 (s), 737 (w), 523 (w). **HR-MS (TOF ES+):** calcd for $C_{10}H_9BrNO_2$ 254.9718 found 254.9718. In agreement with data previously reported.[5]

Methyl 2-(*p*-tolyl)-2H-azirine-3-carboxylate (2d)

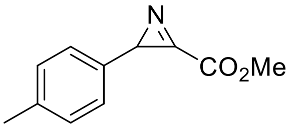

Chemical Formula: $C_{11}H_{11}NO_2$
Molecular Weight: 189.21

Synthesised by Procedure **E**

NMR Yield: 65% (18 mg, 0.01 mmol)

Appearance: Yellow oil

^{1}H-NMR (400 MHz, CDCl$_3$) δ/ppm 7.15 (d, *J* = 7.8 2H) 7.04 (d, *J* = 6.2 Hz, 2H), 4.02 (s, 3H), 3.46 (s, 1H), 2.34 (s, 3H). **^{13}C-NMR (100 MHz, CDCl$_3$)** δ/ppm 163.2 (C), 159.0 (C), 137.8 (C), 135.0 (C), 129.0 (2CH), 126.2 (2CH), 53.4 (CH_3), 38.6 (CH), 21.0 (CH_3). **IR (neat)** ν/cm^{-1}: 2120 (w), 1752 (s), 1715 (s), 1516 (m), 1434 (m), 1321 (w), 1221 (s), 1166 (s), 961 (w), 819 (s), 718 (w), 535 (w). **HR-MS (TOF ES+):** calcd for $C_{11}H_{12}NO_2$ 190.0863 found 190.0863 ($M+H^+$).

Methyl 2-(3,4-dimethylphenyl)-2H-azirine-3-carboxylate (2e)

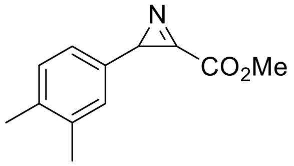

Chemical Formula: $C_{12}H_{13}NO_2$
Molecular Weight: 203.24

Synthesised by Procedure **E**

Yield: 72% (20 mg, 0.01 mmol)

Appearance: Yellow oil

^{1}H-NMR (400 MHz, CDCl$_3$) δ/ppm 7.10 (d, *J* = 8.8 Hz, 1H), 6.88 (m, 2H), 4.02 (s, 3H), 3.43 (s, 1H), 2.24 (s, 6H). **^{13}C-NMR (100 MHz, CDCl$_3$)** δ/ppm 163.5 (C), 159.3 (C), 136.9 (C), 136.8 (C), 135.7 (C), 129.9 (CH), 127.7 (CH), 124.1 (CH), 53.7 (CH_3), 39.0 (CH), 19.9 (CH_3), 19.6 (CH_3). **IR (neat)** ν/cm^{-1}: 1752 (s), 1715 (s), 1504 (w), 1435 (s), 1211 (m), 1215 (s), 1177 (s), 1002 (w), 964 (w), 822 (m), 769 (w). **HR-MS (TOF ES+):** calcd for $C_{12}H_{14}NO_2$ 204.0980 found 204.0980.

Methyl 2-(o-tolyl)-2H-azirine-3-carboxylate (2f)

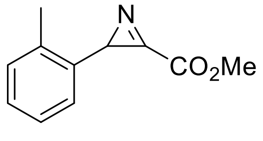

Chemical Formula: $C_{11}H_{11}NO_2$
Molecular Weight: 189.21

Synthesised by Procedure **E**

NMR Yield: 77% (18 mg, 0.01 mmol)

Appearance: Yellow oil

^{1}H-NMR (400 MHz, CDCl$_3$) δ/ppm 7.15-7.11 (m, 3H), 6.79 (d, *J* = 8.5 Hz, 1H), 4.03 (s, 3H), 3.59 (s, 1H), 2.55 (3H). **^{13}C-NMR (100 MHz, CDCl$_3$)** δ/ppm 164.9 (C),

159.2 (C), 137.3 (C), 136.2 (C), 130.4 (CH), 128.0 (CH), 126.2 (CH), 125.5 (CH), 53.8 (CH_3), 36.4 (CH), 19.3 (CH_3). **IR (neat)** ν/cm^{-1}: 2118 (m), 1754 (s), 1715 (s), 1434 (m), 1304 (m), 1230 (s), 1202 (s), 1169 (s), 961 (m), 760 (m), 724 (m). **HR-MS (TOF ES+):** calcd for $C_{11}H_{12}NO_2$ 190.0863 found 190.0863 (M+H$^+$).

Methyl 2-cyclohexyl-2H-azirine-3-carboxylate (2h)

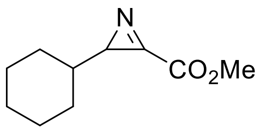

Chemical Formula: $C_{10}H_{15}NO_2$
Molecular Weight: 181.24

Synthesised by Procedure **F**

NMR Yield: 74% (23 mg, 0.126 mmol)

Appearance: Yellow oil

^{1}H-NMR (400 MHz, CDCl$_3$) δ/ppm 3.98 (s, 3H), 2.33 (d, *J* = 4.1 Hz, 1H), 1.73-1.69 (m, 3H), 1.62-1.59 (m, 2H), 1.49 (m, 1H) 1.22-1.15 (m, 3H), 1.01-0.89 (m, 2H). **^{13}C-NMR (100 MHz, CDCl$_3$)** δ/ppm 171.2 (C), 160.1 (C), 53.5 (CH_3), 42.2 (CH), 39.3 (CH), 30.6 (CH_2), 30.5 (CH_2), 26.1 (CH_2), 25.8 ($2CH_2$). **IR (neat)** ν/cm^{-1}: 2923 (m), 2851 (m), 1750 (s), 1733 (s), 1666 (s), 1448 (m), 1313 (m), 1215 (s), 974 (w), 751 (w). **HR-MS (TOF ES+):** calcd for found $C_{10}H_{16}NO_2$ 182.1176 found 182.1176 (M+H$^+$).

Characterisation of 1,3-diazabicyclo[3.1.0]hex-3-ene-4,5-dicarboxylates

Note

Low yield for isomers which can be separated to ensure clean fractions.

(+/-)-Dimethyl (2S,5S,6R)-2,6-diphenyl-1,3-diazabicyclo[3.1.0]hex-3-ene-4,5-dicarboxylate (3a)

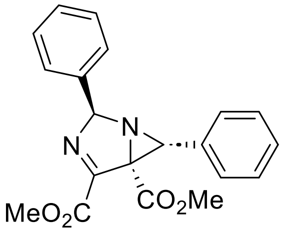

Chemical Formula: $C_{20}H_{18}N_2O_4$
Molecular Weight: 350.37

Yield: 24% (35 mg, 0.1 mmol)

Appearance: Yellow oil

^{1}H-NMR (500 MHz, CDCl$_3$) δ/ppm 7.52 (m, 2H), 7.39 (m, 2H) 7.36-7.26 (m, 6H), 6.92 (s, 1H), 4.01 (s, 3H), 3,56 (s, 3H), 3.01 (s, 1H). **^{13}C-NMR (125 MHz, CDCl$_3$)** δ/ppm 165.2 (C), 163.9 (C), 162.1 (C), 136.2 (C), 133.3 (C), 128.8 (2CH), 128.4 (CH), 128.3 (CH), 128.1 (2CH), 128.1 (2CH), 127.3 (2CH), 96.3 (CH), 65.7 (C), 53.4 (CH_3), 52.7 (CH_3), 49.7 (CH). **IR (neat)** ν/cm^{-1}: 1735 (s), 1727 (s), 1620 (w), 1437 (m), 1336 (m), 1201 (s), 1156 (s), 1093 (m), 966 (m), 915 (m), 813 (w), 730 (s), 697 (s). **HR-MS (TOF ES+):** calcd for found $C_{20}H_{19}N_2O_4$ 351.1337 found 351.1337 (M+H$^+$).

(+/-)-Dimethyl (2R,5S,6R)-2,6-diphenyl-1,3-diazabicyclo[3.1.0]hex-3-ene-4,5-dicarboxylate (3a')

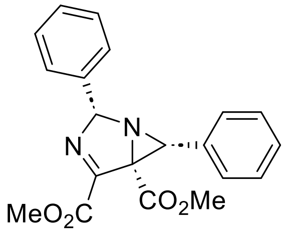

Chemical Formula: $C_{20}H_{18}N_2O_4$
Molecular Weight: 350.37

Yield: 56% (82 mg, 0.23 mmol)

Appearance: Yellow oil

^{1}H-NMR (500 MHz, $CDCl_3$) δ/ppm 7.57 (m, 2H), 7.47-7.41 (m, 4H), 7.38-7.31 (m, 4H), 6.20 (s, 1H), 3.96 (s, 3), 3.58 (s, 3H), 3.15 (s, 1H). **^{13}C-NMR (125 MHz, $CDCl_3$)** δ/ppm 165.5 (C), 162.5 (C), 161.0 (C), 138.8 (C), 133.9 (C), 129.1 (2CH), 128.6 (2CH), 128.4 (2CH), 128.2 (2CH), 127.3 (2CH), 98.5 (CH), 65.3 (C), 54.9 (CH), 53.7 (CH_3), 52.9 (CH_3). **IR (neat)** ν/cm^{-1}: 1727 (s), 1620 (w), 1437 (m), 1336 (m), 1201 (s), 1156 (s), 1093 (m), 966 (w), 915 (w), 730 (m), 697 (s). **HR-MS (TOF ES+):** calcd for $C_{20}H_{19}N_2O_4$ 351.1337 found 351.1337 ($M+H^+$).

(+/-)-Dimethyl (2S,5S,6R)-2,6-bis(4-fluorophenyl)-1,3-diazabicyclo[3.1.0]hex-3-ene-4,5-dicarboxylate (3b)

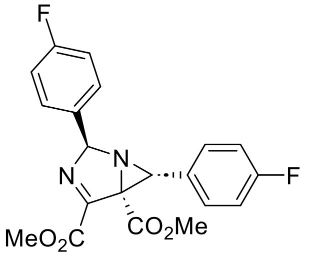

Chemical Formula: $C_{20}H_{16}F_2N_2O_4$
Molecular Weight: 386.35

Yield: 40% (41 mg, 0.11 mmol)

Appearance: Yellow oil

^{1}H-NMR (400 MHz, $CDCl_3$) δ/ppm 7.46 (m, 2H), 7.36 (m, 2H), 7.00 (m, 4H), 6.86 (s, 1H), 4.00 (s, 3H), 3.59 (s, 3H), 2.91(s, 1H). **^{13}C-NMR (100 MHz, $CDCl_3$)** δ/ppm 164.8 (C), 163.9 (C), 163.9 (d, *J* = 246 Hz, CF), 163.8 (d, *J* = 246 Hz, CF) 161.8 (C), 131.8 (d, *J* = 4 Hz, C), 129.6 (dd, *J* = 8, 2CH), 129.0 (dd, *J* = 8 , 2CH), 128.6 (d, *J* = 4 Hz, C), 115.8 (d, *J* = 22 Hz, 2CH), 115.2 (d, *J* = 22 Hz, 2CH), 95.5 (CH), 65.6 (C), 53.3 (CH), 52.6 (CH_3), 48.8 (CH_3). **^{19}F-NMR (376 MHz, $CDCl_3$) δ/ppm** -113.2(m) -113.4 (m). **IR (neat)** ν/cm^{-1}: 1750 (m), 1731 (m), 1606 (m), 1438 (w), 1333 (m), 1221 (m), 1152 (s), 1092 (s), 969 (m), 835 (s), 735 (s), 702 (w), 553 (w). **HR-MS (TOF ES+):** calcd for $C_{20}H_{17}F_2N_2O_4$ 387.1151 found 387.1151 ($M+H^+$).

(+/-)-Dimethyl (2R,5S,6R)-2,6-bis(4-fluorophenyl)-1,3-diazabicyclo[3.1.0]hex-3-ene-4,5-dicarboxylate (3b')

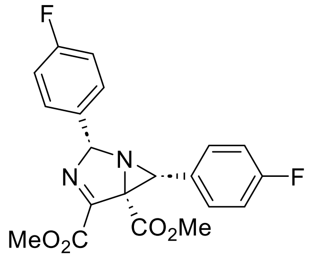

Chemical Formula: $C_{20}H_{16}F_2N_2O_4$
Molecular Weight: 386.35

Yield: 27% (0.07 mmol)

Appearance: Yellow oil

^{1}H-NMR (400 MHz, CDCl$_3$) δ/ppm 7.53 9m, 2H), 7.43 (m, 2H), 7.11 (m, 2H), 7.03 (m, 2H), 6.17 (s, 1H), 3.96 (s, 3H), 3.59 (s, 3H), 3.11 (s, 1H). **^{13}C-NMR (100 MHz, CDCl$_3$)** δ/ppm 165.4 (C), 162.6 (C), 161.9 (C), 164.7 (d, *J* = 246 Hz, CF), 164.4 (d, *J* = 246 Hz, CF), 134.7 (d, *J* = 4 Hz, C), 130.0 (d, J = 8 Hz, 2CH), 129.6 (d, *J* = 4 Hz, C), 129.2 (d, *J* = 8 Hz, 2CH), 116.3 (d, *J* = 22 Hz, 2CH), 115.7 (d, *J* = 22 Hz, 2CH), 97.9 (CH), 54.3 (CH), 53.8 (CH$_3$), 53.1 (CH$_3$). **^{19}F-NMR (376 MHz, CDCl$_3$) δ/ppm** -113.0 (m), -113.4 (m). **IR (neat)** v/cm^{-1}: 1736 (s), 1730 (s), 1603 (m), 1509 (s), 1439 (m), 1226 (s), 1155 (s), 1083 (s), 1036 (m), 963 (w), 837 (w), 810 (w). **HR-MS (TOF ES+):** calcd for $C_{20}H_{17}F_2N_2O_4$ 387.1151 found 387.1151 (M+H$^+$).

(+/-)-Dimethyl (2S,5S,6R)-2,6-bis(4-bromophenyl)-1,3-diazabicyclo[3.1.0]hex-3-ene-4,5-dicarboxylate (3c)

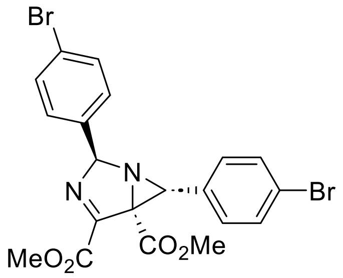

Chemical Formula: $C_{20}H_{16}Br_2N_2O_4$
Molecular Weight: 508.17

Yield: 26% (24 mg, 0.05 mmol)

Appearance: Yellow oil

^{1}H-NMR (500 MHz, CDCl$_3$) δ/ppm 7.47-7.43 (m, 4H), 7.35 (m, 2H), 7.26 (m, 2H), 6.82 (CH), 4.00 (s, 3H), 3.59 (s, 3H), 2.84 (CH). **^{13}C-NMR (125 MHz, CDCl$_3$)** δ/ppm 165.1 (C), 164.4 (C), 162.0 (C), 135.3 (C), 132.4 (2CH), 132.2 (C), 131.7 (2CH), 130.0 (2CH), 129.2 (2CH), 123.1 (C), 129.2 (C), 96.1 (CH), 66.0 (C), 53.8 (CH$_3$), 53.2 (CH$_3$), 49.3 (H). **IR (neat)** v/cm^{-1}: 1749 (s), 1730 (s), 1487 (m(, 1438 (m), 1338 (m), 1204 (s), 1156 (s), 1083 (m), 1010 (s), 814 (m), 769 (m). **HR-MS (TOF ES+):** calcd for $C_{20}H_{17}Br_2N_2O_4$ 508.9530 found 508.9529 (M+H$^+$).

(+/-)-Dimethyl (2R,5S,6R)-2,6-bis(4-bromophenyl)-1,3-diazabicyclo[3.1.0]hex-3-ene-4,5-dicarboxylate (3c′)

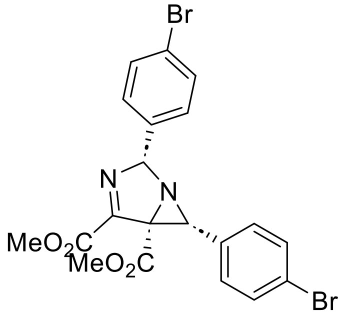

Chemical Formula: $C_{20}H_{16}Br_2N_2O_4$
Molecular Weight: 508.17

Yield: 33% (31 mg, 0.06 mmol)

Appearance: Yellow oil

^{1}H-NMR (400 MHz, CDCl$_3$) δ/ppm 7.56 (m, 2H), 7.48-7.43 (m, 4H), 7.33 (m, 2H), 6.15 (s, 1H), 3.96 (s, 3H), 3.59 (s, 3H), 3.08 (s, 1H). **^{13}C-NMR (125 MHz, CDCl$_3$)** δ/ppm 165.2 (C), 162.7 (C), 161.7 (C), 132.8 (C), 132.4 (2CH), 131.7 (2CH), 129.9 (2CH), 128.7 (2CH),128.7 (C), 123.5 (C), 123.0 (C), 97.8 (CH), 65.3 (C), 54.2 (CH), 53.8 (CH$_3$), 53.2 (CH$_3$). **IR (neat)** v/cm^{-1}: 1749 (s), 1730 (s), 1500 (m), 1487 (m), 1400 (m), 1204 (s), 1156 (s), 1000 (s), 850 (m) 750 (w). **HR-MS (TOF ES+):** calcd for $C_{20}H_{17}Br_2N_2O_4$ 508.9530 found 508.9529 (M+H$^+$).

(+/-)-Dimethyl (2S,5S,6R)-2,6-di-p-tolyl-1,3-diazabicyclo[3.1.0]hex-3-ene-4,5-dicarboxylate (3d)

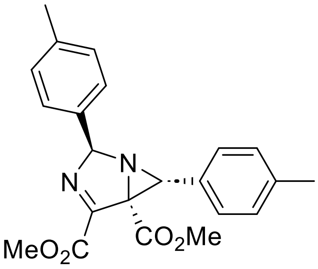

Chemical Formula: $C_{22}H_{22}N_2O_4$
Molecular Weight: 378.43

Yield: 26% (17 mg, 0.04 mmol)

Appearance: Yellow oil

^{1}H-NMR (500 MHz, CDCl$_3$) δ/ppm 7.36 (d, *J* = 8.4 Hz, 2H), 7.28 (d, *J* = 5.5 Hz, 2H), 7.12 (t, *J* = 8.4 Hz, 4H), 6.87 (s, 1H), 4.00 (s, 3H), 3.59 (s, 3H), 2.98 (s, 1H), 2.32 (s, 3H), 2.30 (s, 3H). **^{13}C-NMR (125 MHz, CDCl$_3$)** δ/ppm 165.0 (C), 163.6 (C), 162.0 (C), 137.9 (C), 137.8 (C), 133.0 (C), 130.0 (C), 129.2 (2CH), 128.6 (2CH), 127.7 (2CH), 127.0 (2CH), 95.9 (CH), 65.4 (C), 53.1 (CH$_3$), 52.4 (CH$_3$), 49.5 (CH),21.1 (CH$_3$), 21.0 (CH$_3$). **IR (neat)** v/cm^{-1}: 1748 (s), 1728 (s), 1514 (w), 1437 (m), 1338 (m), 1200 (s), 1153 (s), 1081 (s), 1020 (m), 962 (m), 825 (m), 799 (m). **HR-MS (TOF ES+):** calcd for found $C_{22}H_{23}N_2O_4$ 379.1652 found for 379.1659 (M+H$^+$).

(+/-)-Dimethyl (2R,5S,6R)-2,6-di-p-tolyl-1,3-diazabicyclo[3.1.0]hex-3-ene-4,5-dicarboxylate (3d')

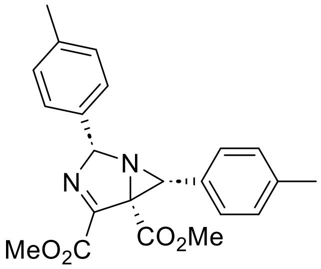

Chemical Formula: $C_{22}H_{22}N_2O_4$
Molecular Weight: 378.43

Yield: 50% (32 mg, 0.08 mmol)

Appearance: Yellow oil

^{1}H-NMR (500 MHz, $CDCl_3$) δ/ppm 7.44 (d, *J* = 6.1 Hz, 2H), 7.34 (d, *J* = 8.1 Hz, 2H), 7.22 (d, *J* = 8.1 Hz, 2H), 7.14 (d, *J* = 7.8 Hz, 2H), 6.15 (s, 1H), 3.95 (s, 3H), 3.59 (s, 3H), 3.09 (s. 1H), 2.37 (s, 3H), 2.34 (s, 3H). **^{13}C-NMR (125 MHz, $CDCl_3$)** δ/ppm 165.6 (C), 162.4 (C), 162.1 (C), 138.9 (C), 138.4 (C), 136.1 (C), 130.9 (C), 129.8 (2CH), 129.1 (2CH), 128.1 (2CH), 127.2 (2CH), 98.4 (CH), 66.2 (C), 55.1 (CH), 53.6 (CH_3), 52.9 (CH_3), 21.6 ($2CH_3$). **IR (neat)** ν/cm^{-1}: 1749 (s), 1729 (s), 1617 (w) 1514 (w), 1428 (m), 1333 (m), 1310 (m), 1200 (s), 1156 (s), 1092 (s), 1035 (w), 1017 (w), 842 (m), 781 (m), 755 (m). **HR-MS (TOF ES+):** calcd for $C_{22}H_{23}N_2O_4$ 379.1654 found 379.1654 ($M+H^+$).

(+/-)-Dimethyl (5S,6R)-2,6-bis(3,4-dimethylphenyl)-1,3-diazabicyclo[3.1.0]hex-3-ene-4,5-dicarboxylate (3e/3e')

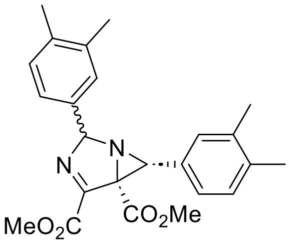

Chemical Formula: $C_{24}H_{26}N_2O_4$
Molecular Weight: 406.48

Yield: 95% (98 mg, 0.24 mmol)

Appearance: Yellow oil

^{1}H-NMR (500 MHz, $CDCl_3$) δ/ppm 7.29-7.06 (m, 12H,), 6.85 (s, 1H,), 6.11 (s, 1H), 4.01 (s, 3H), 3.95 (s, 3H), 3.60 (s, 3H), 3.59 (s, 3H), 3.05 (s, 1H), 2.99 (s, 1H), 2.30-2.24 (s, 12H), 2.22-2.20 (s, 12H). **^{13}C-NMR (125 MHz, $CDCl_3$)** δ/ppm 165.6 (C), 165.5 (C), 164.1 (C), 162.6 (C), 162.4 (C), 162.1 (C), 137.6 (C), 137.3 (C), 137.2 (C), 137.0 (C), 137.0 (C), 136.9 (C), 136.6 (C), 136.6 (C), 136.4 (C), 133.8 (C), 131.4 (C), 130.9 (C),130.3 (CH), 130.3 (CH), 129.7 (2CH), 129.5 (CH), 129.3 (CH), 128.9 (CH), 128.5 (CH), 125.6 (CH), 125.5 (CH), 124.9 (CH), 1124.6 (CH), 98.5 (CH), 96.4 (CH), 65.9 C), 65.2 (C), 55.2 (CH), 53.6 ($2CH_3$,), 52.9 ($2CH_3$), 50.1 (CH), 20.1 ($8CH_3$). **IR (neat)** ν/cm^{-1}: 2966 (w), 2921 (w), 1571 (s), 1749 (s), 1734 (s), 1608 (w), 1437 (s), 1333 (m), 1271 (m), 1201 (s), 1158 (s), 1085 (m), 969 (w), 809 (m), 737 (m). **HR-MS (TOF ES+):** calcd for $C_{24}H_{27}N_2O_4$ 407.1965 found 407.1965 ($M+H^+$).

(+/-)-Dimethyl (5S,6R)-2,6-di-o-tolyl-1,3-diazabicyclo[3.1.0]hex-3-ene-4,5-dicarboxylate (3f/3f')

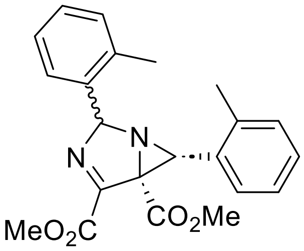

Chemical Formula: $C_{22}H_{22}N_2O_4$
Molecular Weight: 378.43

Yield: 71% (75 mg, 0.20 mmol)

Appearance: Yellow oil

^{1}H-NMR (500 MHz, CDCl$_3$) δ/ppm 7.58 (m, 1H), 7.40 (m, 1H), 7.35 (m, 1H), 7.22-7.08 (m, 12H), 6.98 (d, *J* = 7.7 Hz, 1H), 6.92 (s, 1H), 6.33 (s, 1H), 4.04 (s, 3H), 3.99 (s, 3H'), 3.53 (s, 3H), 3.51 (s, 3H), 3.20 (s, 1H), 2.95 (s, 1H), 2.75 (s, 3H), 2.60 (s, 3H), 2.44 (s, 3H), 2.31 (s, 3H). **^{13}C-NMR (125 MHz, CDCl$_3$)** δ/ppm 165.6 (C), 165.6 (C) 164.8 (C), 163.4 (C), 162.2 (C), 162.0 (C), 137.0 (C), 136.9 (C), 136.8 (C), 137.8 (C), 135.7 (C), 134.2 (C), 132.1 (C), 132.0 (C), 130.8 (CH), 130.7 (CH), 129.7 (CH),129.5 (CH), 129.0 (CH), 128.8 (CH), 128 2 (CH), 128.0 (CH), 127.7 (CH), 127.4 (CH), 126.6 (2CH),125.9 (CH), 125.7 (CH), 125.6 (CH), 95.5 (CH), 94.3 (CH), 64.2 (C), 64.1 (C), 54.5 (CH), 53.4 (2CH$_3$), 52.7 (2CH$_3$), 47.5 (CH), 20.0 (CH$_3$), 19.8 (CH3), 19.0 (CH$_3$), 18.9 (CH$_3$). **IR (neat)** v/cm^{-1}: 2953 (w), 2917 (w), 1756 (s), 1727 (s), 1628 (w), 1488 (m), 1374 (w), 1319 (w), 1207 (s), 1177 (s), 1088 (m), 821 (w), 747 (m). **HR-MS (TOF ES+):** calcd for $C_{22}H_{23}N_2O_4$ 379.1652 found 379.1652 (M+H$^+$).

Compounds 3g could not be isolated cleanly.

(+/-)-Dimethyl (2R,5S,6R)-2,6-bis(4-methoxyphenyl)-1,3-diazabicyclo[3.1.0]hex-3-ene-4,5-dicarboxylate (3g')

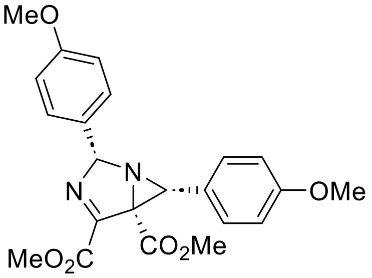

Chemical Formula: $C_{22}H_{22}N_2O_6$
Molecular Weight: 410.43

Yield: 32% (46 mg, 0.11 mmol)

Appearance: Yellow oil

^{1}H-NMR (500 MHz, CDCl$_3$) δ/ppm 7.46 (m, 2H), 7.37 (m, 2H), 6.95 (m, 2H), 6.86 (m, 2H), 6.12 (s, 1H), 3.94 (s, 3H), 3.82 (s, 3H), 3.80 (s, 3H), 3.59 (s, 3H), 3.07 (s, 1H). **^{13}C-NMR (125 MHz, CDCl$_3$)** δ/ppm 165.3 (C), 161.9 (C), 161.7 (C), 159.9 (C), 159.6 (C), 130.9 (C), 129.0 (2CH), 129.1 (2CH), 125.7 (C), 114.1 (2CH), 113.5 (2CH), 97.7 (CH), 64.8 (C), 55.3 (CH$_3$), 55.2 (CH$_3$), 54.6 (CH), 53.2 (CH$_3$), 52.5 (CH$_3$). **IR (neat)** v/cm^{-1}: 3003 (w), 2837 (w), 1749 (m), 1729 (m), 1601 (m), 1489 (m), 1454 (m), 1434 (m), 1337 (m), 1320 (m), 1202 (m), 1150 (s), 1088 (m), 967 (w), 910 (w), 777 (m), 728 (s), 682 (m). **HR-MS (TOF ES+):** calcd for $C_{22}H_{23}N_2O_6$ 411.1554 found 411.1551.

Compound 3h could not be isolated cleanly.

(+/-)-Dimethyl (2R,5S,6R)-2,6-bis(2-(trifluoromethyl)phenyl)-1,3-diazabicyclo[3.1.0]hex-3-ene-4,5-dicarboxylate (3h')

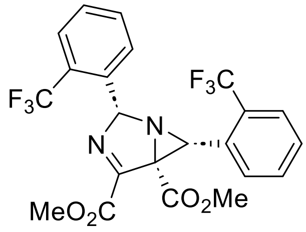

Chemical Formula: $C_{22}H_{16}F_6N_2O_4$
Molecular Weight: 486.37

Yield: 7% (26 mg, 0.05 mmol)

Appearance: Yellow oil

^{1}H-NMR (400 MHz, CDCl$_3$) δ/ppm 7.77 (m, 2H), 7.67 (m, 2H), 7.62 (m, 2H), 7.51 (m, 2H), 7.42 (m, 2H), 6.56 (s, 1H), 4.01 (s, 3H), 3.52 (s, 3H), 3.51 (s, 1H). **^{13}C-NMR (125 MHz, CDCl$_3$)** δ/ppm 165.4 (C), 164.1 (C), 160.9 (C), 136.3 (C), 132.8 (2CH), 131.9 (2CH), 129.6 (CH), 129.3 (CH), 128.8 (CH), 128.7 (q, *J* = 30 Hz, C), 128.4 (CH), 127.8 (q, *J* = 30 Hz, C), 126.2 (q, *J* = 5 Hz, C), 125.7 (q, *J* = 5 Hz, C), 123.0 (C), 93.8 (q, *J* = 2 Hz, CH), 64.7 (C), 53.5 (CH$_3$), 52.8 (CH$_3$), 52.5 (q, *J* = 2 Hz, CH). **^{19}F-NMR (376 MHz, CDCl$_3$) δ/ppm** -57.3 (s), -60.2 (s).**HR-MS (TOF ES+):** calcd for $C_{22}H_{17}F_6N_2O_4$ 487.1087 found 487.1088.

(+/-)-Dimethyl (5S,6R)-2,6-bis(2,4-difluorophenyl)-1,3-diazabicyclo[3.1.0]hex-3-ene-4,5-dicarboxylate (3i/3i')

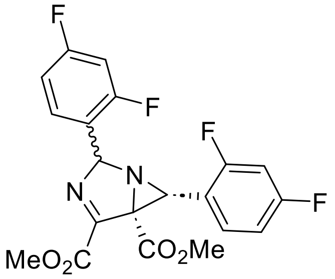

Chemical Formula: $C_{20}H_{14}F_4N_2O_4$
Molecular Weight: 422.34

Mixture of diastereomers

Yield: 99% (405 mg, 0.95 mmol)

Appearance: Yellow oil

^{1}H-NMR (600 MHz, CDCl$_3$) δ/ppm 7.48 (m, 1H), 7.42 (m, 2H), 7.16 (m, 1H), 6.95-6.82 (m, 8H), 6.72 (m, 1H) ,6.40 (s, 1H), 4.02 (s, 3H), 3.98 (s, 3H), 3.61 (s, 3H), 3.60 (s, 3H), 3.27 (s, 1H), 2.96 (s, 1H). **^{13}C{^{19}F}-NMR (100 MHz, CDCl$_3$)** δ/ppm 165.4 (C), 165.1 (C), 164.9 (C), 163.6 (C), 163.3 (C), 163.1 (C), 163.0 (C), 161.7 (C), 161.6 (C), 161.3 (C), 161.1 (C), 160.5 (C), 160.5 (C), 160.2 (C), 130.7 (CH), 130.3 (CH), 130.1 (CH), 129.7 (CH), 121.9 (C), 119.1 (C), 117.2 (C), 117.0 (C), 112.0 (CH), 111.5 (CH), 111.2 (CH), 112.2 (CH), 104.8 (CH), 104.4 (CH), 103.6 (CH), 103.4 (CH), 92.0 (CH), 91.2 (CH), 64.2 (C), 64.1 (C), 53.9 (2CH$_3$), 53.0 (CH$_3$), 52.9 (CH$_3$), 49.1 (CH), 43.1 (CH$_3$). **^{19}F-NMR (376 MHz, CDCl$_3$) δ/ppm** -107.9 (m), -108.3 (m), -109.3 (m), -112.2 (m), -114.1 (m), -114.4 (m). **IR (neat)** v/cm^{-1}: 1753 (m), 1731 (m), 1617 (m), 1504 (s), 1392 (w), 1312 (m), 1273 (m), 1203 (m), 1160 (s), 1092 (m), 963 (s), 848 (s), 749 (w), 613 (w). **HR-MS (TOF ES+):** calcd for $C_{20}H_{15}F_4N_2O_4$ 423.0966 found 423.0962.

(+/-)-Dimethyl (2S,5S,6R)-2,6-bis(3-methoxyphenyl)-1,3-diazabicyclo[3.1.0]hex-3-ene-4,5-dicarboxylate (3j)

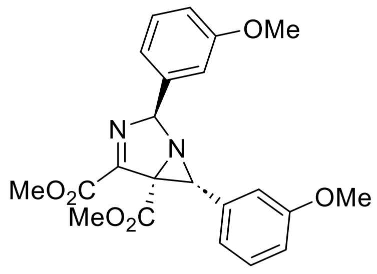

Chemical Formula: $C_{22}H_{22}N_2O_6$
Molecular Weight: 410.43

Yield: 33% (19 mg, 0.05 mmol)

Appearance: Yellow oil

^{1}H-NMR (500 MHz, $CDCl_3$) δ/ppm 7.25 (m, 2H), 7.07 (m, 2H), 6.97 (m, 2H), 6.87 (s, 1H), 6.81 (m, 2H), 4.00 (s, 3H), 3.79 (s, 3H), 3.73 (s, 3H), 3.59 (s, 3H). **^{13}C-NMR (125 MHz, $CDCl_3$)** δ/ppm 165.1 (C), 163.9 (C), 162.1 (C), 159.9 (C), 159.5 (C), 137.6 (C), 134.7 (C), 129.9 (C), 129.2 (C), 120.5 (CH), 119.6 (CH), 114.3 (CH), 114.1 (CH), 113.3 (CH), 112.7 (CH), 96.2 (CH), 65.7 (C), 55.4 ($2CH_3$), 53.4 (CH_3), 52.8 (CH_3), 49.9 (CH). **IR (neat)** v/cm^{-1}: 3003 (w), 2837 (w), 1749 (m), 1729 (m), 1601 (m), 1489 (m), 1454 (m), 1434 (m), 1337 (m), 1320 (m), 1202 (m), 1150 (s), 1088 (m), 967 (w), 910 (w), 777 (m), 728 (s), 682 (m). **HR-MS (TOF ES+):** calcd for $C_{22}H_{23}N_2O_6$ 411.1554 found 411.1551.

(+/-)-Dimethyl (2R,5S,6R)-2,6-bis(3-methoxyphenyl)-1,3-diazabicyclo[3.1.0]hex-3-ene-4,5-dicarboxylate (3j')

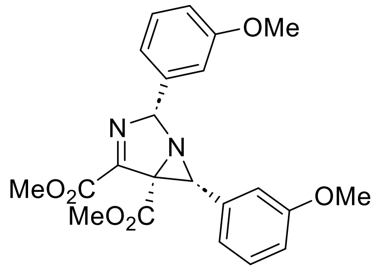

Chemical Formula: $C_{22}H_{22}N_2O_6$
Molecular Weight: 410.43

Yield: 33% (27mg, 0.06 mmol)

Appearance: Yellow oil

^{1}H-NMR (500 MHz, $CDCl_3$) δ/ppm 7.33 (m, 1H), 7.26 (m, 1H)m 7.16 (m, 1H0, 7.04 (m, 1H), 6.92 (m, 1H), 6.85 (m, 1H), 6.16 (s, 1H), 3.95 (s, CH_3), 3.84 (s, CH_3), 3.80 (s, CH_3), 3.59 (s, CH_3), 3.08 (s, 1H). **^{13}C-NMR (125 MHz, $CDCl_3$)** δ/ppm 165.1 (C), 162.1 (C), 161.6 (C), 160.0 (C), 159.5 (C), 140.0 (C), 135.2 (C), 129.9 (CH), 129.2 (CH), 120.3 (CH), 119.4 (CH), 114.7 (CH), 114.2 (CH), 113.2 (CH), 112.4 (CH), 98.2 (CH), 65.1 (C), 55.4 ($2CH_3$), 54.4 (CH), 53.4 (CH_3), 52.7 (CH_3). **IR (neat)** v/cm^{-1}: 2850 (w), 1742 (m), 1720 (m), 1601 (m), 1454 (m), 1420 (m), 1320 (m), 1190 (m), 1060 (m), 900 (w), 765 (m), 682 (m). **HR-MS (TOF ES+):** calcd for $C_{22}H_{23}N_2O_6$ 411.1554 found 411.1551.

(+/-)-Di-tert-butyl (2S,5S,6R)-2,6-diphenyl-1,3-diazabicyclo[3.1.0]hex-3-ene-4,5-dicarboxylate (3k)

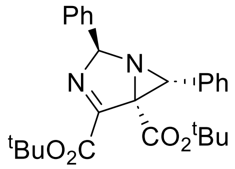

Chemical Formula: $C_{26}H_{30}N_2O_4$
Molecular Weight: 434.54

Yield: 10% (24 mg, 0.06 mmol)

Appearance: Colourless oil

^{1}H-NMR (500 MHz, CDCl$_3$) δ/ppm 7.54 (m, 2H), 7.39 (m, 2H), 7.33 -7.23 (m, 6H), 6.86 (s, 1H), 2.93 (s, 1H), 1.64 (s, 9H), 1.17 (s, 9H). **^{13}C-NMR (125 MHz, CDCl$_3$)** δ/ppm 165.9 (C), 164.3 (C), 161.1 (C), 136.8 (C), 134.1 (C), 128.7 (2CH), 128.2 (CH), 128.2 (2CH), 128.0 (2CH), 127.8 (CH), 127.4 (2CH), 95.8 (CH), 84.2 (C), 83.0 (C), 66.3 (C), 49.3 (CH), 28.2 (3CH$_3$), 27.7 (3CH$_3$). **IR (neat)** ν/cm^{-1}: 2980 (w), 2901 (w), 1744 (m), 1715 (m), 1495 (w), 1368 (w), 1313 (m), 1152 (s), 1081 (w), 1024 (w), 839 (w), 731 (s). **HR-MS (TOF ES+):** calcd for $C_{28}H_{31}N_2O_4$ 435.2278 found 435.2279.

(+/-)-Di-tert-butyl (2R,5S,6R)-2,6-diphenyl-1,3-diazabicyclo[3.1.0]hex-3-ene-4,5-dicarboxylate (3k')

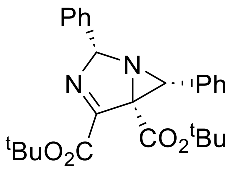

Chemical Formula: $C_{26}H_{30}N_2O_4$
Molecular Weight: 434.54

Yield: 26% (60 mg, 0.14 mmol)

Appearance: Colourless oil

^{1}H-NMR (500 MHz, CDCl$_3$) δ/ppm 7.59 (m, 2H), 7.46-7.40 (m, 4H), 7.38-7.27 (m, 4H), 6.11 (s, 1H), 3.05 (s, 1H), 1.60 (s, 1H), 1.23 (s, 1H). **^{13}C-NMR (125 MHz, CDCl$_3$)** δ/ppm 164.0 (C), 160.5 (C), 139.1 (C), 134.5 (C), 128.7 (2CH), 128.7 (CH), 128.1 (2CH), 128.0 (CH), 128.0 (2CH), 127.2 (2CH), 98.1 (CH), 84.3 (C), 82.7 (C), 66.1 (C), 54.0 (CH), 28.1 (3CH$_3$), 27.7 (2CH$_3$) **IR (neat)** ν/cm^{-1}: 2979 (w), 1743 (w), 1717 (m), 1495 (w), 1455 (w), 1347 (w), 1251 (m), 1153 (s), 1094 (w), 840 (w), 728 (w). **HR-MS (TOF ES+):** calcd for $C_{28}H_{31}N_2O_4$ 435.2278 found 435.2279.

(+/-)-Di-tert-butyl (2S,5S,6R)-2,6-bis(4-fluorophenyl)-1,3-diazabicyclo[3.1.0]hex-3-ene-4,5-dicarboxylate (3l)

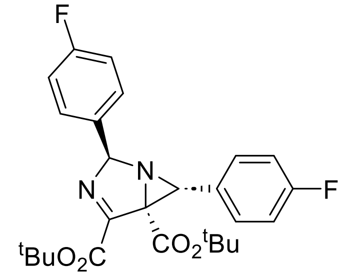

Chemical Formula: $C_{26}H_{28}F_2N_2O_4$
Molecular Weight: 470.52

Yield: 22% (26 mg, 0.06 mmol)

Appearance: Colourless oil

^{1}H-NMR (500 MHz, CDCl$_3$) δ/ppm 7.74 (m, 2H), 7.60 (m, 2H), 7.28 (m, 2H), 7.05 (s, 1H), 3.08 (s, 1H), 1.89 (s, 3CH$_3$), 1.46 (s, 3CH$_3$). **^{13}C-NMR (125 MHz, CDCl$_3$)** δ/ppm 165.9 (C), 164.0 (C), 162.6 (d, *J*= 250 Hz, CF), 162.5 (d, *J* = 250 Hz, CF), 160.9 (C), 132.5 (d, *J* = 3 Hz, C), 129.7 (d, *J* = 8 Hz, 2CH), 129.7 (d, *J* = 3 Hz, C), 129.1 (d, *J* = 8 Hz, 2CH), 115.8 (d, *J* = 22 Hz, 2CH), 115.0 (d, *J* = 22 Hz, 2CH), 95.3 (CH), 84.6 (C), 83.2 (C), 66.5 (C), 48.4 (CH), 28.1 (3CH$_3$), 27.7 (3CH$_3$). **^{19}F-NMR (376 MHz, CDCl$_3$) δ/ppm** -113.7 (m), 114.1 (m). **IR (neat)** v/cm^{-1}: 2978 (w), 1744 (w), 1715 (w), 1605 (m), 1393 (w), 1276 (w), 1148 (s), 1079 (m), 1015 (w), 893 (w), 821 (m). **HR-MS (TOF ES+):** calcd for $C_{26}H_{29}F_2N_2O_4$ 471.2090 found 471.2091.

(+/-)-Di-tert-butyl (2R,5S,6R)-2,6-bis(4-fluorophenyl)-1,3-diazabicyclo[3.1.0]hex-3-ene-4,5-dicarboxylate (3l')

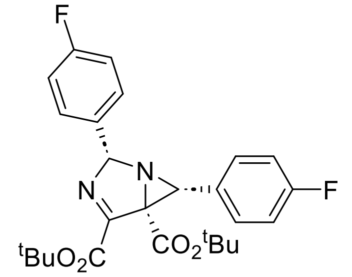

Chemical Formula: $C_{26}H_{28}F_2N_2O_4$
Molecular Weight: 470.52

Yield: 59% (71 mg, 0.15 mmol)

Appearance: Colourless oil

^{1}H-NMR (500 MHz, CDCl$_3$) δ/ppm 7.55 (m, 2H), 7.42 (m, 2H), 7.08 (m, 2H), 7.03 (m, 2H), 6.08 (s, 1H), 3.01 (s, 1H), 1.60 (s, 3CH$_3$), 1.25 (3CH$_3$). **^{13}C-NMR (125 MHz, CDCl$_3$)** δ/ppm 164.0 (C), 163.8 (C), 163.0 (d, *J* = 250 Hz, CF), 162.7 (d, *J* = 250 Hz, CF), 160.3 (C), 134.9 (d, *J* = 3 Hz, C), 130.1 (d, *J* = 3Hz, C), 129.5 (d, *J* = 8 Hz, 2CH), 129.0 (d, *J* = 8 Hz, 2CH), 115.6 (d, *J* = 22 Hz, 2CH), 115.0 (d, *J* = 22 Hz, 2CH), 97.3 (CH), 84.5 (C), 83.1 (C), 66.0 (C), 53.3 (CH), 28.1 (3CH$_3$), 27.8 (3CH$_3$). **IR (neat)** v/cm^{-1}: 2966 (m), 2901 (m), 1745 (s), 1721 (s), 1406 (m), 1393 (m), 1312 (w), 1259 (s), 1200 (w), 1065 (s), 1055 (s), 797 (w). **HR-MS (TOF ES+):** calcd for $C_{26}H_{29}F_2N_2O_4$ 471.2090 found 471.2091.

(+/-)-Di-tert-butyl (2S,5S,6R)-2,6-bis(4-methoxyphenyl)-1,3-diazabicyclo[3.1.0]hex-3-ene-4,5-dicarboxylate (3m)

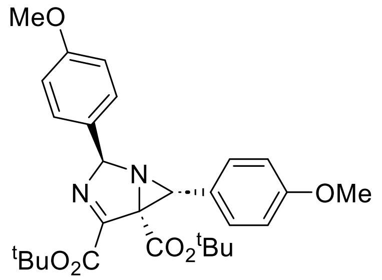

Chemical Formula: $C_{28}H_{34}N_2O_6$
Molecular Weight: 494.59

Yield: 6% (11 mg, 0.02 mmol)

Appearance: Colourless oil

^{1}H-NMR (500 MHz, CDCl$_3$) δ/ppm 7.43 (m, 2H),7.30 (m, 2H), 6.84-881 (m, 4H), 6.78 (s, 1H), 3.78 (s, 3H), 3.76 (s, 3H), 2.88 (s, 1H), 1.64 (s, 3CH$_3$), 1.21 (s, 3CH$_3$). **^{13}C-NMR (125 MHz, CDCl$_3$)** δ/ppm 165.6 (C), 164.2 (C), 161.1(C), 159.2 (C), 130.2 (C), 129.2 (2CH), 128.9 (C), 128.5 (2CH), 126.1 (C), 113.9 (2CH), 113.3 (2CH), 95.4 (CH), 84.0 (C), 82.8 (C), 66.4 (C), 55.3 (CH$_3$), 55.2 (CH$_3$), 48.9 (CH), 28.1 (3CH$_3$), 27.7 (3CH$_3$). **IR (neat)** ν/cm^{-1}: 2978 (w), 1743 (m), 1714 (m), 1612 (w), 1513 (m), 1457 (w), 1248 (s), 1153 (s), 1080 (m), 837 (w). **HR-MS (TOF ES+):** calcd for $C_{28}H_{35}N_2O_6$ 495.2490 found 495.2489.

(+/-)-Di-tert-butyl (2R,5S,6R)-2,6-bis(4-methoxyphenyl)-1,3-diazabicyclo[3.1.0]hex-3-ene-4,5-dicarboxylate (3m')

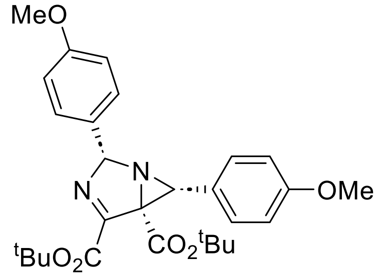

Chemical Formula: $C_{28}H_{34}N_2O_6$
Molecular Weight: 494.59

Yield: 16% (32 mg, 0.06 mmol)

Appearance: Colourless oil

^{1}H-NMR (500 MHz, CDCl$_3$) δ/ppm 7.48 (m, 2H), 7.36 (m, 2H), 6.94 (m, 2H), 6.86 (m, 2H), 6.05 (s, 1H), 3.82 (s, CH$_3$), 3.79 (s, CH$_3$), 2.97 (s, 1H), 1.59 (s, 9H), 1.26 (s, 9H). **^{13}C-NMR (125 MHz, CDCl$_3$)** δ/ppm 164.1 (C), 163.7 (C), 160.6 (C), 159.9 (C), 159.5 (C), 131.7 (C), 129.1 (2CH), 128.5 (2CH), 126.7 (C), 114.1 (2CH), 113.5 (2CH), 97.7 (CH), 84.2 (C), 82.7 (C), 66.0 (C), 55.4 (CH$_3$)55.4 (CH$_3$), 53.9 (CH), 28.1 (3CH$_3$), 27.8 (3CH$_3$). **IR (neat)** ν/cm^{-1}: 2978 (w), 1741 (m), 1716 (m), 1612 (w), 1513 (m), 1368 (w), 1247 (s), 1152 (s), 1035 (m), 829 (w). **HR-MS (TOF ES+):** calcd for $C_{28}H_{35}N_2O_6$ 495.2490 found 495.2489.

(+/-)-Di-tert-pentyl (2S,5S,6R)-2,6-bis(4-fluorophenyl)-1,3-diazabicyclo[3.1.0]hex-3-ene-4,5-dicarboxylate (3n)

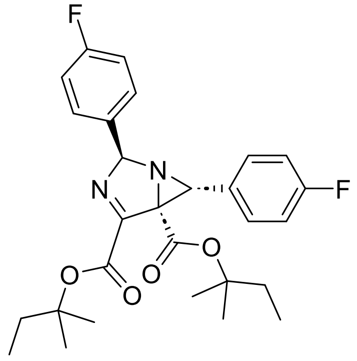

Chemical Formula: $C_{28}H_{32}F_2N_2O_4$
Molecular Weight: 498.57

Yield: 15% (47 mg, 0.09 mmol)

Appearance: Colourless oil

^{1}H-NMR (500 MHz, CDCl$_3$) δ/ppm 7.49 (m, 2H), 7.36 (m, 2H), 7.03-6.96 (m, 4H), 6.79 (s, 1H), 2.81 (s, 1H), 1.94 (m, 2H), 1.61 (s, 6H), 1.55 (m, 2H), 1.20 (s, 3H), 1.17 (s, 3H), 0.99 (m, 3H), 0.65 (t, *J* = 7.5 Hz, 3H). **^{13}C-NMR (125 MHz, CDCl$_3$)** δ/ppm 166.0 (C), 164.1 (C), 162.6 (d, *J* = 250 Hz, CF), 162.4 (d, *J* = 250 Hz, CF), 160.8 (C), 132.6 (d, *J* = 3 Hz, C), 129.8 (d, *J* = 8 Hz, 2CH), 129.7 (d, *J* = 3 Hz, C), 129.2 (d, *J* = 8 Hz, 2CH), 115.7 (d, *J* = 22 Hz, 2CH), 115.0 (d, *J* = 22 Hz, 2CH), 95.3 (CH), 87.0 (C), 86.1 (C), 66.6 (C), 48.4 (CH), 33.9 (CH$_2$), 33.0 (CH$_2$), 25.5 (CH$_3$), 25.3 (CH$_3$), 25.4 (CH$_3$), 25.2 (CH$_3$), 8.4 (CH$_3$), 8.2 (CH$_3$). **HR-MS (TOF ES+):** calcd for $C_{28}H_{33}F_2N_2O_4$ 499.2403 found 499.2405.

(+/-)-Di-tert-pentyl (2R,5S,6R)-2,6-bis(4-fluorophenyl)-1,3-diazabicyclo[3.1.0]hex-3-ene-4,5-dicarboxylate (3n')

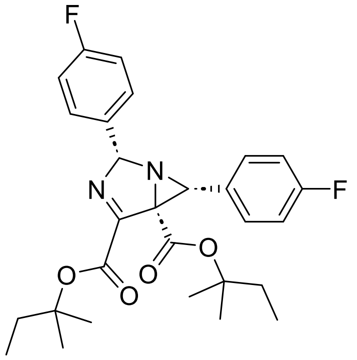

Chemical Formula: $C_{28}H_{32}F_2N_2O_4$
Molecular Weight: 498.57

Yield: 4% (12 mg, 0.02 mmol)

Appearance: Colourless oil

^{1}H-NMR (500 MHz, CDCl$_3$) δ/ppm 7.55 (m, 2H), 7.40 (m, 2H), 7.09 (m, 2H), 7.01 (m, 2H), 6.08 (s, 1H), 3.02 (s, 1H), 1.89 (m, 2H), 1.58 (s, 3H), 1.57 (s, 3H), 1.54 (m, 2H), 1.22 (s, 3H), 1.20 (s, 3H), 0.96 (t, J = 7.5 Hz, 3H), 0.67 (t, J = 7.5 Hz, 3H). **13C-NMR (125 MHz, CDCl3) δ/ppm** 164.2 (C), 164.0 (C), 163.8 (d, J = 250 Hz, CF), 162.5 (d, J = 250 Hz, CF), 160.3 (C), 135.0 (d, J = 3 Hz, C), 130.1 (d, J = 3 Hz, C), 129.6 (d, J = 8 Hz, 2CH), 129.0 (d, J = 8 Hz, 2CH), 115.5 (d, J = 22 Hz, 2CH), 115.0 (d, J = 22 Hz, 2CH), 97.3 (CH), 87.1 (C), 85.8 (C), 65.9 (C), 53.4 (CH), 33.7 (CH$_2$), 33.3 (CH$_2$), 25.5 (CH$_3$), 25.4 (CH$_3$), 25.2 (CH$_3$), 25.2 (CH$_3$), 8.5 (CH$_3$), 8.1 (CH$_3$). **HR-MS (TOF ES+):** calcd for $C_{28}H_{33}F_2N_2O_4$ 499.2403 found 499.2405.

(+/-)-Methyl (1R,4R,6R)-4-(4-fluorophenyl)-1,6-diphenyl-3-azabicyclo[3.1.0]hex-2-ene-2-carboxylate (5a) – proposed relative stereochemistry

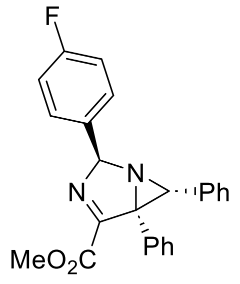

Chemical Formula: $C_{24}H_{19}FN_2O_2$
Molecular Weight: 386.43

Yield: 16% (22 mg, 0.06 mmol)

Appearance: Colourless oil

^{1}H-NMR (400 MHz, CDCl$_3$) δ/ppm 8.01 (m, 2H), 7.56-7.46 (m, 5H), 7.38-7.29 (m, 5H), 6.99 (m, 3H), 6.87 (s, 1H), 3.52 (s, 3H), 2.93 (s, 1H). **^{13}C-NMR (100 MHz, CDCl$_3$)** δ/ppm 169.4 (C), 166.3 (C), 162.6 (d, *J* = 250 Hz, CF), 137.8 (C), 131.6 (CH), 131.6 (C), 130.2 (d, *J* = 3 Hz, C), 129.4 (d, *J* = 8 Hz, 2CH), 129.3 (2CH), 128.6 (d, *J* = 8 Hz, 2CH), 128.1 (CH), 127.5 (2CH), 115.4 (2CH), 115.2 (2CH), 94.9 (CH), 65.6 (C), 52.6 (CH), 47.7 (CH_3) **^{19}F-NMR (376 MHz, CDCl$_3$) δ/ppm** -114.1 (m). **IR (neat)** v/cm^{-1}: 3063 (w), 1728 (m), 1605 (w), 1512 (m), 1348 (w), 1231 (w), 1013 (w), 881 (w), 821 (w), 688 (s), 522 (w). **HR-MS (TOF ES+):** calcd for $C_{24}H_{20}FN_2O_2$ 387.1503 found 387.1503.

(+/-)-Methyl (1R,4S,6R)-4-(4-fluorophenyl)-1,6-diphenyl-3-azabicyclo[3.1.0]hex-2-ene-2-carboxylate (5a') – proposed relative stereochemistry

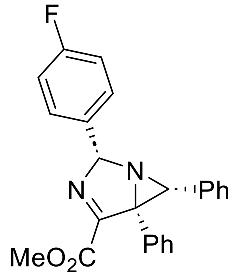

Chemical Formula: $C_{24}H_{19}FN_2O_2$
Molecular Weight: 386.43

Yield: 20% (8 mg, 0.07 mmol)

Appearance: Colourless oil

^{1}H-NMR (400 MHz, CDCl$_3$) δ/ppm 7.97 (m, 2H), 7.63 (m, 2H), 7.51 (m, 1H), 7.48-7.43 (m, 6H), 7.39 (m, 1H), 7.08 (m, 2H), 6.17 (s, 1H), 3.51 (s, 3H), 3.12 (s, 1H). **^{13}C-NMR (100 MHz, CDCl$_3$)** δ/ppm 168.5 (C), 166.5 (C), 162.7 (d, *J* = 250 Hz, CF), 140.2 (C), 131.7 (CH), 131.4 (C), 130.5 (d, *J* = 3 Hz, C), 129.3 (d, *J* = 8 Hz, 2CH), 129.2 (2CH), 128.7 (d, *J = 8* Hz, 2CH), 128.6 (CH), 127.2 (2CH), 115.4 (2CH), 115.2 (2CH), 97.3 (CH), 64.9 (C), 53.2 (CH), 52.6 (CH_3). **IR (neat)** v/cm^{-1}: 3031 (w), 1734 (m), 1604 (w), 1509 (m), 1447 (w), 1326 (w), 1196 (m), 918 (w), 784 (m), 693 (s).**^{19}F-NMR (376 MHz, CDCl$_3$) δ/ppm** -114.1 (m). **HR-MS (TOF ES+):** calcd for $C_{24}H_{20}FN_2O_2$ 387.1503 found 387.1503.

(+/-)-Methyl (2S,5S,6R)-5,6-diphenyl-2-(p-tolyl)-1,3-diazabicyclo[3.1.0]hex-3-ene-4-carboxylate (5b) – proposed relative stereochemistry

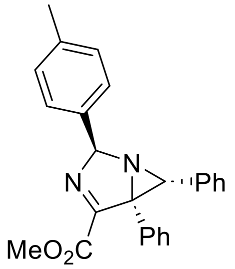

Chemical Formula: $C_{25}H_{22}N_2O_2$
Molecular Weight: 382.46

Yield: 4% (6 mg, 0.016 mmol)

Appearance: Colourless oil

^{1}H-NMR (500 MHz, CDCl$_3$) δ/ppm 8.03 (m, 2H), 7.53 (m, 3H), 7.48 (m, 2H), 7.31 (m, 2H), 7.27 (m, 3H), 7.12 (m, 2H), 6.87 (s, 1H), 3.52 (s, 3H), 2.94 (s, 1H), 2.33 (s, 3H). **^{13}C-NMR (125 MHz, CDCl$_3$)** δ/ppm 169.6 (C), 166.3 (C), 137.9 (C), 137.8 (C), 131.7 (C), 131.5 (CH), 131.4 (C), 129.4 (2CH), 129.0 (2CH), 128.6 (2CH), 128.6 (2CH), 128.0 (CH), 127.6 (2CH), 127.6 (2CH), 94.9 (CH), 65.8 (C), 52.5 (CH_3), 48.5 (CH), 21.4 (CH_3). **IR (neat)** v/cm^{-1}: 3061 (w), 1738 (s), 1605 (w), 1515 (w), 1494 (w), 1331 (m), 1223 (w), 1019 (w), 9090 (w), 695 (s). **HR-MS (TOF ES+):** calcd for $C_{25}H_{23}N_2O_2$ 383.1754 found 383.1754.

(+/-)-Methyl (2R,5S,6R)-5,6-diphenyl-2-(p-tolyl)-1,3-diazabicyclo[3.1.0]hex-3-ene-4-carboxylate (5b') – proposed relative stereochemistry

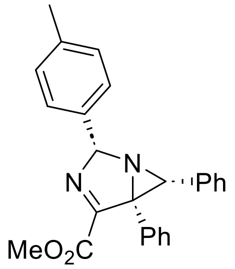

Chemical Formula: $C_{25}H_{22}N_2O_2$
Molecular Weight: 382.46

Yield: 13% (22 mg, 0.06 mmol)

Appearance: Colourless oil

^{1}H-NMR (500 MHz, CDCl$_3$) δ/ppm 7.99 (m, 2H), 7.64 (m, 2H), 7.51 (m, 1H), 7.44 (m, 4H), 7.38 (m, 3H), 7.17 (m, 2H), 6.16 (s, 1H), 3.51 (s, 3H), 3.12 (s, 1H), 2.36 (s, 3H). **^{13}C-NMR (125 MHz, CDCl$_3$)** δ/ppm 168.67 (C), 166.6 (C), 140.5 (C), 137.9 (C), 131.8 (C), 131.6 (CH), 131.5 (C), 129.3 (2CH), 129.0 (2CH), 128.8 (2CH), 128.7 (2CH), 128.5 (CH), 127.5 (2CH), 127.2 (2CH), 97.2 (CH), 65.1 (C), 54.1 (CH), 52.5 (CH_3), 21.4 (CH_3). **IR (neat)** v/cm^{-1}: 3061 (w), 1741 (s), 1611 (m), 1515 (w), 1494 (w), 1344 (m), 1222 (m), 1174 (m), 1060 (m), 926 (m), 774 (w). **HR-MS (TOF ES+):** calcd for $C_{25}H_{23}N_2O_2$ 383.1754 found 383.1754

(+/-) (2R,5S,6R)-2,4,5,6-tetraphenyl-1,3-diazabicyclo[3.1.0]hex-3-ene (6) – proposed relative stereochemistry

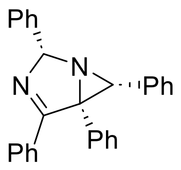

Chemical Formula: $C_{28}H_{22}N_2$
Molecular Weight: 386.50

Yield: 13% (8 mg, 0.21 mmol)

Appearance: Colourless oil

^{1}H-NMR (500 MHz, $CDCl_3$) δ/ppm 7.80 (m, 2H), 7.50 (m, 2H), 7.40-7.35 (m, 2H), 7.30 (m, 2H), 7.26-7.23 (m, 5H), 7.09 (m, 1H), 7.07 (m, 2H), 6.95 (m, 2H), 6.30 (s, 1H), 3.28 (s, 1H). **^{13}C-NMR (125 MHz, $CDCl_3$)** δ/ppm 173.1 (C), 142.0 (C), 136.2 (C), 133.3 (C), 132.2 (C), 130.9 (2CH), 130.8 (CH), 129.3 (2CH), 128.7 (2CH), 128.4 (2CH), 128.2 (2CH), 128.1 (2CH), 128.1 (4CH), 127.9 (CH), 127.6 (CH), 127.1 (CH), 95.7 (CH), 67.9 (C), 54.3 (CH). **IR (neat)** ν/cm^{-1}: 3061 (w), 3029 (w), 1601 (w), 1494 (w), 1320 (w), 1175 (w), 1026 (w)m 909 (w), 751 (m), 696 (s). **HR-MS (TOF ES+):** calcd for $C_{28}H_{23}N_2$ 387.1856 found 383.1858.

Characterisation of 1,6-dihydropyrimidine-4,5-dicarboxylate

Dimethyl 2,6-diphenyl-1,6-dihydropyrimidine-4,5-dicarboxylate (6a)

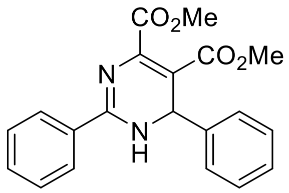

Chemical Formula: $C_{20}H_{18}N_2O_4$
Molecular Weight: 350.37

Yield: 87% (159 mg, 0.45 mmol)

Appearance: Yellow solid

Melting point: 195 °C

^{1}H-NMR (400 MHz, $CDCl_3$) δ/ppm 7.75 (m, 2H), 7.50 (m, 1H), 7.39 (m, 2H), 7.34-7.27 (m, 5H), 6.45 (s, 1H), 5.65 (s, 1H), 3.91 (s, 3H), 3.63 (s, 3H).**^{13}C-NMR (100 MHz, $CDCl_3$)** δ/ppm 171.3 (C), 166.3 (C), 165.5 (C), 158.3 (C), 149.3 (C), 149.3 (C), 133.2 (CH), 132.0 (CH), 129.1 (2CH), 128.7 (2CH), 128.6 (CH), 127.4 (CH), 127.0 (2CH), 105.3 (C), 53.7 (CH), 52.7 (CH_3), 51.9 (CH_3). **IR (neat)** ν/cm^{-1}: 3252 (w), 2950 (w), 1723 (m), 1704 (m), 1609 (w), 1536 (w), 1490 (s), 1433 (s), 1357 (m), 1311 (w), 1228 (s), 1102 (m), 834 (w), 775 (w), 685 (w). **HR-MS (TOF ES+):** calcd for $C_{20}H_{19}N_2O_4$ 351.1339 found 351.1346.

Dimethyl 2,6-bis(4-fluorophenyl)-1,6-dihydropyrimidine-4,5-dicarboxylate (6b)

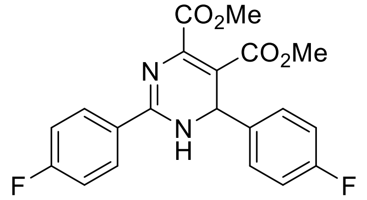

Chemical Formula: $C_{20}H_{16}F_2N_2O_4$
Molecular Weight: 386.35

Yield: 89% (290 mg, 0.77 mmol)

Appearance: Yellow solid

Melting point: 80 °C

^{1}H-NMR (500 MHz, DMSO) δ/ppm 9.66 (d, *J* = 3.8 Hz, 1H), 7.93 (m, 2H), 7.35-7.33 (m, 4H), 7.22 (m, 2H), 5.33 (d, *J* =3.8 Hz, 1H), 3.77 (s, 3H), 3.58 (s, 3H). **^{13}C-NMR (125 MHz, DMSO)** δ/ppm 167.4 (C) 165.4 (d, *J* = 250 Hz, CF), 164.7 (C), 162.8 (d, *J* = 250 Hz, CF), 156.7 (C), 150.0 (C), 140.3 (d, *J* = 2 Hz, C), 130.3 (d, *J* = 9 Hz, 2CH), 128.9 (d, *J* = 3 Hz, C), 128.5 (d, *J* = 9 Hz, 2CH), 115.8 (d, *J* = 2 Hz, 2CH), 115.6 (d, *J* = 2 Hz, 2CH), 102.9 (C), 52.1 (CH_3), 51.7 (CH), 51.0 (CH_3). **^{19}F-NMR (376 MHz, $CDCl_3$) δ/ppm** -107.9 (m), -114.0 (m) **IR (neat)** v/cm^{-1}: 3489 (w), 1729 (m), 1684 (w), 1545 (m), 1432 (m), 1227 (m), 1069 (s), 1007 (s), 982 (s), 849 (m), 803 (m), 677 (w), 568 (w). **HR-MS (TOF ES+):** calcd for $C_{20}H_{17}F_2N_2O_4$ 387.1151 found 387.1150 (M+H).

Dimethyl 2,6-bis(4-bromophenyl)-1,6-dihydropyrimidine-4,5-dicarboxylate (6c)

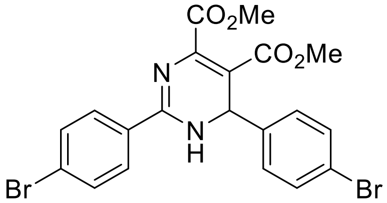

Chemical Formula: $C_{20}H_{16}Br_2N_2O_4$
Molecular Weight: 508.1660

Yield: 82 % (22 mg, 0.04 mmol)

Appearance: Yellow oil

^{1}H-NMR (400 MHz, $CDCl_3$) δ/ppm 7.59 (d, *J* = 8.6 Hz, 2H), 7.51 (d, *J* = 8.5 Hz, 2H), 7.46 (d, *J* = 8.5 Hz, 2H), 7.25 (d, *J* = 8.5 Hz, 2H), 6.64 (s, 1H), 5.57 (s, 1H), 3.90 (s, 3H), 3.63 (s, 3H). **^{13}C-NMR (100 MHz, $CDCl_3$)** δ/ppm 168.4 (C), 165.4 (C), 157.5 (C), 149.3 (C), 143.0 (C), 132.7 (2CH), 132.5 (2CH), 132.1 (C), 129.3 (2CH), 129.0 (2CH), 127.6 (C), 123.3 (C), 105.9 (C), 53.6 (CH_3), 53.2 (CH), 52.4 (CH_3). **IR (neat)** v/cm^{-1}: 2949 (m), 1743 (s), 1735 (s), 1617 (w), 1590 (s), 1534 (m)m 1308 (s), 1231 (s), 1158 (m), 1096 (m), 1069 (m), 965 (m), 750 (m) 632 (s). **HR-MS (TOF ES+):** calcd for $C_{20}H_{17}Br_2N_2O_4$ 508.9533 found 508.9530 (M+H$^+$).

Dimethyl 2,6-di-*p*-tolyl-1,6-dihydropyrimidine-4,5-dicarboxylate (6d)

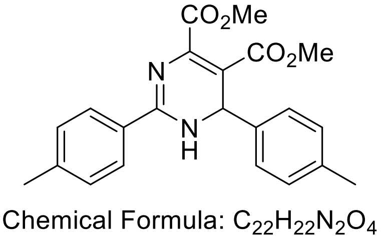

Chemical Formula: $C_{22}H_{22}N_2O_4$
Molecular Weight: 378.42

Yield: 74% (33 mg, 0.09 mmol)

Appearance: Yellow solid

Melting point: 182 °C

^{1}H-NMR (400 MHz, $CDCl_3$) δ/ppm 7.62 (m, 2H), 7.59 (m, 2H), 7.16-7.10 (m, 4H), 6.89 (s, 1H), 5.54 (s, 1H), 3.88 (s, 3H), 3.59 (s, 3H), 2.35 (s, 3H), 2.31 (s, 3H). **^{13}C-NMR (100 MHz, $CDCl_3$)** δ/ppm 171.3 (C), 168.4 (C), 165.5

(C), 158.1 (C), 149.3 (C), 142.6 (C), 141.2 (C), 138.4 (C), 130.4 (C), 129.7 (2CH), 129.4 (2CH), 127.4 (2CH), 127.0 (2CH), 53.5 (CH), 52.6 (CH_3), 51.8 (CH_3), 21.6 (CH_3), 21.2 (CH_3). **IR (neat)** v/cm^{-1}: 3329 (s), 1724 (m), 1701 (m), 1544 (m), 1430 (m), 1367 (m), 1311 (m), 1233 (s), 1101 (s), 1068 (m), 954 (w), 759 (w), 663 (w). **HR-MS (TOF ES+):** calcd for $C_{22}H_{23}N_2O_4$ 379.1653 found 379.1652 ($M+H^+$).

Dimethyl 2,6-bis(3-methoxyphenyl)-1,6-dihydropyrimidine-4,5-dicarboxylate (6e)

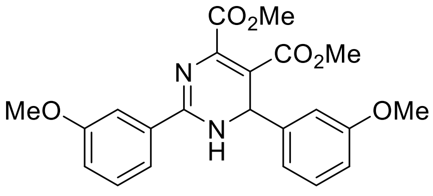

Chemical Formula: $C_{22}H_{22}N_2O_6$
Molecular Weight: 410.42

Yield: 86% (62 mg, 0.15 mmol)

Appearance: Yellow oil

^{1}H-NMR (500 MHz, CDCl$_3$) δ/ppm 7.35 (m, 1H), 7.30-7.20 (m, 3H), 7.01 (m, 2H), 6.94 (m, 1H), 6.86 (m, 1H), 6.42 (d, *J* = 3.0 Hz, 1H), 5.61 (d, *J* = 3.0 Hz, 1H), 3.91 (CH_3), 3.82 (CH_3), 3.77 (CH_3), 3.64 (CH_3). **^{13}C-NMR (125 MHz, CDCl$_3$)** δ/ppm 168.2 (C), 165.4 (C), 160.3 (C), 160.0 (C), 158.1 (C), 149.1 (C), 145.3 (C), 134.8 (C), 130.3 (CH), 129.9 (CH), 119.3 (CH), 119.2 (CH), 118.5 (C), 114.2 (CH), 112.7 (CH), 112.5 (CH), 105.8 (C), 55.7 (CH_3), 55.4 (CH_3), 53.9 (CH), 52.7 (CH_3), 52.0 (CH_3). **IR (neat)** v/cm^{-1}: 3331 (w), 2950 (w), 1737 (m), 1700 (m), 1599 (m), 1499 (m), 1433 (m), 1278 (m), 1278 (s), 1099 (s), 1067 (s), 908 (m), 727 (s), 646 (m), 562 (w). **HR-MS (TOF ES+):** calcd for $C_{22}H_{23}N_2O_6$ 379.1652 found 379.1652 ($M+H^+$).

Dimethyl 2,6-bis(2,4-difluorophenyl)-1,6-dihydropyrimidine-4,5-dicarboxylate (6f)

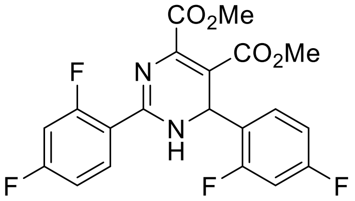

Chemical Formula: $C_{20}H_{14}F_4N_2O_4$
Molecular Weight: 422.33

Yield: 87% (52 mg, 0.12 mmol)

Appearance: Yellow solid

Melting point: 116 °C

^{1}H-NMR (400 MHz, CDCl$_3$) δ/ppm 8.26 (m, 1H), 7.38 (m, 1H), 7.06 (d, J = 10 Hz, 1H), 6.97-6.78 (m, 4H), 5.97 (d, *J* = 3.0 Hz, 1H), 3.94 (s, 3H), 3.66 (s, 3H). **^{13}C-NMR (100 MHz, CDCl$_3$)** δ/ppm 167.8 (C), 164.8 (C), 166.6 (dd, *J* = 250, 10 Hz, CF), 164.4 (d, *J* = 250, 10 Hz, CF), 162.8 (dd, *J* = 250 Hz, 10 Hz, CF) 161.3 (dd, J = 250, 10 Hz, CF), 154.4 (d, *J* = 2Hz, C), 150.4 (C), 134.0 (dd , *J* = 10, 3 Hz, CH), 130.2 (dd , *J* = 10, 5 Hz, CH), 125.3 (dd, *J*= 13, 3 Hz, C), 116.3 (dd , *J* = 3 Hz, C) 112.9 (dd , *J* = 21, 3 Hz, CH), 112.7 (d, *J* = 21, 3 Hz, CH), 112.3 (d, *J* = 3 Hz, CH), 104.7 (m, 2CH), 102.5 (C), 52.9 (CH_3), 52.2 (CH_3), 46.5 (CH). **^{19}F-NMR (376 MHz, CDCl$_3$) δ/ppm** -102.4 (m), 108.8 (m), 110.9 (m), 116.1 (m). **IR (neat)** v/cm^{-1}: 3316 (w), 2954 (w), 1736 (m)m 1705 (m), 1608 (m), 1496 (s), 1433 (m), 1363 (m), 1300 (m), 1215 (s), 1141 (m), 1092 (s), 1064 (s), 966 (m), 789 (m), 603 (w). **HR-MS (TOF ES+):** calcd 423.0963 found 423.0962.

Dimethyl 2,6-bis(2-(trifluoromethyl)phenyl)-1,6-dihydropyrimidine-4,5-dicarboxylate (6g)

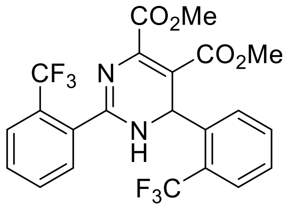

Chemical Formula: $C_{22}H_{16}F_6N_2O_4$
Molecular Weight: 486.37

Yield: 80% (57 mg, 0.12 mmol)

Appearance: Yellow solid

Melting point: 121 °C

^{1}H-NMR (400 MHz, CDCl$_3$) δ/ppm 7.80 (m, 1H)m 7.69 (m, 2H), 7.63-7.46 (m, 5H), 6.13 (s, 1H), 6.10 (d, *J* = 2.7 Hz, CH), 3.94 (s, 3H), 3.59 (s, 3H). **^{13}C-NMR (100 MHz, CDCl$_3$)** δ/ppm 167.5 (C), 164.7 (C), 157.9 (C), 150.3 (C), 139.4 (C), 133.5 (CH), 132.4 (CH), 131.3 (2CH), 131.0 (2CH), 130.9 (CH), 128.9 (CH), 128.1 (q, *J* = 30 Hz, C), 127.3 (q, *J* = 30 Hz, C), 126.5 (q, *J* = 5 Hz, C), 126.2 (q, *J* = 5 Hz, C), 125.9 (C), 104.3 (C), 52.9 (CH_3), 52.1 (CH_3), 49.5 (CH). **IR (neat)** v/cm^{-1} 3091 (w), 2955 (w), 1742 (m), 1709 (m), 1619 (w), 1507 (m), 1436 (m), 1309 (s), 1246 (s), 1095 (s), 1054 (s), 767 (m), 655 (w). **HR-MS (TOF ES+):** calcd for $C_{22}H_{17}F_6N_2O_4$ 487.1087 found 487.1088.

Bi-tert-butyl 2,6-diphenyl-1,6-dihydropyrimidine-4,5-dicarboxylate (6h)

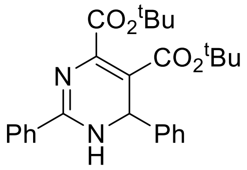

Chemical Formula: $C_{26}H_{30}N_2O_4$
Molecular Weight: 434.54

Yield: 80% (173 mg, 0.06 mmol)

Appearance: Yellow solid

Melting point: 179 °C

^{1}H-NMR (400 MHz, CDCl$_3$) 7.73 (m, 2H), 7.32 (m, 1H), 7.40-7.28 (m, 7H), 6.27 (s, 1H), 5.59 (s, 1H), 1.59 (s, 9H), 1.32 (s, 9H). **^{13}C-NMR (125 MHz, CDCl$_3$)** δ/ppm 168.8 (C), 164.1 (C), 156.8 (C), 148.3 (C), 144.2 (C), 133.7 (C), 131.5 (CH), 128.7 (2CH), 128.6 (2CH), 128.3 (CH), 127.3 (2CH), 127.3 (2CH), 107.3 (C), 82.1 (C), 80.9 (C), 54.5 (CH), 28.0 ($3CH_3$), 28.0 ($3CH_3$). **IR (neat)** v/cm^{-1}: 2977 (w), 1697 (s), 1695 (s), 1606 (w), 1502 (s), 1363 (m), 1212 (w), 1230 (s), 1157 (s), 1058 (s), 827 (w), 691 (m). **HR-MS (TOF ES+):** calcd for $C_{26}H_{31}N_2O_4$ 435.2278 found 435.2280.

Di-tert-butyl 2,6-bis(4-fluorophenyl)-1,6-dihydropyrimidine-4,5-dicarboxylate (6i)

Tautomerising in solution to 1,4-isomer.

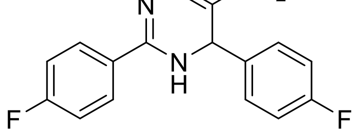

Chemical Formula: $C_{26}H_{28}F_2N_2O_4$
Molecular Weight: 470.52

Yield: 88% (127 mg, 0.27 mmol)

Appearance: Yellow solid

Melting point: 202°C

^{1}H-NMR (400 MHz, CDCl$_3$) δ/ppm 7.73 (m, 2H), 7.37 (m, 2H), 7.05-7.00 (m, 4H), 6.41 (d, *J* = 3 Hz, 1H), 5.53 (d, *J* = 3 Hz, 1H), 1.60 (s, 9H), 1.31 (s, 9H). **^{13}C-NMR (100 MHz, CDCl$_3$**

166.8 (C), 163.7 (C), 162.6 (d, J = 250 Hz, CF), 162.4 (d, J = 250 Hz, CF), 156.0 (C), 148.5 (C), 140.3 (d, J = 3 Hz, C), 129.7 (d, J = 3 Hz, C), 129.6 (d, J = 9 Hz, 2CH), 128.8 (d, J = 9 Hz, 2CH), 115.7 (d, J = 4 Hz, 2CH), 115.8 (d, J = 4 Hz, 2CH), 107.0 (C), 82.4 (C), 81.1 (C), 53.8 (CH), 28.2 ($3CH_3$), 28.1 ($3CH_3$). **IR (neat)** ν/cm^{-1}: 3274 (m), 2984 (w), 1711 (w), 1694 (m), 1503 (m), 1455 (w), 1232 (s), 1156 (s), 1062 (m), 844 (s), 722 (w). **HR-MS (TOF ES+):** calcd for $C_{26}H_{28}F_2N_2O_4$ 471.2090 found 471.2096.

Di-tert-butyl 2,6-bis(4-methoxyphenyl)-1,6-dihydropyrimidine-4,5-dicarboxylate (6j)

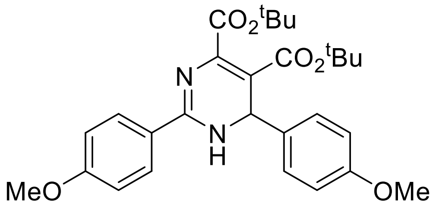

Chemical Formula: $C_{28}H_{34}N_2O_6$
Molecular Weight: 494.59

Yield: 88% (25 mg, 0.05 mmol)

Appearance: Yellow solid

Melting point: 195 °C

^{1}H-NMR (500 MHz, $CDCl_3$) δ/ppm 7.69 (m, 2H), 7.31 (m, 2H), 6.87-6.82 (m, 4H), 5.48 (s, 1H), 3.81 (s, 3H), 3.77 (s, 3H), 1.59 (s, 9H), 1.33 (s, 9H). **^{13}C-NMR (125 MHz, $CDCl_3$)** δ/ppm 164.5 (C), 162.4 (C), 159.6 (C), 137.0 (C), 131.4 (C), 128.9 (2CH), 128.8 (C), 128.6 (2CH), 126.1 (C), 114.1 (2CH), 114.0 (2CH), 80.7 (2C), 60.4 (CH), 55.4 (CH_3), 55.3 (CH_3), 28.1 ($3CH_3$), 28.0 ($3CH_3$). Two quaternary carbons not apparent. **IR (neat)** ν/cm^{-1}: 3336 (w), 2976 (w), 1731 (m), 1691 (m), 1581 (m), 1498 (s), 1351 (m), 1224 (s), 1157 (s), 1023 (s), 796 (m). **HR-MS (TOF ES+):** calcd for $C_{28}H_{35}N_2O_6$ 495.2490 found 495.2488.

Di-tert-pentyl 2,6-bis(4-fluorophenyl)-1,6-dihydropyrimidine-4,5-dicarboxylate (6k)

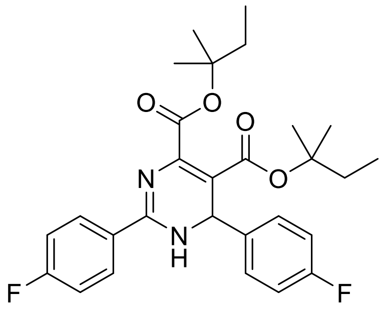

Chemical Formula: $C_{28}H_{32}F_2N_2O_4$
Molecular Weight: 498.57

Yield: 80% (28 mg, 0.06 mmol)

Appearance: Yellow solid

Melting point: 176 °C

^{1}H-NMR (400 MHz, $CDCl_3$) δ/ppm 7.71 (m, 2H), 7.38 (m, 2H), 7.10-7.07 (m, 4H), 6.45 (s, 1H), 5.56 (s, 1H), 1.89 (m, 2H), 1.65 (m, 2H), 1.59 (s, 3H), 1.56 (s, 3H), 1.29 (s, 6H), 0.97 (t, J = 7.5 Hz, 3H), 0.68 (t, J = 7.5 Hz, 3H). **^{13}C-NMR (100 MHz, $CDCl_3$)** δ/ppm 164.9 (d, J = 250 Hz, C), 163.9 (C), 162.5 (d, J = 250 Hz, C), 161.5 (C), 140.1 (C), 129.8 (d, J = 3 Hz, C), 129.4 (d, J = 3 Hz, C), 129.2 (C), 129.1 (d, J = 8 Hz, 4CH), 115.9 (d, J = 8 Hz, 2CH), 115.0 (d, J = 8 Hz, 2CH), 83.9 (2C), 53.8 (CH not apparent), 33.9 (CH_2), 33.3 (CH_2), 25.7 (CH_3), 25.5 (CH_3), 25.4 ($2CH_3$), 8.4 (CH_3), 8.2 (CH_3). **^{19}F-NMR (376 MHz, $CDCl_3$) δ/ppm** -107.7 (m), -113.5 (m). **IR (neat)** ν/cm^{-1}: 3006 (w), 1740 (m), 1717 (s), 1543 (s), 1366 (s), 1291 (m), 1230 (m), 1145 (m), 1080 (w), 789 (w), 723 (w), 648 (s). **HR-MS (TOF ES+):** calcd for $C_{28}H_{33}N_2O_4$ 499.2403 found at 499.2403.

Characterisation of pyrimidine-4,5-dicarboxylates

Dimethyl 2,6-diphenylpyrimidine-4,5-dicarboxylate (7a)

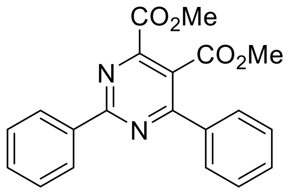

Chemical Formula: $C_{20}H_{16}N_2O_4$
Molecular Weight: 348.36

Yield: 70% (74 mg, 0.21 mmol)

Appearance: White solid

Melting point: 128 °C

^{1}H-NMR (500 MHz, $CDCl_3$) δ/ppm 8.60 (m, 2H), 7.82 (m, 2H), 7.54-7.49 (m, 6H), 4.05 (s, 3H), 3.82 (s, 3H). **^{13}C-NMR (125 MHz, $CDCl_3$)** δ/ppm 167.8 (C), 165.8 (C), 165.3 (C), 165.0 (C), 155.4 (C), 137.4 (C), 136.5 (C), 132.2 (CH), 131.1 (CH), 129.3 (2CH), 129.1 (4CH), 129.0 (2CH), 123.4 (C), 53.9 (CH_3), 53.4 (CH_3). **IR (neat)** ν/cm^{-1}: 2950 (w), 1722 (s), 1547 (m), 1530 (m), 1441 (m), 1397 (m), 1067 (m), 1025 (m), 1025 (m), 999 (w), 766 (w), 691 (s), 670 (s). **HR-MS (TOF ES+):** calcd for $C_{20}H_{17}N_2O_4$ 349.1183 found 349.1183.

Dimethyl 2,6-bis(4-fluorophenyl)pyrimidine-4,5-dicarboxylate (7b)

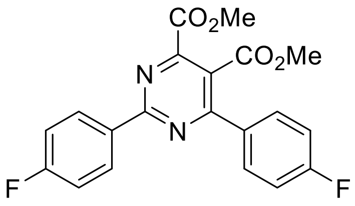

Chemical Formula: $C_{20}H_{14}F_2N_2O_4$
Molecular Weight: 384.34

Yield: 71% (50 mg, 0.13 mmol)

Appearance: Yellow solid

Melting point: 114 °C

^{1}H-NMR (400 MHz, $CDCl_3$) δ/ppm 8.60 (m, 2H), 7.62 (m, 2H), 7.22-7.16 (m, 4H), 4.05 (s, CH_3), 3.84 (s, CH_3). **^{13}C-NMR (100 MHz, $CDCl_3$)** δ/ppm 167.4 (C), 164.9 (C), 166.8 (d, *J* = 250 Hz, CF), 165.8 (d, *J* = 250 Hz, CF) 164.9 (C), 164.5 (C), 155.3 (C), 133.2 (d, *J* = 3Hz, C), 132.4 (d, *J* = 3 Hz, C) ,131.4 (d, *J* = 9 Hz, 2CH) ,131.1 (d, *J* = 9 Hz, 2CH), 122.9 (C), 116.1 (d, *J* = 13 Hz, 2CH), 115.8 (d, *J* = 13 Hz, 2CH), 53.7 (CH_3), 53.3 (CH_3). **^{19}F-NMR (376 MHz, $CDCl_3$) δ/ppm** -107.9 (m), -109.0 (m). **IR (neat)** ν/cm^{-1}: 1731 (s), 1600 (m), 1544 (s), 1437 (m), 1275 (s), 1193 (s), 1072 (m), 973 (w), 848 (m), 743 (m), 578 (m). **IR (neat)** ν/cm^{-1}: **HR-MS (TOF ES+):** calcd for $C_{20}H_{15}F_2N_2N_2O_4$ 385.0996 found 385.0994.

Dimethyl 2,6-bis(4-bromophenyl)pyrimidine-4,5-dicarboxylate (7c)

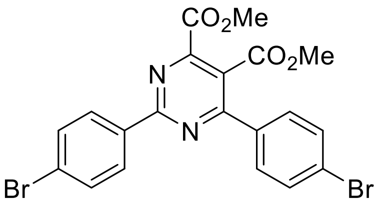

Chemical Formula: $C_{20}H_{14}Br_2N_2O_4$
Exact Mass: 503.93

Yield: 66% (45 mg, 0.09 mmol)

Appearance: White solid

Melting point: 145 °C

^{1}H-NMR (400 MHz, $CDCl_3$) δ/ppm 8.44 (m, 2H), 7.65 (m, 6H), 4.05 (s, CH_3), 3.84 (s, CH_3). **^{13}C-NMR (100 MHz, $CDCl_3$)**

167.5 (C), 165.1 (C), 164.9 (C), 164.3 (C), 155.7 (C), 136.1 (C), 135.3 (C), 132.5 (2CH), 132.4 (2CH), 130.9 (2CH), 130.7 (2CH), 127.4 (C), 126.2 (C), 123.5 (C), 54.1 (CH_3), 53.7 (CH_3). **IR (neat)** v/cm^{-1}: 2995 (w), 1732 (s), 1719 (s), 1542 (s), 1488 (w), 1361 (m), 1275 (m), 1186 (s), 1139 (s), 1005 (s), 974 (m), 846 (w), 751 (m), 692 (s), 670 (s). **HR-MS (TOF ES+):** calcd for $C_{20}H_{15}Br_2N_2O_4$ found 504.9391 found 504.9393.

Dimethyl 2,6-di-p-tolylpyrimidine-4,5-dicarboxylate (7d)

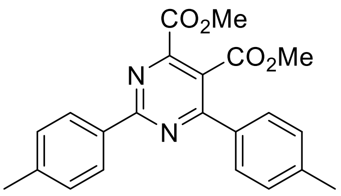

Chemical Formula: $C_{22}H_{20}N_2O_4$
Exact Mass: 376.14

Yield: 61% (76 mg, 0.20 mmol)

Appearance: Yellow oil

^{1}H-NMR (400 MHz, $CDCl_3$) δ/ppm 8.47 (m, 2H), 7.73 (m, 2H), 7.32 (m, 4H), 4.04 (s, 3H), 3.84 (s, 3H), 2.43 (s, 2CH_3). **^{13}C-NMR (100 MHz, $CDCl_3$)** 167.8 (C), 165.3 (C), 165.2 (C), 164.7 (C), 155.0 (C), 142.3 (C), 141.2 (C), 134.3 (C), 133.7 (C), 129.5 (4CH), 129.0 (2CH), 128.7 (2CH), 122.5 (C), 53.5 (CH_3), 53.1 (CH_3), 21.7 (CH_3), 21.6 (CH_3). **IR (neat)** v/cm^{-1}: 3033 (w), 2951 (w), 1727 (s), 1611 (w), 1508 (s), 1427 (m), 1395 (m), 1295 (s), 1196 (s), 1083 (s), 880 (w), 811 (w), 738 (s), 640 (w). **HR-MS (TOF ES+):** calcd for $C_{22}H_{21}N_2O_4$ 377.1504 found 377.1496.

Dimethyl 6-(2-methoxyphenyl)-2-(3-methoxyphenyl)pyrimidine-4,5-dicarboxylate (7e)

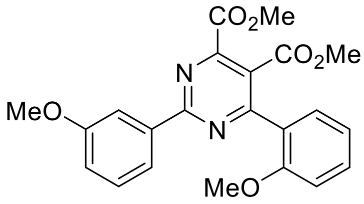

Chemical Formula: $C_{22}H_{20}N_2O_6$
Exact Mass: 408.13

Yield: 58% (175 mg, 0.42 mmol)

Appearance: Yellow solid

Melting point: 110 °C

^{1}H-NMR (400 MHz, $CDCl_3$) δ/ppm 8.19 (m, 1H), 8.11 (m, 1H), 7.44-7.39 (m, 2H), 7.38-7.33 (m, 2H), 4.05 (s, CH_3), 3.91 (s, CH_3), 3.88 (s, CH_3), 3.83 (s, CH_3). **^{13}C-NMR (100 MHz, $CDCl_3$)** 167.5 (C), 165.4 (C), 165.0 (C), 164.6 (C), 160.1 (C), 159.9 (C), 155.0 (C), 138.4 (C), 137.7 (C), 129.9 (2CH), 123.4 (C), 121.7 (CH), 121.1 (CH), 118.1 (CH), 116.8 (CH), 114.1 (CH), 113.9 (CH), 55.6 (CH_3), 55.5 (CH_3), 53.6 (CH_3), 53.2 (CH_3). **IR (neat)** v/cm^{-1}: **IR (neat)** v/cm^{-1}: 2970 (m), 2908 (m), 1731 (m), 1598 (m), 1462 (w), 1443 (w), 1279 (m), 1226 (s), 1166 (w), 1076 (s), 898 (w), 844 (w), 777 (m), 446 (w). **HR-MS (TOF ES+):** calcd for $C_{22}H_{21}N_2O_6$ 409.1394 found 409.1394.

Dimethyl 2,6-bis(2,4-difluorophenyl)pyrimidine-4,5-dicarboxylate (7f)

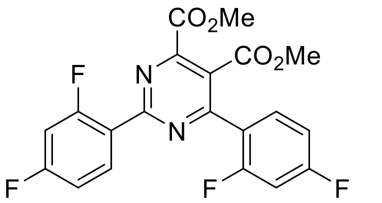

Chemical Formula: $C_{20}H_{12}F_4N_2O_4$
Molecular Weight: 420.32

Yield: 47% (79 mg, 0.19 mmol)

Appearance: Yellow solid

Melting point: 129 °C

^{1}H-NMR (400MHz, $CDCl_3$) δ/ppm 8.25 (m, 1H), 7.80 (m, 1H), 7.07 (m, 1H), 7.02 (m, 1H), 6.98-6.90 (m, 2H), 4.04 (s, 3H), 2.82 (s, 3H). **^{13}C-NMR (100 MHz, $CDCl_3$)** 165.5 (C), 165.2 (C), 165.1 (dd, *J* = 250, 12 Hz, CF), 164.7 (dd, *J* = 250, 12 Hz, CF), 162.5 (dd, *J* = 250, 12 Hz, CF), 161.4 (C), 160.5 (dd, *J* = 250, 12 Hz, CF), 160.5 (dd, *J* = 250, 12 Hz, CF), 157.5 (C), 133.9 (dd, *J* = 10, 2Hz, CH), 132.8 (dd, *J* = 10, 2 Hz, CH), 122.5 (C), 121.4 (m, C), 121.3 (m, C), 112.4 (dd, *J* = 22, 3Hz, CH), 111.9 (dd, *J* = 22, 3Hz, CH), 105.5 (t, *J* = 25 Hz, CH), 104.3 (t, *J* = 25 Hz, CH), 53.7 (CH_3), 53.2 (CH_3). **^{19}F-NMR (376 MHz, $CDCl_3$)** -104.3 (m), -105.2 (m), - 107.0 (m), -110.7 (m). **IR (neat)** ν/cm^{-1}: 2954 (m), 2670 (m), 1733 (s), 1617 (m), 1597 (m), 1544 (m), 1444 (m), 1430 (m), 1283 (s), 1226 (m), 1095 (s), 971 (m), 859 (m) 843 (w), 729 (w), 671 (w). **HR-MS (TOF ES+):** calcd for $C_{22}H_{13}F4N_2O_4$ 421.0806 found 421.0806.

Di-tert-butyl 2,6-diphenylpyrimidine-4,5-dicarboxylate (7g)

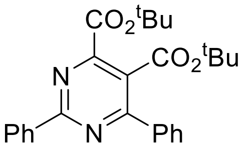

Chemical Formula: $C_{26}H_{28}N_2O_4$
Molecular Weight: 432.52

Yield: 83% (130 mg, 0.30 mmol)

Appearance: Yellow solid

Melting point: 180 °C

^{1}H-NMR (400 MHz, $CDCl_3$) δ/ppm 8.58 (m, 2H), 7.76 (m, 2H), 7.54-7.48 (m, 6H), 1.68 (s, 9H), 1.38 (s, 9H). **^{13}C-NMR (100 MHz, $CDCl_3$)** δ/ppm 165.8 (C), 165.6 (C), 164.5 (C), 164.1 (C), 157.9 (C), 138.0 (C), 146.7 (C), 131.6 (CH), 130.2 (CH), 129.0 (2CH), 129.0 (2CH), 128.7 (2CH), 128.5 (2CH), 123.0 (C), 84.1 (C), 83.5 (C), 28.2 ($3CH_3$), 27.8 ($3CH_3$). **IR (neat)** ν/cm^{-1}: 2975 (w), 1740 (m), 1717 (s), 1543 (s), 1530 (m), 1366 (s), 1230 (s), 1144 (s), 1080 (s), 835 (w), 742 (w), 692 (s). **HR-MS (TOF ES+):** calcd for $C_{28}H_{29}N_2O_4$ 433.2122 found 433.2123.

Di-tert-butyl 2,6-bis(4-methoxyphenyl)pyrimidine-4,5-dicarboxylate (7h)

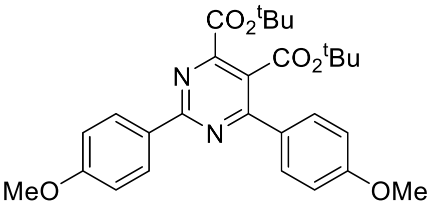

Chemical Formula: $C_{28}H_{32}N_2O_6$
Molecular Weight: 492.57

Yield: 71% (73 mg, 0.14 mmol)

Appearance: White solid

Melting point: 156 °C

^{1}H-NMR (500 MHz, CDCl$_3$) δ/ppm 8.53 (m, 2H), 7.76 (m, 2H), 7.01-6.97 (m, 4H), 3.88 (s, 3H), 3.88 (s, 3H), 1.67 (s, 9H), 1.43 (s, 9H). **^{13}C-NMR (125 MHz, CDCl$_3$)** δ/ppm 166.1 (C), 164.8 (C), 164.7 (C), 163.6 (C), 162.5 (C), 161.5 (C), 157.8 (C), 130.8 (2CH), 130.7 (2CH), 130.5 (C), 129.5 (C), 121.7 (C), 113.9 (2CH), 113.9 (2CH), 83.8 (C), 83.3 (C), 55.6 (CH_3) 55.5 (CH_3), 28.2 (3CH_3), 27.9 (3CH_3). **IR (neat)** v/cm^{-1}: 3969 (w), 1721 (m), 1606 (m), 1543 (m), 1390 (m), 1251 (s), 1146 (s), 1073 (m), 816 (m), 616 (w). **HR-MS (TOF ES+):** calcd for $C_{28}H_{33}N_2O_6$ 493.2333 found 493.2335.

Di-tert-pentyl 2,6-bis(4-fluorophenyl)pyrimidine-4,5-dicarboxylate (7i)

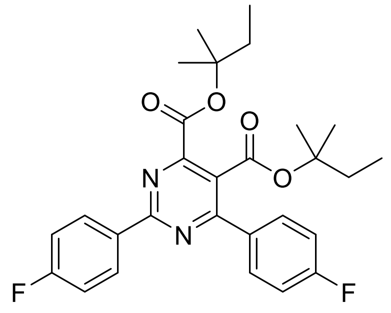

Chemical Formula: $C_{28}H_{30}F_2N_2O_4$
Molecular Weight: 496.55

Yield: 55% (60 mg, 0.12 mmol)

Appearance: White solid

Melting point: °C

^{1}H-NMR (400 MHz, CDCl$_3$) δ/ppm 8.57 (m, 2H), 7.78 (m, 2H), 7.20-7.14 (m, 4H), 1.99 (q, *J* = 7.5 Hz, 2H), 1.64 (s, 6H), 1.61 (q, *J* = 7.5 Hz, 2H), 1.06 (t, *J* = 7.5 Hz, 3H), 0.67 (t, *J* = 7.5 Hz, 3H). **^{13}C-NMR (100 MHz, CDCl$_3$)** δ/ppm 165.5 (C), 165.1 (d, *J* = 250 Hz, C), 164.1 (d, *J* = 250 Hz, C), 164.2 (C), 164.1 (C), 162.8 (C), 157.7 (C), 134.0 (d, *J* = 3 Hz, C), 132.8 (d, *J* = 3 Hz, C), 131.2 (d, *J* = 4 Hz, 2CH), 131.1 (d, *J* = 4 Hz, 2CH), 123.1 (C), 115.8 (d, *J* = 4 Hz, 2CH), 115.6 (d, *J* = 7 Hz, 2CH), 86.7 (C), 86.5 (C), 34.4 (CH_2), 33.7 (CH_2), 25.6 (2CH_3), 24.7 (2CH_3), 8.4 (CH_3), 8.0 (CH_3). **^{19}F-NMR (376 MHz, CDCl$_3$)** -108.8 (m), -110.3 (m). **IR (neat)** v/cm^{-1}: 2977 (w), 1713 (s), 1600 (w), 1543 (m), 1509 (m), 1383 (m), 1233 (s), 1145 (s), 1079 (s), 924 (w), 795 (m(, 579 (w). **HR-MS (TOF ES+):** calcd for $C_{28}H_{31}F_2N_2O_4$ 497.2246 found 497.2248.

Dimethyl 2,6-di-*o*-tolylpyrimidine-4,5-dicarboxylate (7j)

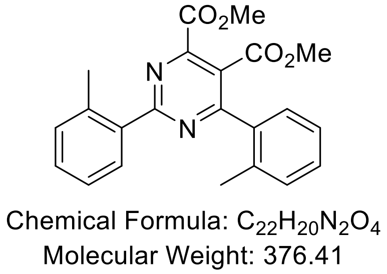

Yield: 77% (133 mg, 0.35 mmol)

Appearance: Yellow oil

^{1}H-NMR (500 MHz, CDCl$_3$) δ/ppm 7.98 (m, 1H), 7.38 (m, 2H), 7.34-7.31 (m, 3H), 7/27 (m, 2H), 4.05 (s, 3H), 3.69 (s, 3H), 2.63 (s, 3H), 2.30 (s, 3H). **^{13}C-NMR (125 MHz, CDCl$_3$)** 167.7 (C), 167.5 (C), 166.5 (C), 165.0 (C), 154.1 (C), 138.2 (C), 136.7 (C), 136.5 (C), 136.1 (C), 131.7 (CH), 131.2 (CH), 130.7 (CH), 130.5 (CH), 129.7 (CH), 128.3 (CH), 126.2 (CH), 125.7 (CH), 124.0 (C), 53.7 (CH_3), 53.0 (CH_3), 21.6 (CH_3), 19.9 (CH_3). **IR (neat)** v/cm^{-1}: 3065 (w), 3040 (w), 1730 (s), 1603 (w), 1543 (s), 1491 (s), 1436 (w), 1392 (m), 1268 (m), 1225 (s), 1173 (s), 1127 (m), 1037 (m), 973 (w), 859 (w), 740 (s), 641 (w). **HR-MS (TOF ES+):** $_4$ calcd for $C_{22}H_{21}N_2O$ 377.1499 found 377.1496**.**

Di-tert-butyl 2,6-bis(4-fluorophenyl)pyrimidine-4,5-dicarboxylate (7k)

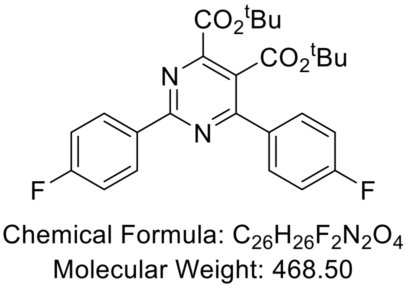

Yield: 66% (89 mg, 0.19 mmol)

Appearance: Yellow oil

^{1}H-NMR (400 MHz, CDCl$_3$) δ/ppm 8.57 (m, 2H), 7.78 (m, 2H), 7.17 (m, 4H), 1.67 (s, 9H), 1.41 (s, 9H). **^{13}C-NMR (100 MHz, CDCl$_3$)** δ/ppm 165.3 (d, J = 250 Hz, C), 165.5 (C), 164.5 (C), 164.3 (C), 164.2 (d, J = 250 Hz, C), 158.0 (C), 163.1 (C), 133.9 (d, J = 3 Hz, C), 132.7 (d, J = 3 Hz, C), 131.2 (d, J = 9 Hz, 2CH), 131.1 (d, J = 9 Hz, 2CH), 122.9 (C), 115.80 (d, J = 9 Hz, 2CH), 115.5 (d, J = 9 Hz, 2CH), 84.2 (C), 83.8 (C), 28.2 (3CH_3), 27.8 (3CH_3). **^{19}F-NMR (376 MHz, CDCl$_3$)** **IR (neat)** v/cm^{-1}: 2981 (w), 1738 (w), 1714 (m), 1601 (w), 1542 (w), 1508 (w). 1380 (w), 1294 (w), 1147 (s), 844 (w), 748 (w), 636 (w). **HR-MS (TOF ES+):** calcd for $C_{26}H_{27}F_2N_2O_4$ 469.1933 found 469.1934.

X-Ray data

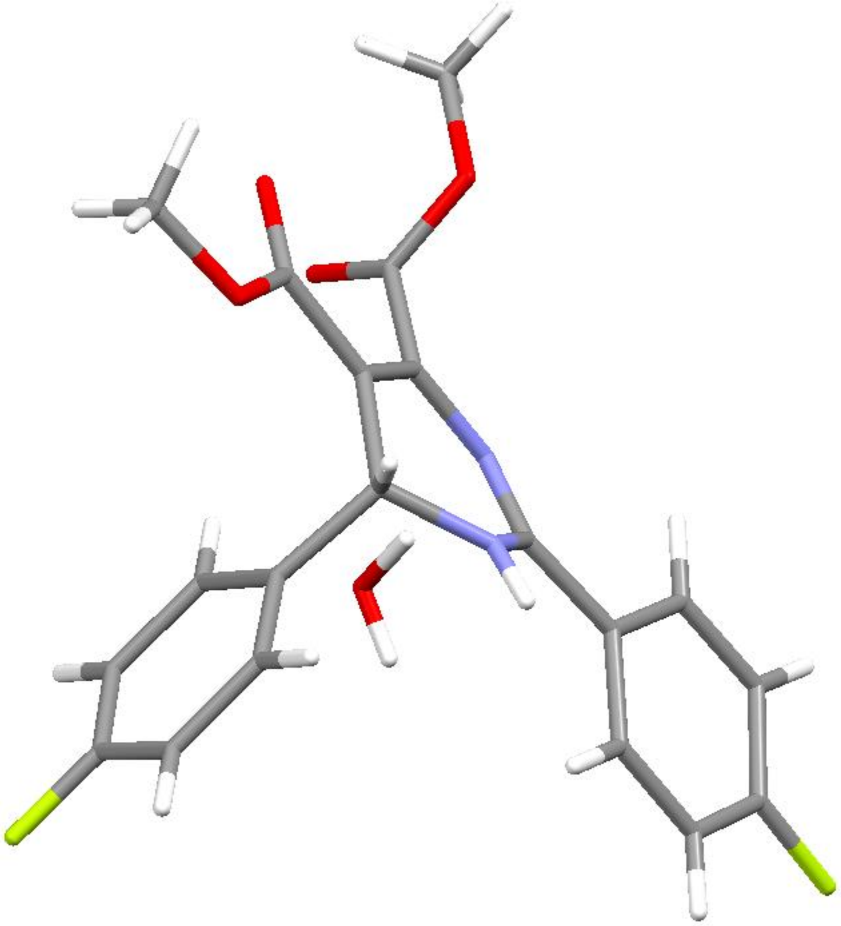

Identification Code	rc-b-5-169_exp1744
CCDC Code	2347211
Empirical F	C_{20} H_{16} F_2 N_2 O_4, H_2 O
Space Group	**I a**
a/Å	8.28920 (10)
b/Å	17.7499 (2)
c/Å	12.7111 (2)
α/Å	90
β/Å	100.0580 (10)
γ/ Å	90
Cell volume	1841.47
Z, Z’	**Z**: 4 **Z'**: 0
R-factor (%)	2.87

Pictures of the flow equipment

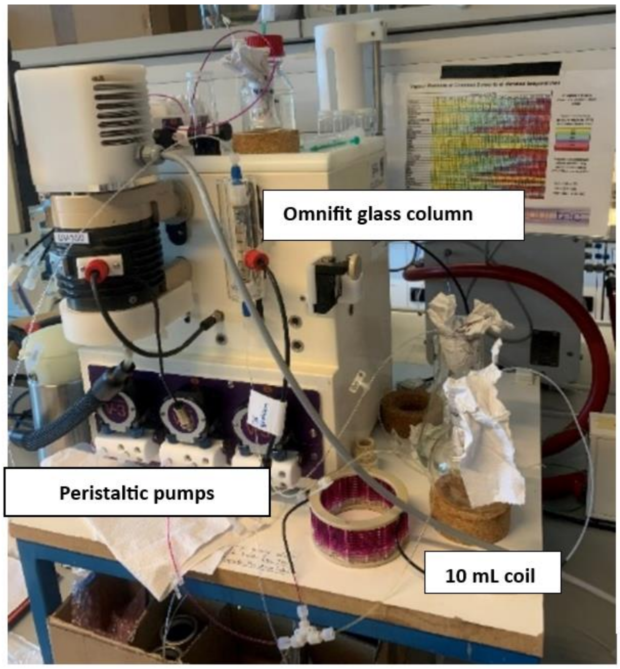

Figure 1. Picture of the telescoped set-up for the tautomerisation and oxidation.

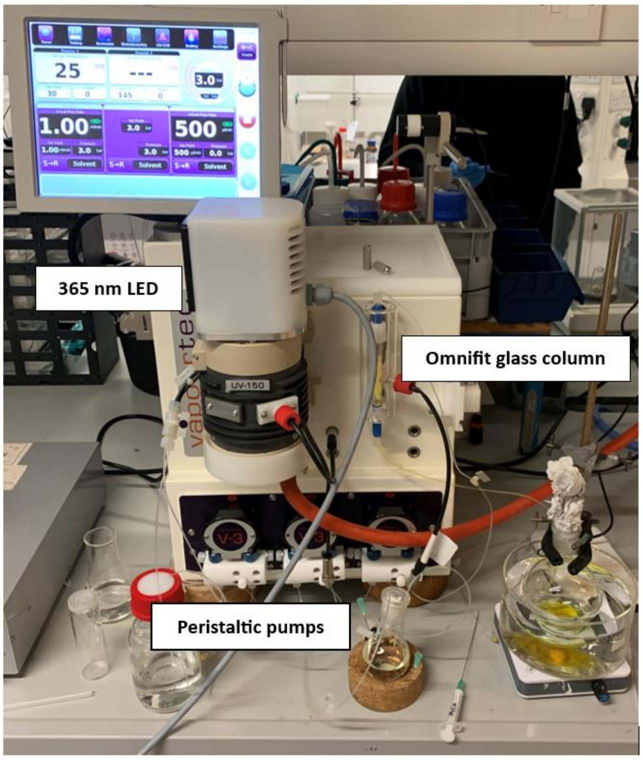

Figure 2. Picture of the telescoped synthesis of pyrimidine-4,5-dicarboxylate (7b).

References

(1) Baykal, A.; Plietker, B. A Bu4N[Fe(CO)3NO)]-Catalyzed Hemetsberger–Knittel Indole Synthesis. *Eur. J. Org. Chem.* **2020**, *2020* (9), 1145-1147. DOI: 10.1002/ejoc.201901864.

(2) Bonnamour, J.; Bolm, C. Iron(II) Triflate as a Catalyst for the Synthesis of Indoles by Intramolecular C−H Amination. *Org. Lett.* **2011**, *13* (8), 2012-2014. DOI: 10.1021/ol2004066.

(3) Wang, Y.; Lei, X.; Tang, Y. Rh(ii)-catalyzed cycloadditions of 1-tosyl 1,2,3-triazoles with 2H-azirines: switchable reactivity of Rh-azavinylcarbene as [2C]- or aza-[3C]-synthon. *Chem. Commun.* **2015**, *51* (21), 4507-4510. DOI: 10.1039/C5CC00268K.

(4) O'Brien, A. G.; Levesque, F.; Seeberger, P. H. Continuous flow thermolysis of azidoacrylates for the synthesis of heterocycles and pharmaceutical intermediates. *Chem. Commun.(Camb)* **2011**, *47* (9), 2688-2690. DOI: 10.1039/c0cc04481d From NLM Medline.

(5) Nguyen, T. K.; Titov, G. D.; Khoroshilova, O. V.; Kinzhalov, M. A.; Rostovskii, N. V. Light-induced one-pot synthesis of pyrimidine derivatives from vinyl azides. *Org. Biomol. Chem.* **2020**, *18* (26), 4971-4982. DOI: 10.1039/d0ob00693a.

Copies of NMR Data

Methyl 2-azidoacetate

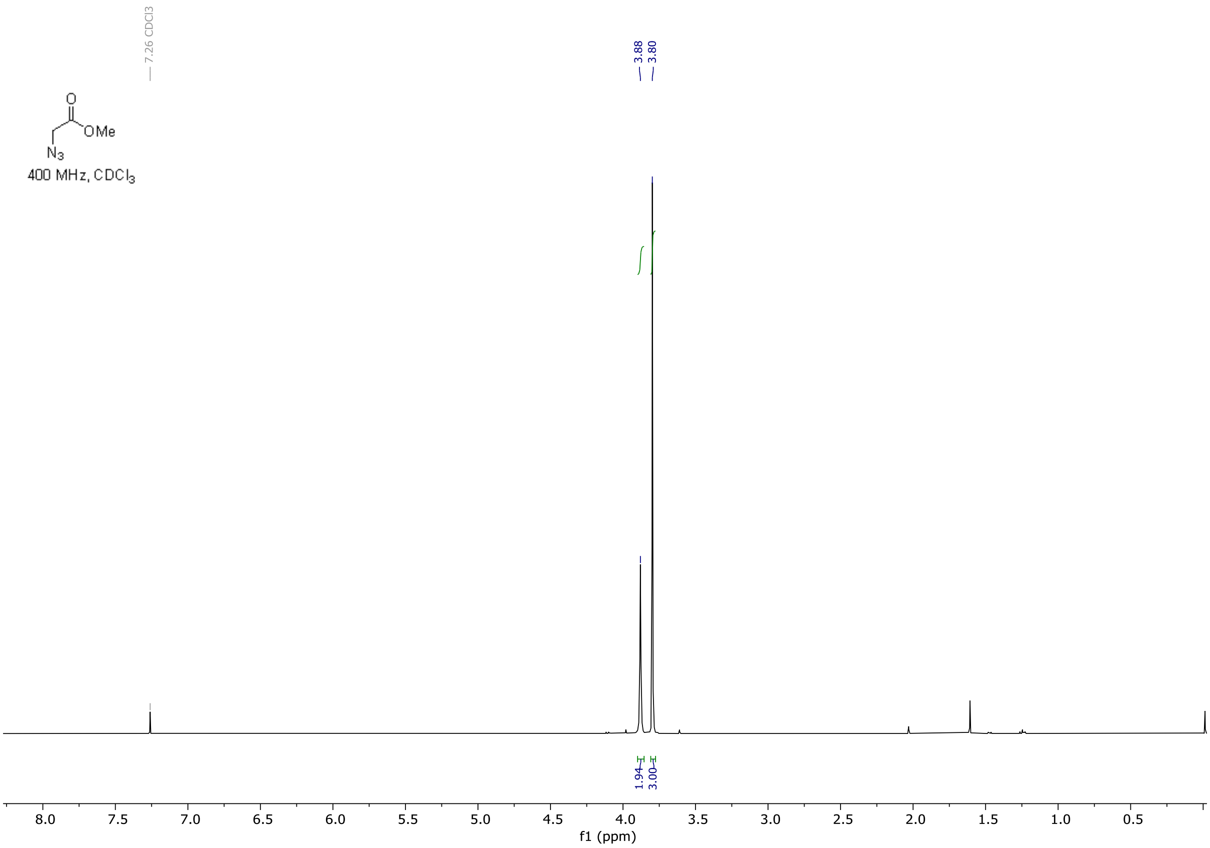

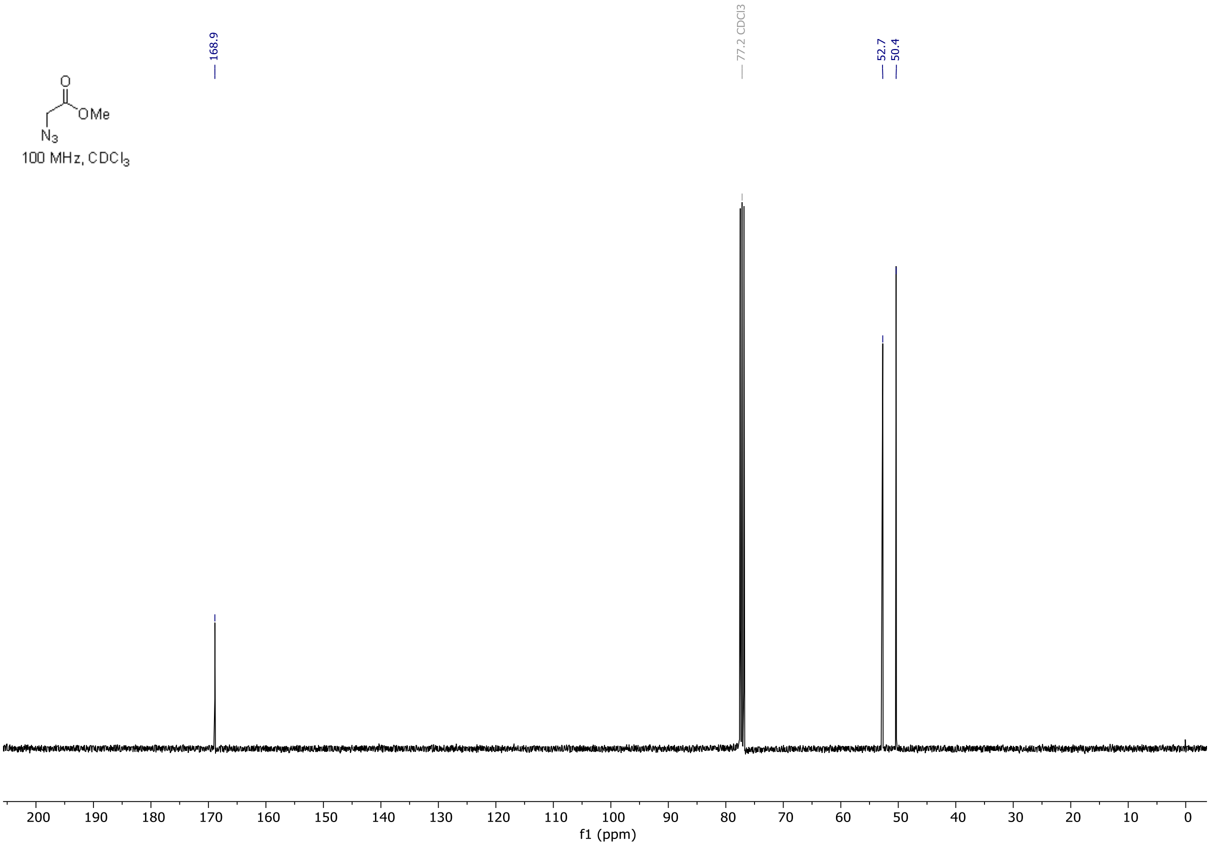

Methyl (*Z*)-2-azido-3-phenylacrylate (1a)

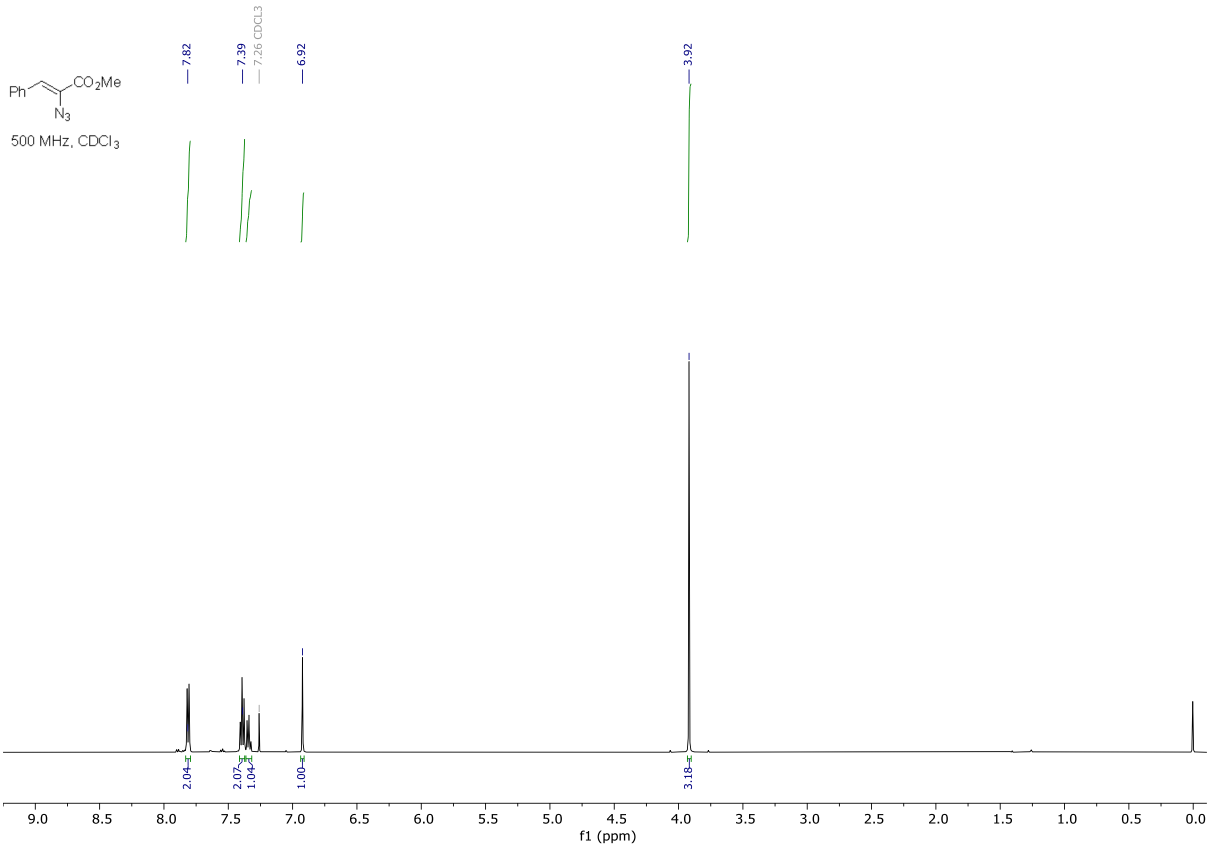

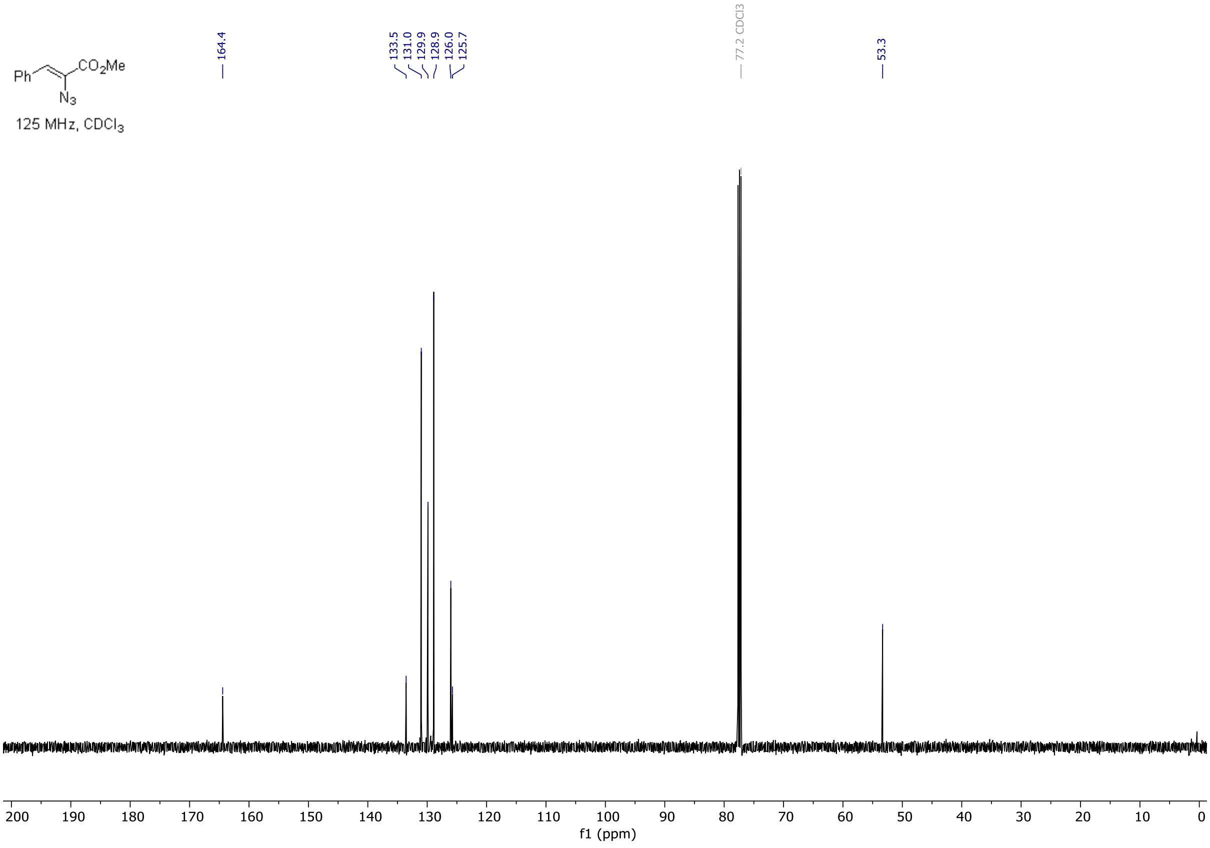

Methyl (*Z*)-2-azido-3-(4-fluorophenyl)acrylate (1b)

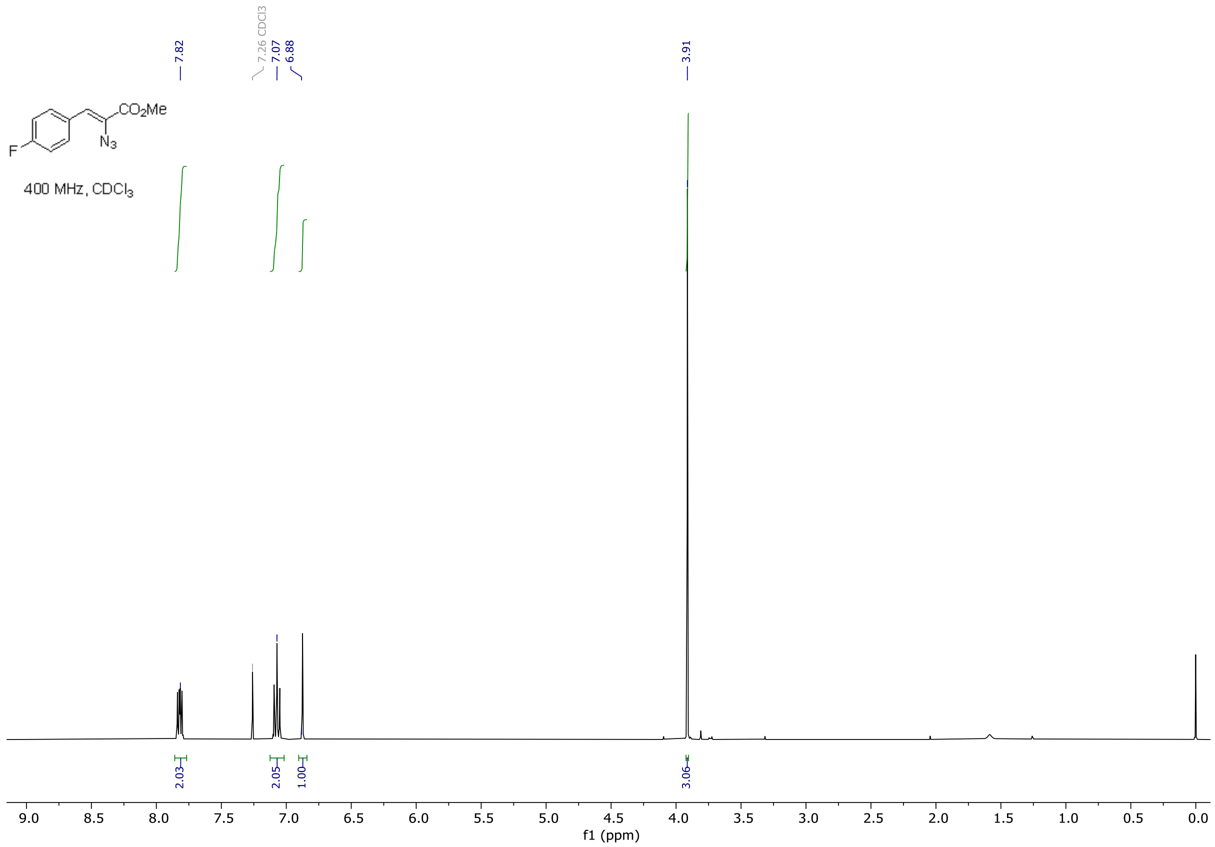

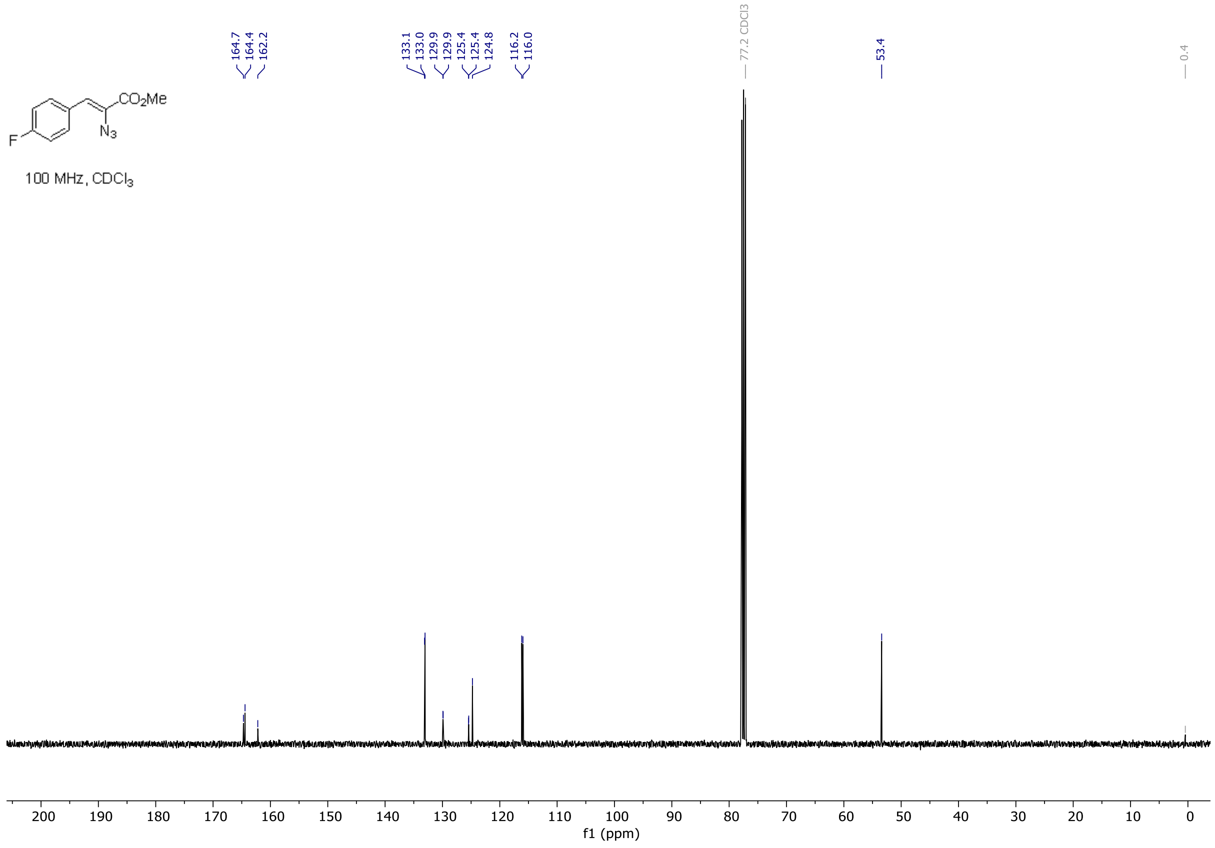

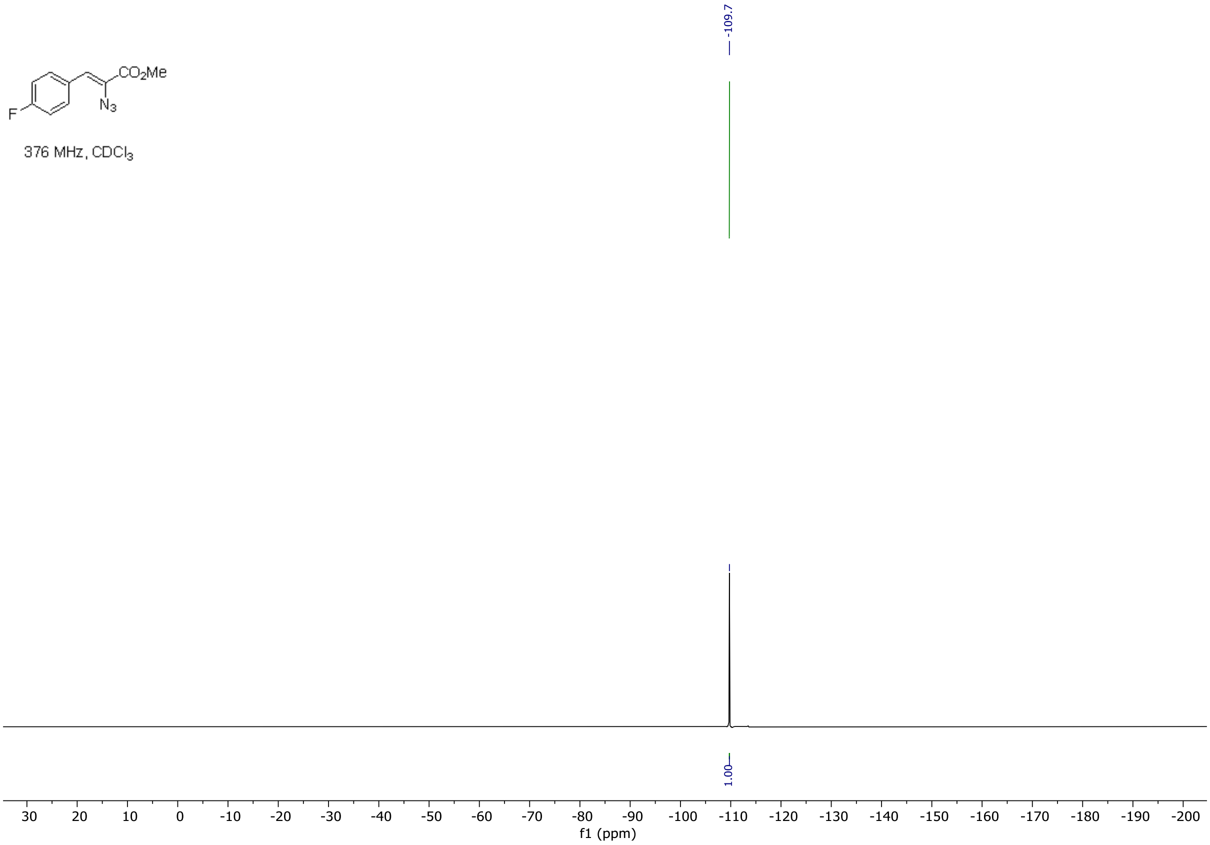
CO2Me
N3
F
376 MHz, CDCl3
-109.7
1.00
30
20
10
0
-10
-20
-30
-40
-50
-60
-70
-80
-90
-100
-110
-120
-130
-140
-150
-160
-170
-180
-190
-200
f1 (ppm)

Methyl (Z)-2-azido-3-(4-bromophenyl)acrylate (1c)

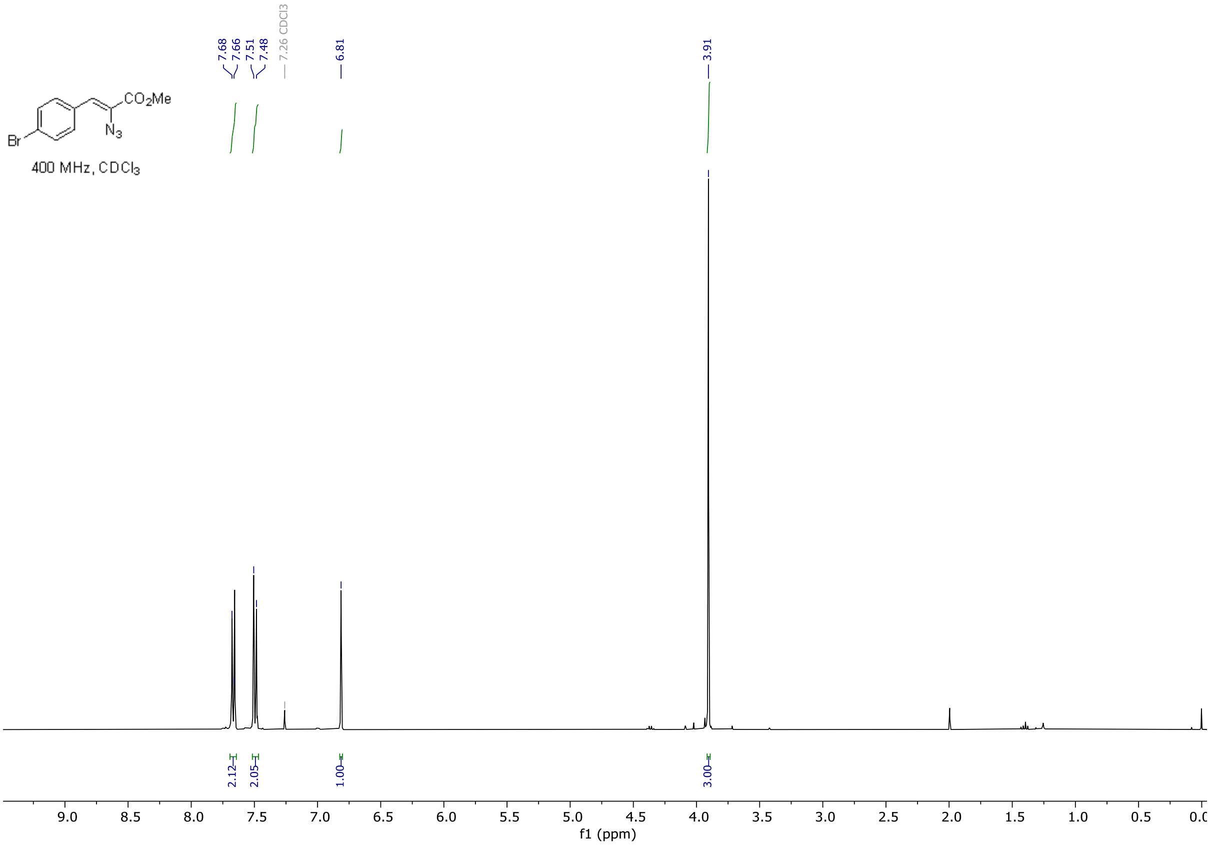

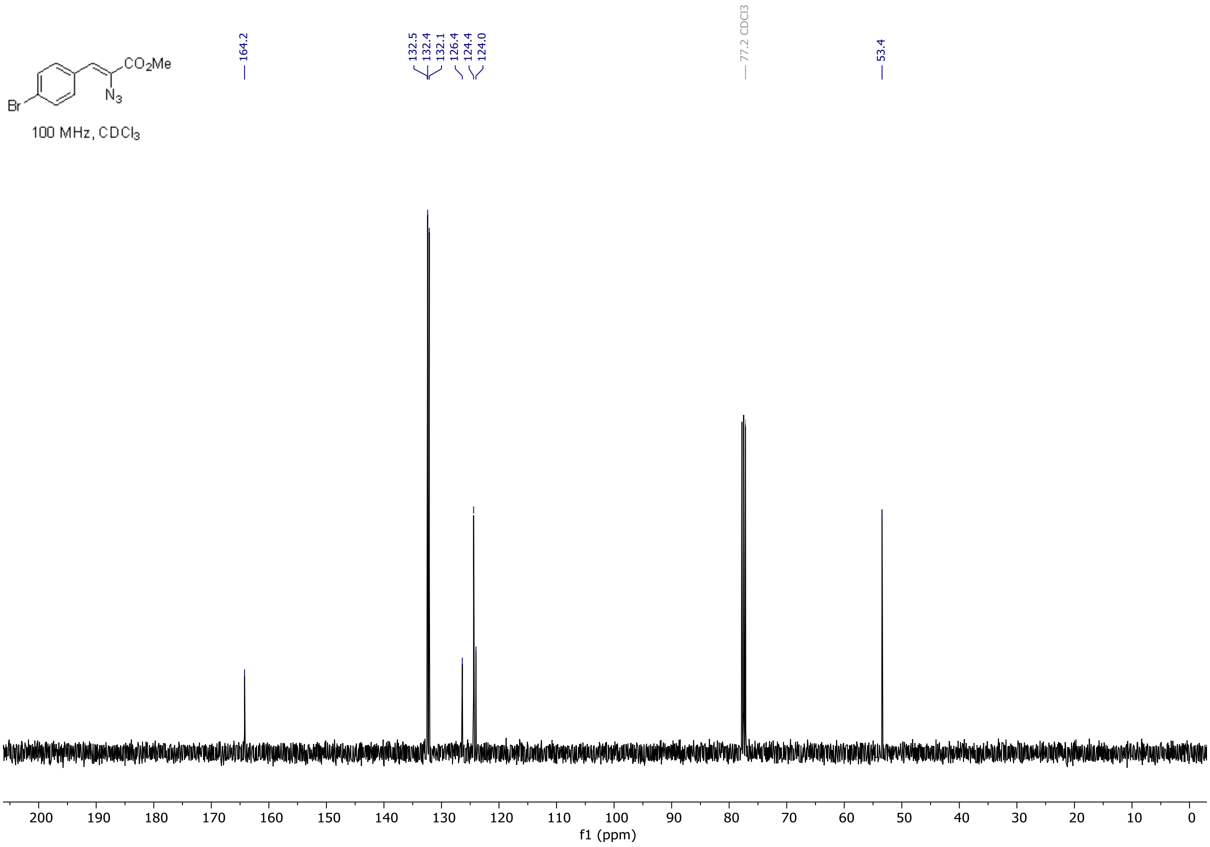

Methyl (*Z*)-2-azido-3-(p-tolyl)acrylate (1d)

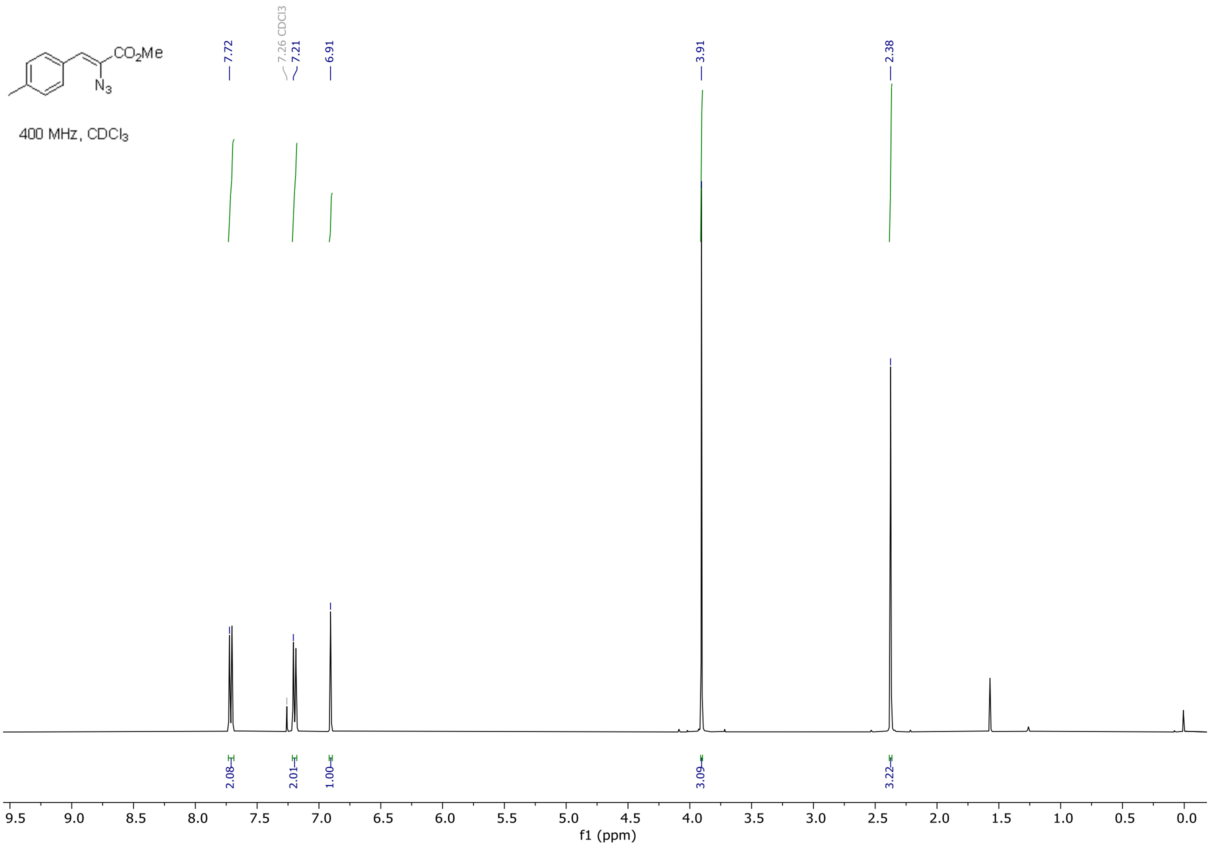

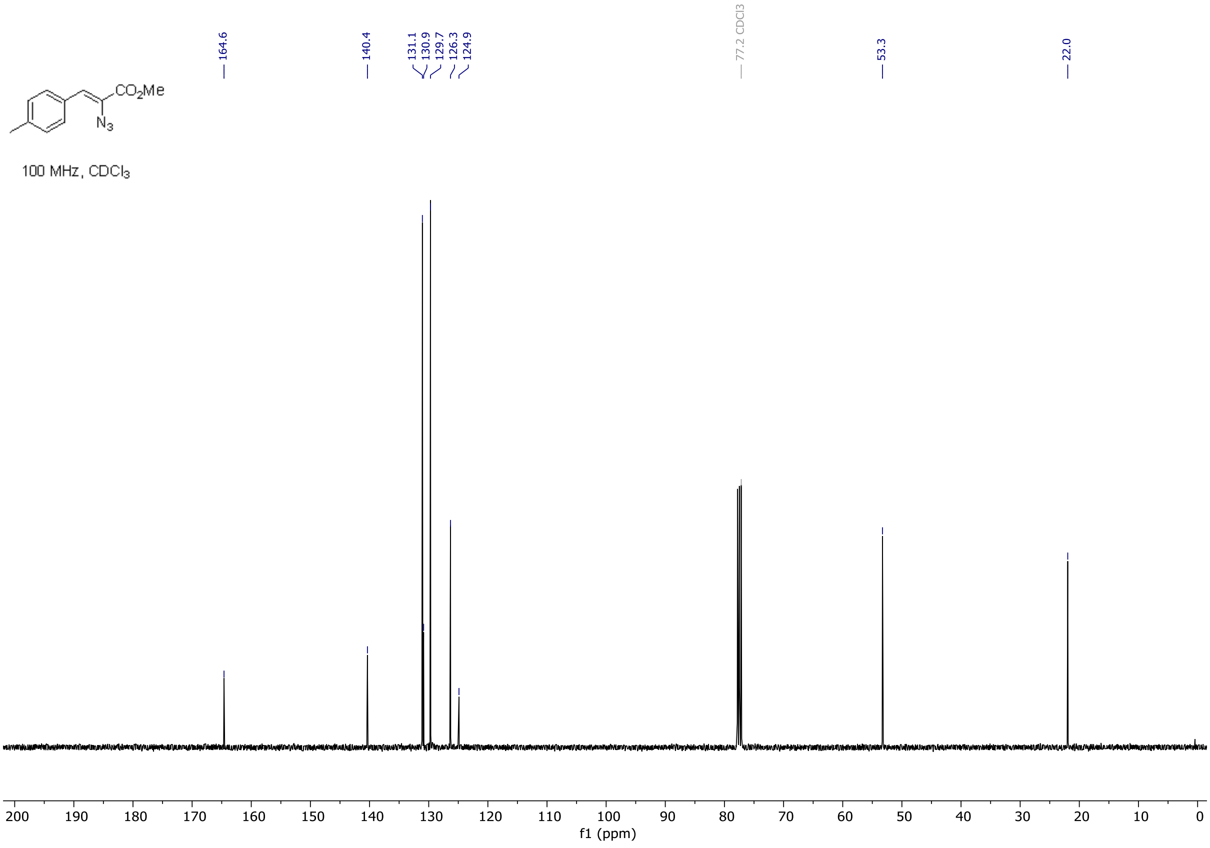

Methyl (*Z*)-2-azido-3-(3,4-dimethylphenyl)acrylate (1e)

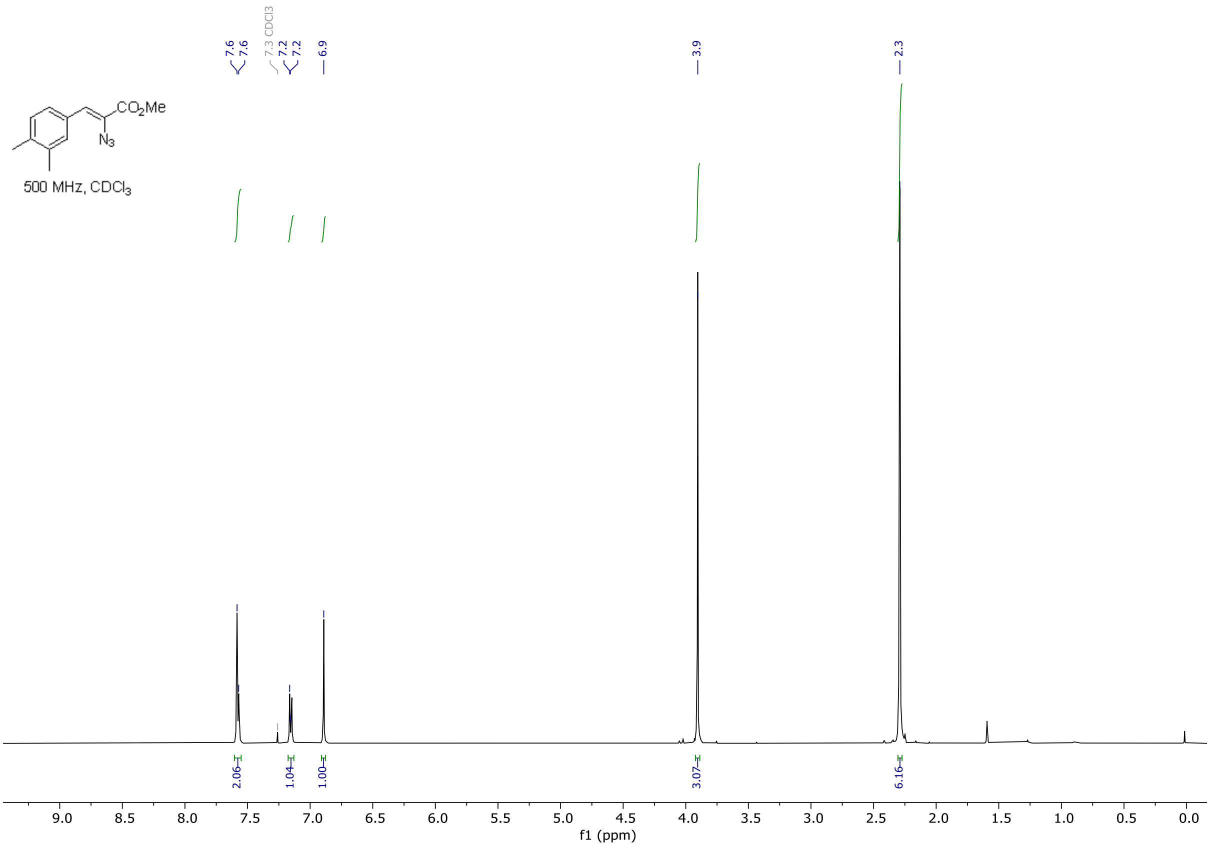

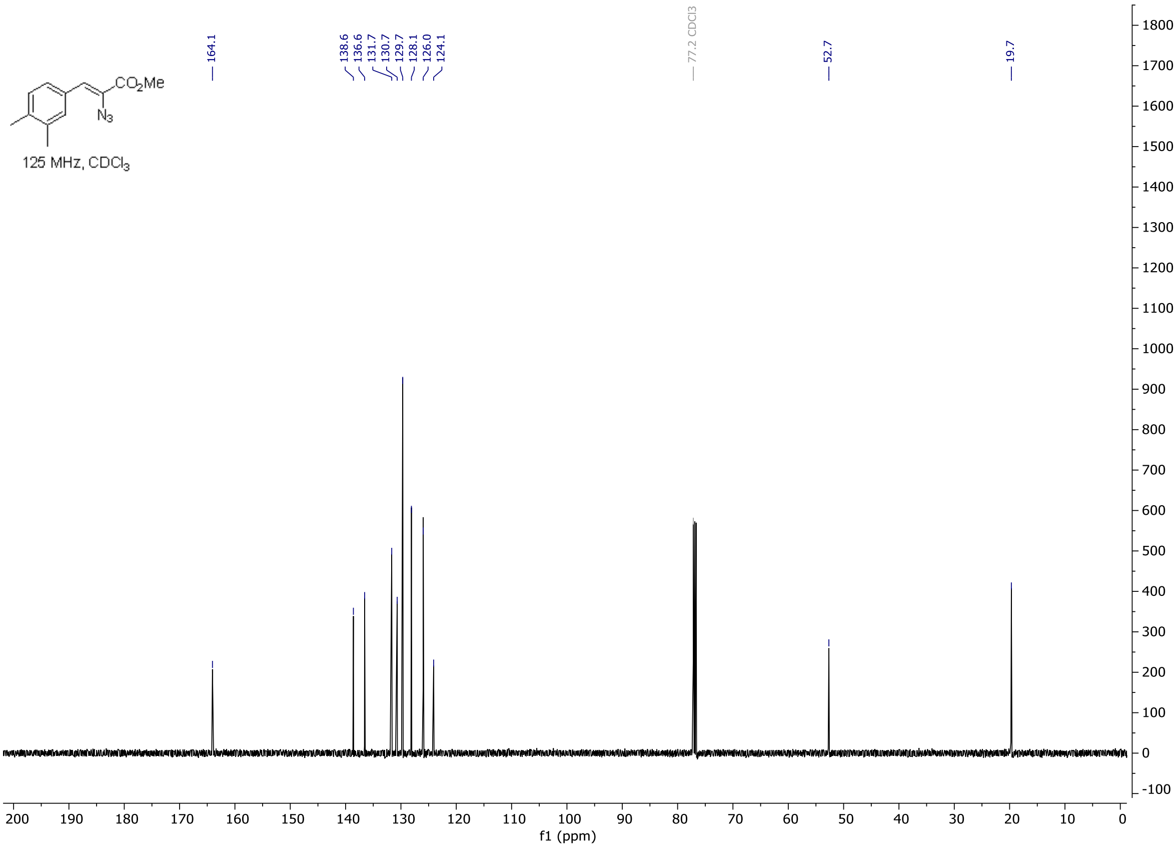

Methyl (*Z*)-2-azido-3-(o-tolyl)acrylate (1f)

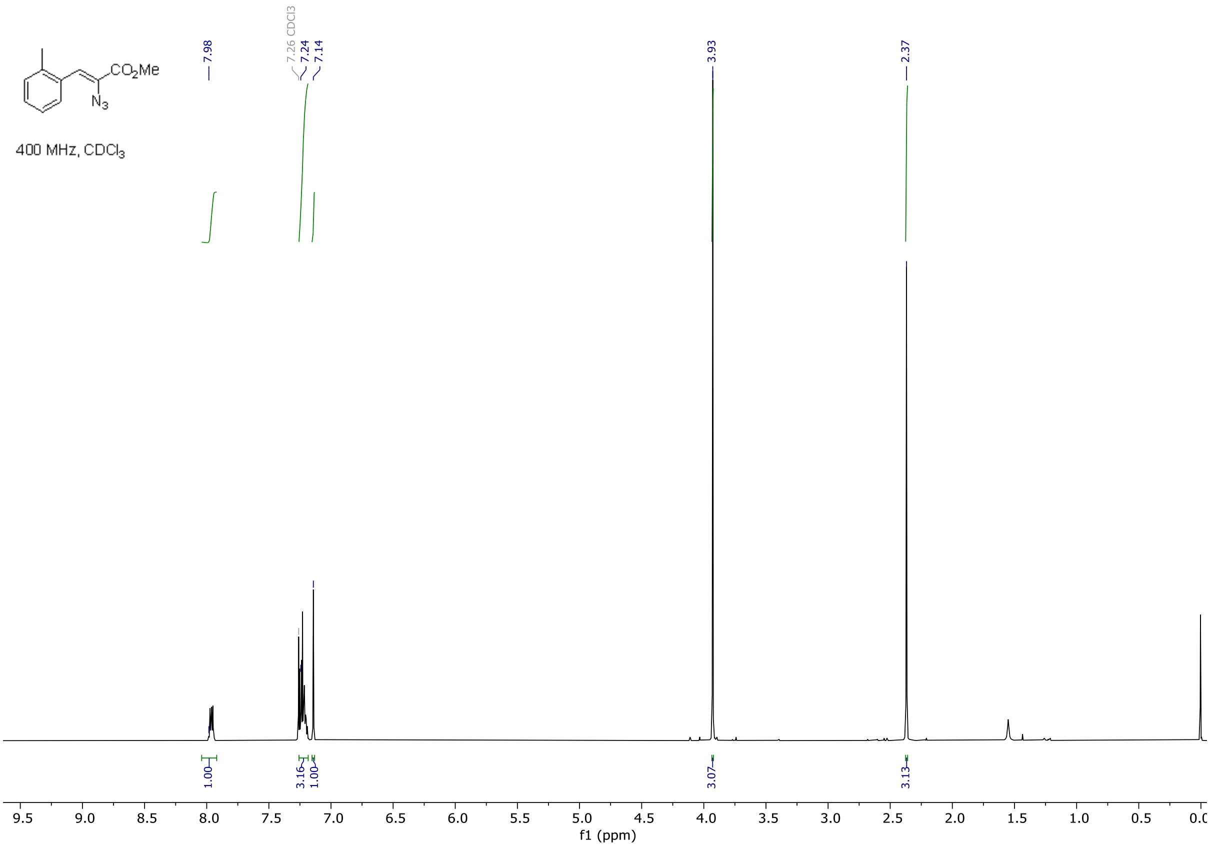

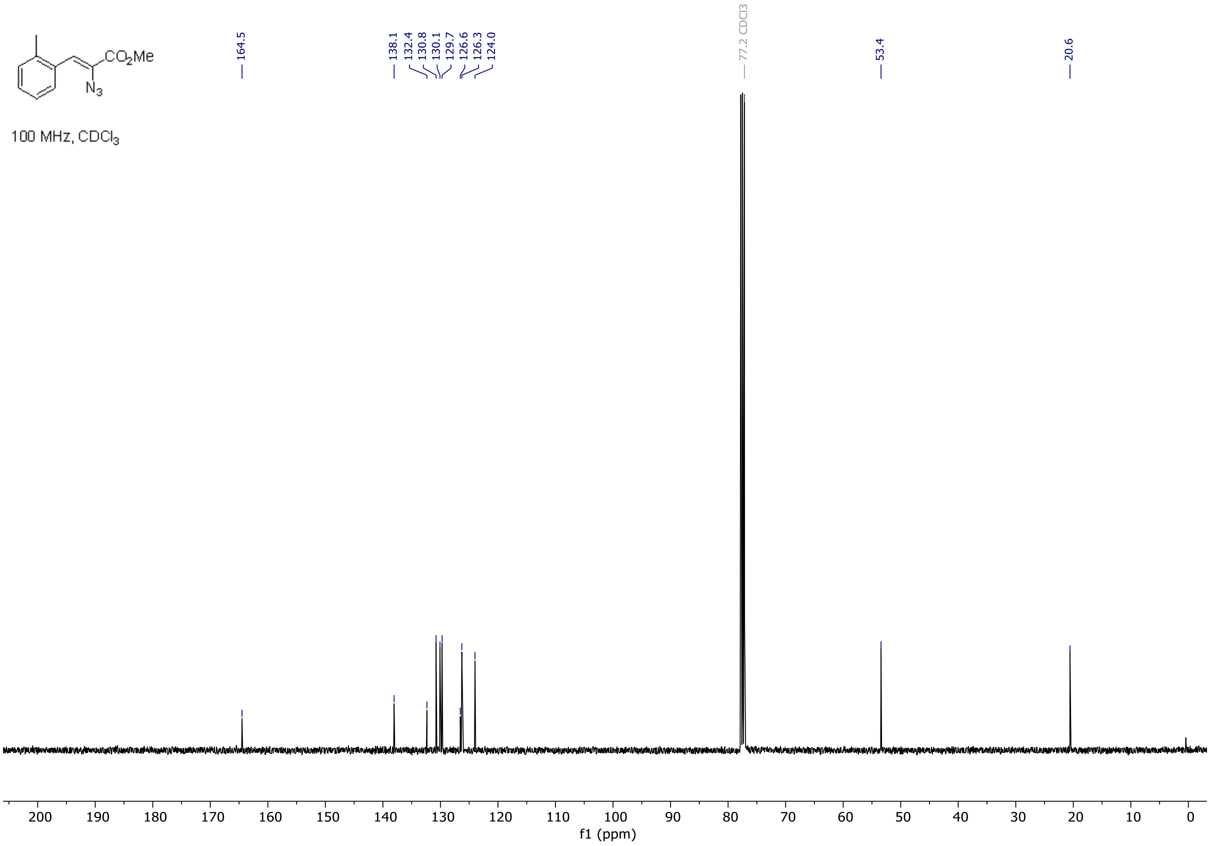

Methyl (*Z*)-2-azido-3-(4-methoxyphenyl)acrylate (1g)

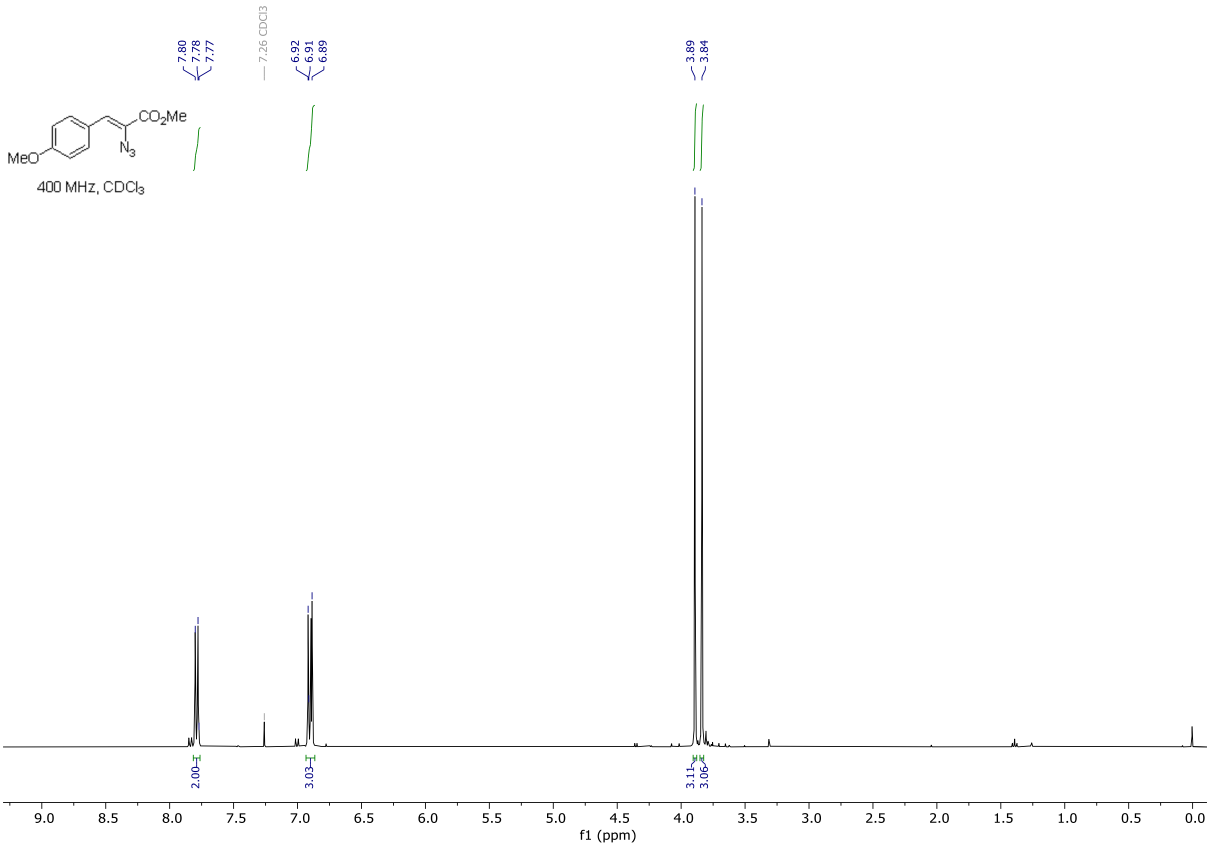

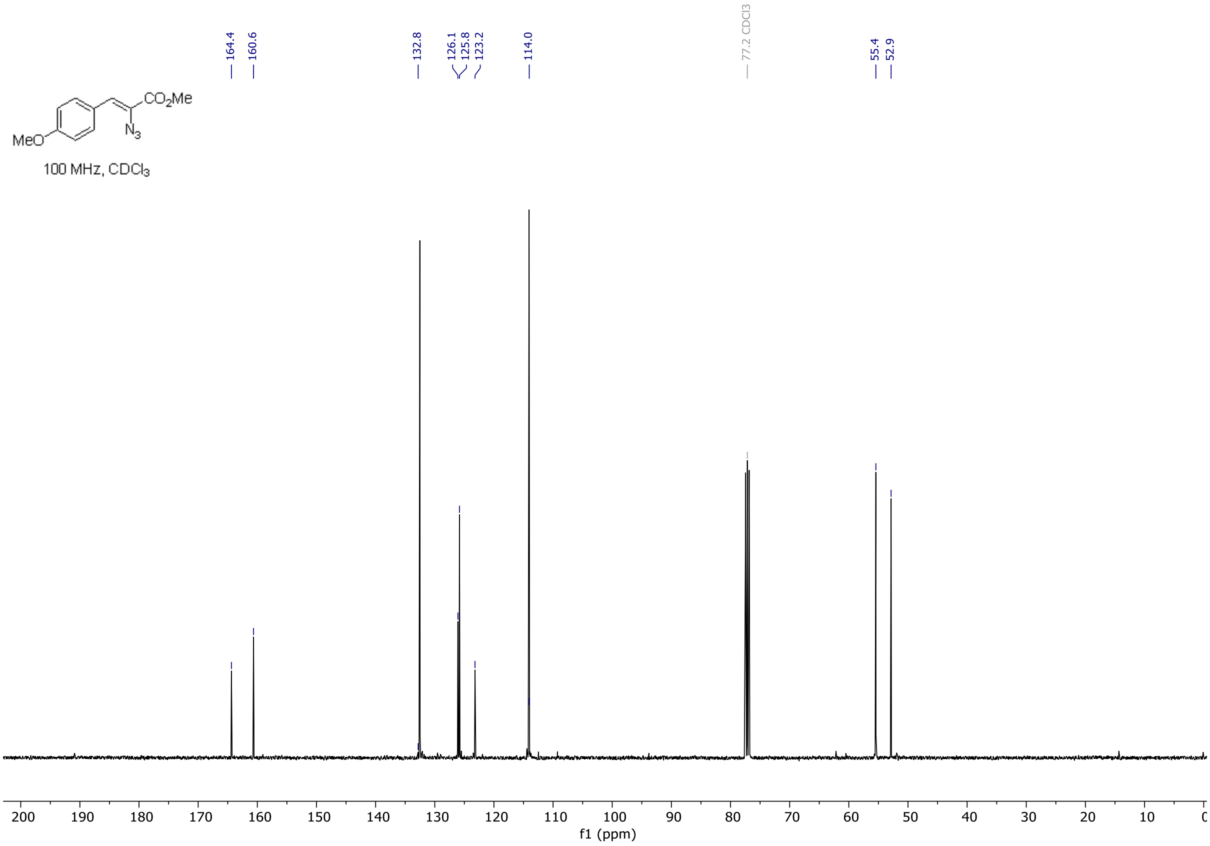

Methyl (*Z*)-2-azido-3-cyclohexylacrylate (1h)

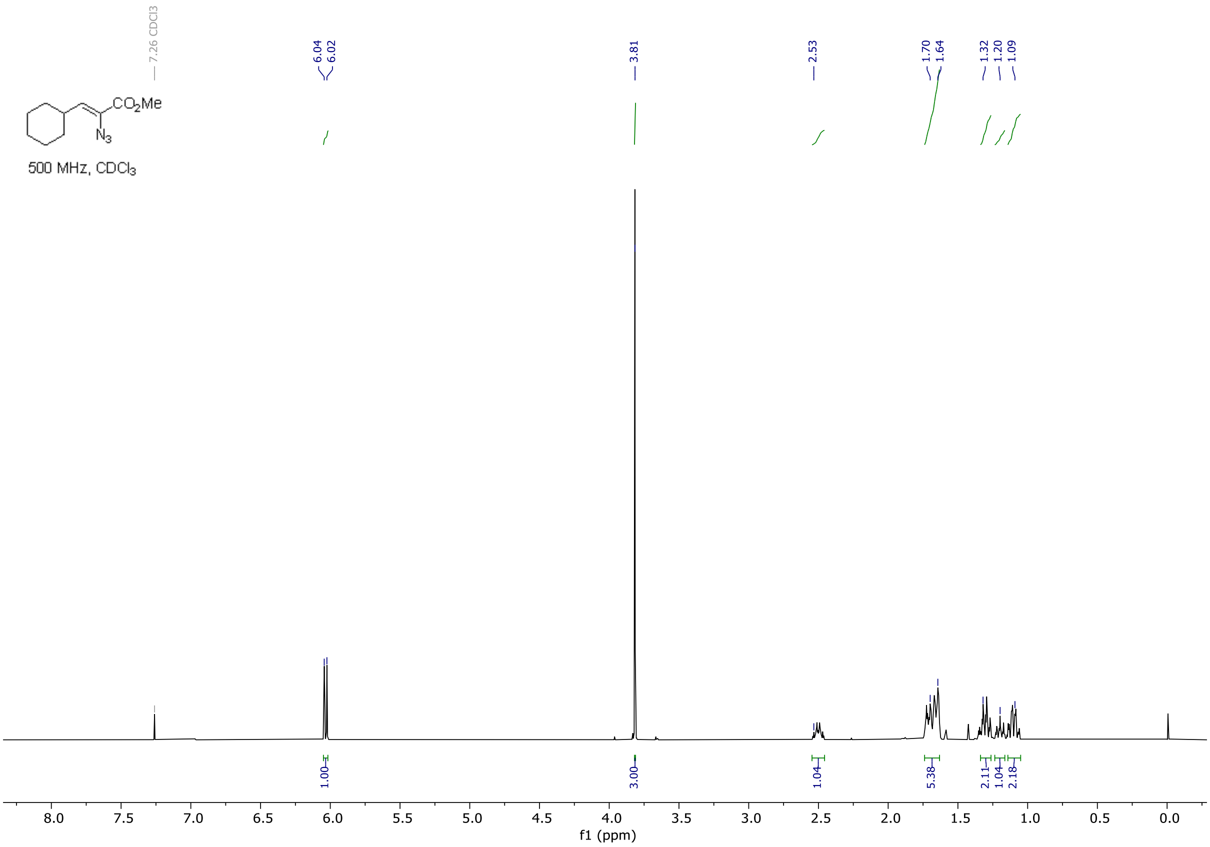

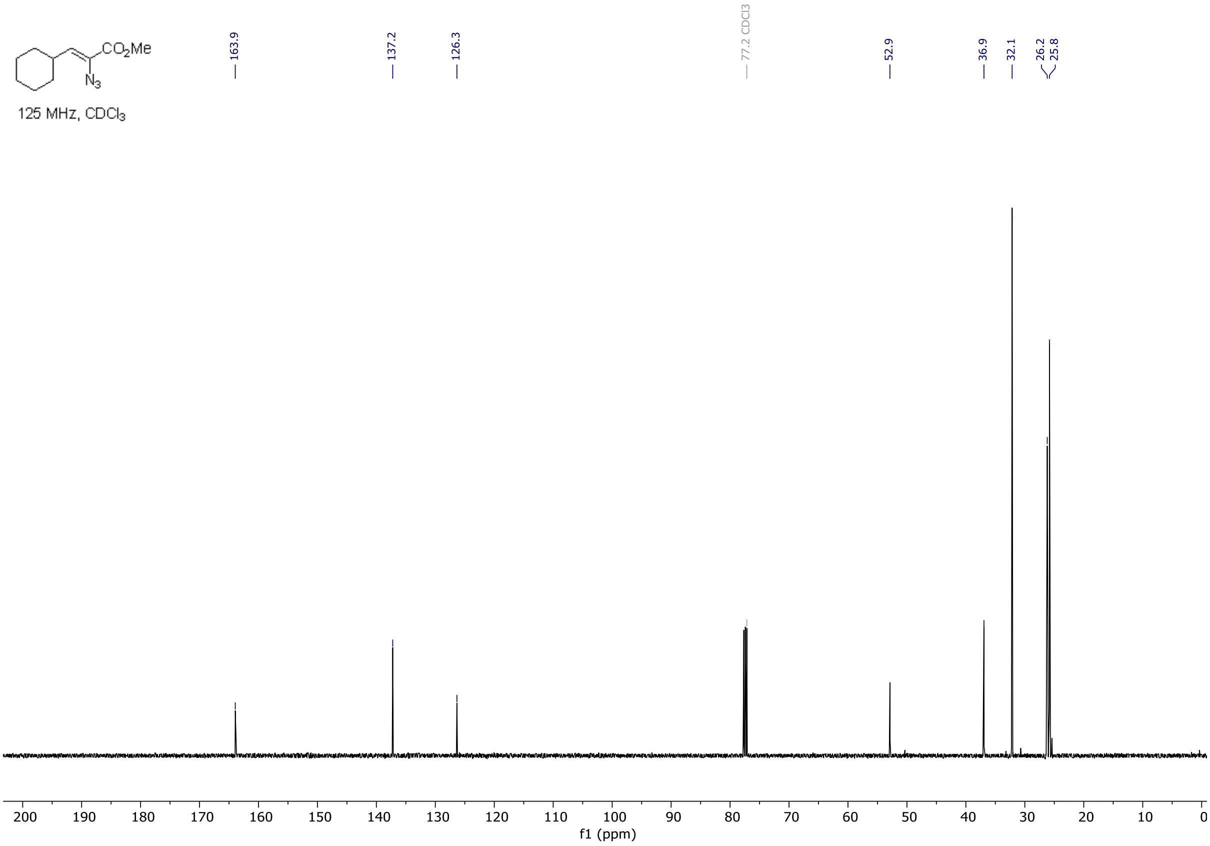

Methyl (Z)-2-azido-3-(3-methoxyphenyl)acrylate (2i)

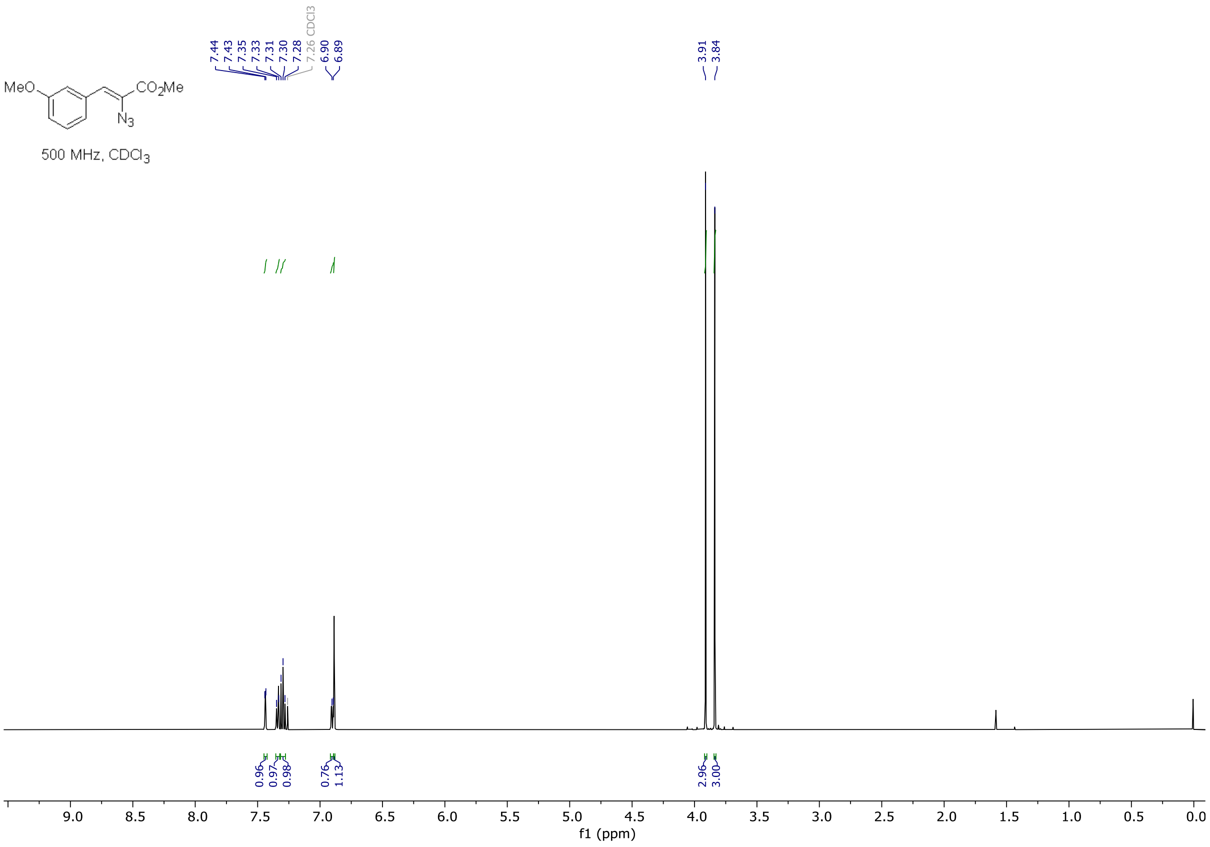

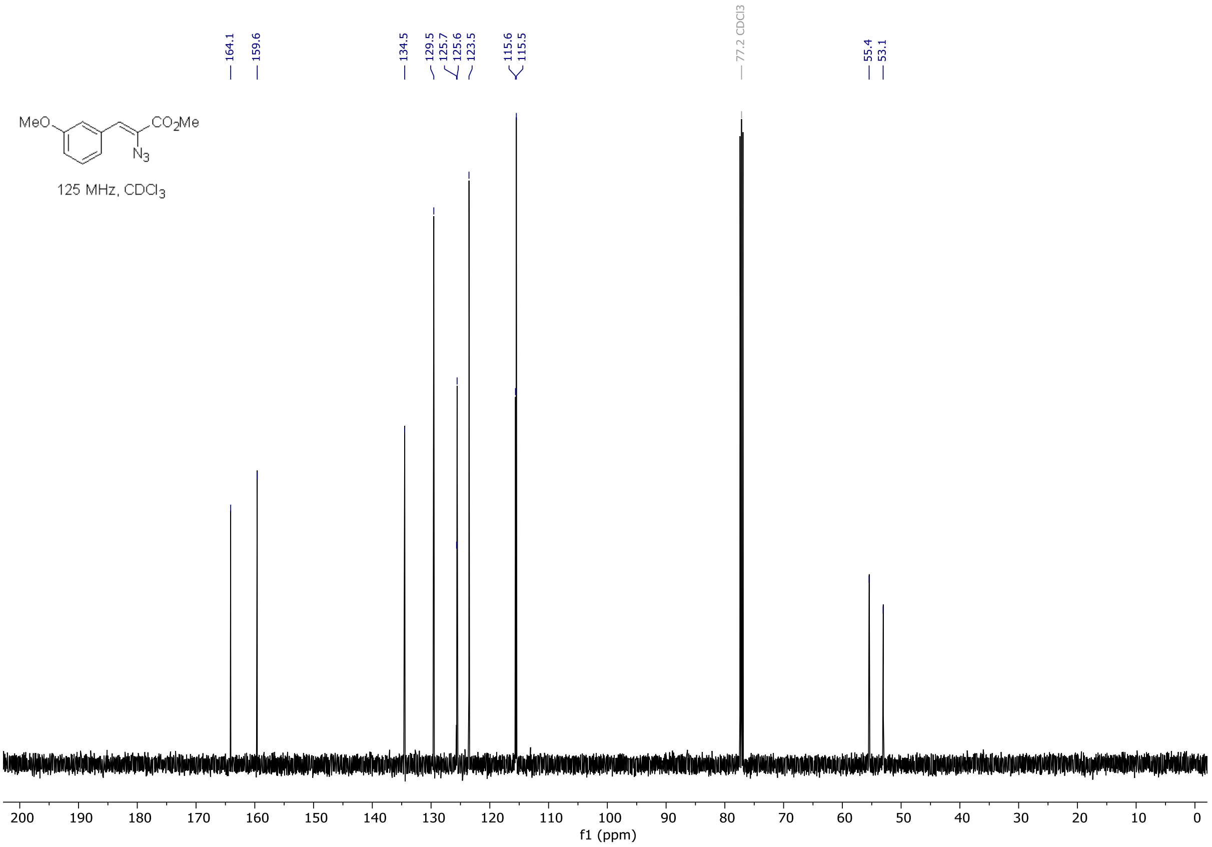

Methyl (*Z*)-2-azido-3-(2,4-difluorophenyl)acrylate (2j)

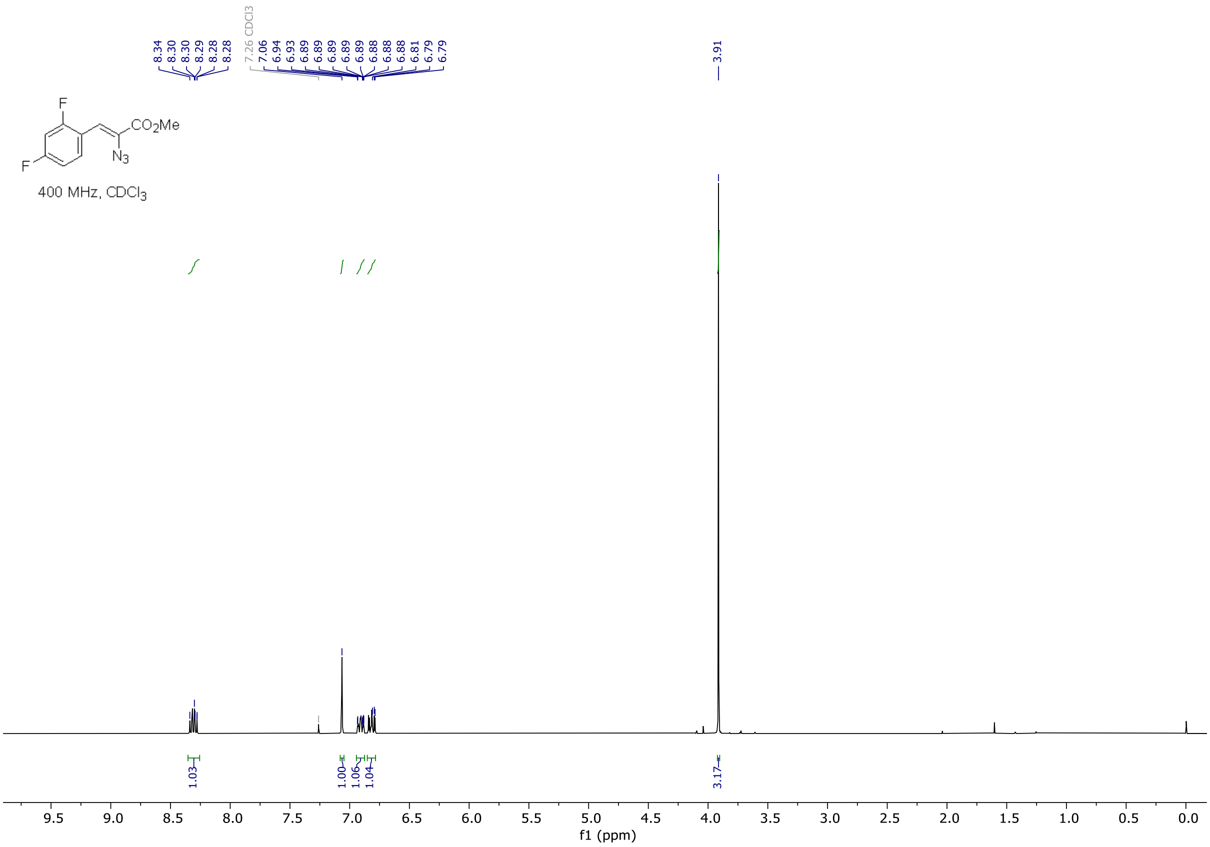

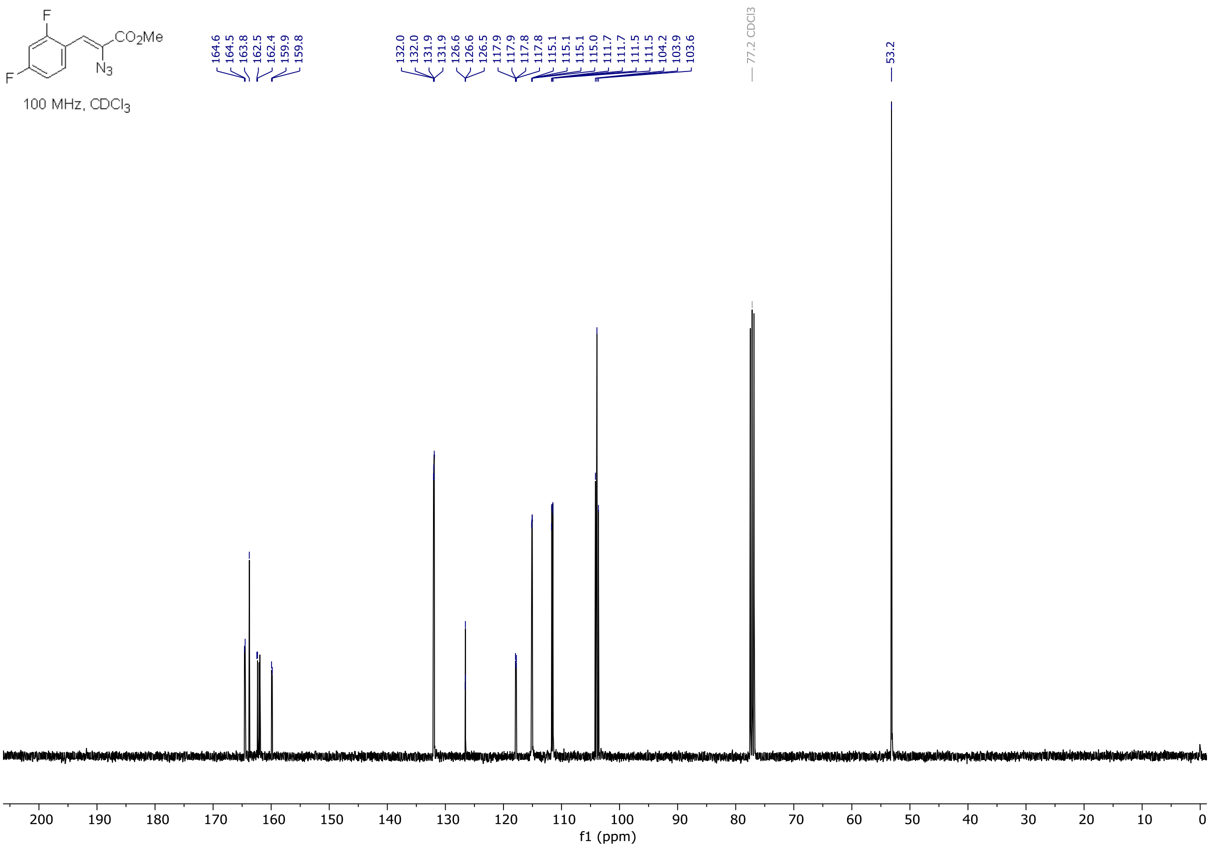

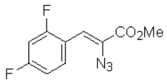

376 MHz, $CDCl_3$

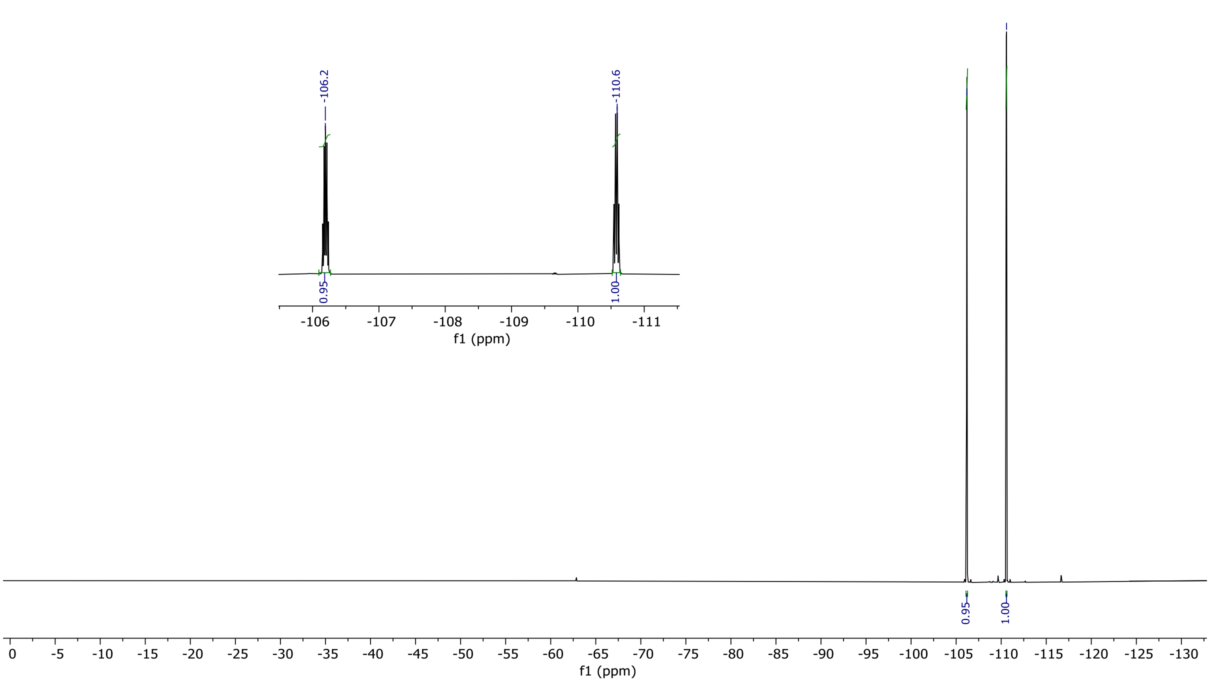

Tert-butyl (Z)-2-azido-3-phenylacrylate (1k)

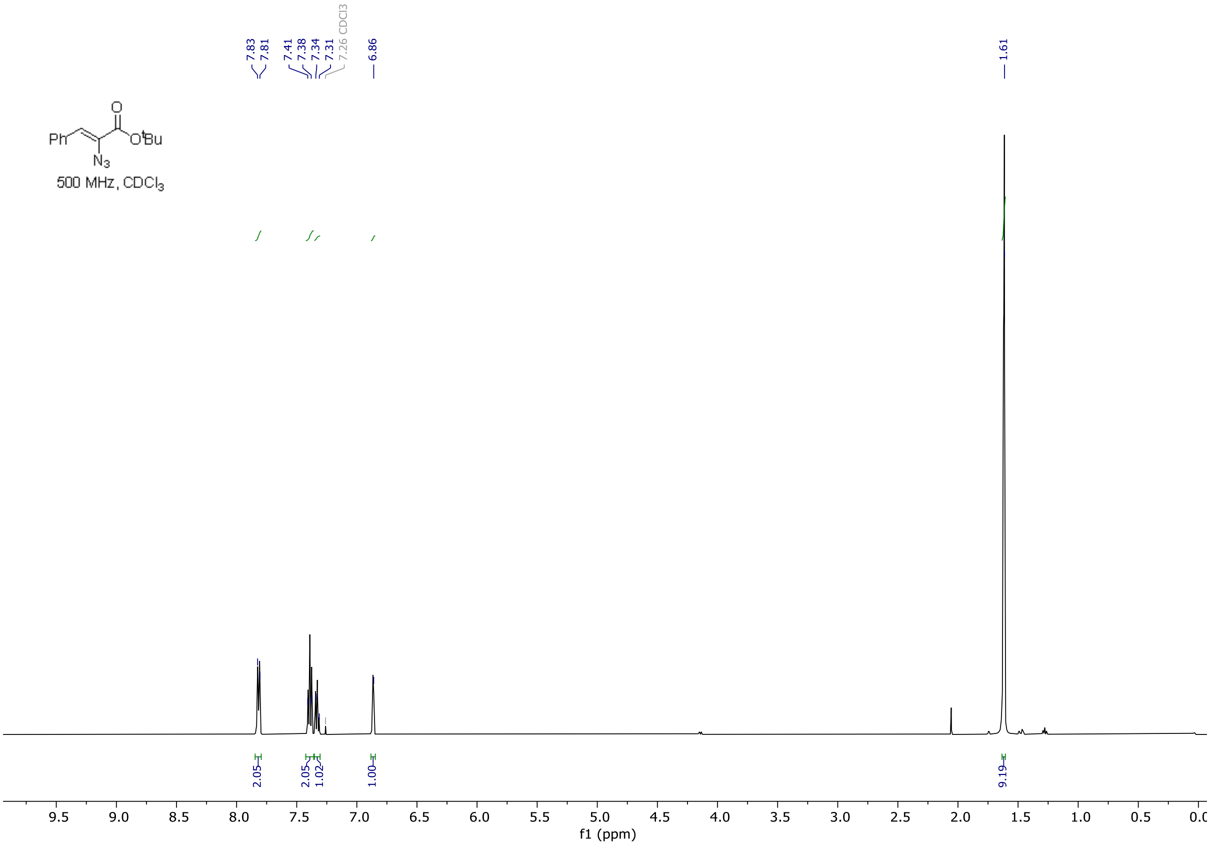

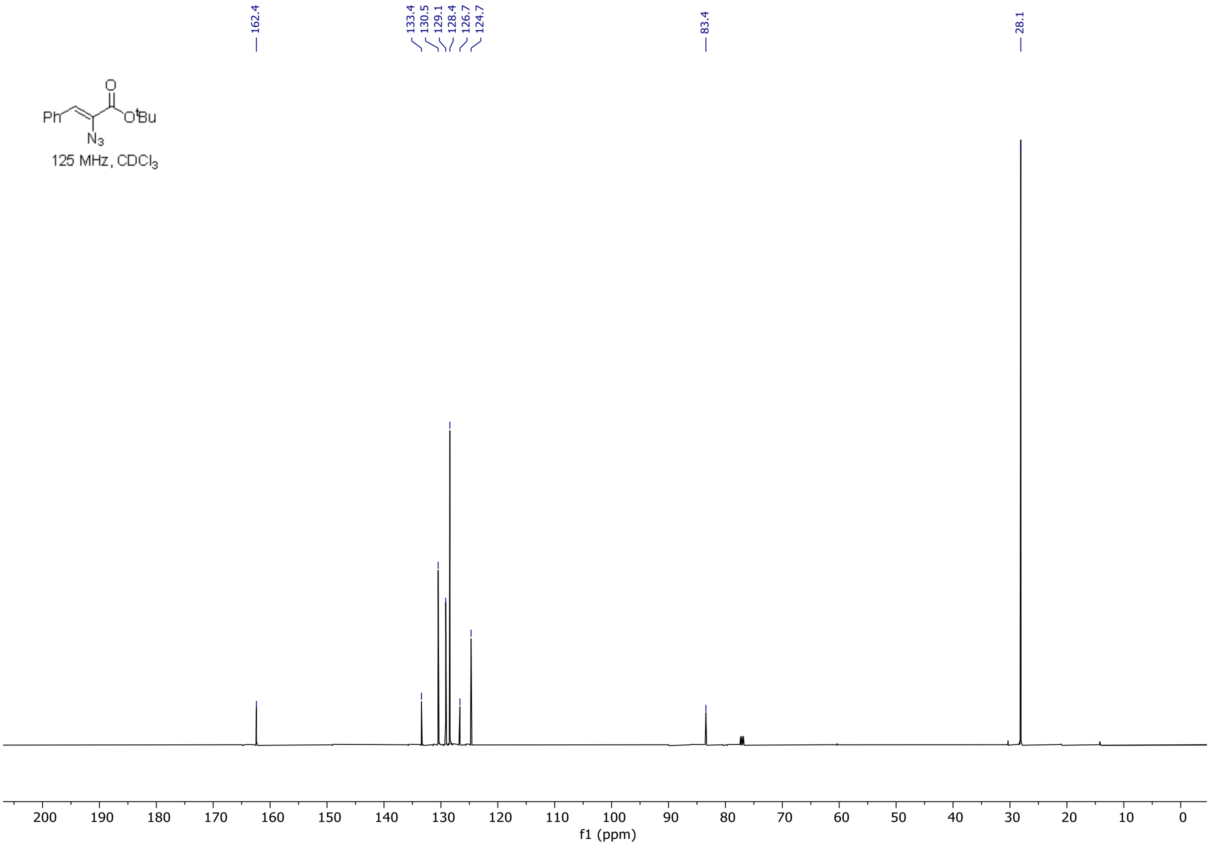

Tert-butyl (Z)-2-azido-3-(4-fluorophenyl)acrylate (1l)

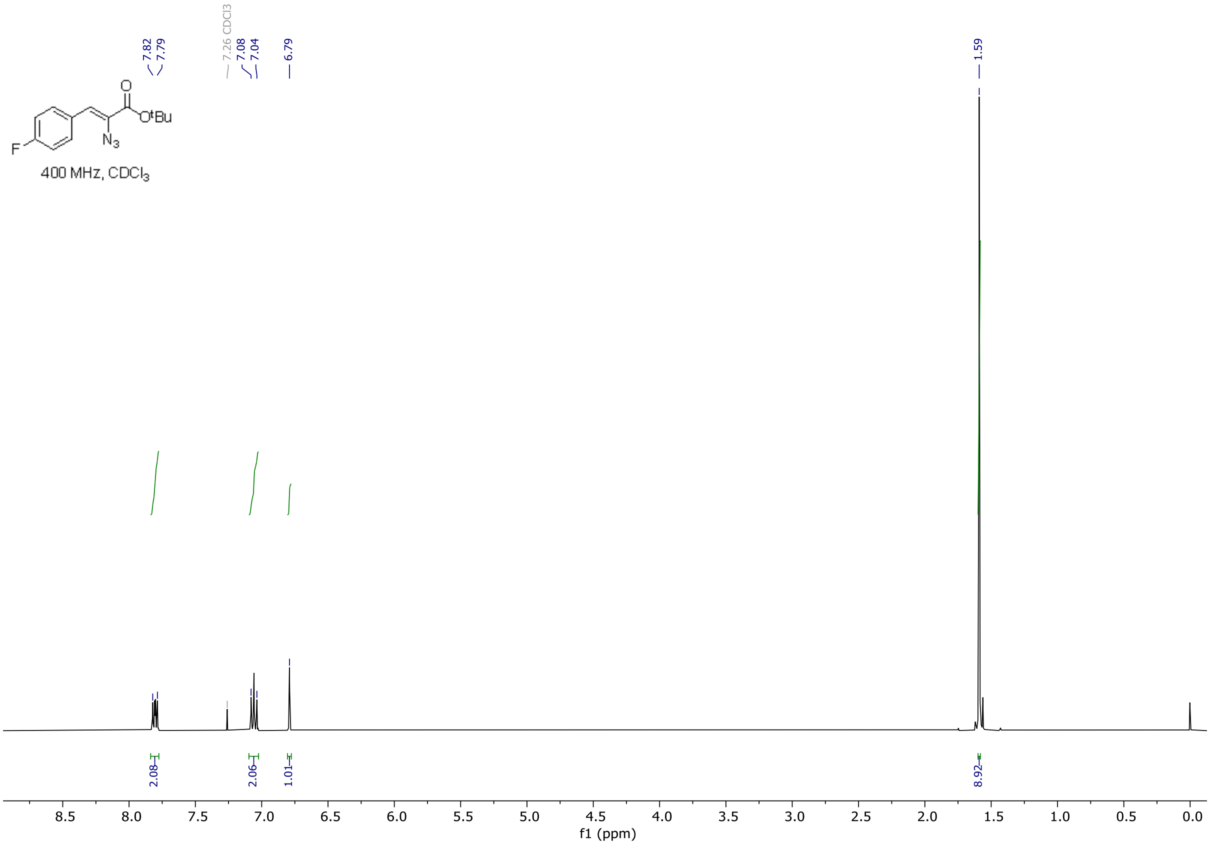

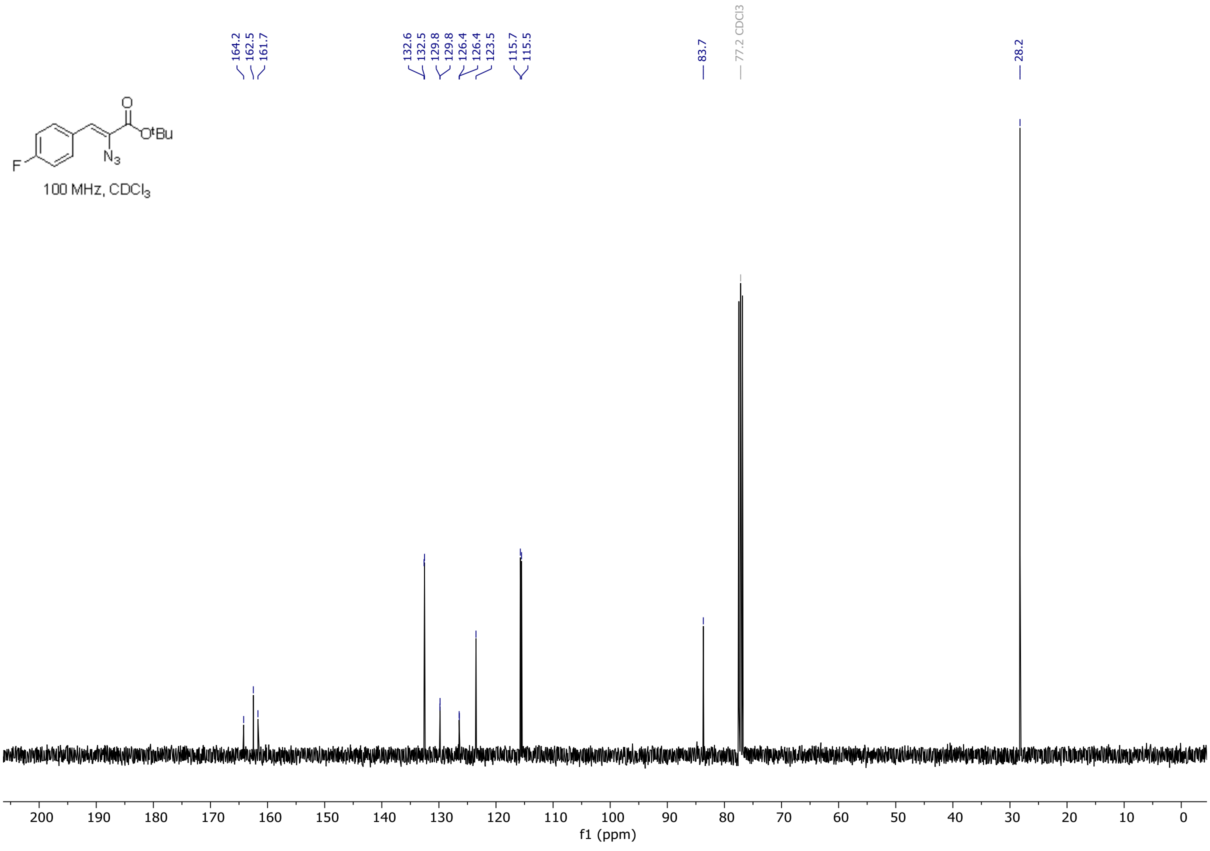

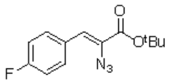

376 MHz, $CDCl_3$

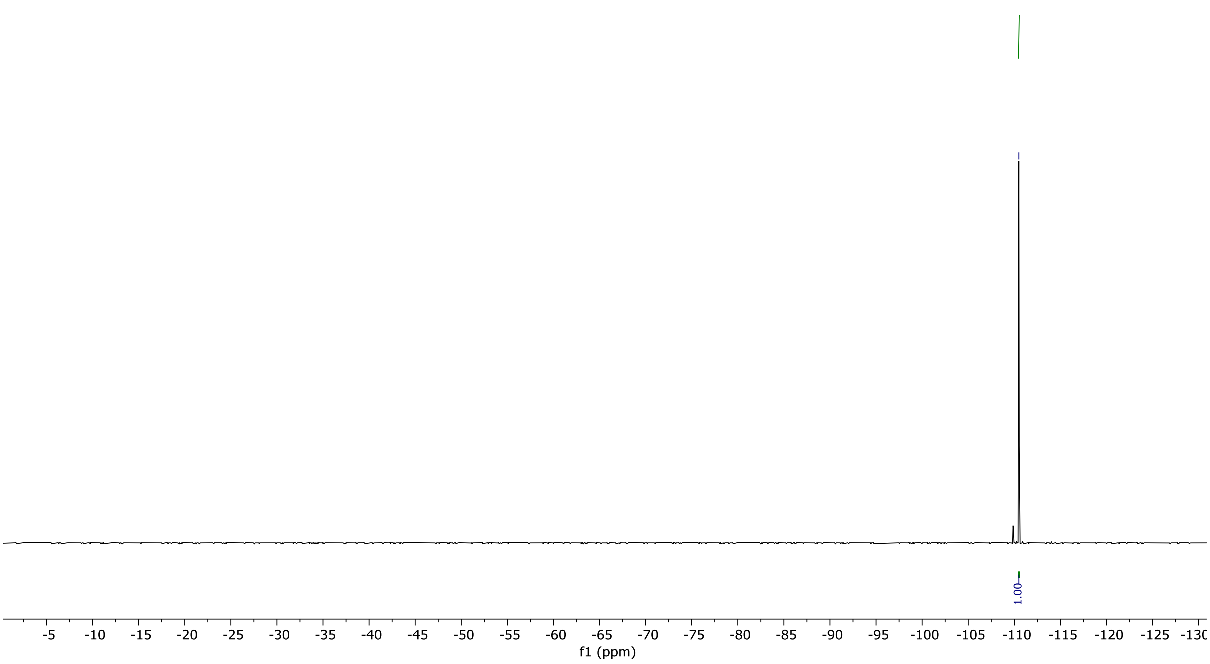

Tert-butyl (Z)-2-azido-3-(4-methoxyphenyl)acrylate (1m)

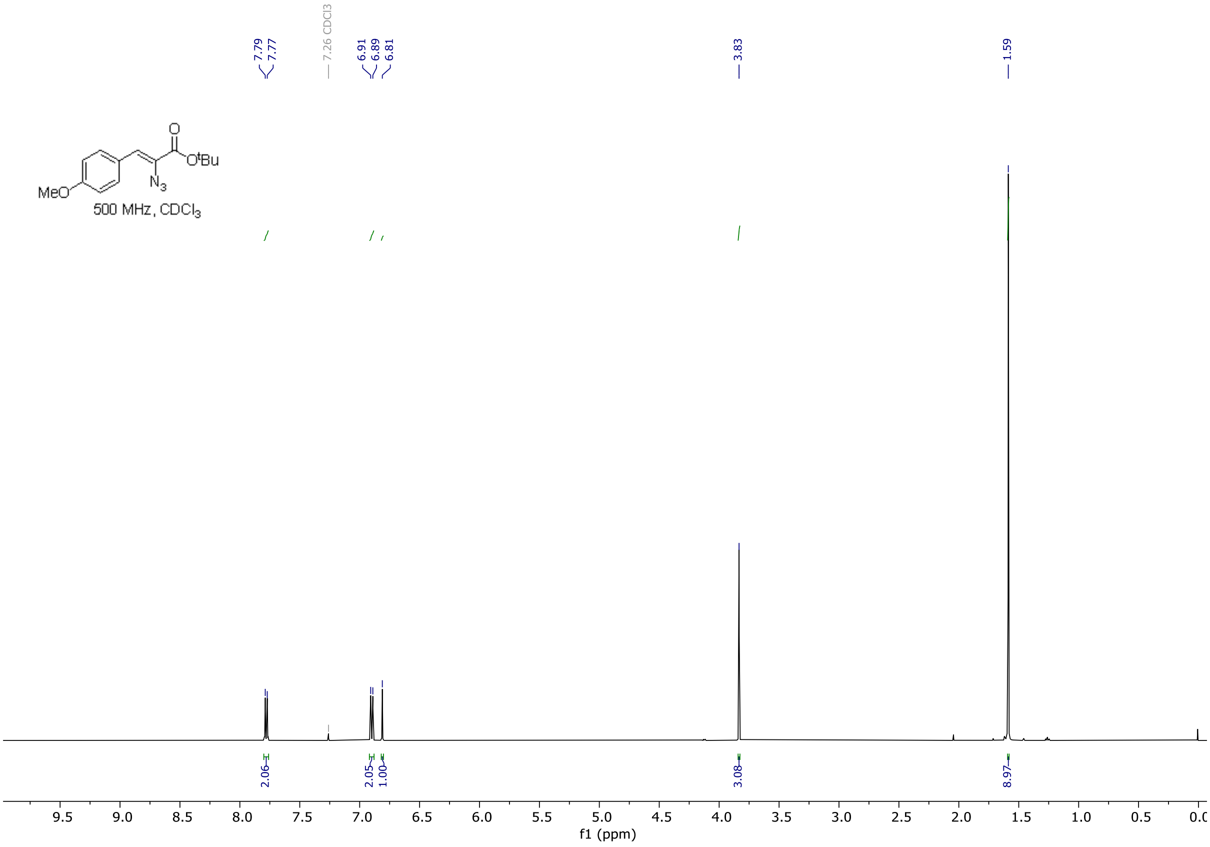

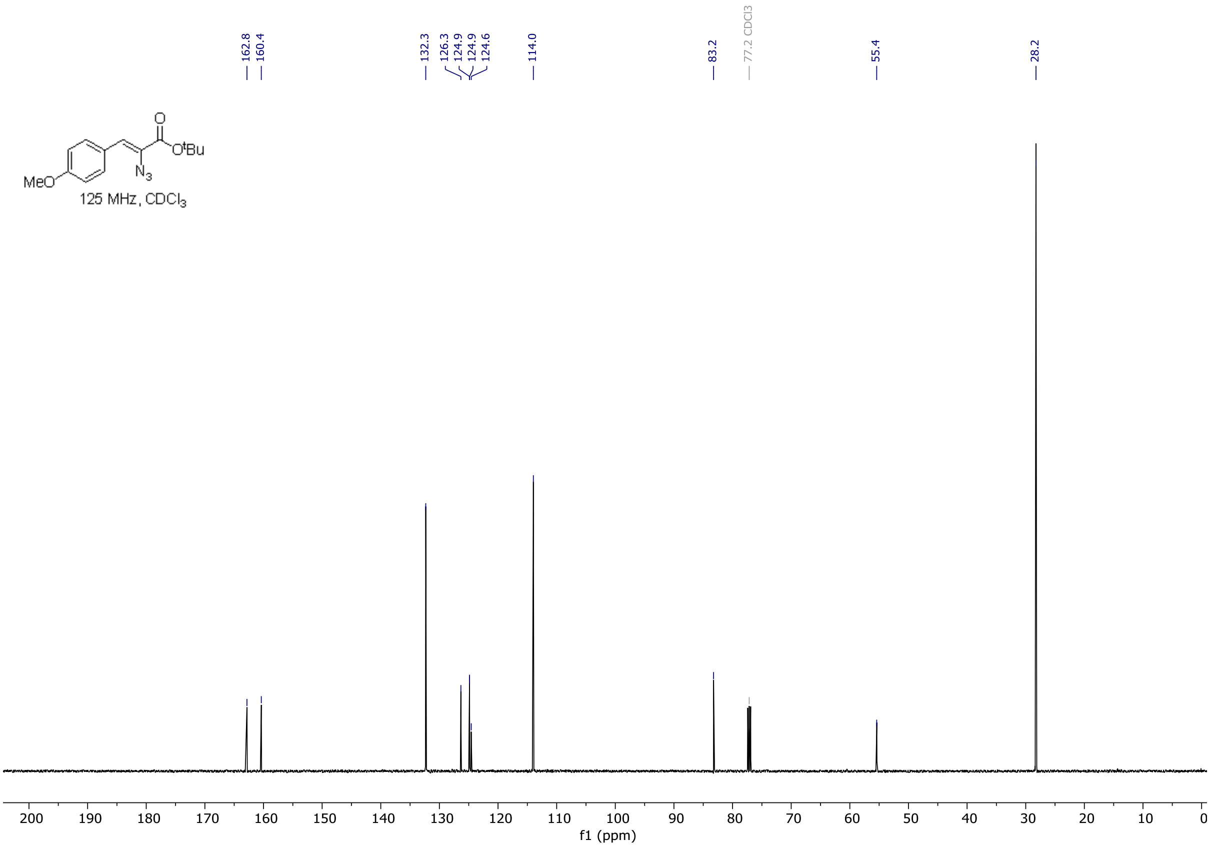

Tert-pentyl (Z)-2-azido-3-(4-fluorophenyl)acrylate (1n)

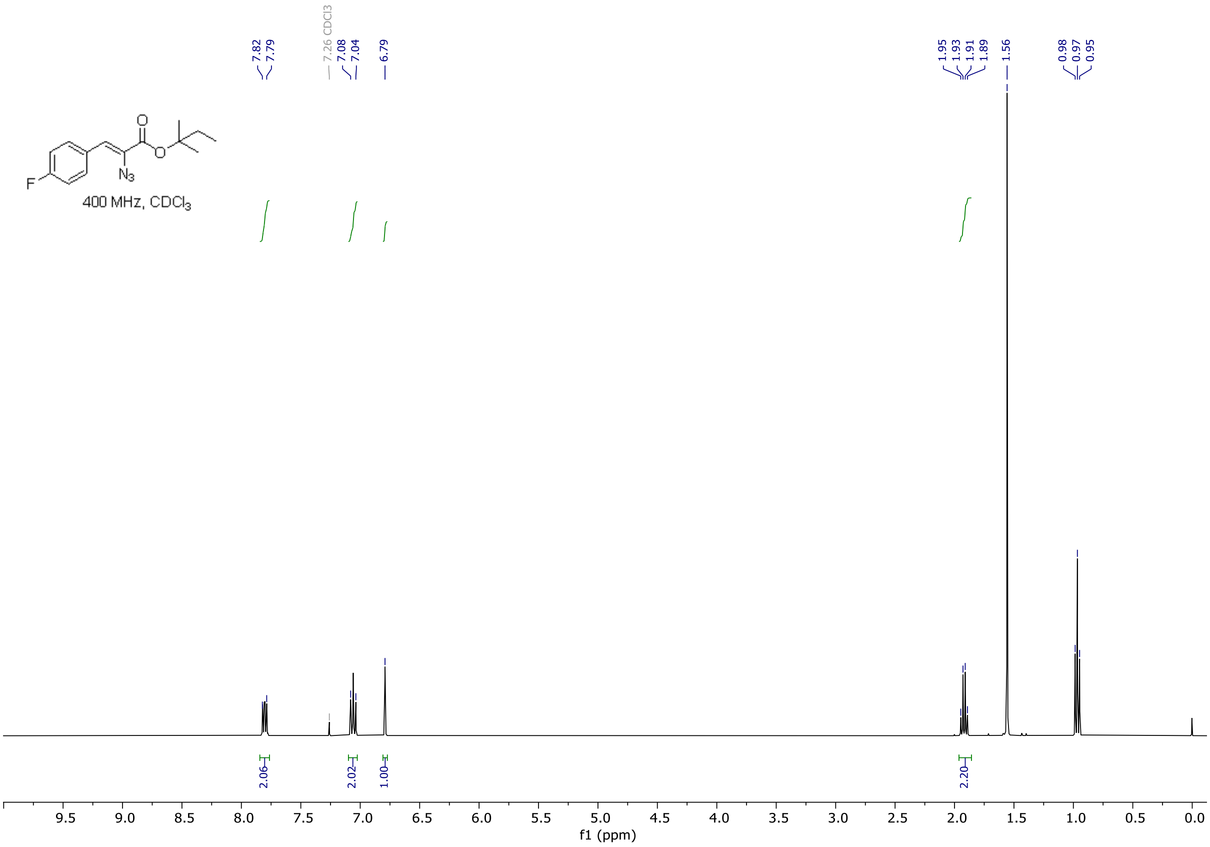

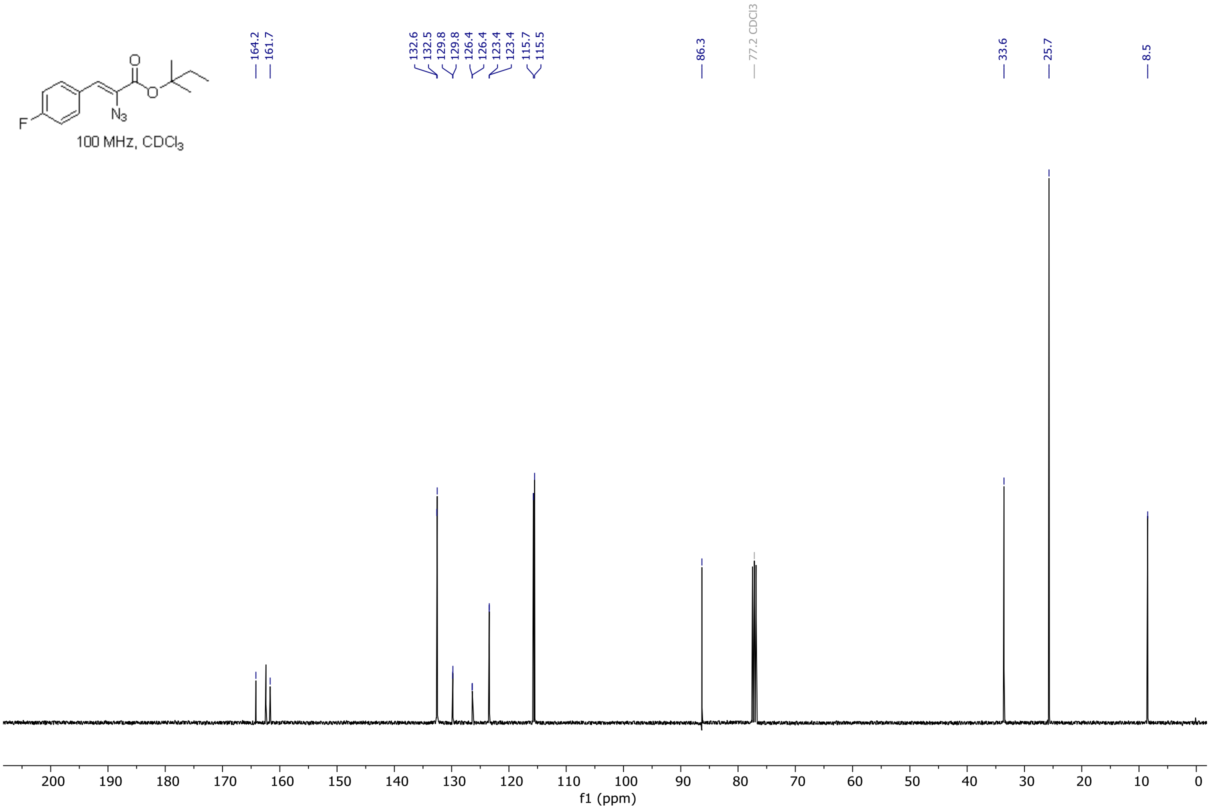

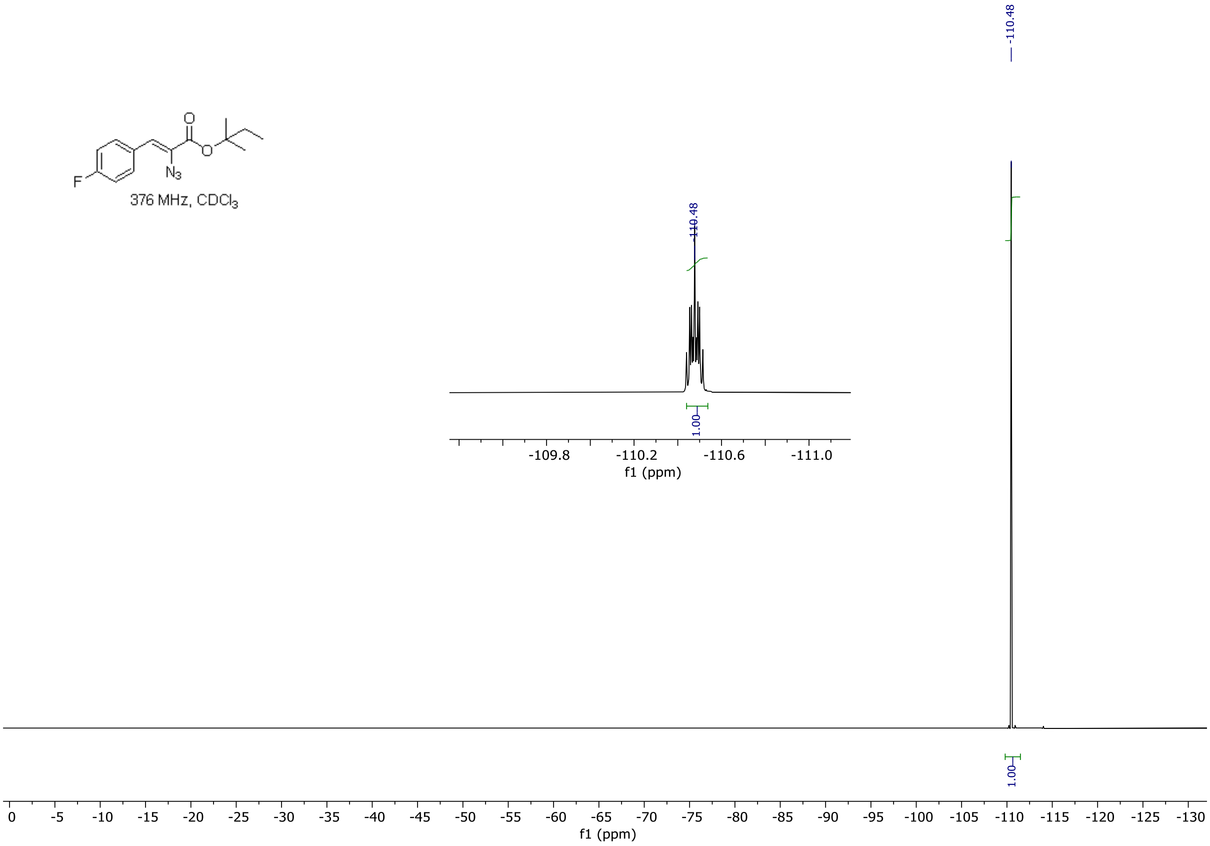
376 MHz, CDCl3
-110.48
1.00
-109.8
-110.2
-110.6
-111.0
f1 (ppm)
0
-5
-10
-15
-20
-25
-30
-35
-40
-45
-50
-55
-60
-65
-70
-75
-80
-85
-90
-95
-100
-105
-110
-115
-120
-125
-130

Methyl 2-phenyl-2H-azirine-3-carboxylate (2a) Crude ^{1}H-NMR and ^{13}C-NMR

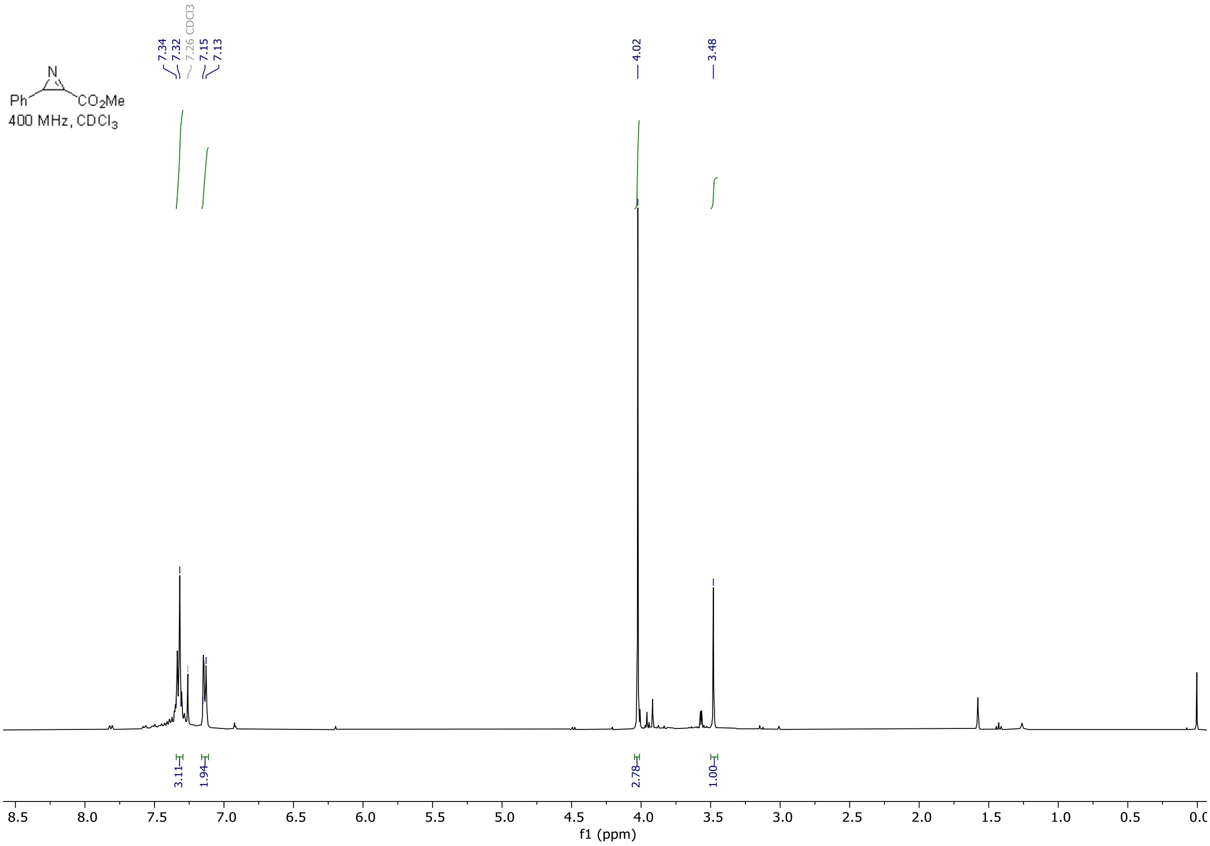

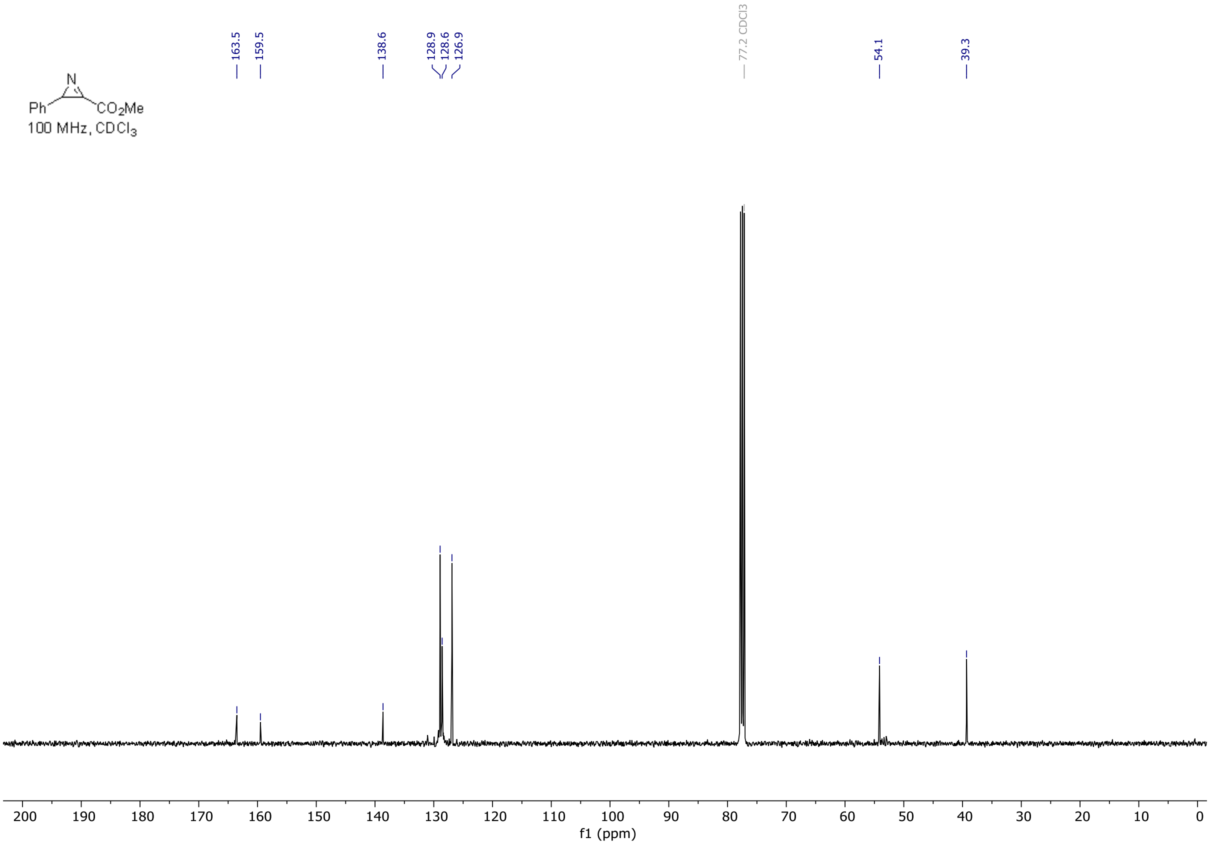

Methyl 2-(4-fluorophenyl)-2H-azirine-3-carboxylate (2b) Crude ^{1}H-NMR and ^{13}C-NMR

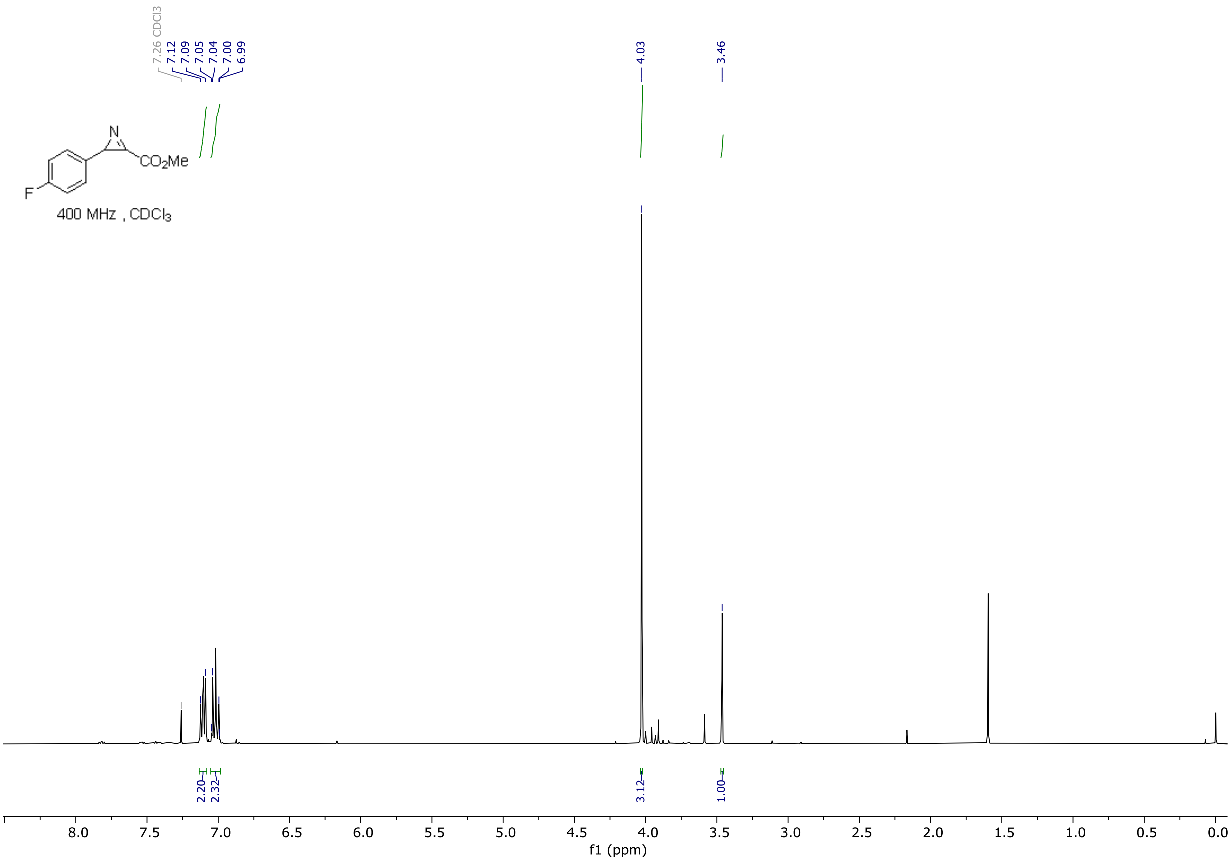

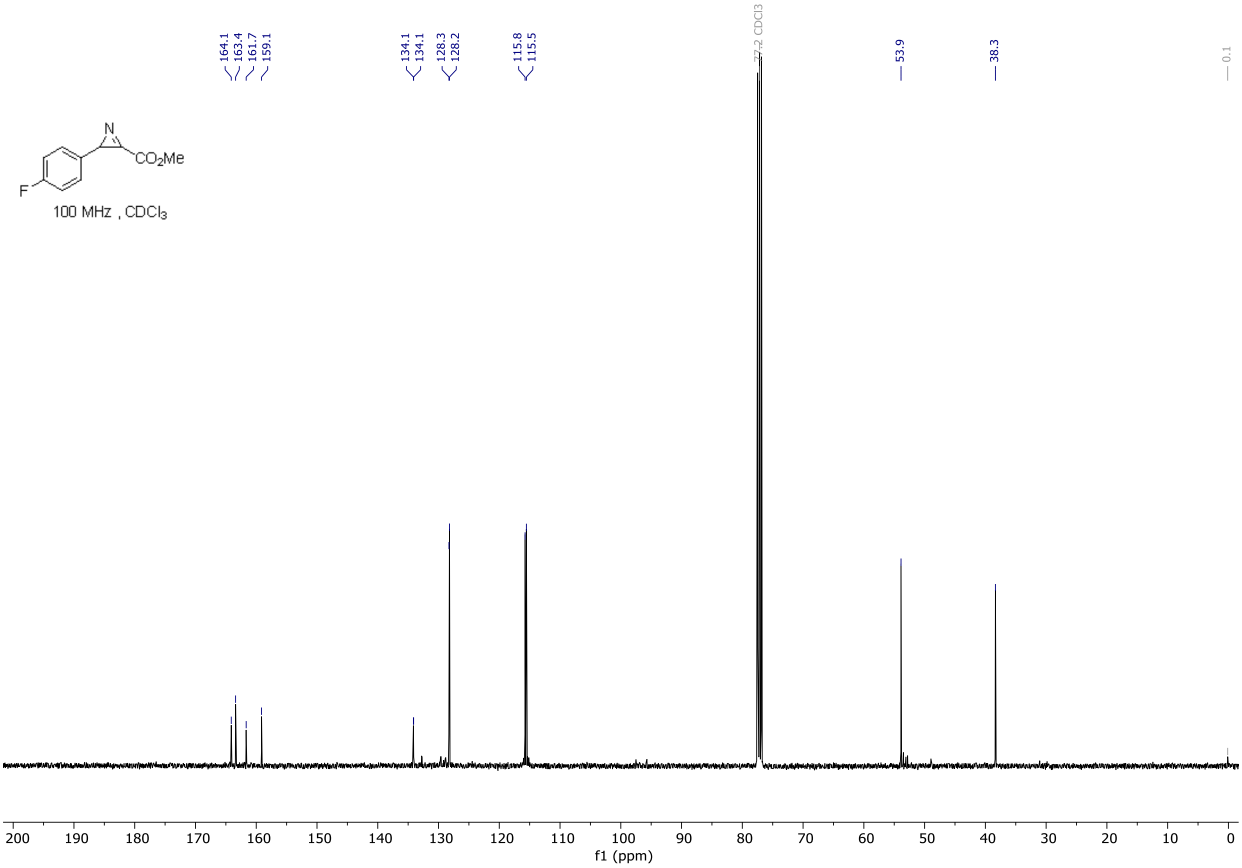
164.1
163.4
161.7
159.1
134.1
134.1
128.3
128.2
115.8
115.5
77.2 CDCl3
53.9
38.3
0.1
N
CO_2Me
F
100 MHz , $CDCl_3$
f1 (ppm)

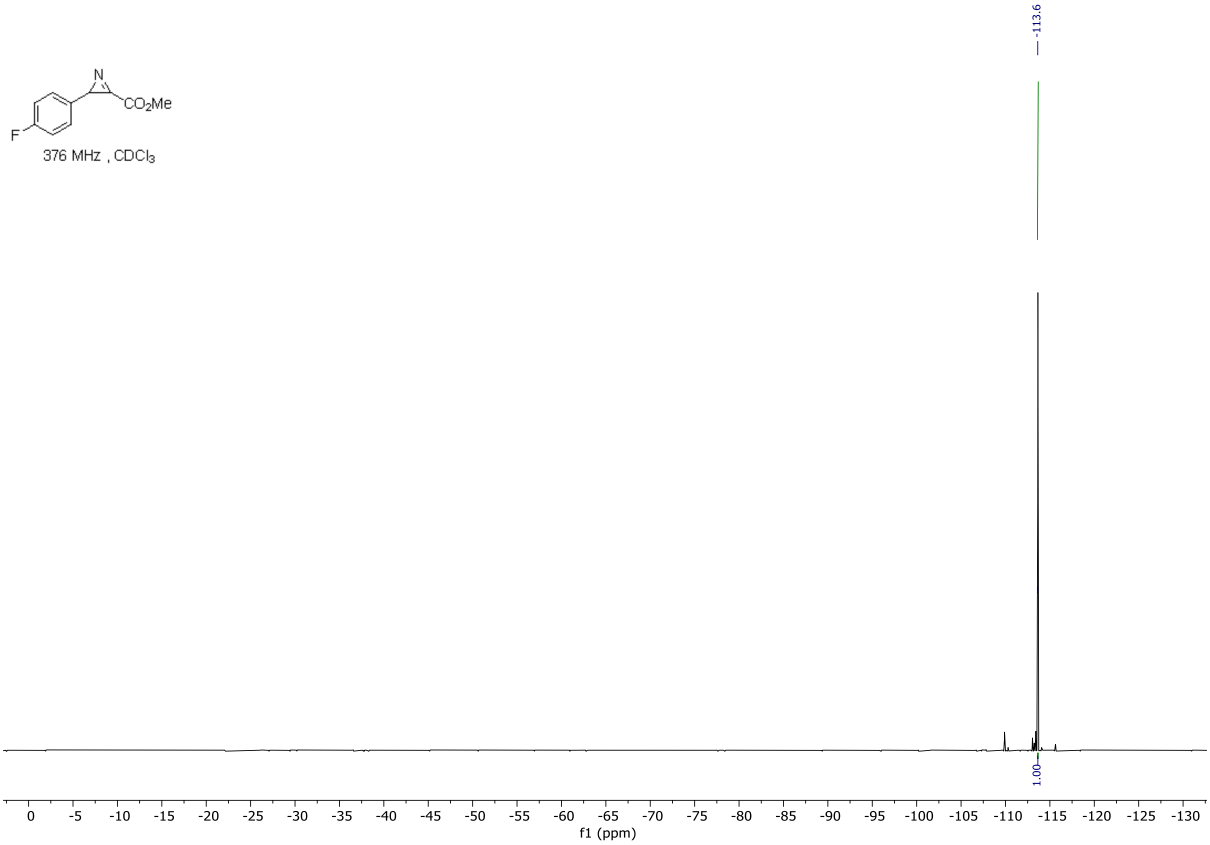
-113.6
N
CO_2Me
F
376 MHz , $CDCl_3$
1.00
f1 (ppm)

Methyl 2-(4-bromophenyl)-2H-azirine-3-carboxylate (2c)

Crude ^{1}H-NMR and ^{13}C-NMR

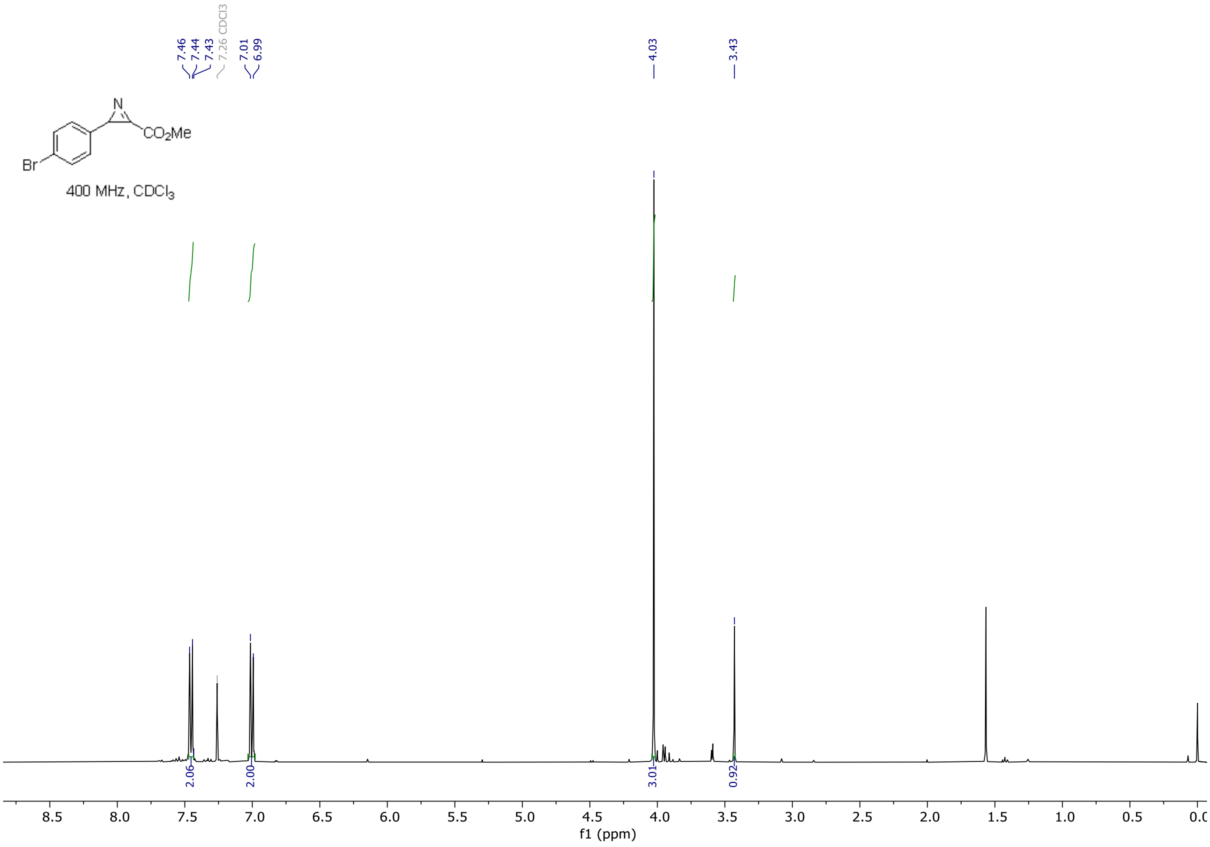

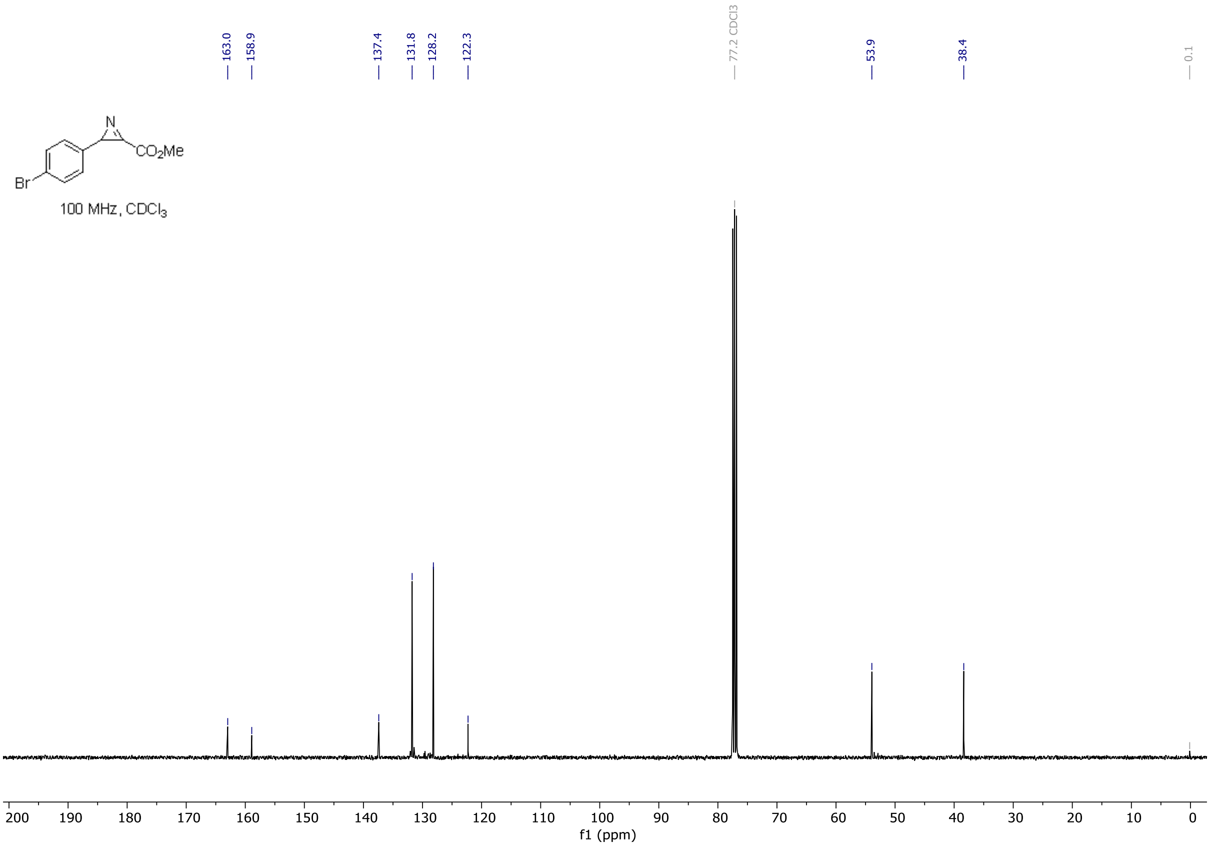

Methyl 2-(p-tolyl)-2H-azirine-3-carboxylate (2d)

Crude ^{1}H-NMR and ^{13}C-NMR

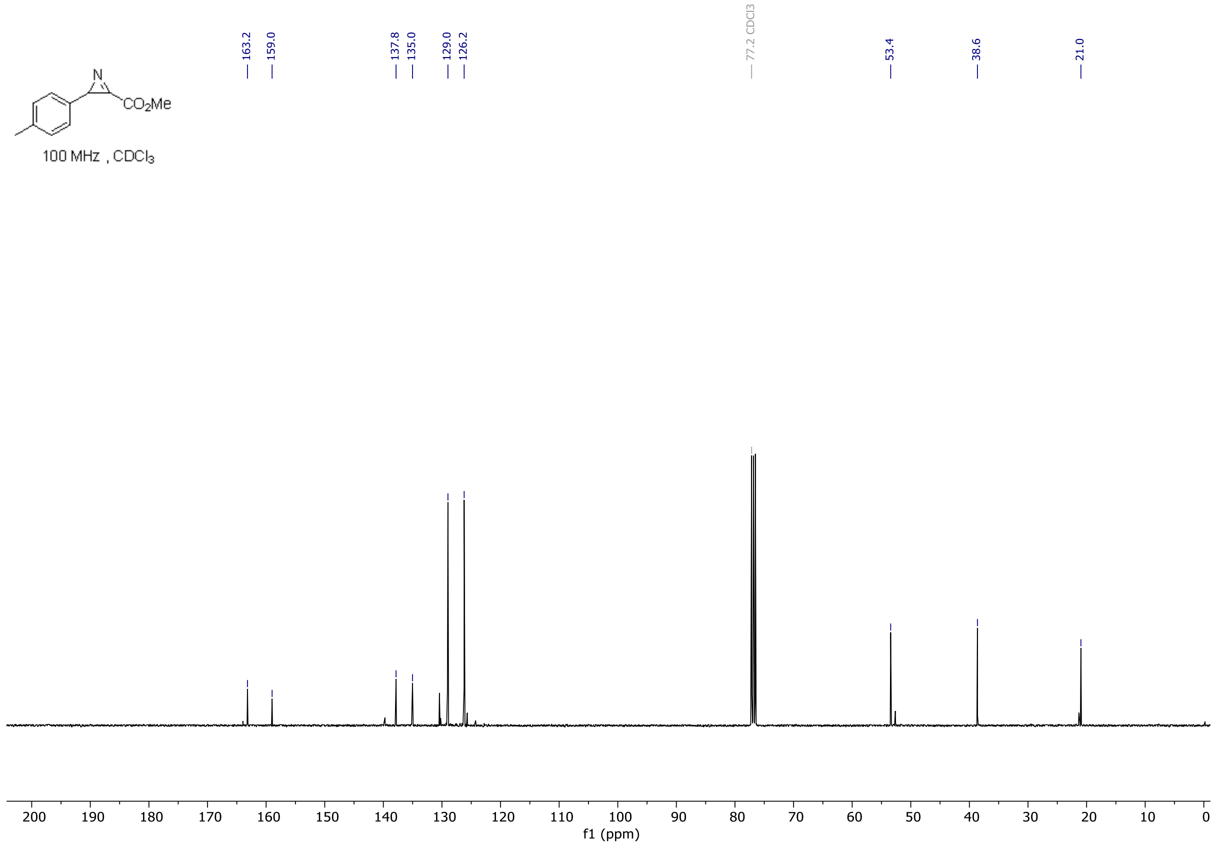

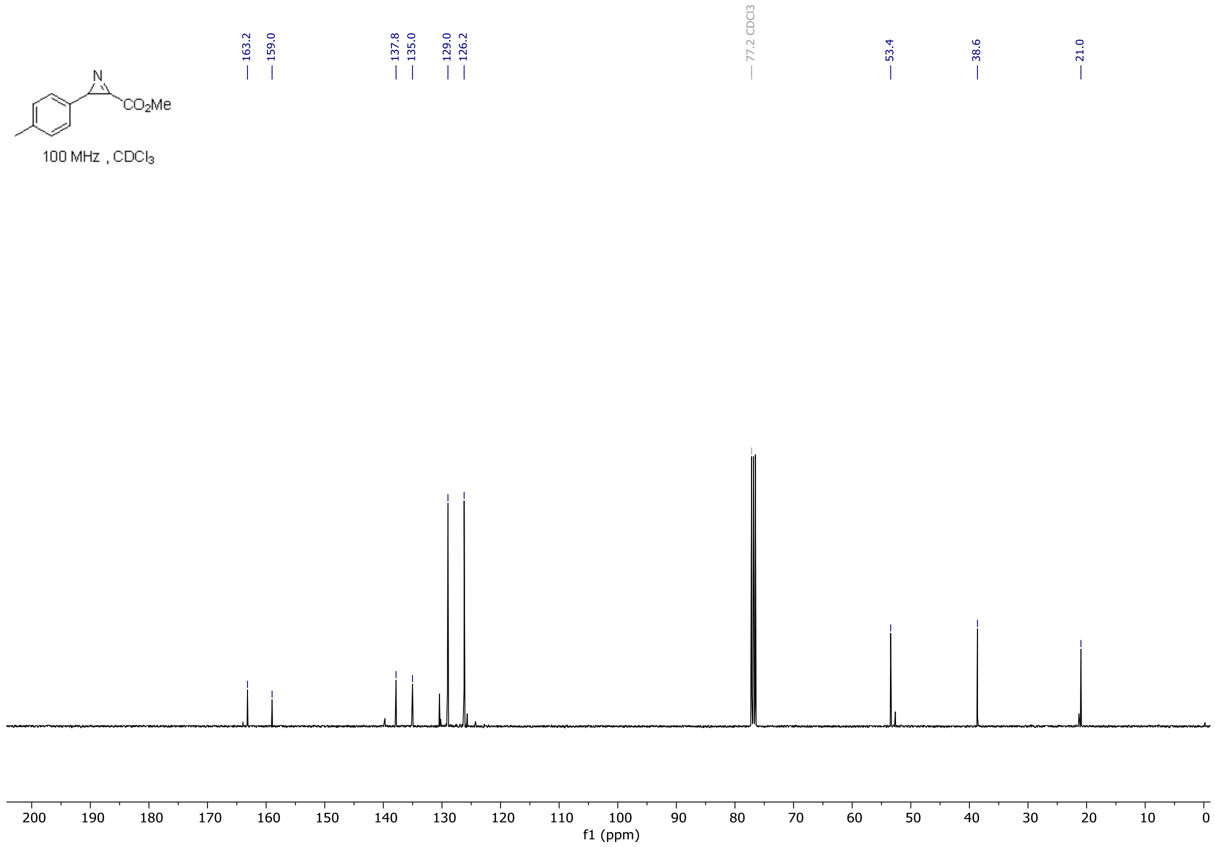

Methyl 2-(3,4-dimethylphenyl)-2H-azirine-3-carboxylate (2e)

Crude ^{1}H-NMR and ^{13}C-NMR

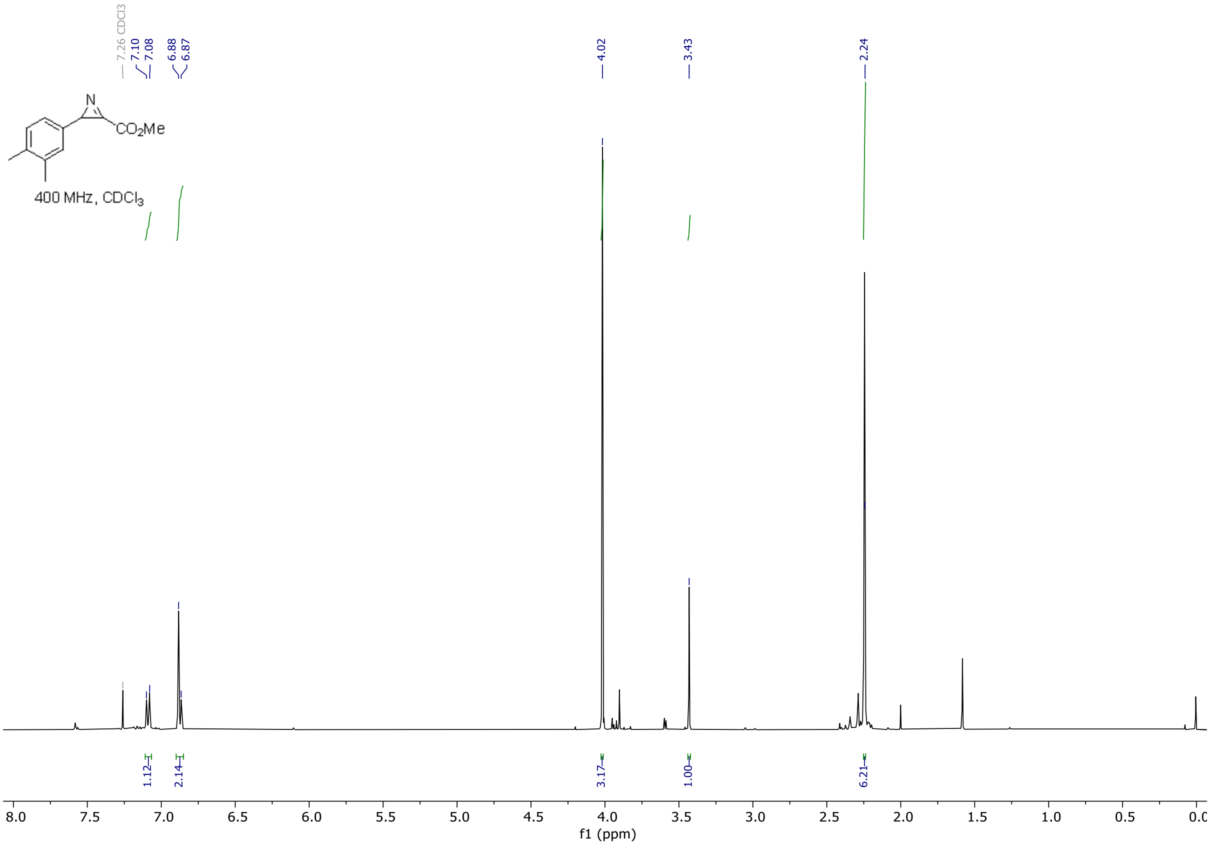

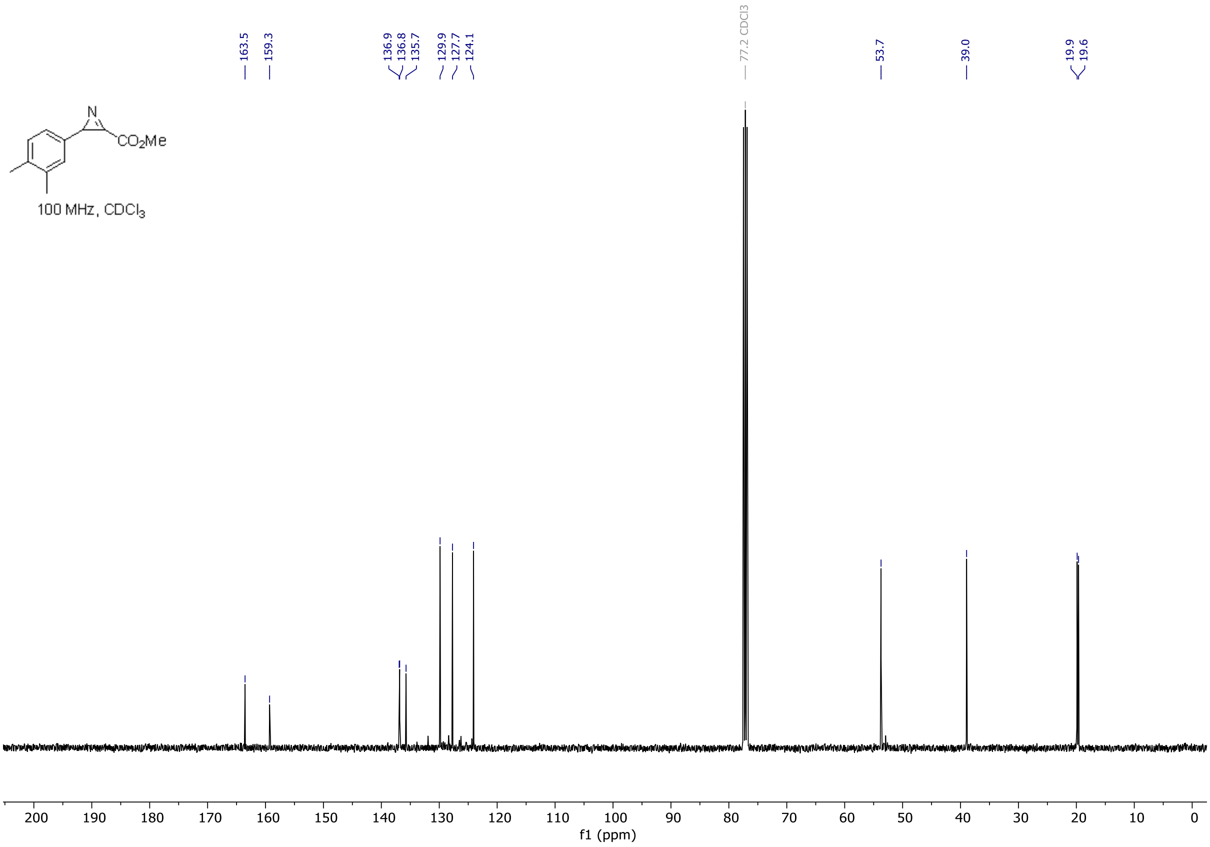

Methyl 2-(o-tolyl)-2H-azirine-3-carboxylate (2f)

Crude ^{1}H-NMR and ^{13}C-NMR

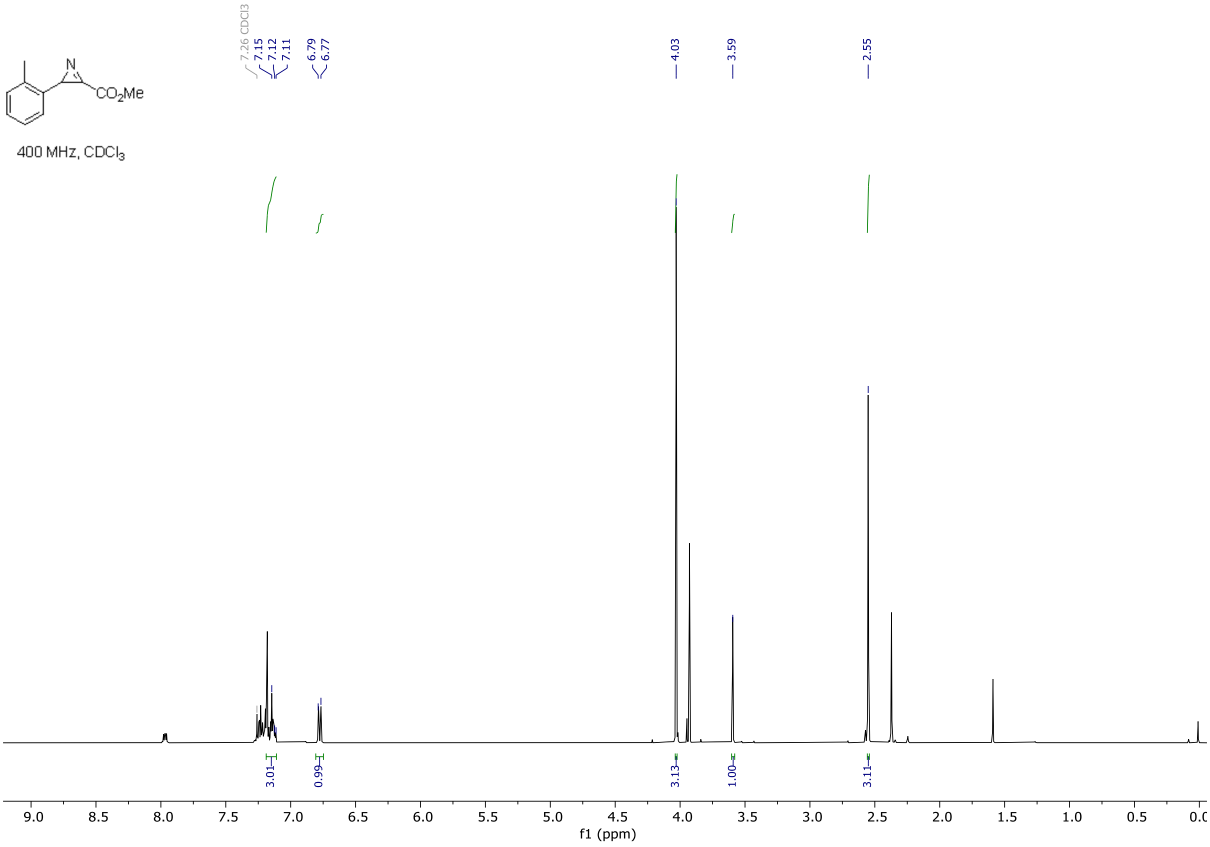

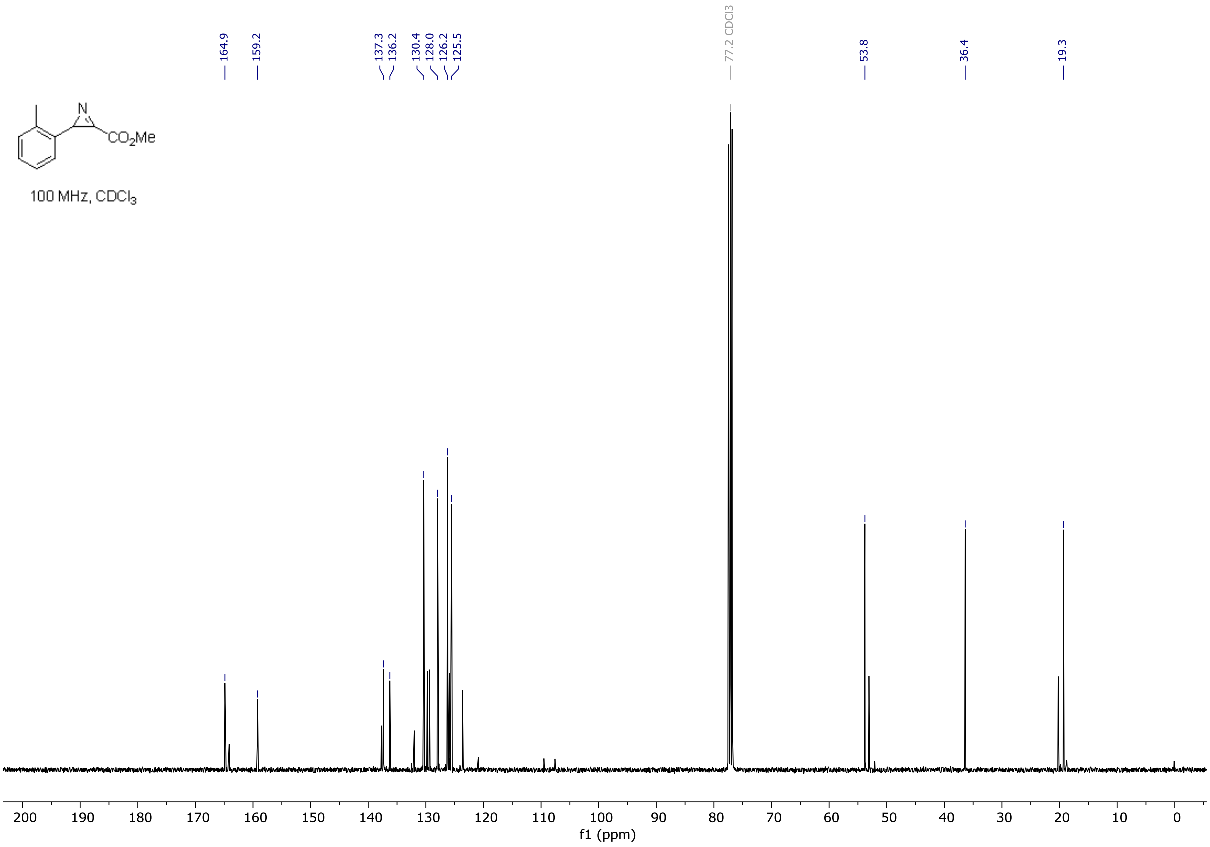

Methyl 2-cyclohexyl-2H-azirine-3-carboxylate (2h)

Crude ^{1}H-NMR and ^{13}C-NMR

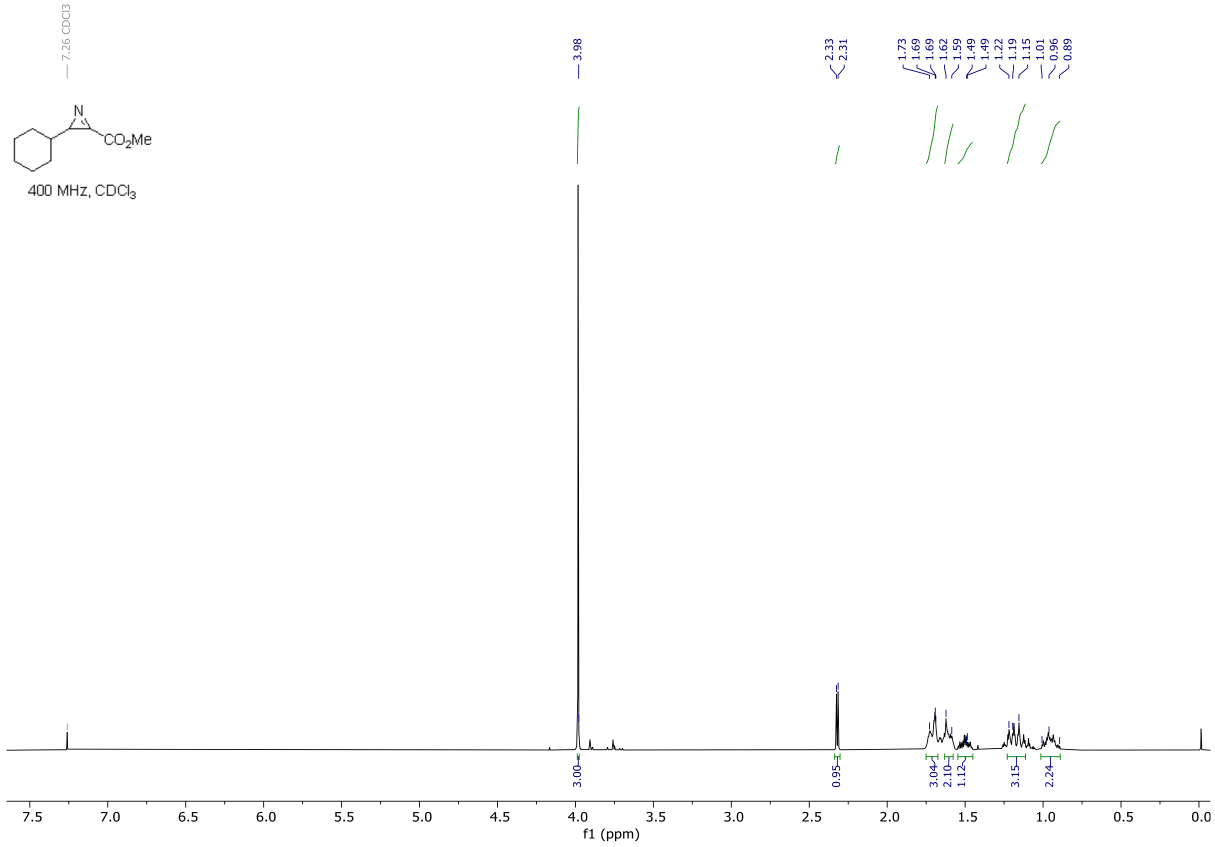

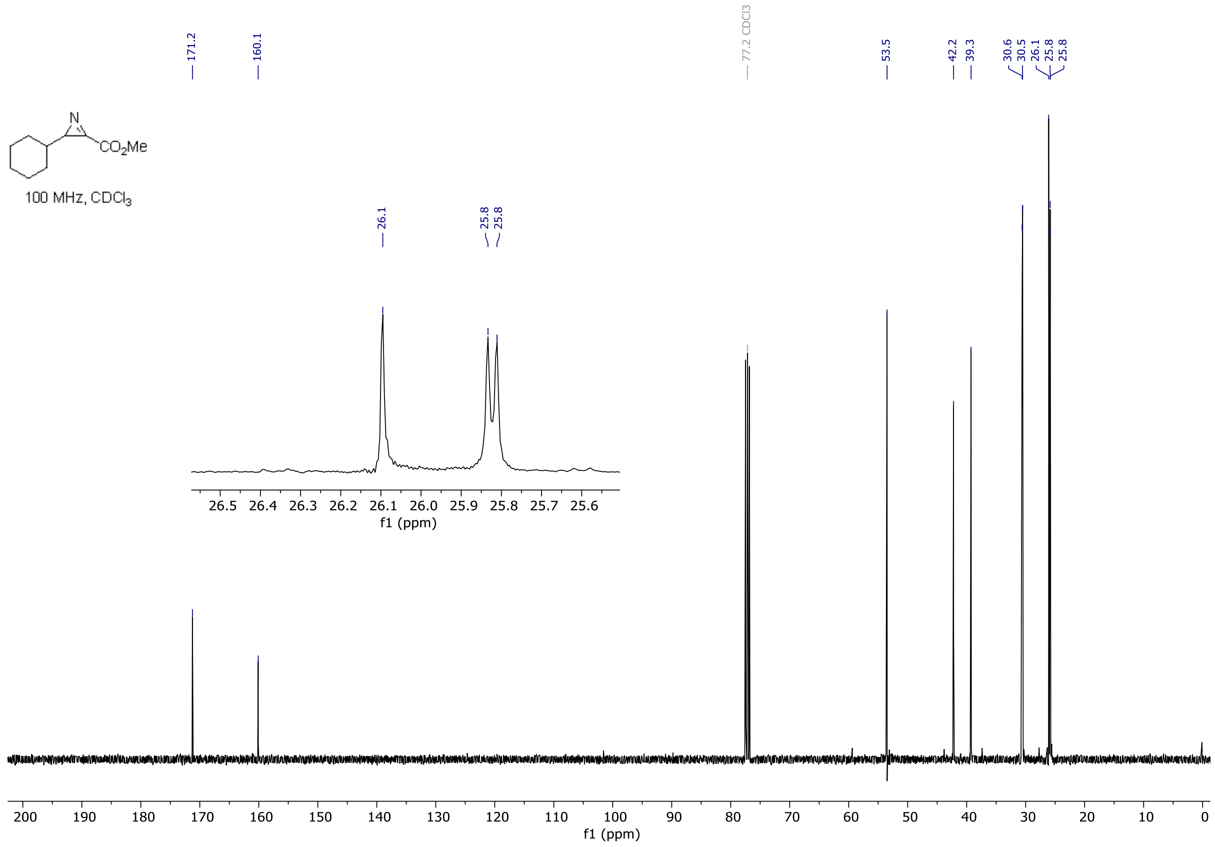

(+/-)Dimethyl(2S,5S,6R)-2,6-diphenyl-1,3-diazabicyclo[3.1.0]hex-3-ene-4,5-dicarboxylate (3a)

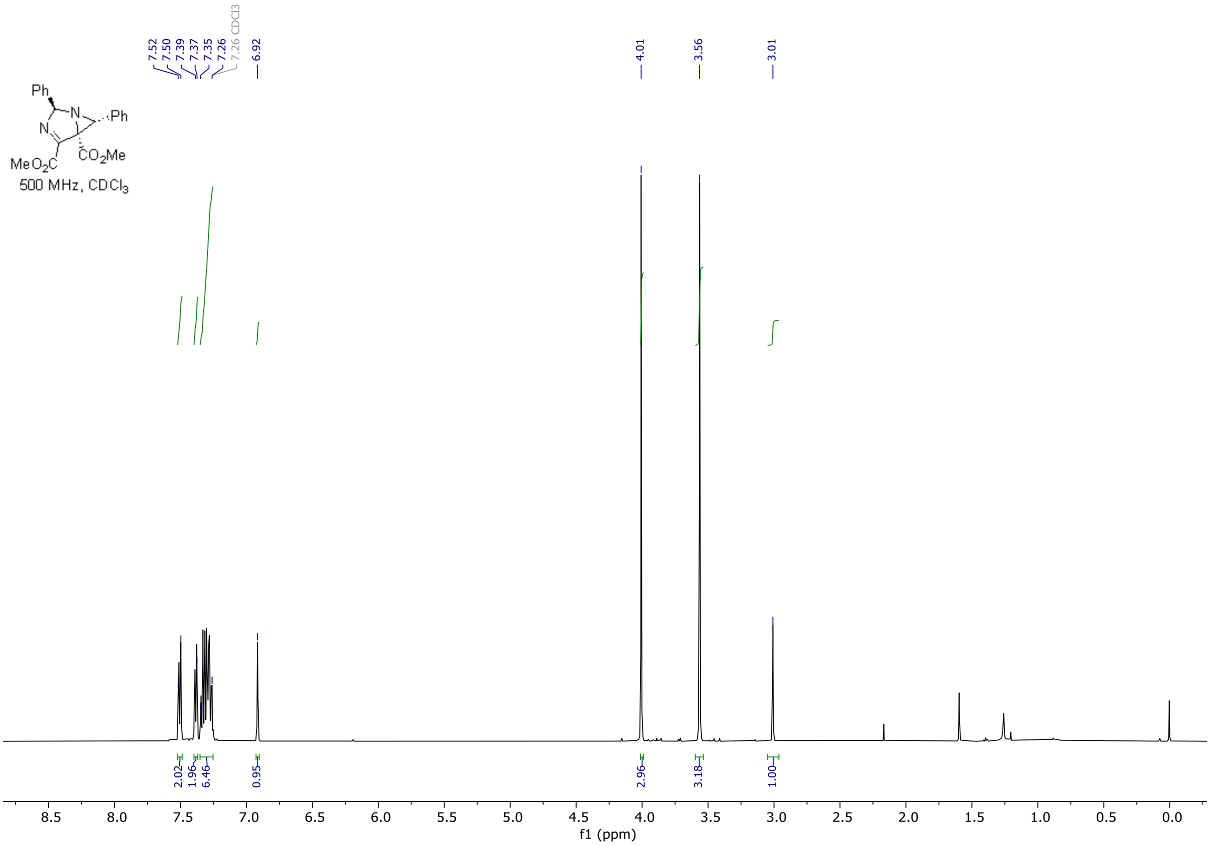

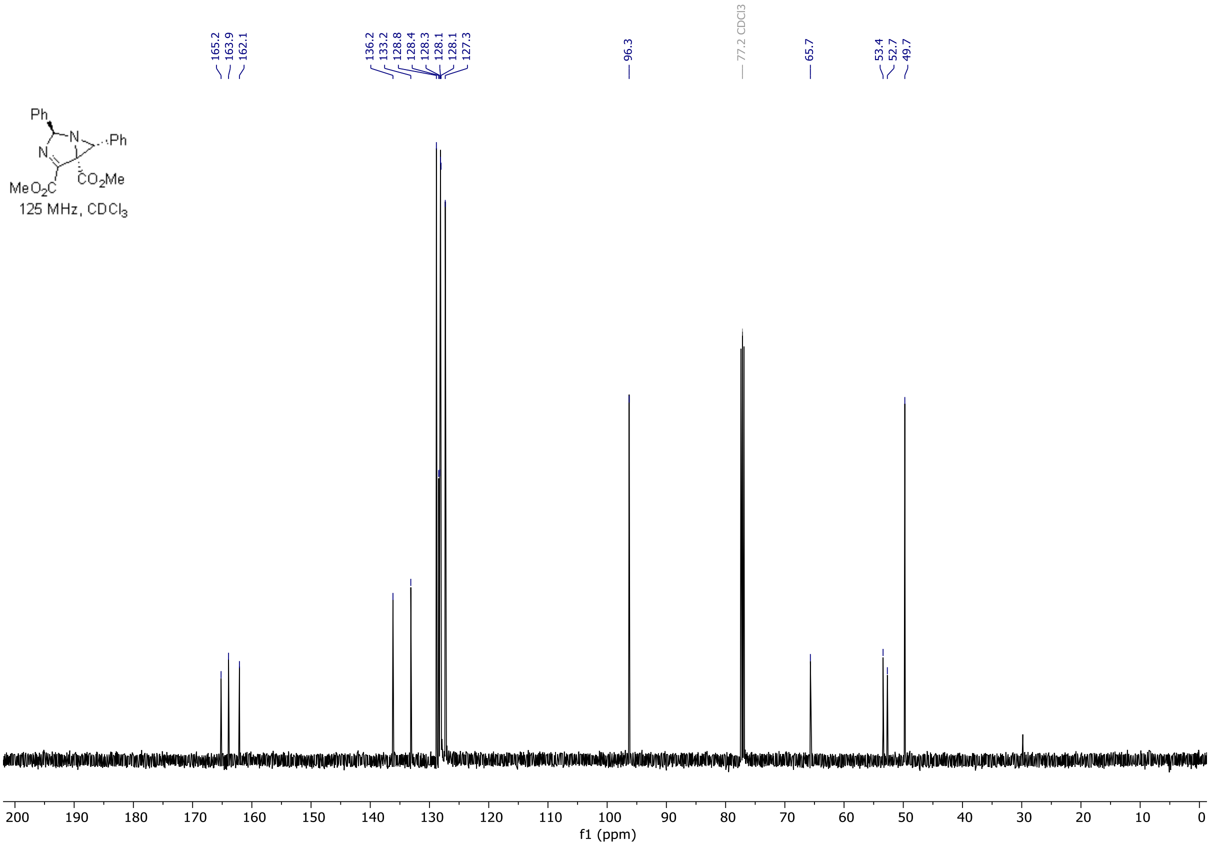

(+/-)Dimethyl(2R,5S,6R)-2,6-diphenyl-1,3-diazabicyclo[3.1.0]hex-3-ene-4,5-dicarboxylate (3a')

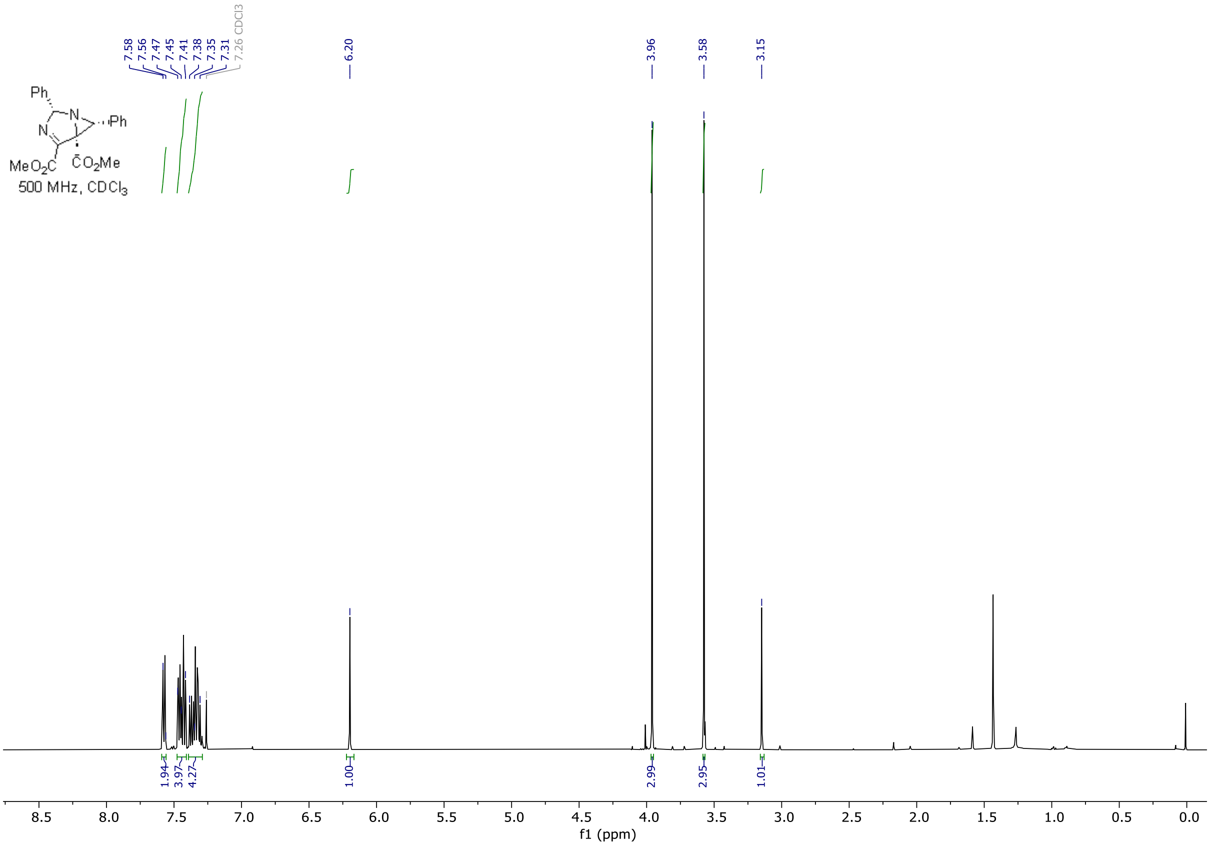

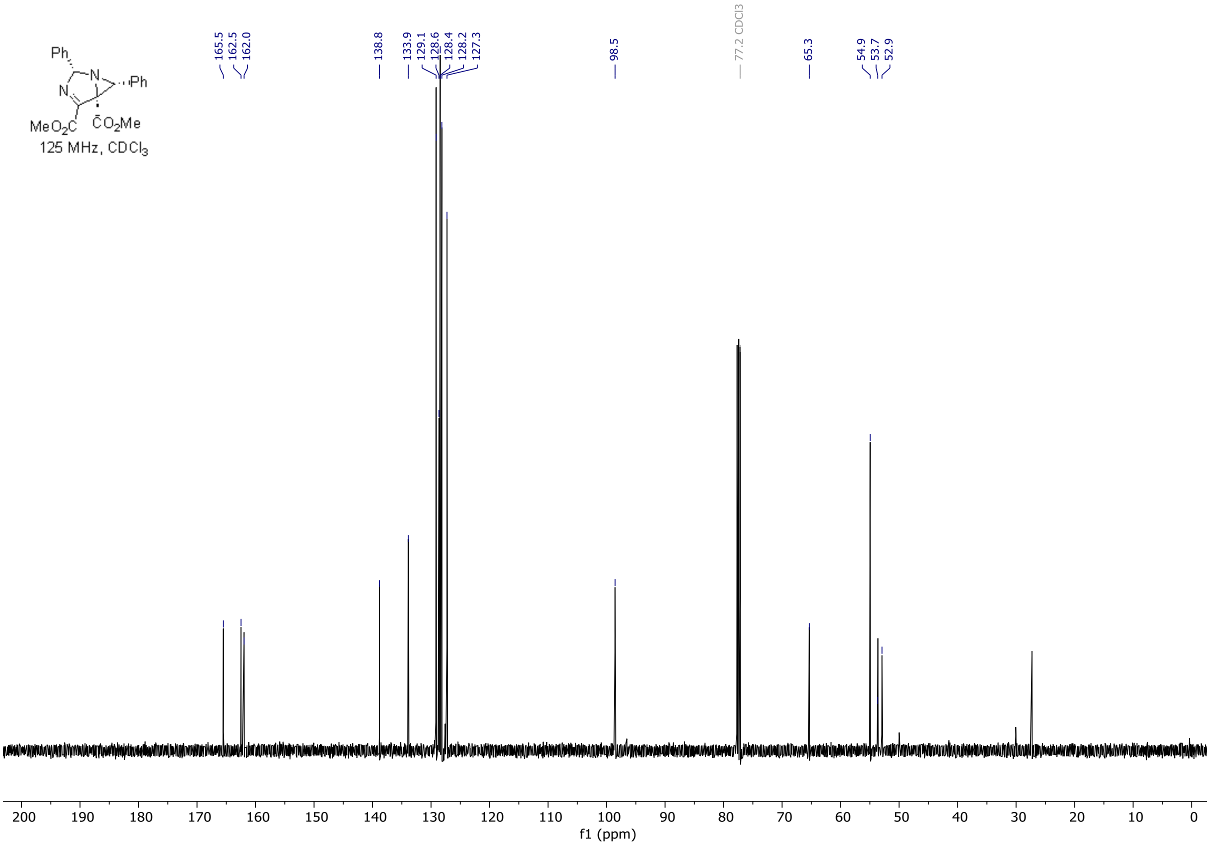

(+/-)Dimethyl(2S,5S,6R)-2,6-bis(4-fluorophenyl)-1,3-diazabicyclo[3.1.0]hex-3-ene-4,5-dicarboxylate (3b)

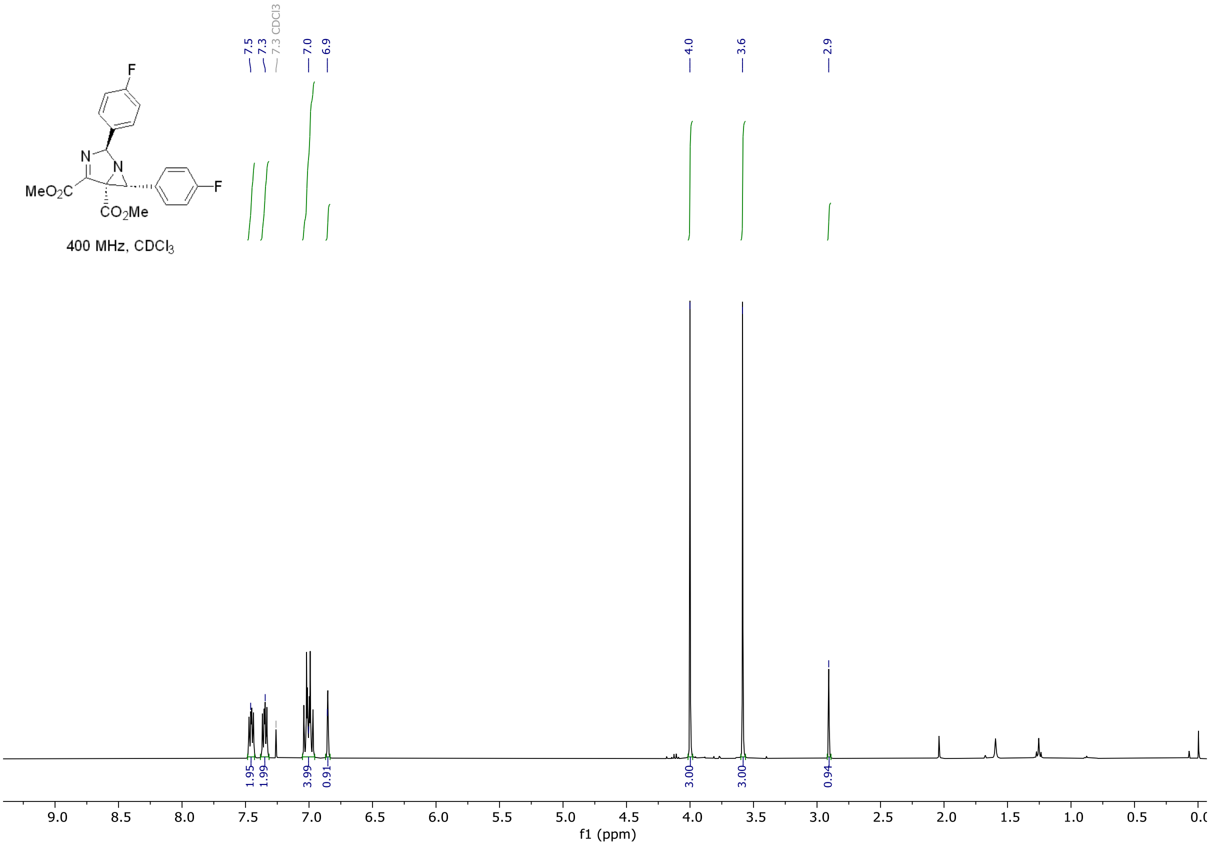

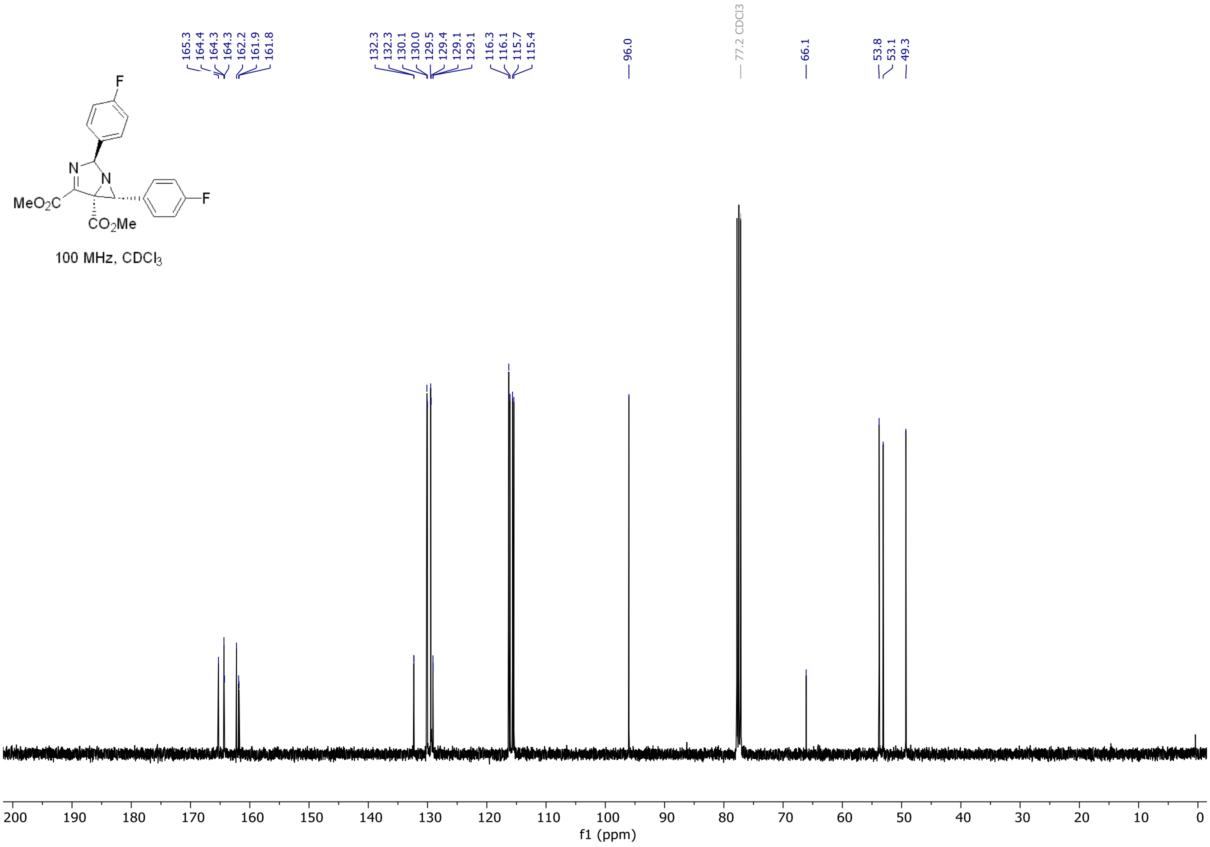

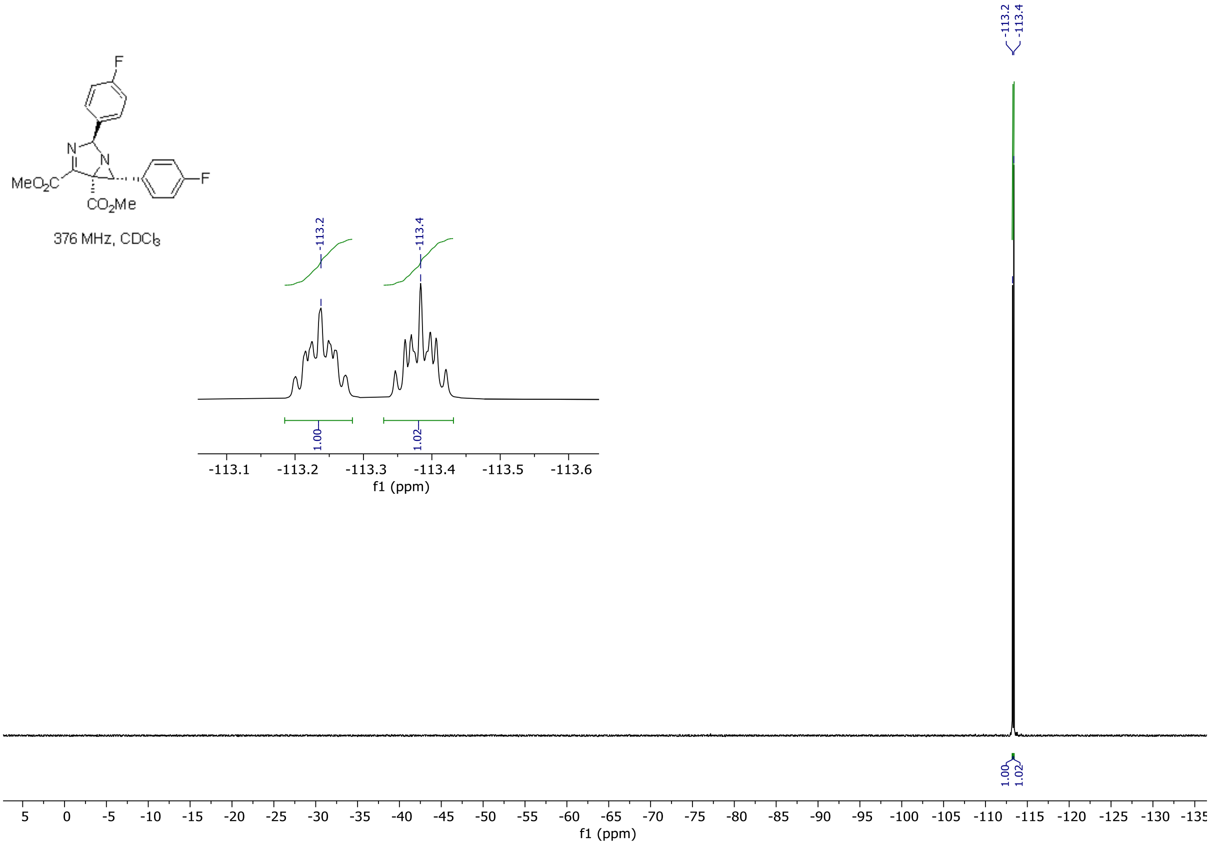

(+/-)Dimethyl(2R,5S,6R)-2,6-bis(4-fluorophenyl)-1,3-diazabicyclo[3.1.0]hex-3-ene-4,5-dicarboxylate (3b')

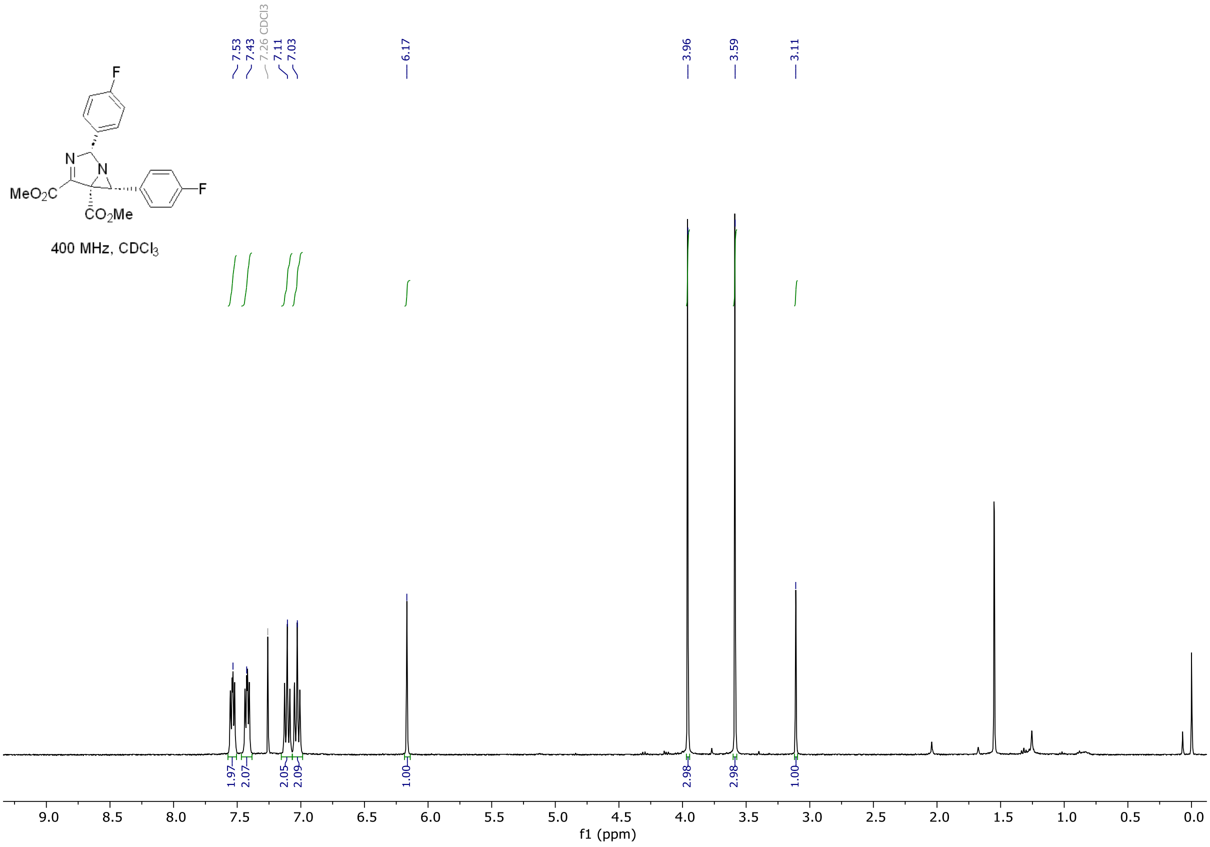

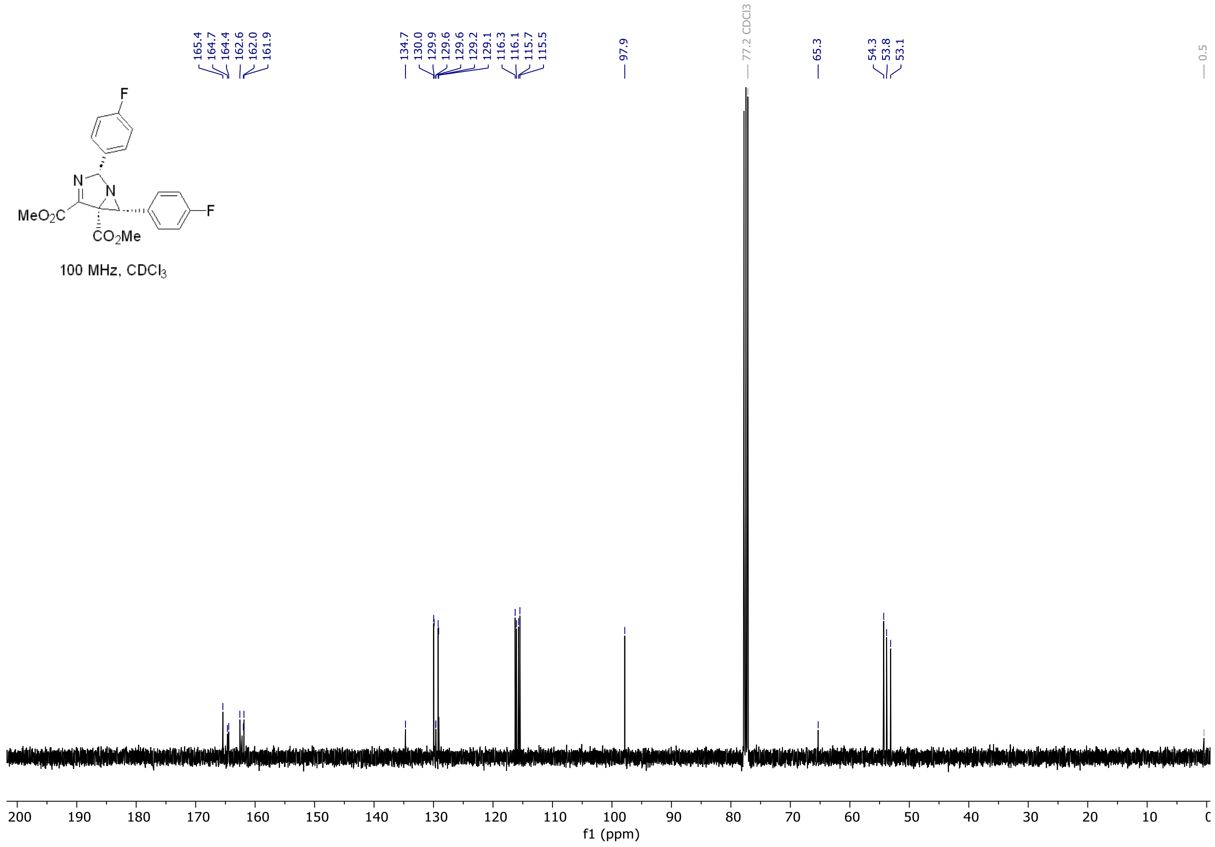
165.4
164.7
164.4
162.6
162.0
161.9
134.7
130.0
129.9
129.6
129.6
129.2
129.1
116.3
116.1
115.7
115.5
97.9
77.2 CDCl3
65.3
54.3
53.8
53.1
0.5
F
N
N
MeO2C
F
CO2Me
100 MHz, CDCl3
200 190 180 170 160 150 140 130 120 110 100 90 80 70 60 50 40 30 20 10 0
f1 (ppm)

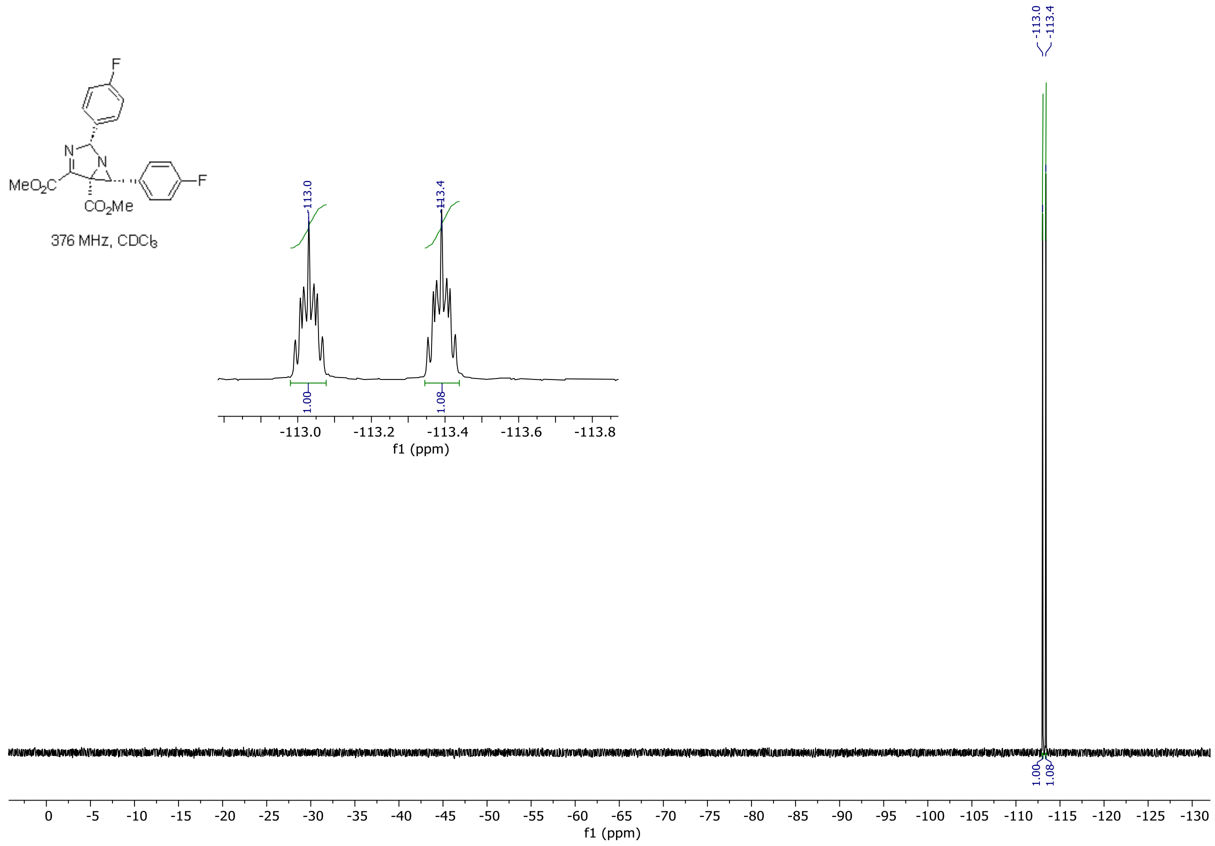
-113.0
-113.4
F
N
N
MeO2C
F
CO2Me
376 MHz, CDCl3
113.0
113.4
1.00
1.08
-113.0 -113.2 -113.4 -113.6 -113.8
f1 (ppm)
0 -5 -10 -15 -20 -25 -30 -35 -40 -45 -50 -55 -60 -65 -70 -75 -80 -85 -90 -95 -100 -105 -110 -115 -120 -125 -130
f1 (ppm)

(+/-)Dimethyl(2S,5S,6R)-2,6-bis(4-bromophenyl)-1,3-diazabicyclo[3.1.0]hex-3-ene-4,5-dicarboxylate (3c)

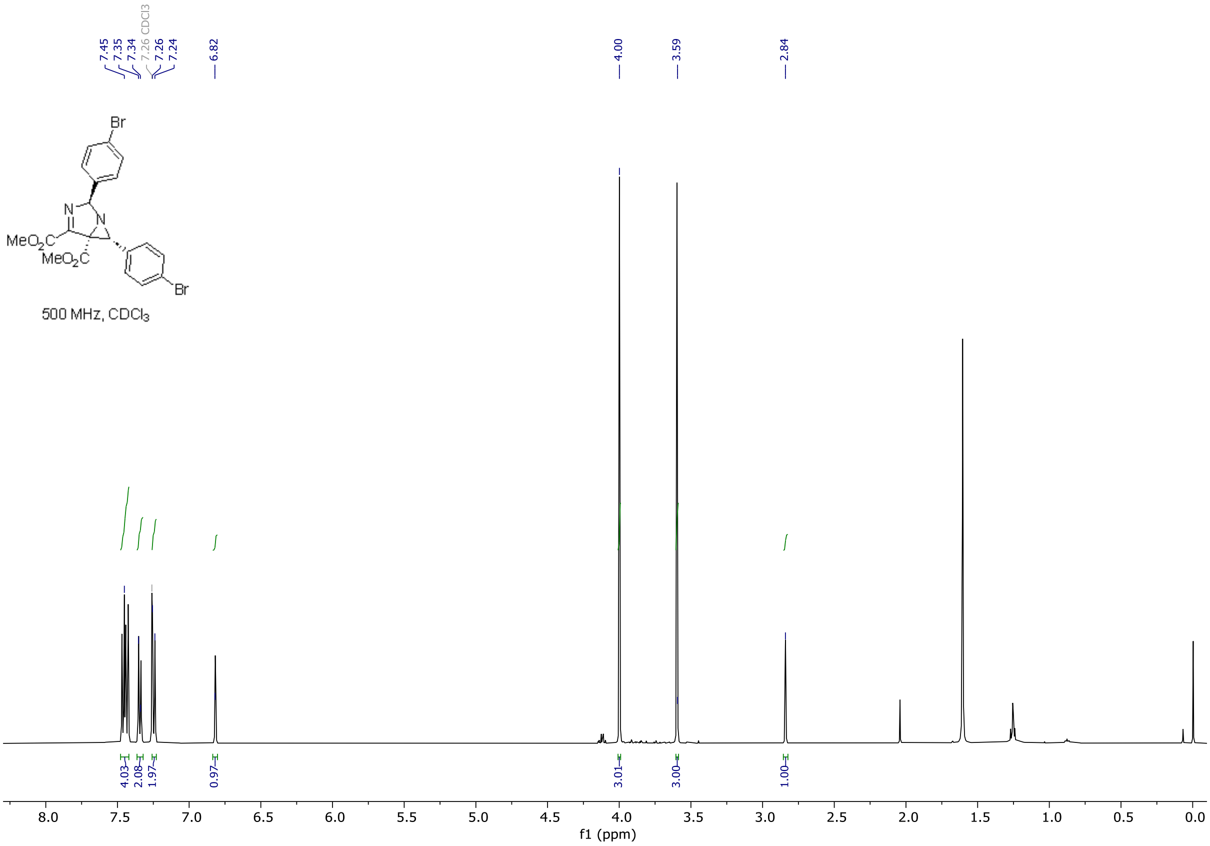

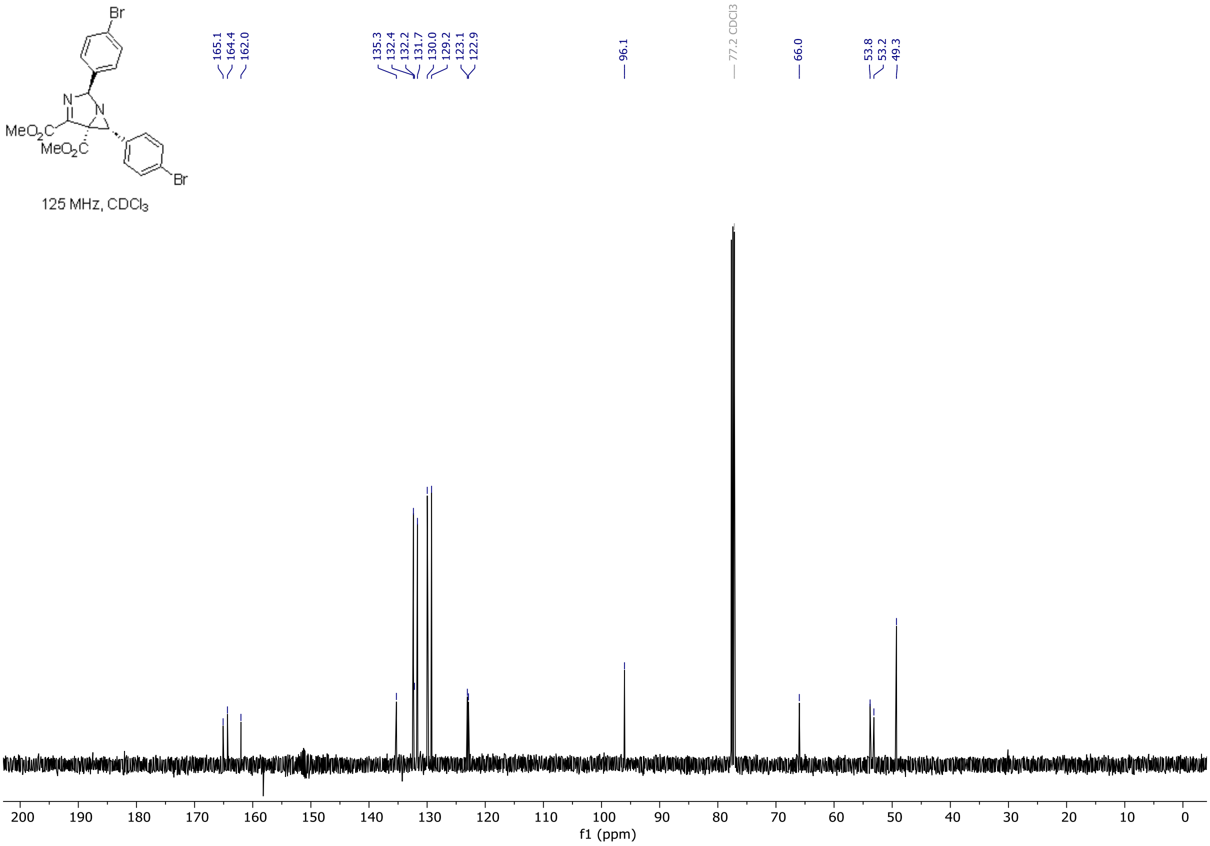

(+/-)Dimethyl(2R,5S,6R)-2,6-bis(4-bromophenyl)-1,3-diazabicyclo[3.1.0]hex-3-ene-4,5-dicarboxylate (3c')

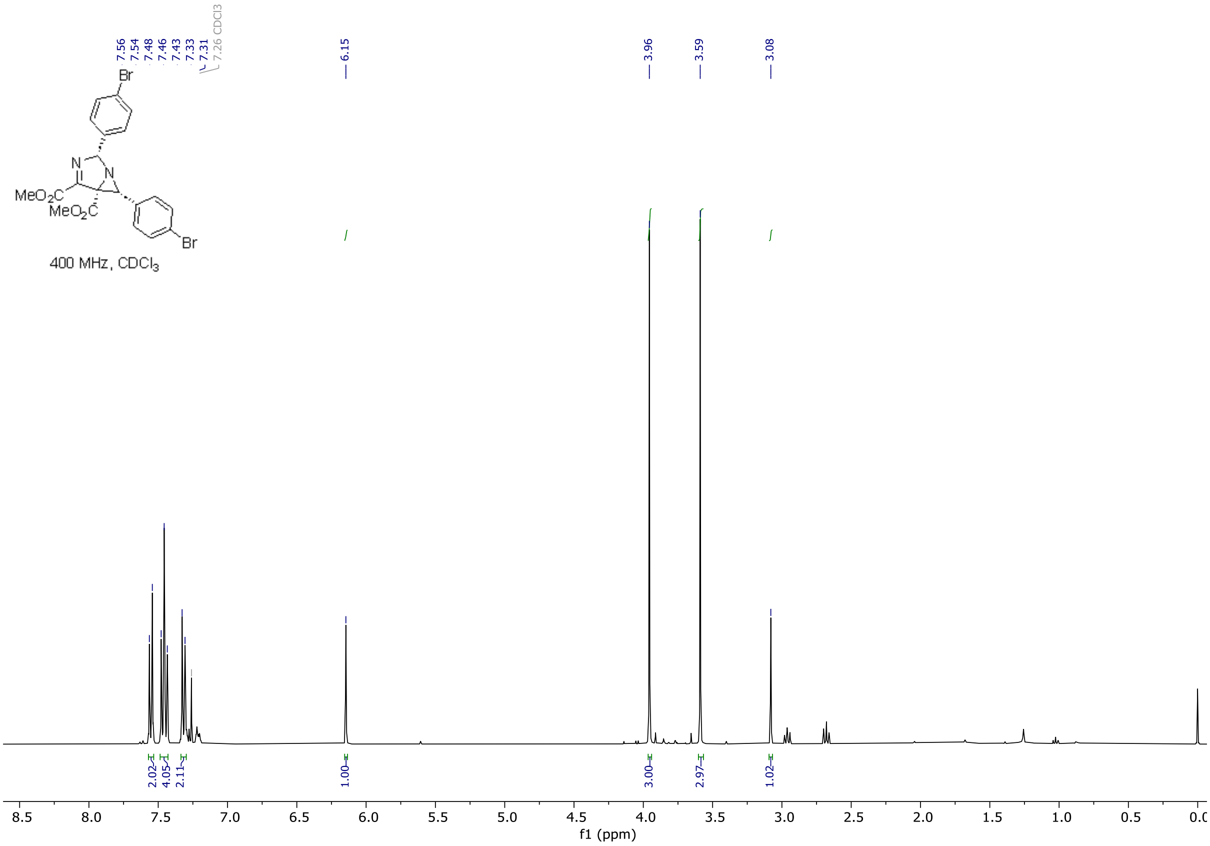

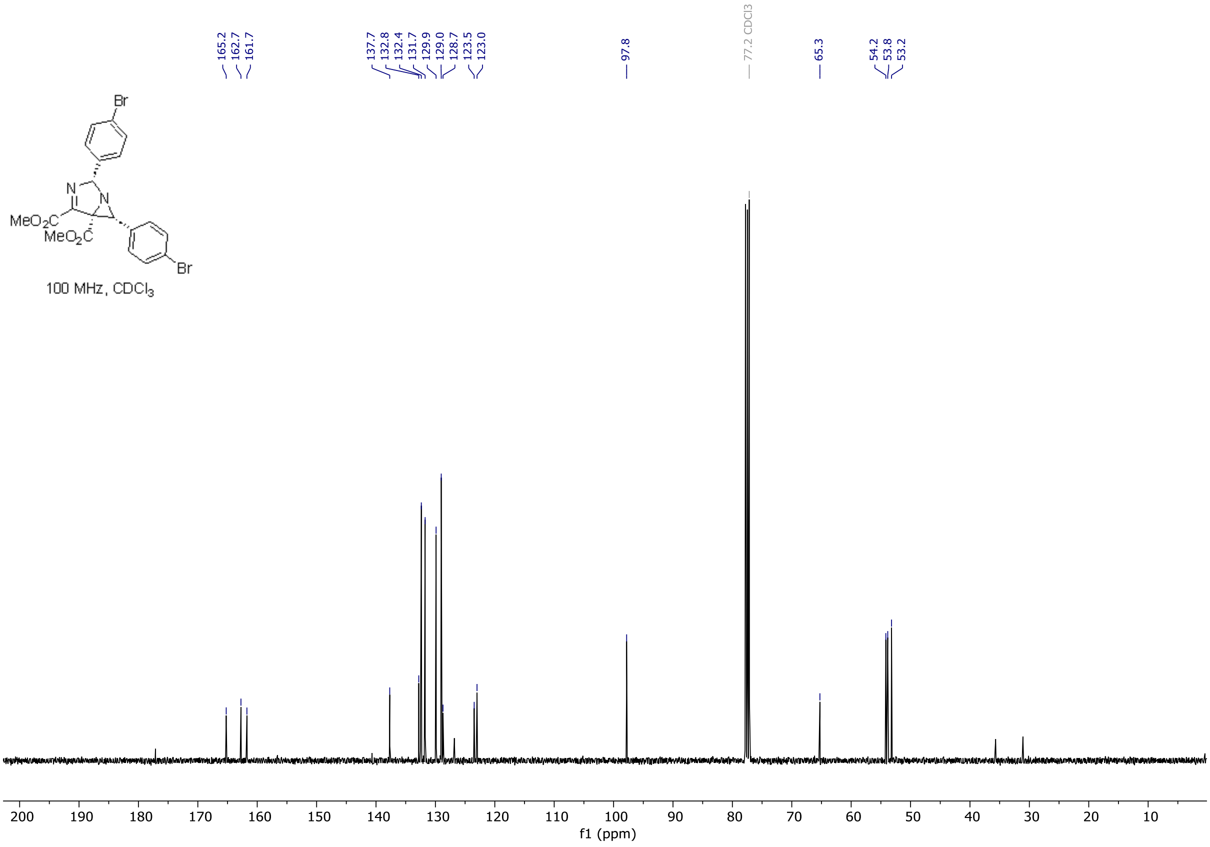

(+/-)Dimethyl (2S,5S,6R)-2,6-di-p-tolyl-1,3-diazabicyclo[3.1.0]hex-3-ene-4,5-dicarboxylate (3d)

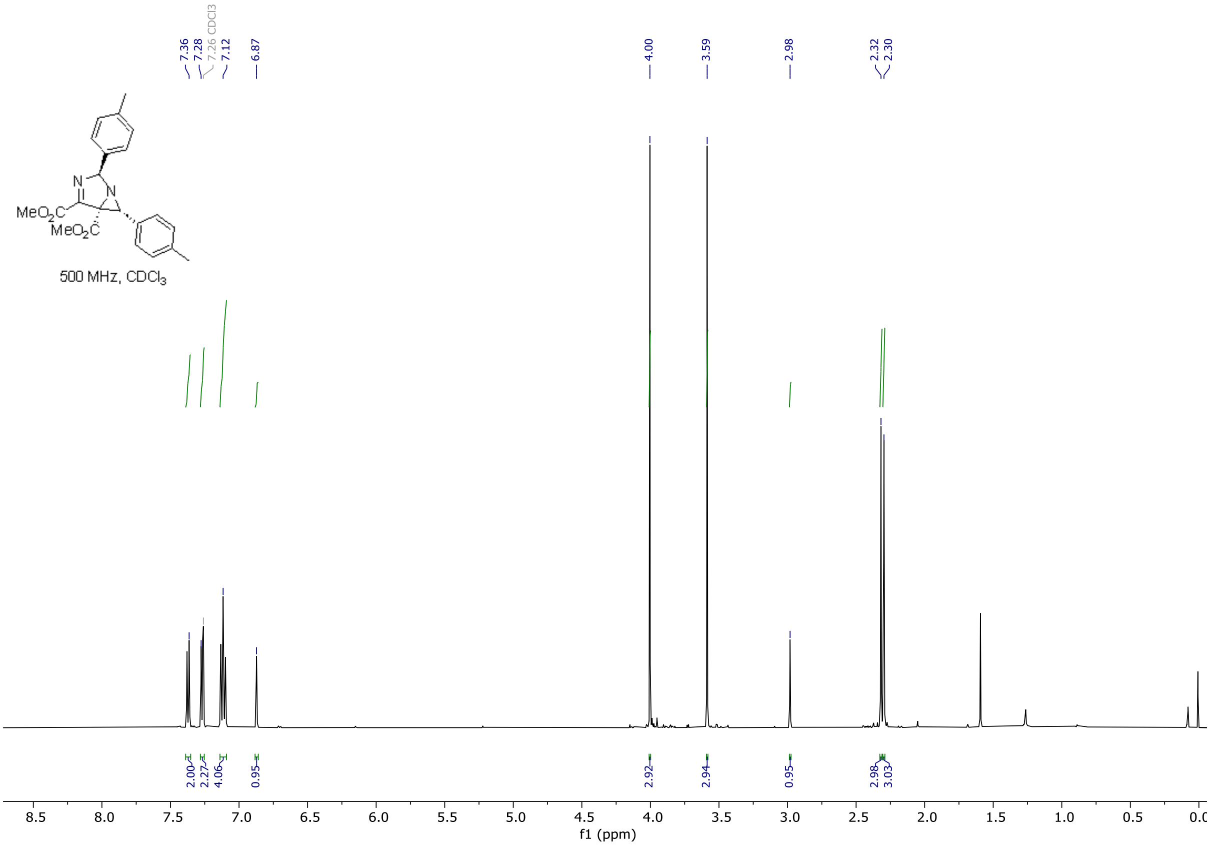

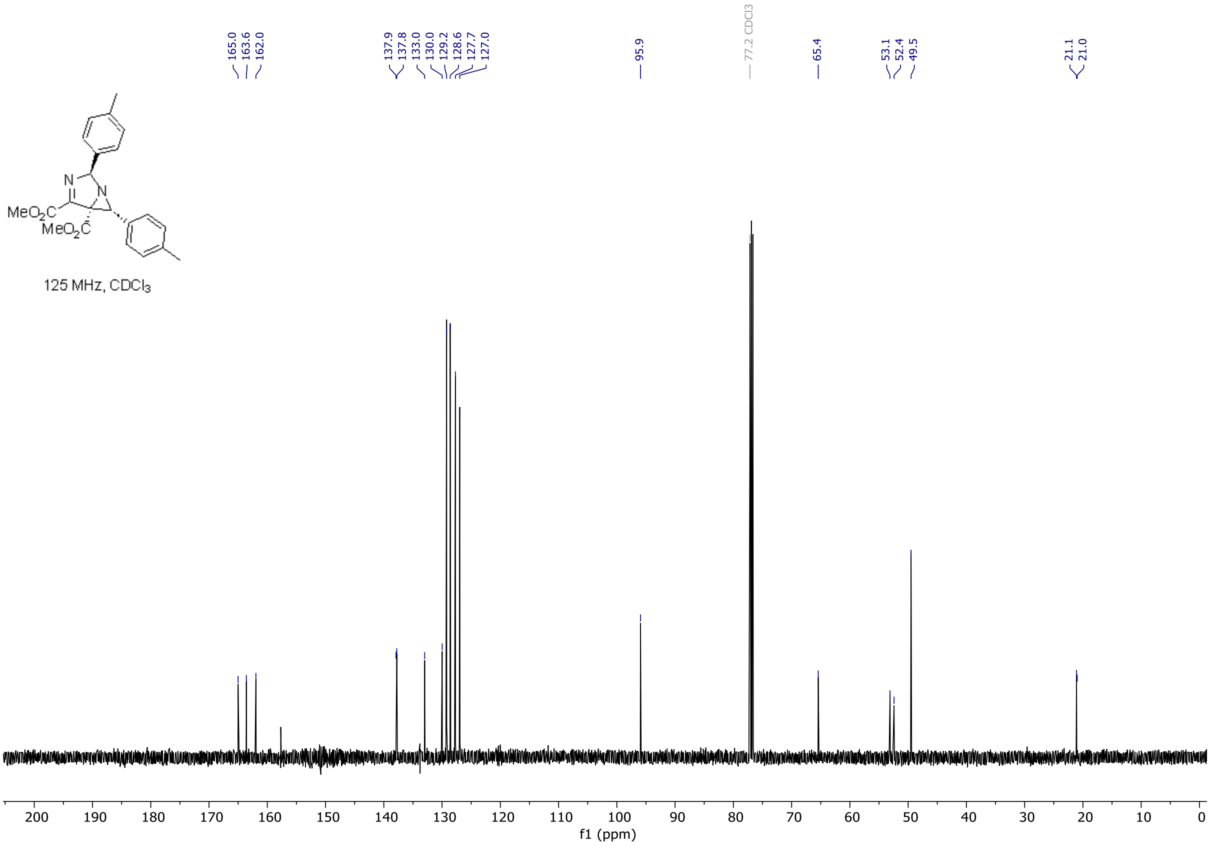

(+/-)Dimethyl(2R,5S,6R)-2,6-di-p-tolyl-1,3-diazabicyclo[3.1.0]hex-3-ene-4,5-dicarboxylate (3d')

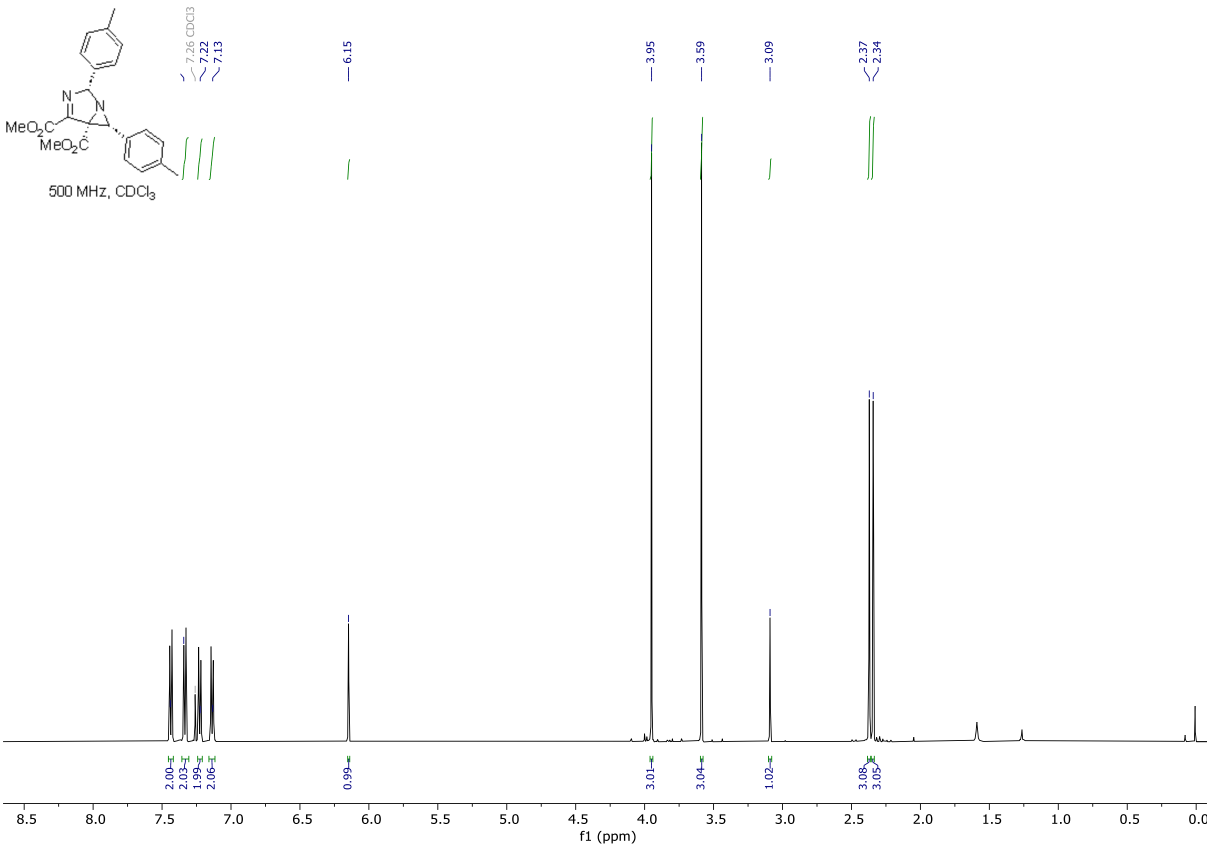

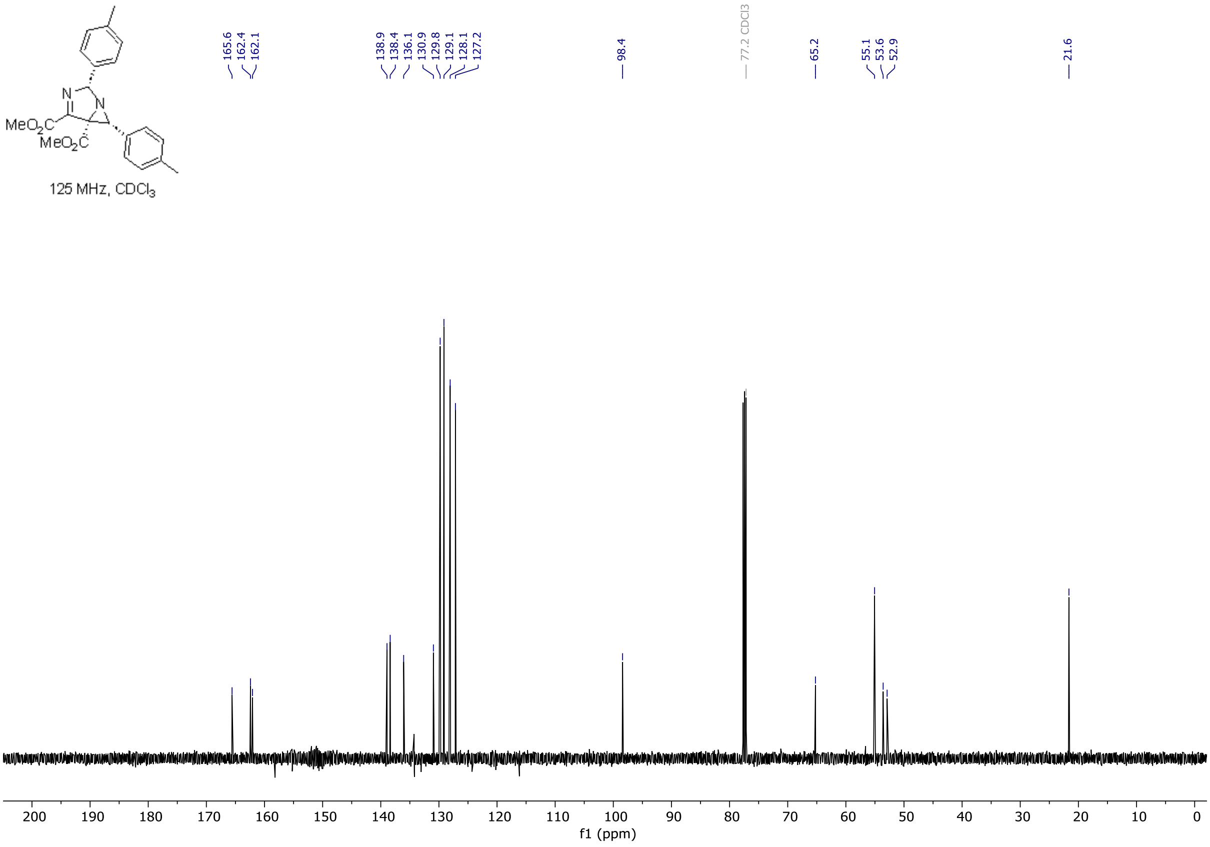

(+/-)Dimethyl(5S,6R)-2,6-bis(3,4-dimethylphenyl)-1,3-diazabicyclo[3.1.0]hex-3-ene-4,5-dicarboxylate (3e and 3e')

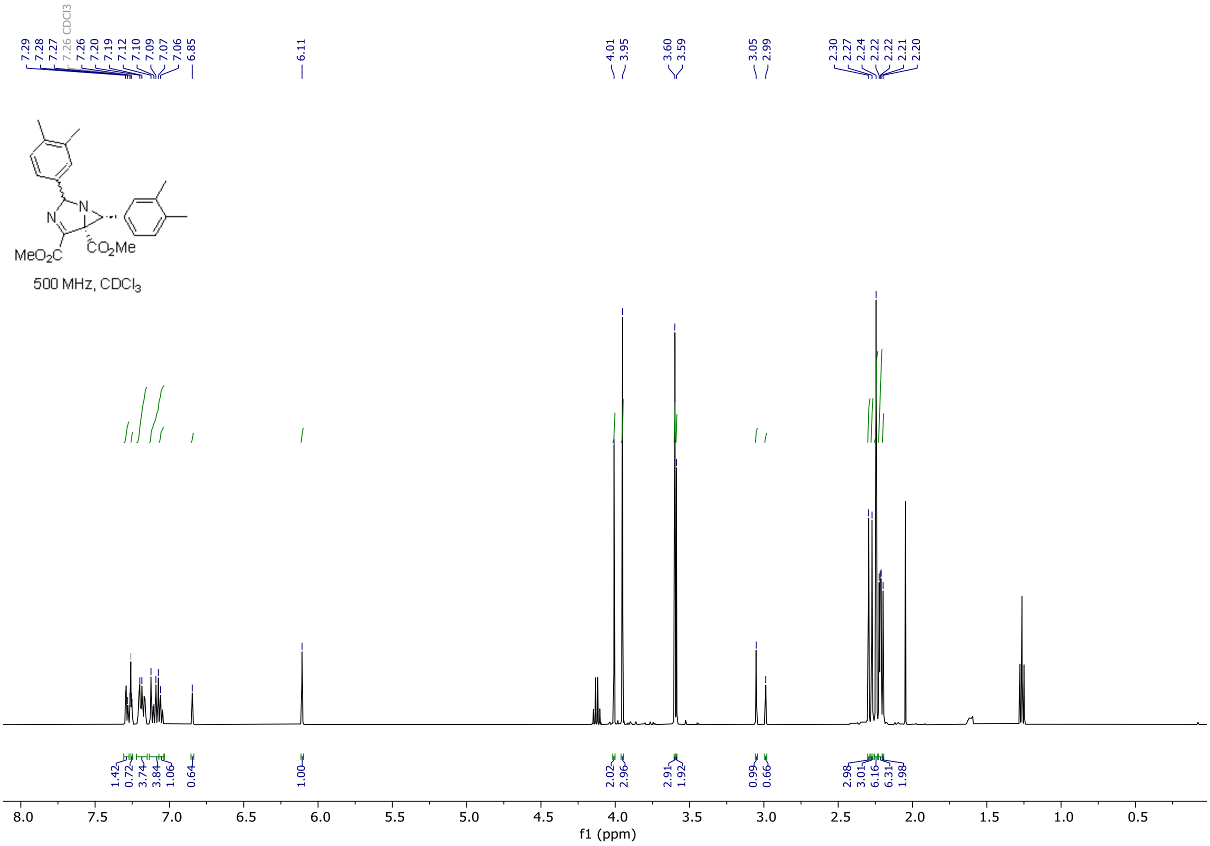

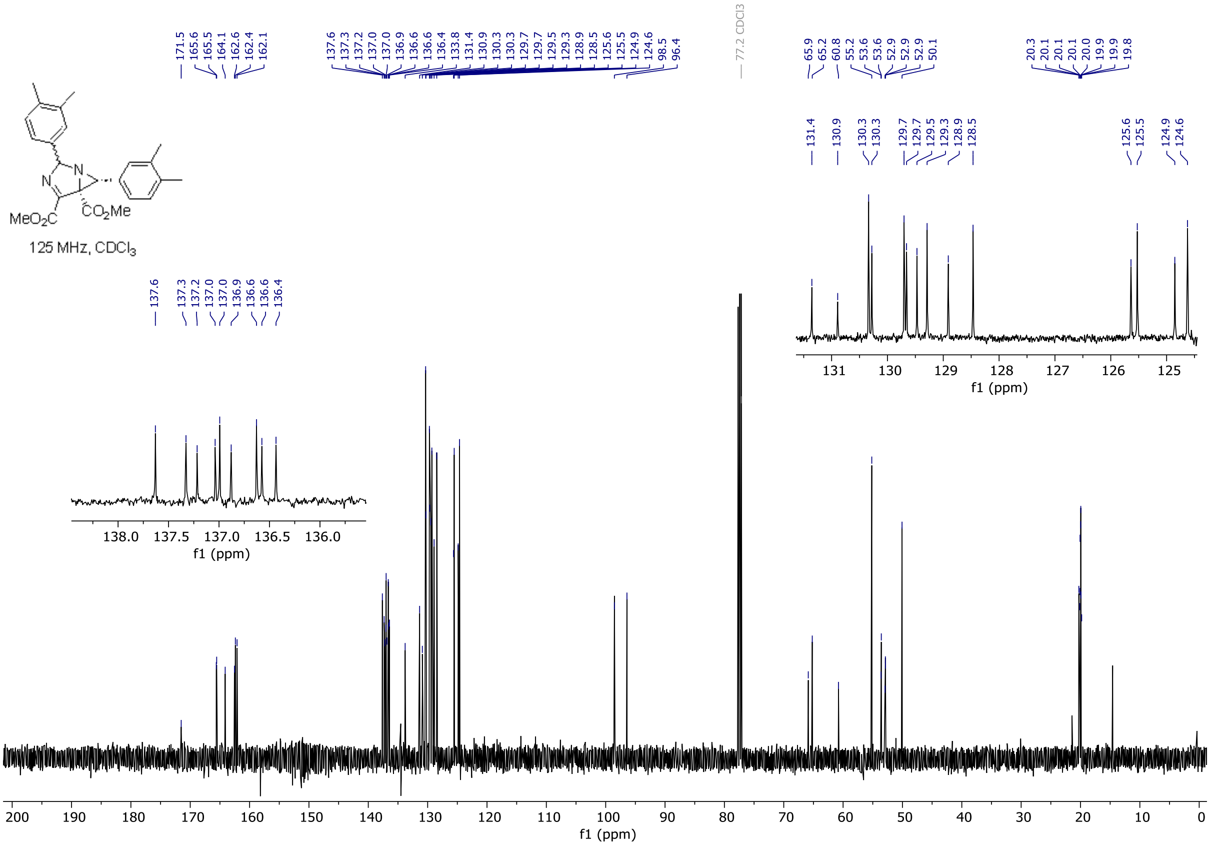

(+/-)Dimethyl(5S,6R)-2,6-di-o-tolyl-1,3-diazabicyclo[3.1.0]hex-3-ene-4,5-dicarboxylate (3f/3f')

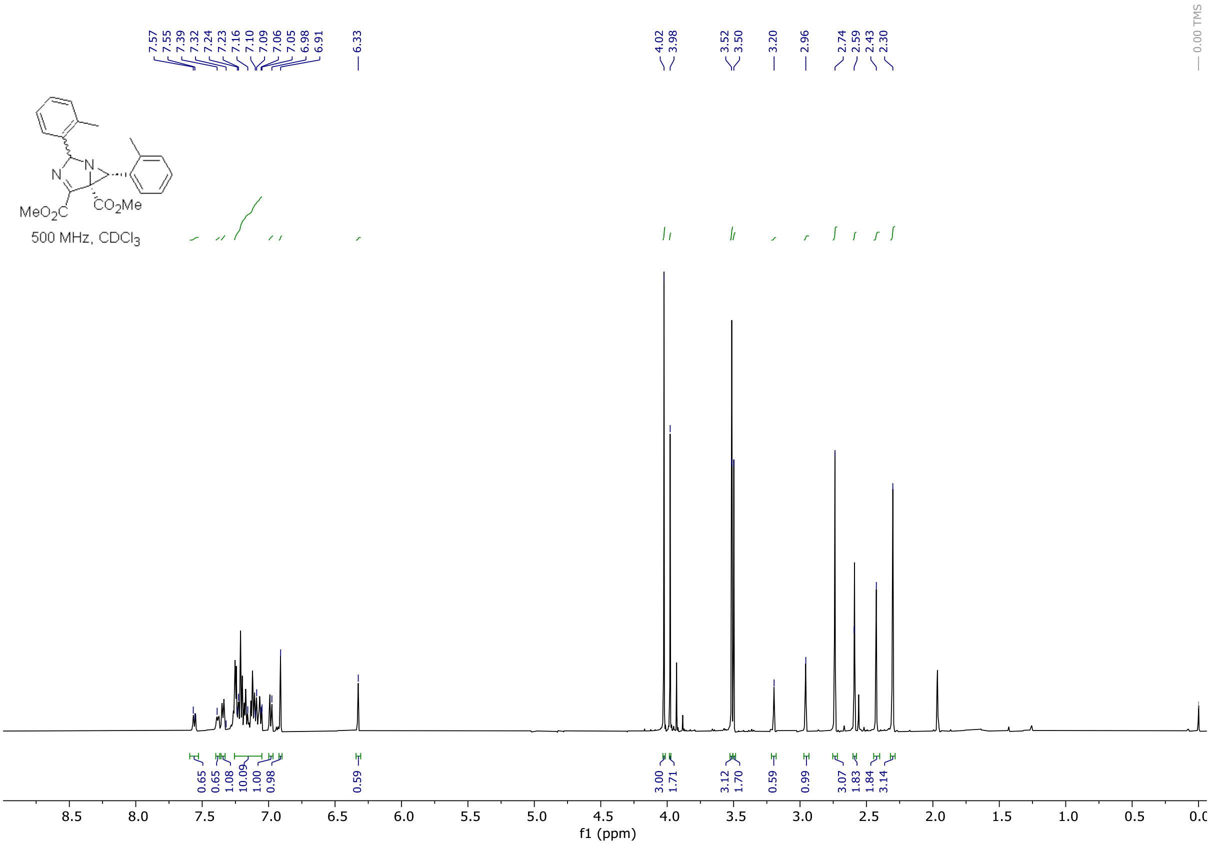

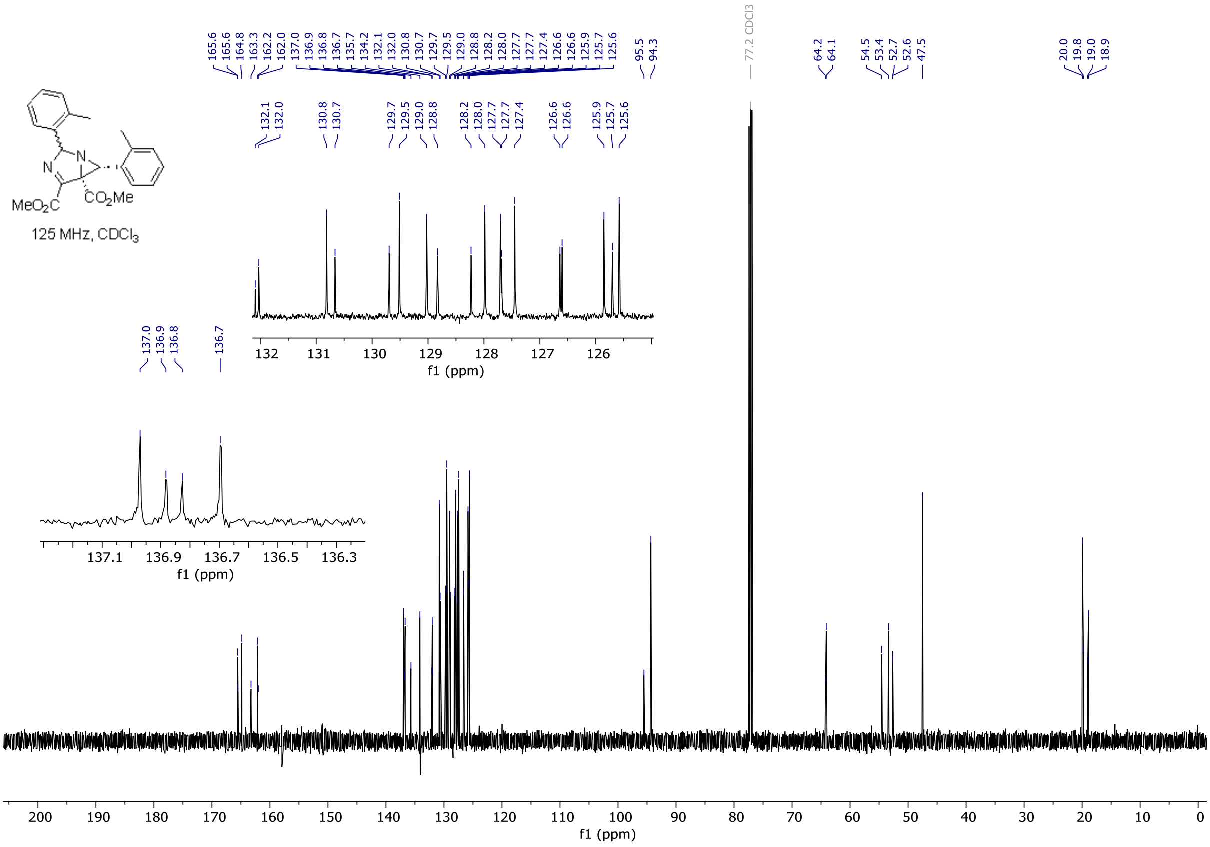

(+/-)Dimethyl(2R,5S,6R)-2,6-bis(4-methoxyphenyl)-1,3-diazabicyclo[3.1.0]hex-3-ene-4,5-dicarboxylate (3g')

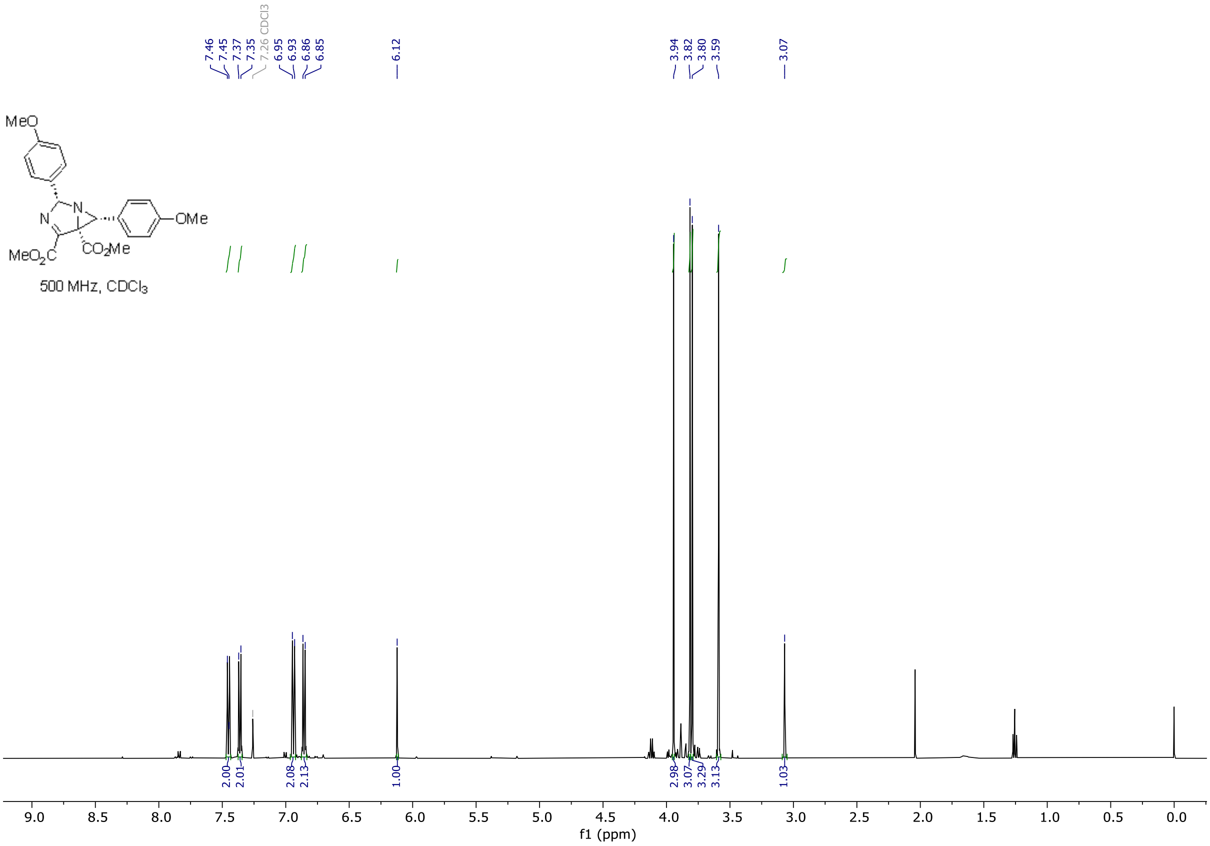

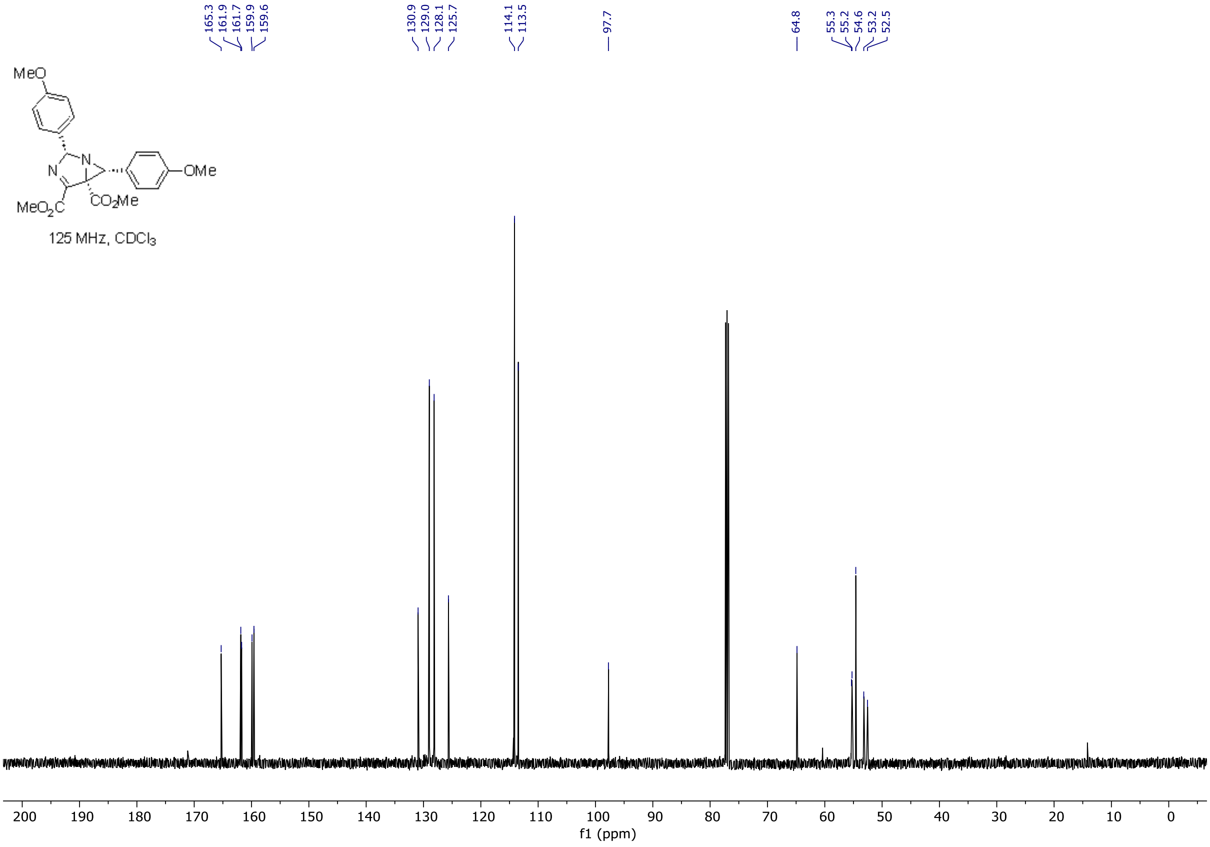

(+/-)Dimethyl(2R,5S,6R)-2,6-bis(2-(trifluoromethyl)phenyl)-1,3-diazabicyclo[3.1.0]hex-3-ene-4,5-dicarboxylate (3h')

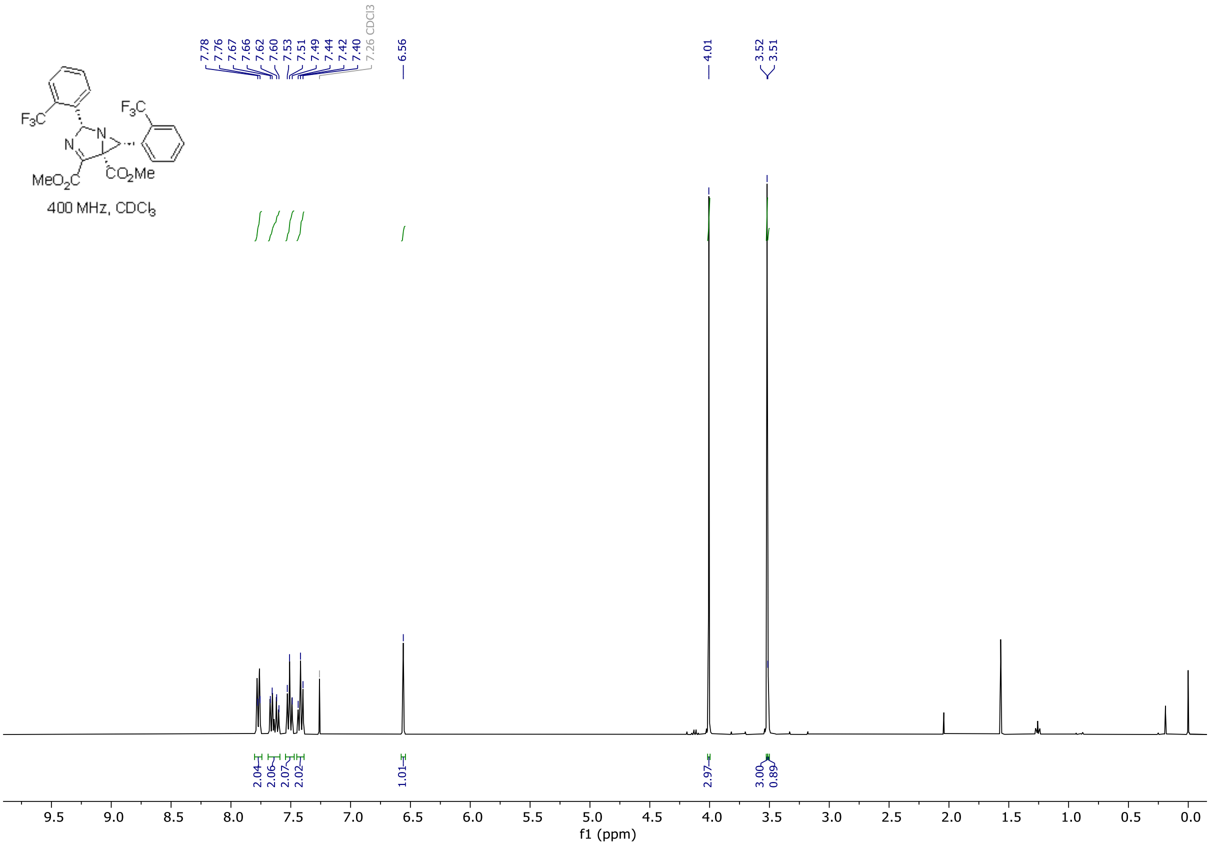

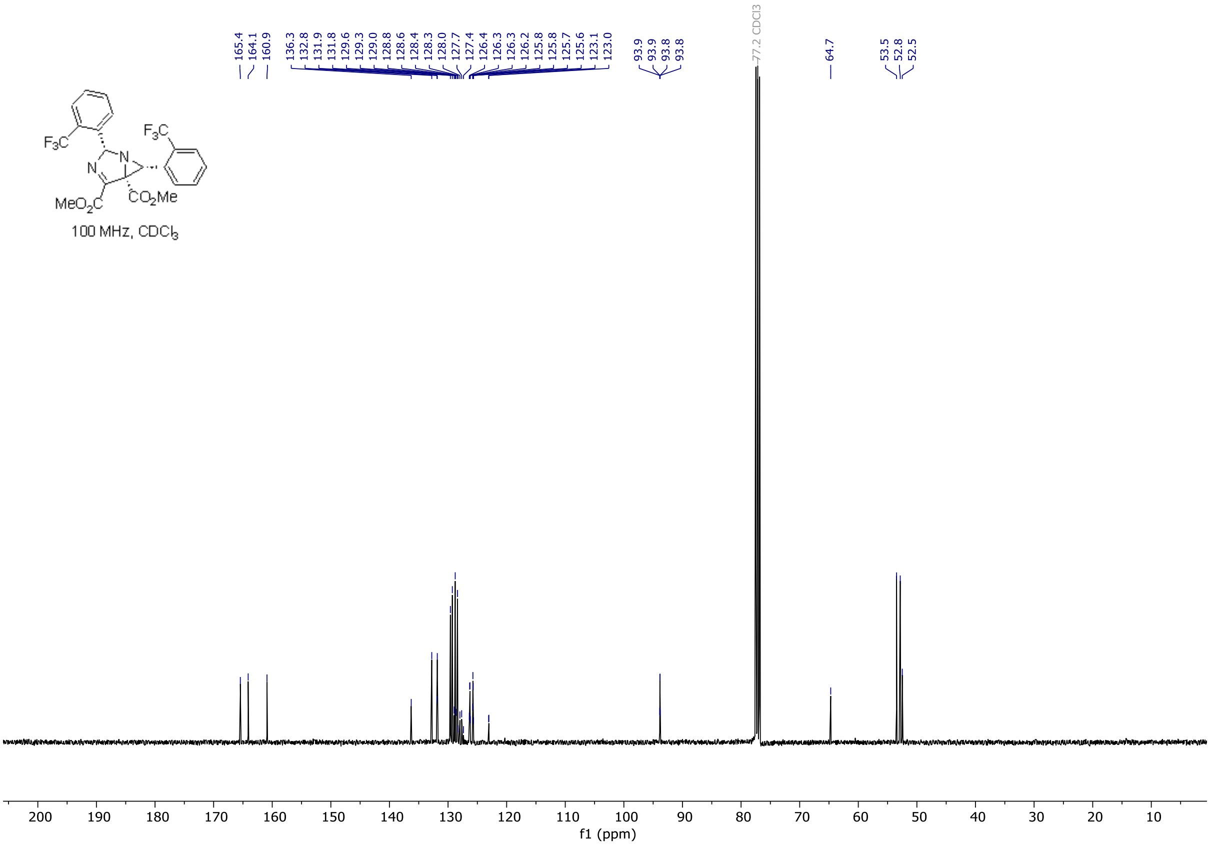

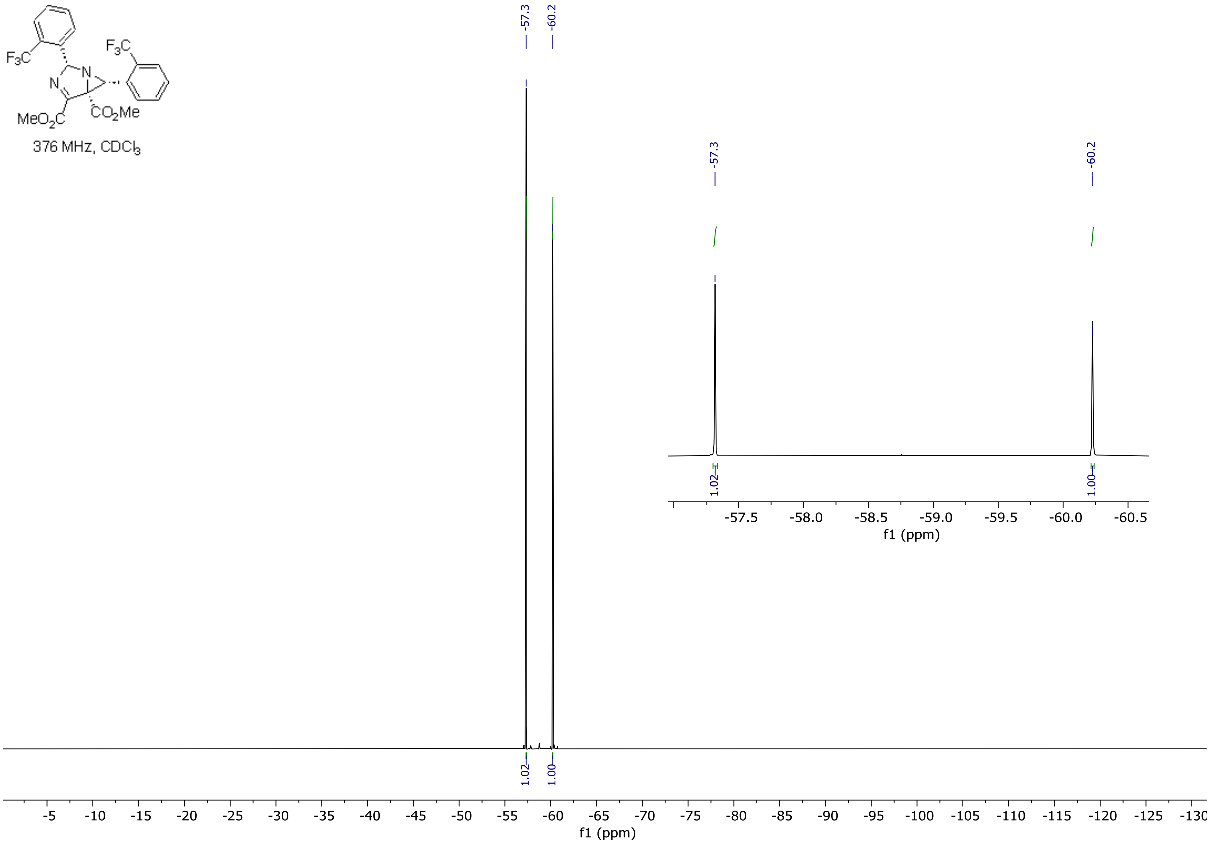
F3C
F3C
N
N
MeO2C
CO2Me
376 MHz, CDCl3
-57.3
-60.2
1.02
1.00
f1 (ppm)

(+/-)Dimethyl(5S,6R)-2,6-bis(2,4-difluorophenyl)-1,3-diazabicyclo[3.1.0]hex-3-ene-4,5-dicarboxylate (3i/3i')

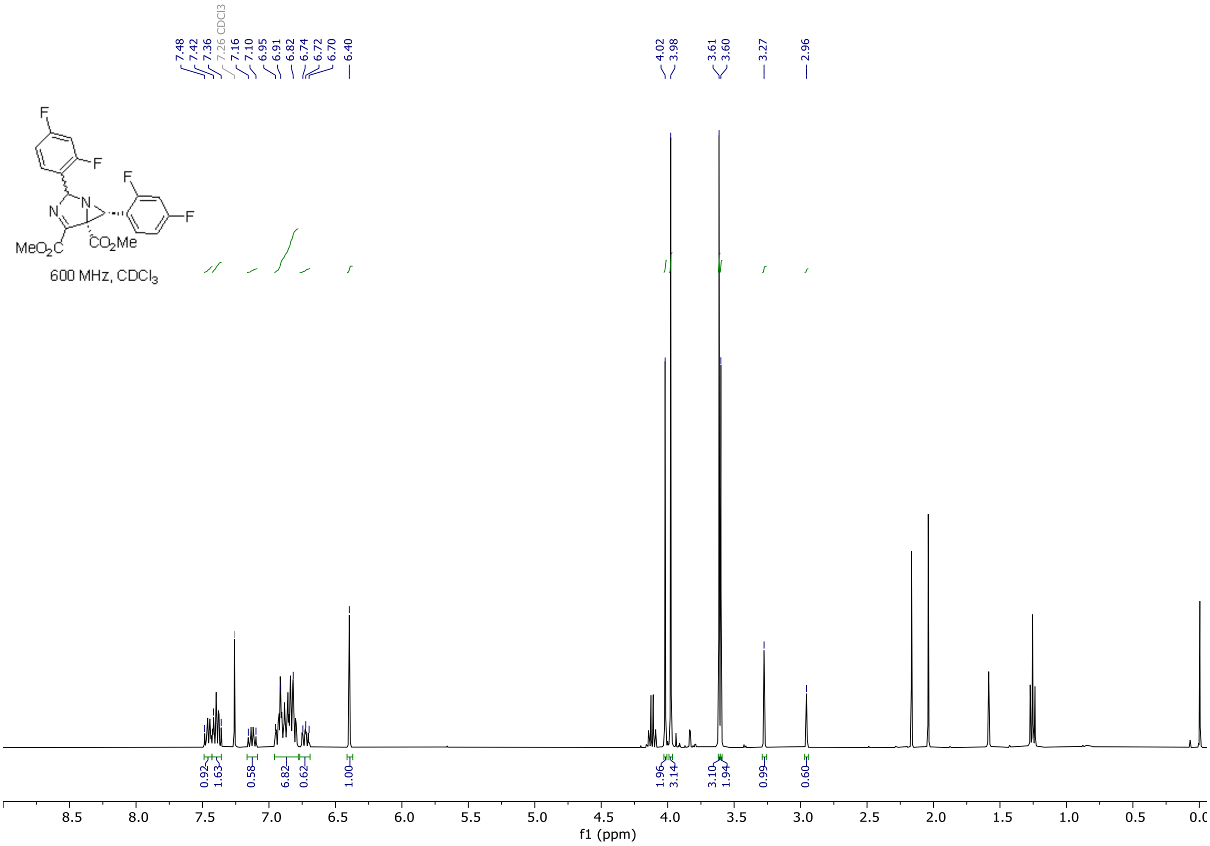

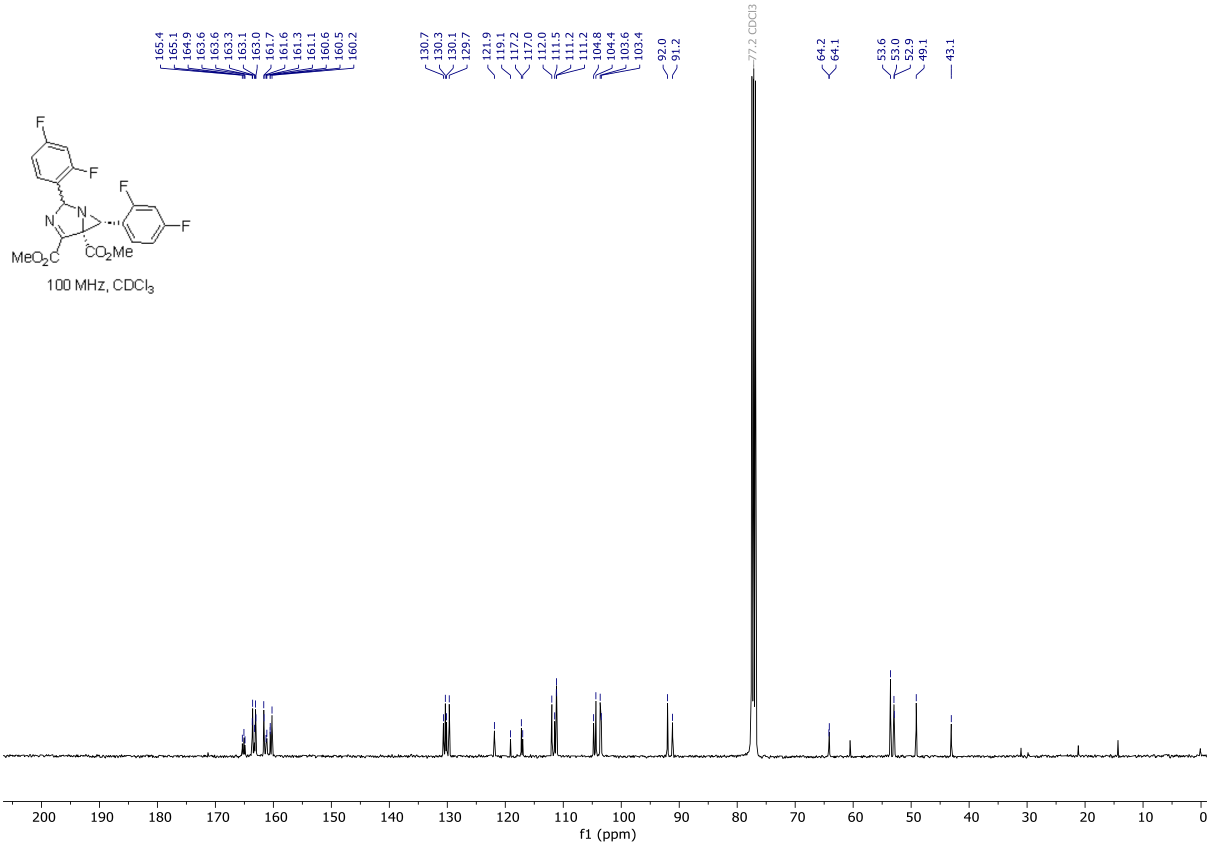

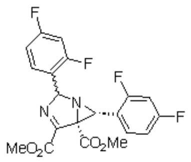
376 MHz, $CDCl_3$

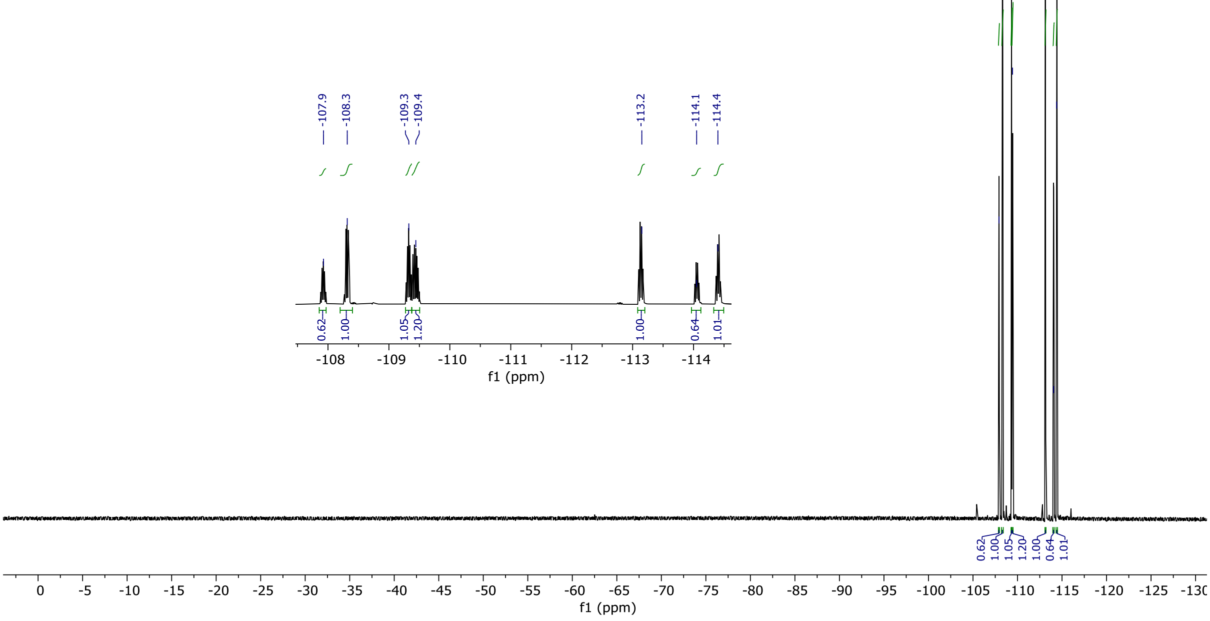

(+/-)Dimethyl(2S,5S,6R)-2,6-bis(3-methoxyphenyl)-1,3-diazabicyclo[3.1.0]hex-3-ene-4,5-dicarboxylate (3j)

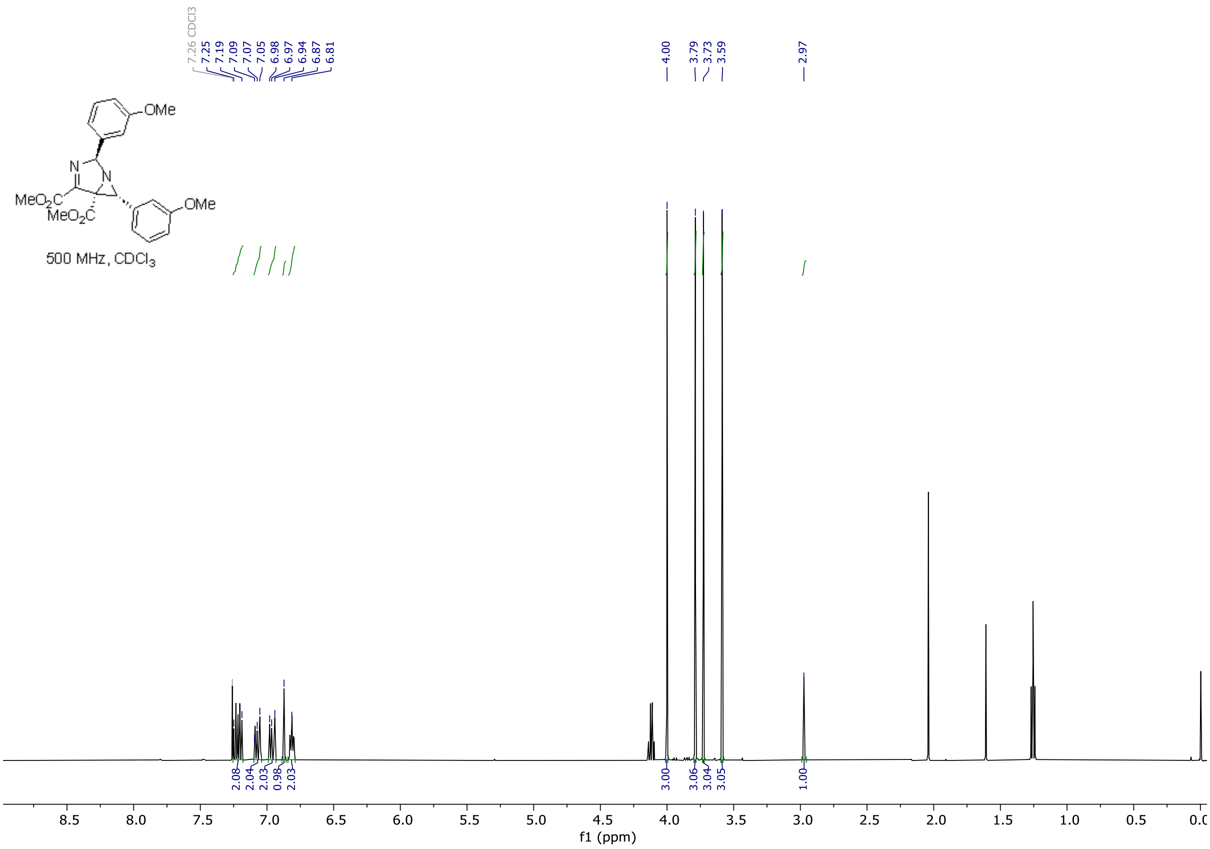

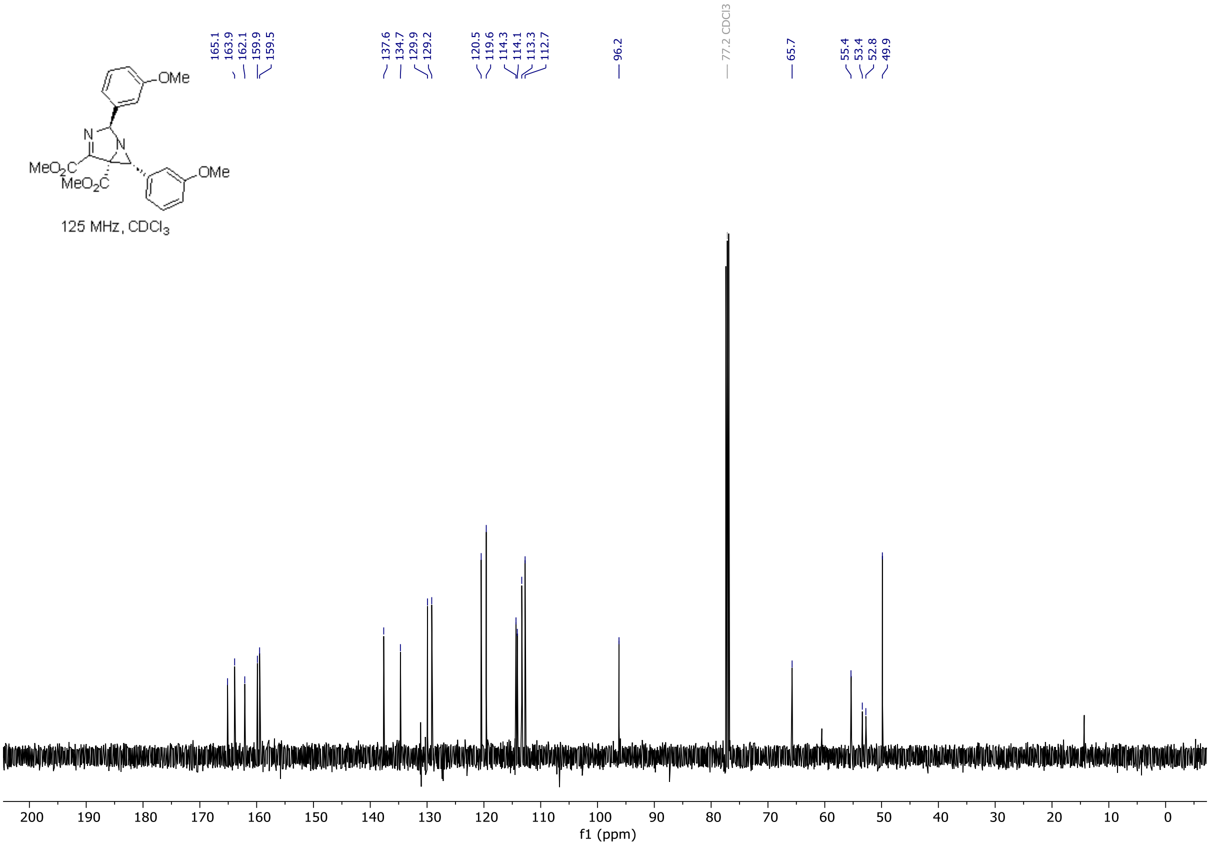

(+/-)Dimethyl(2R,5S,6R)-2,6-bis(3-methoxyphenyl)-1,3-diazabicyclo[3.1.0]hex-3-ene-4,5-dicarboxylate (3j')

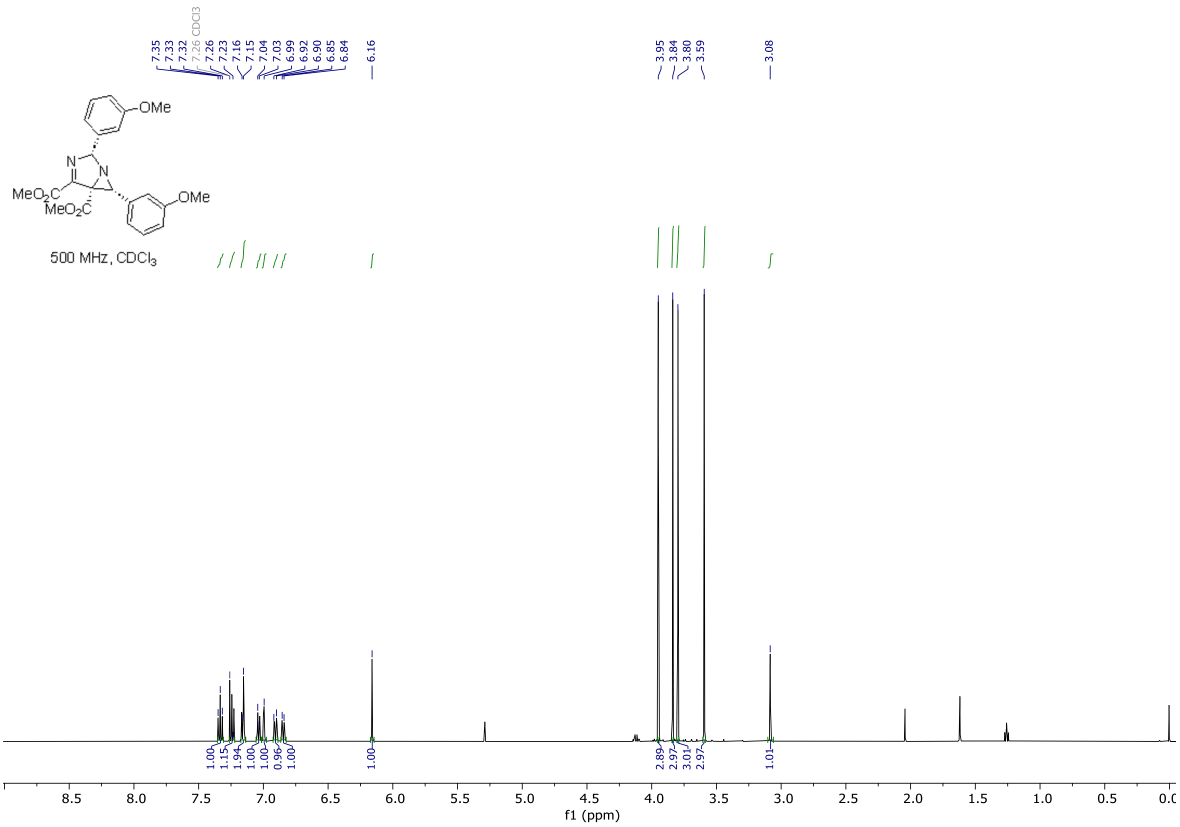

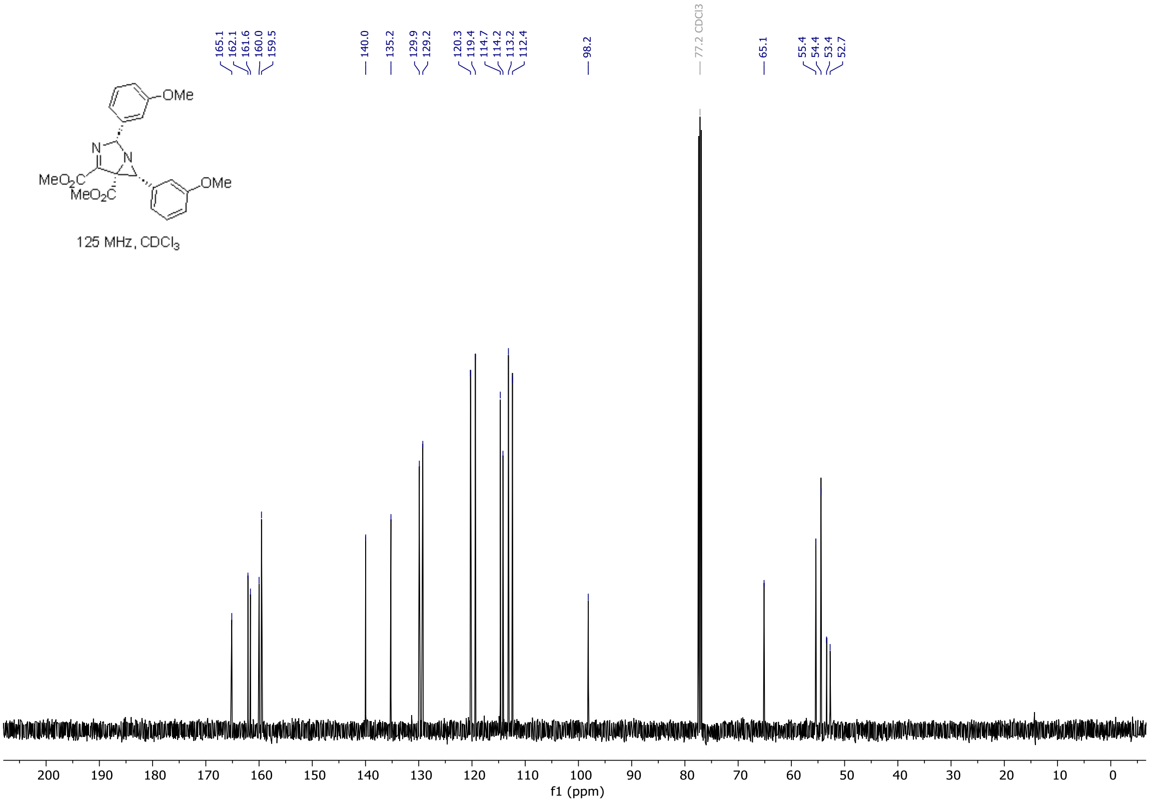

(+/-)Di-tert-butyl(2S,5S,6R)-2,6-diphenyl-1,3-diazabicyclo[3.1.0]hex-3-ene-4,5-dicarboxylate (3k)

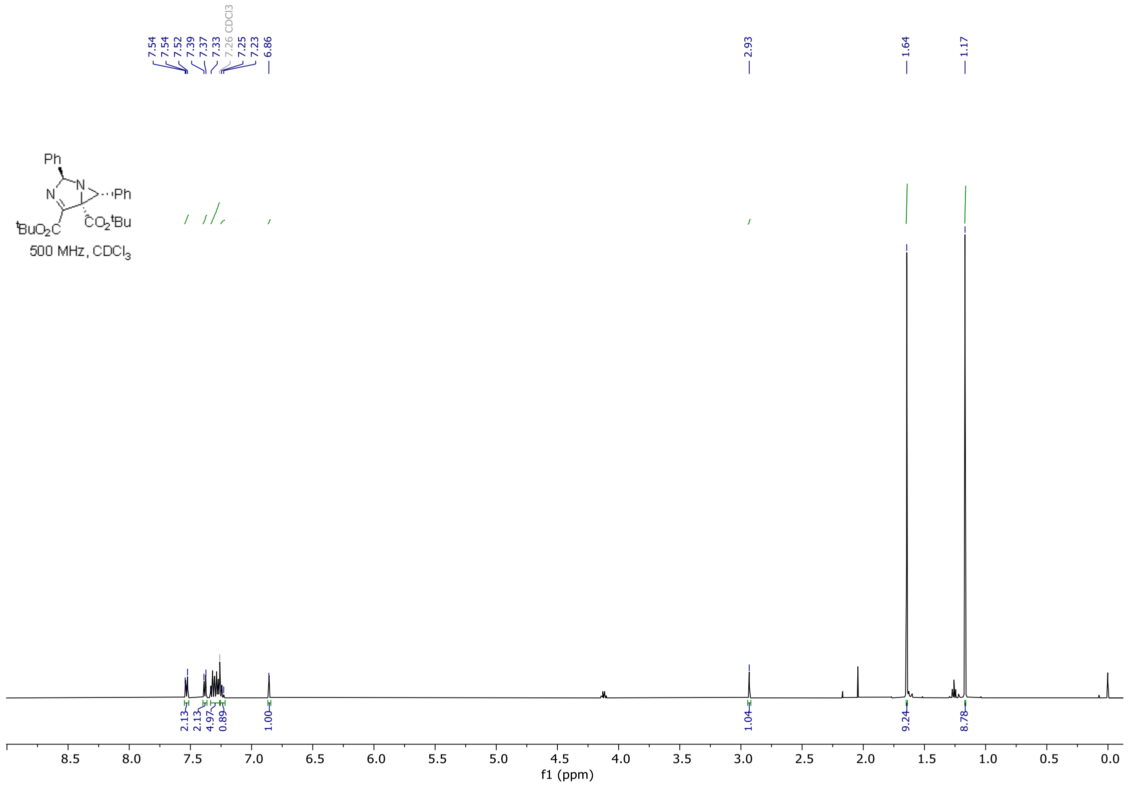

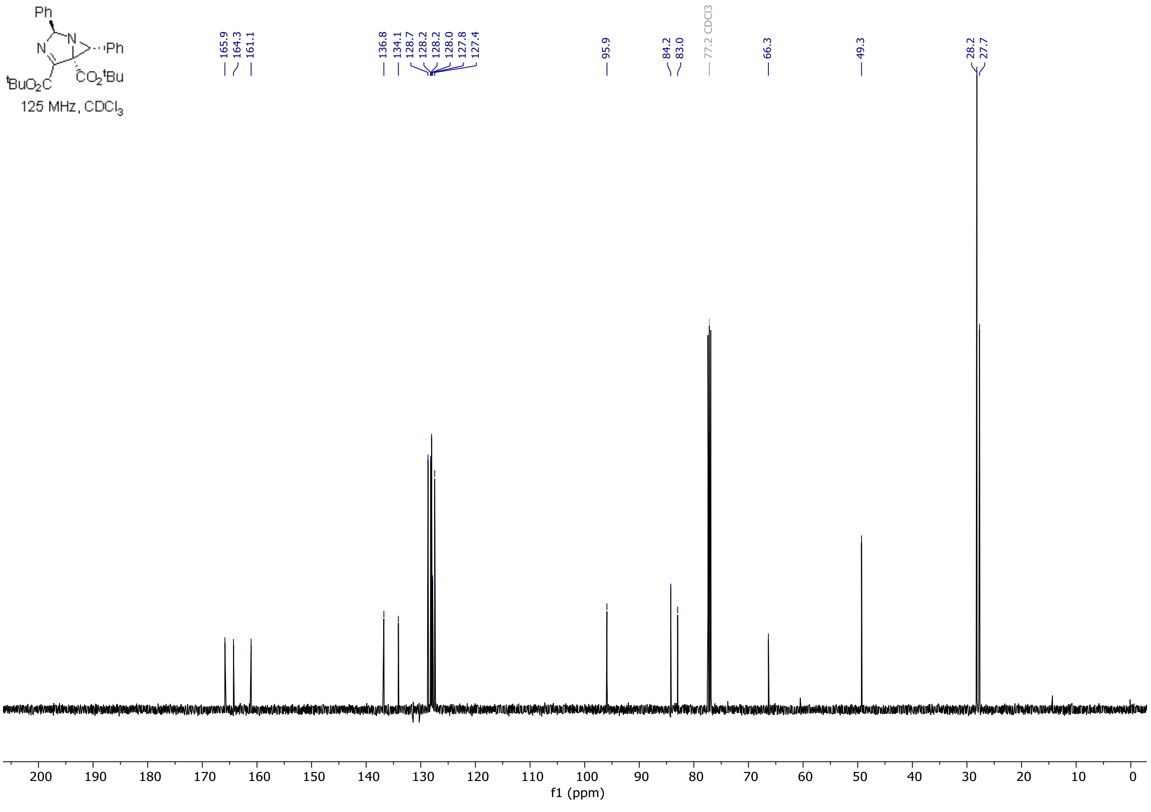

(+/-)Di-tert-butyl(2R,5S,6R)-2,6-diphenyl-1,3-diazabicyclo[3.1.0]hex-3-ene-4,5-dicarboxylate (3k')

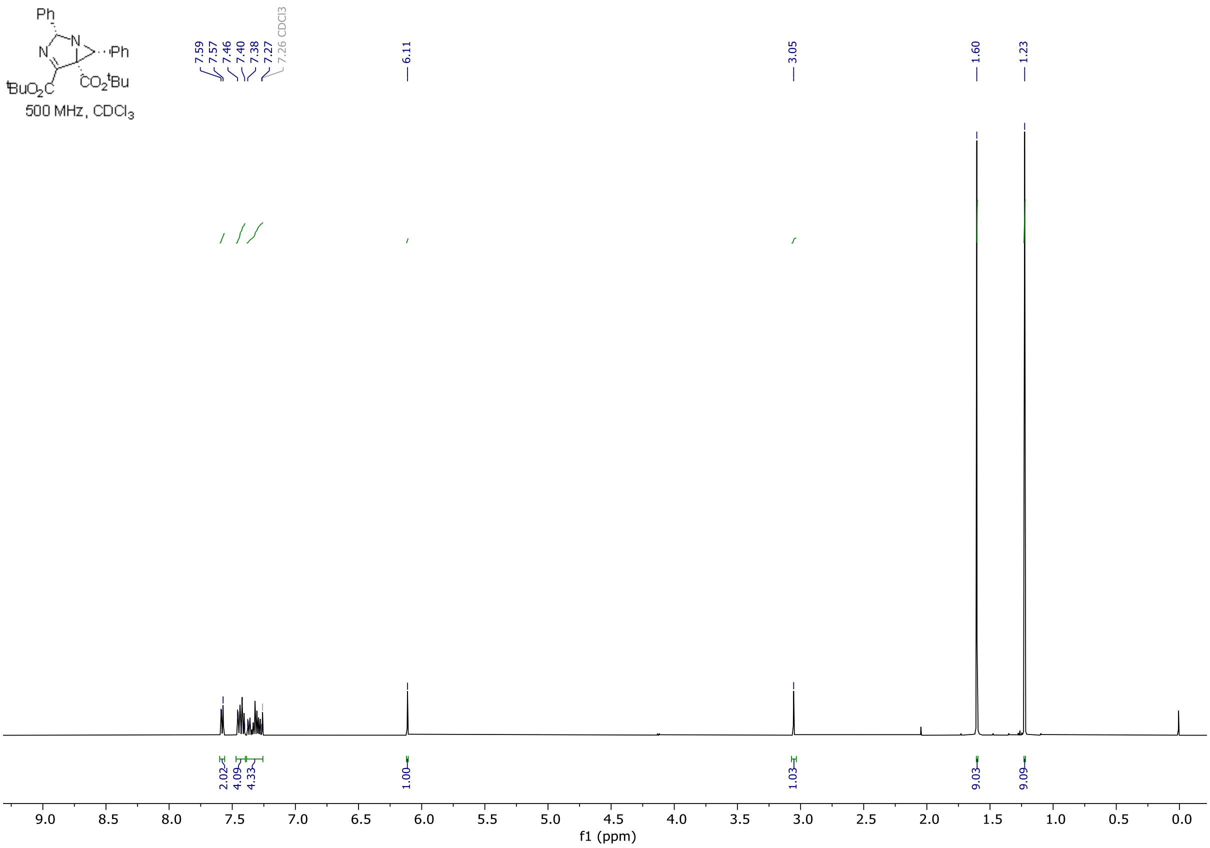

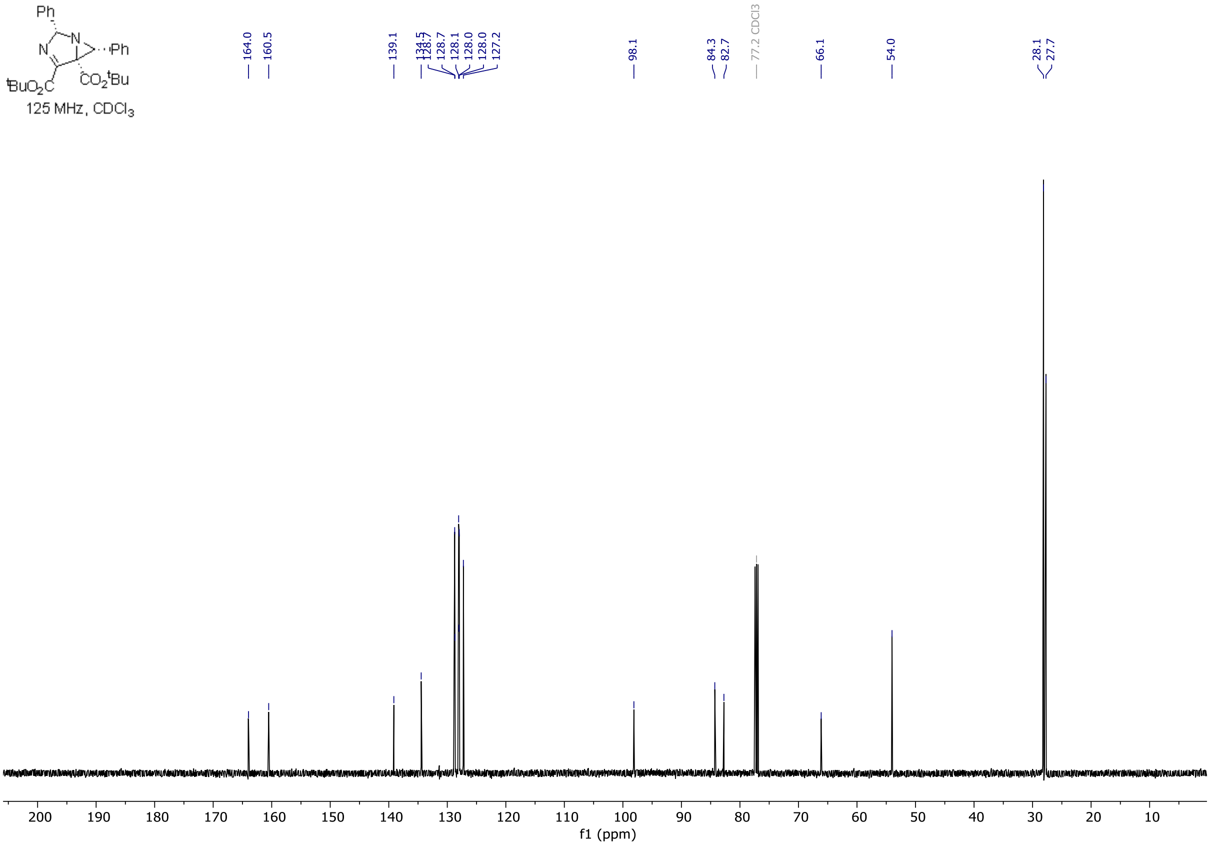

(+/-)Di-tert-butyl(2S,5S,6R)-2,6-bis(4-fluorophenyl)-1,3-diazabicyclo[3.1.0]hex-3-ene-4,5-dicarboxylate (3l)

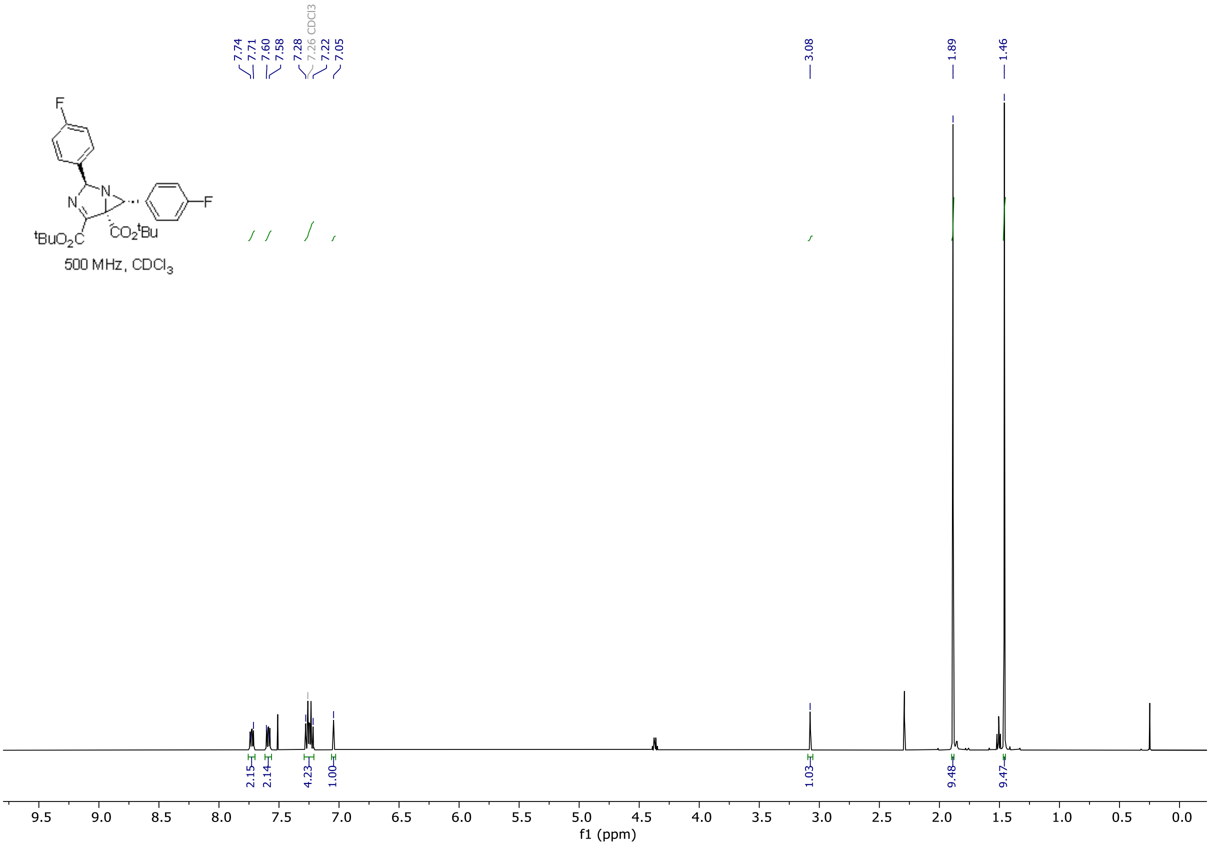

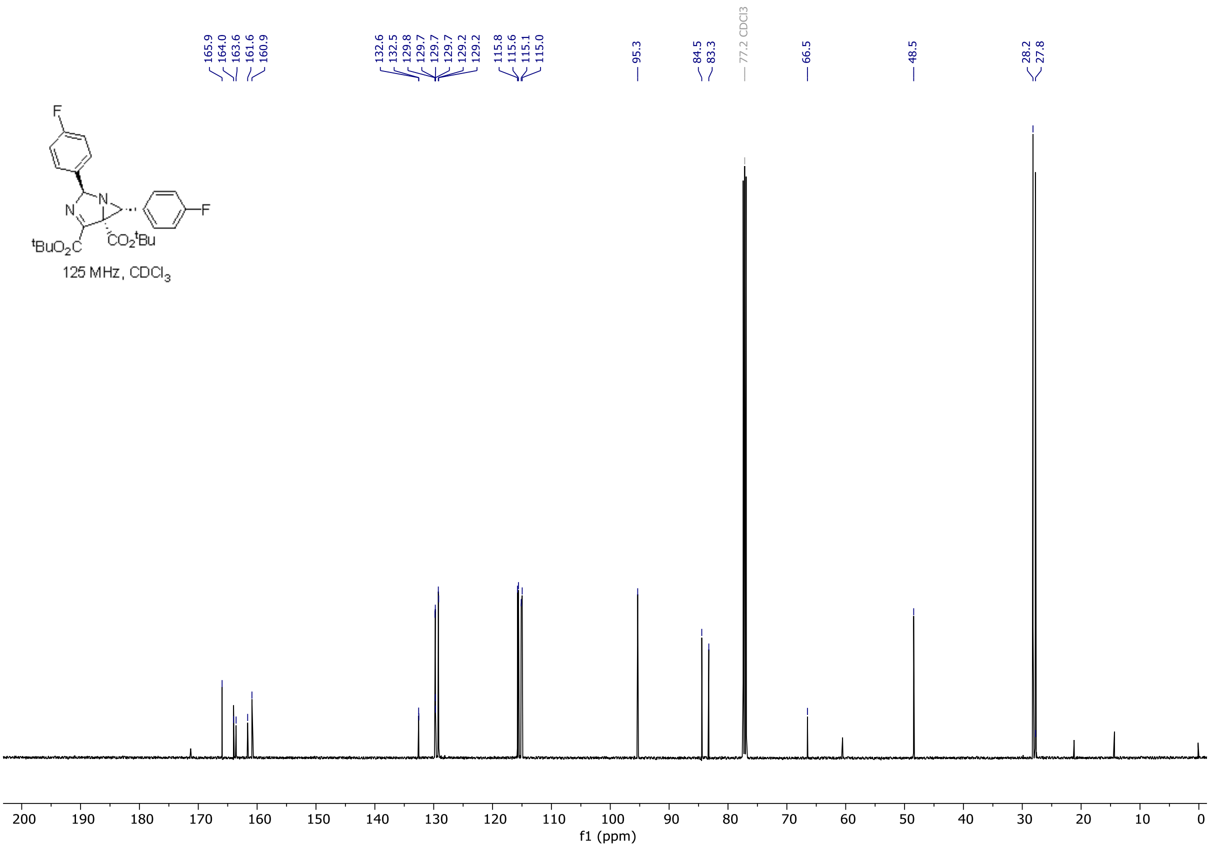

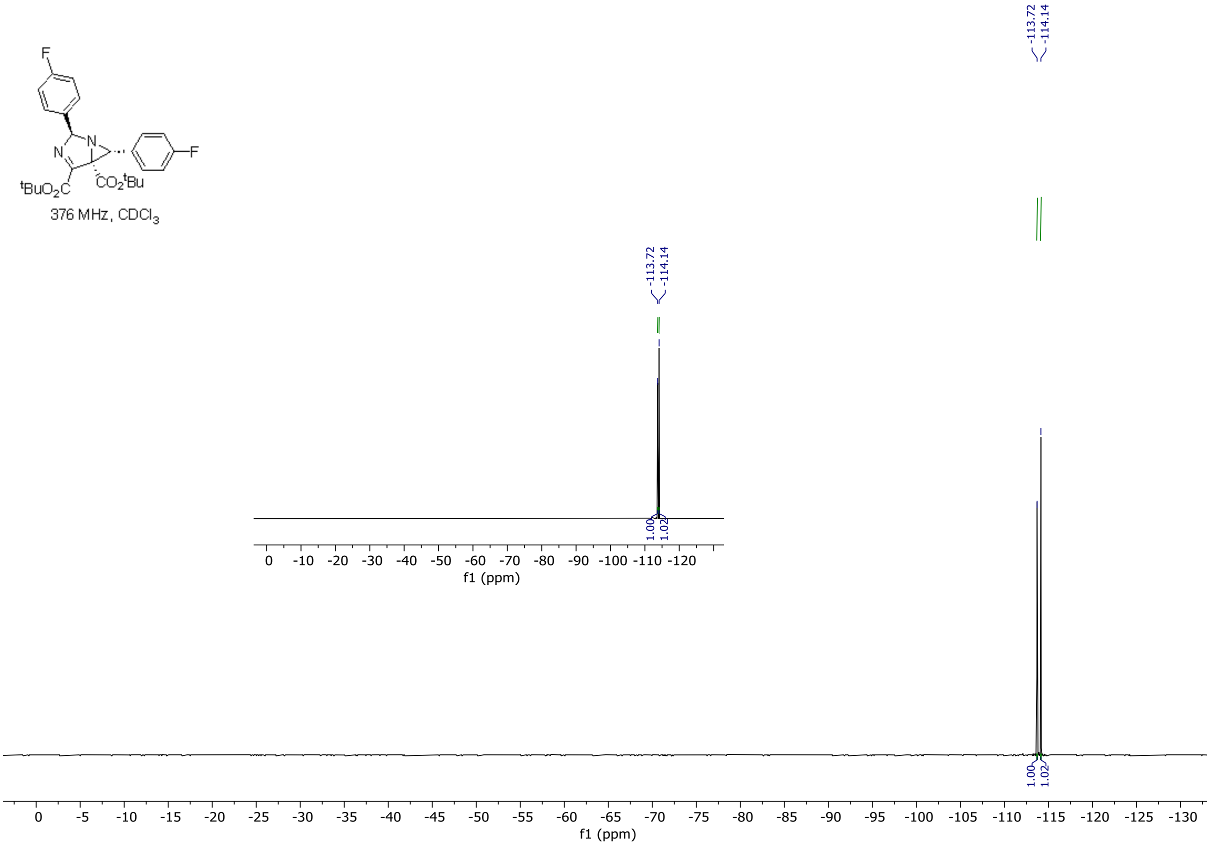

376 MHz, CDCl3
-113.72
-114.14
1.00
1.02
f1 (ppm)

(+/-)Di-tert-butyl(2R,5S,6R)-2,6-bis(4-fluorophenyl)-1,3-diazabicyclo[3.1.0]hex-3-ene-4,5-dicarboxylate (3l')

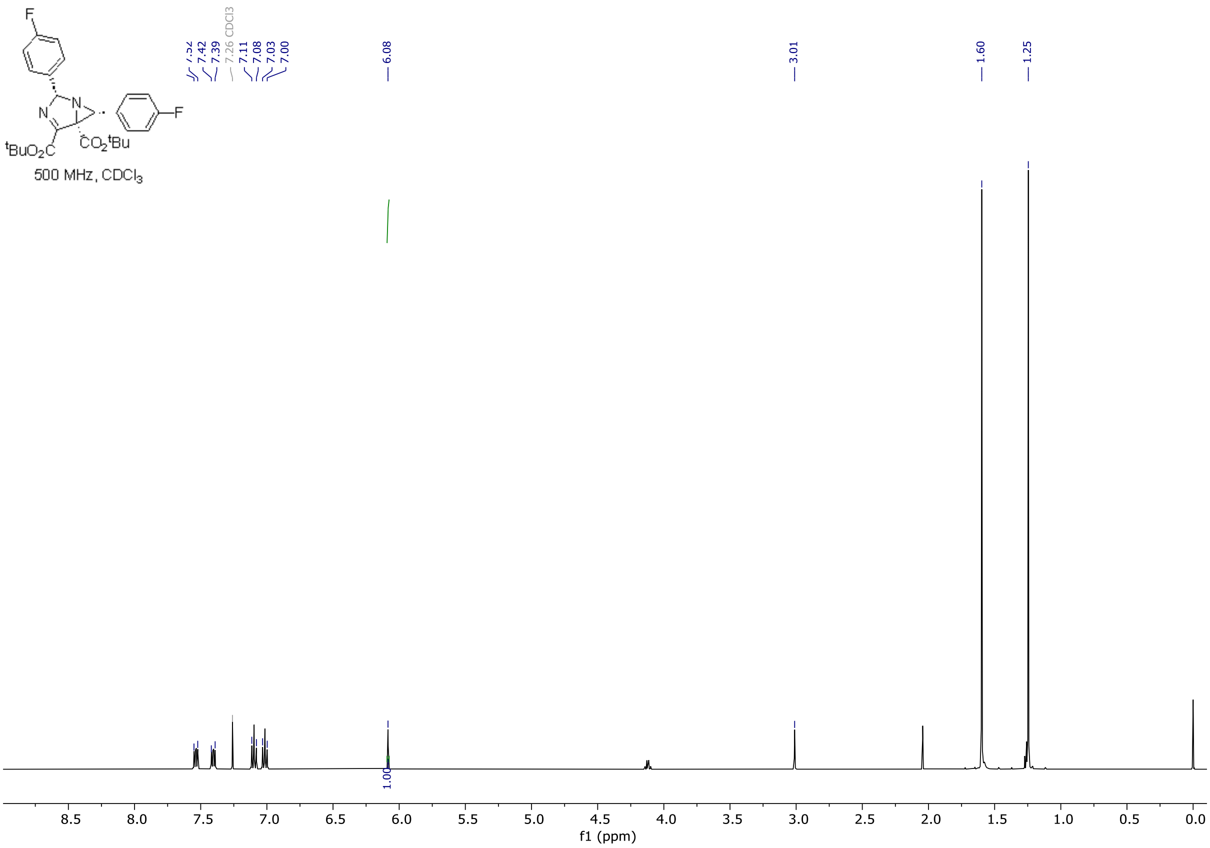

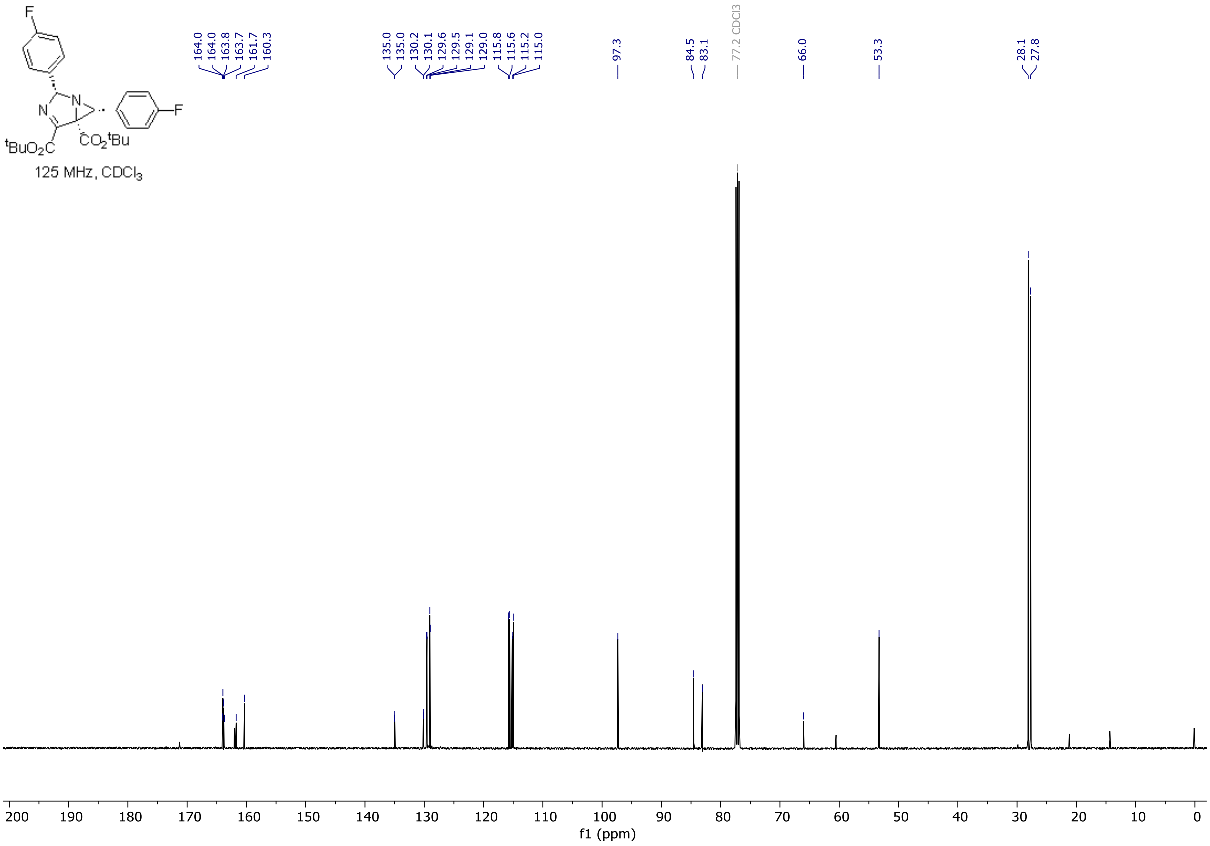

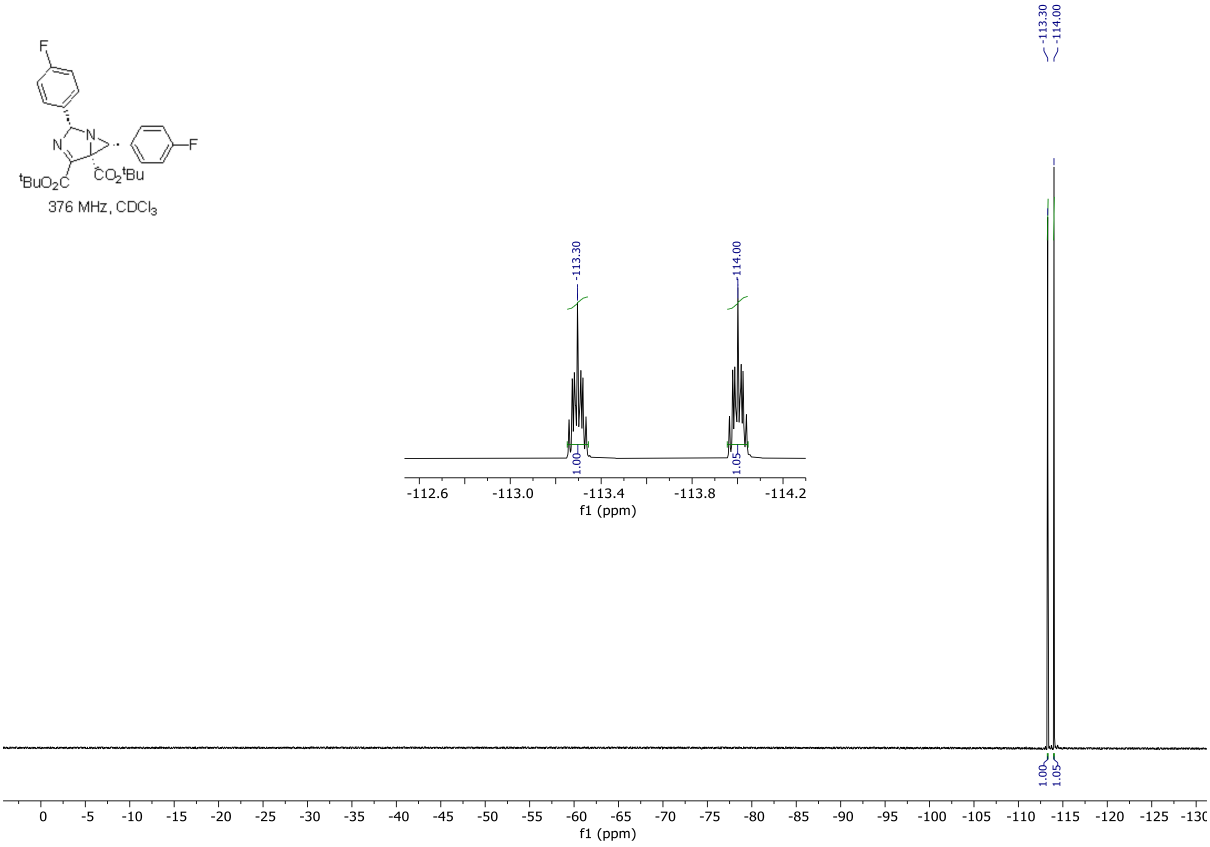

F
N
N
F
ᵗBuO₂C
CO₂ᵗBu
376 MHz, CDCl₃
-113.30
-114.00
1.00
1.05
-112.6
-113.0
-113.4
-113.8
-114.2
f1 (ppm)
0 -5 -10 -15 -20 -25 -30 -35 -40 -45 -50 -55 -60 -65 -70 -75 -80 -85 -90 -95 -100 -105 -110 -115 -120 -125 -130
f1 (ppm)

(+/-)Di-tert-butyl(2S,5S,6R)-2,6-bis(4-methoxyphenyl)-1,3-diazabicyclo[3.1.0]hex-3-ene-4,5-dicarboxylate (3m)

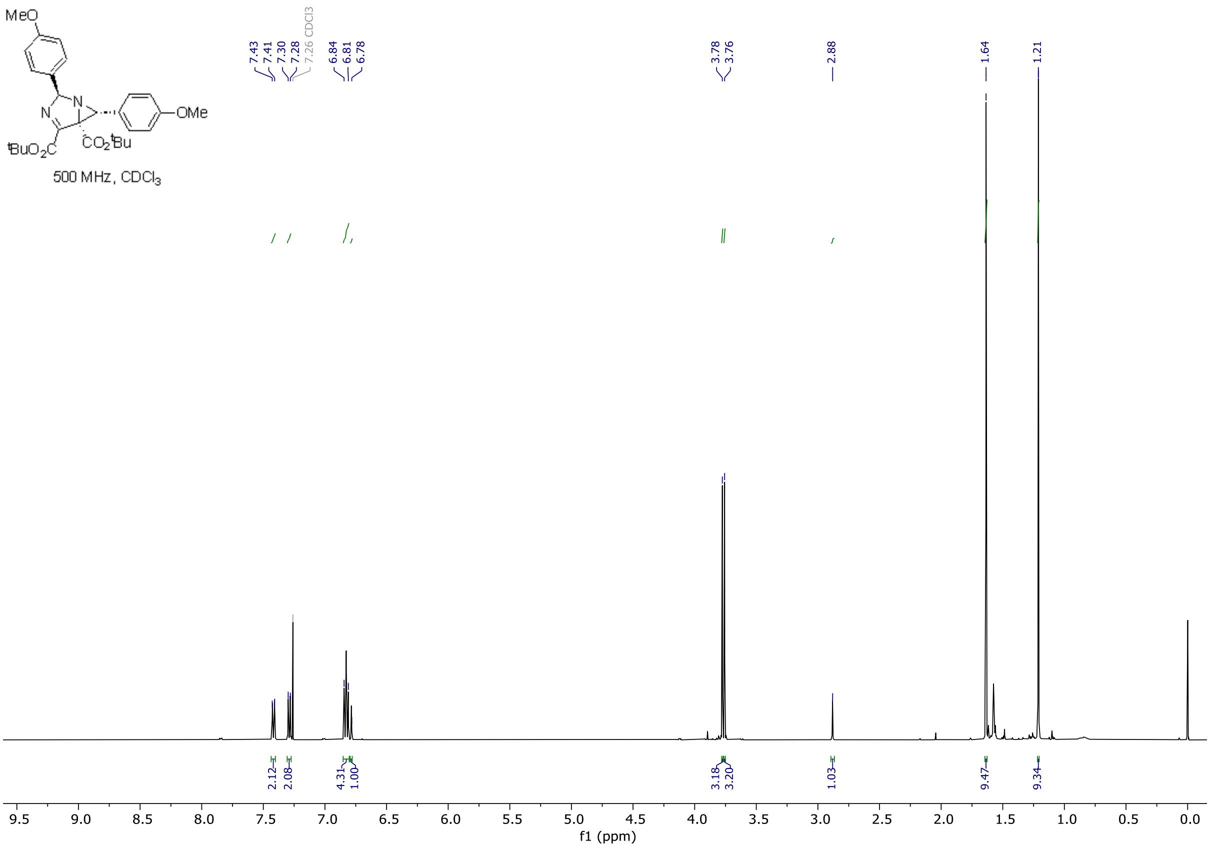

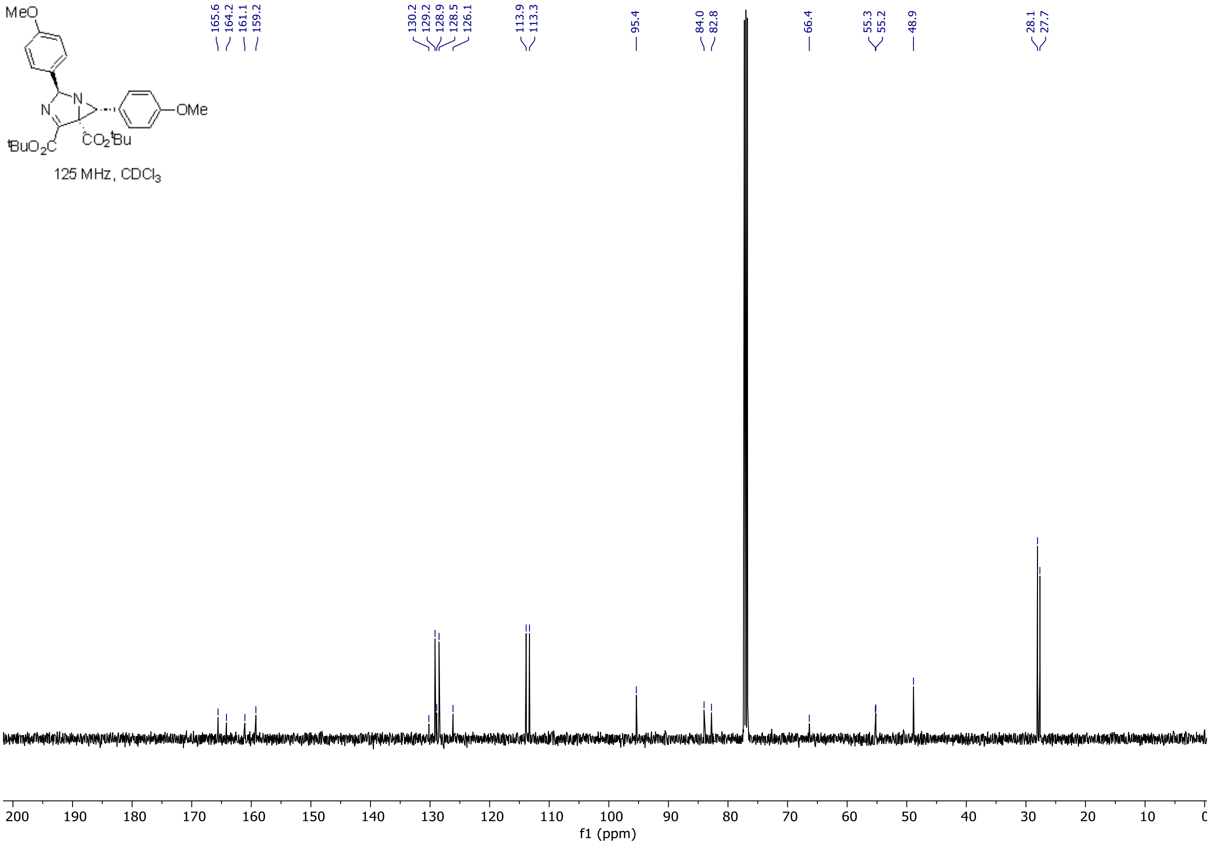

(+/-)Di-tert-butyl(2R,5S,6R)-2,6-bis(4-methoxyphenyl)-1,3-diazabicyclo[3.1.0]hex-3-ene-4,5-dicarboxylate (3m')

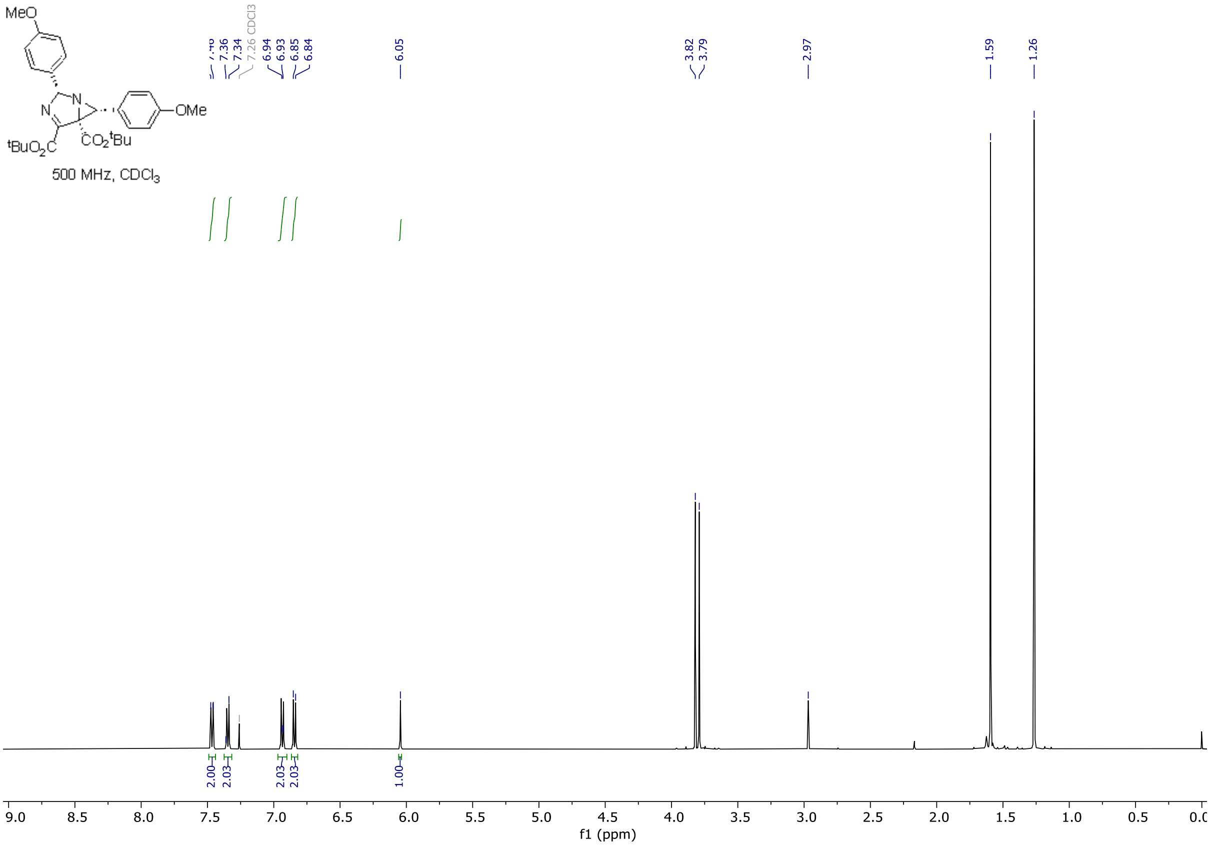

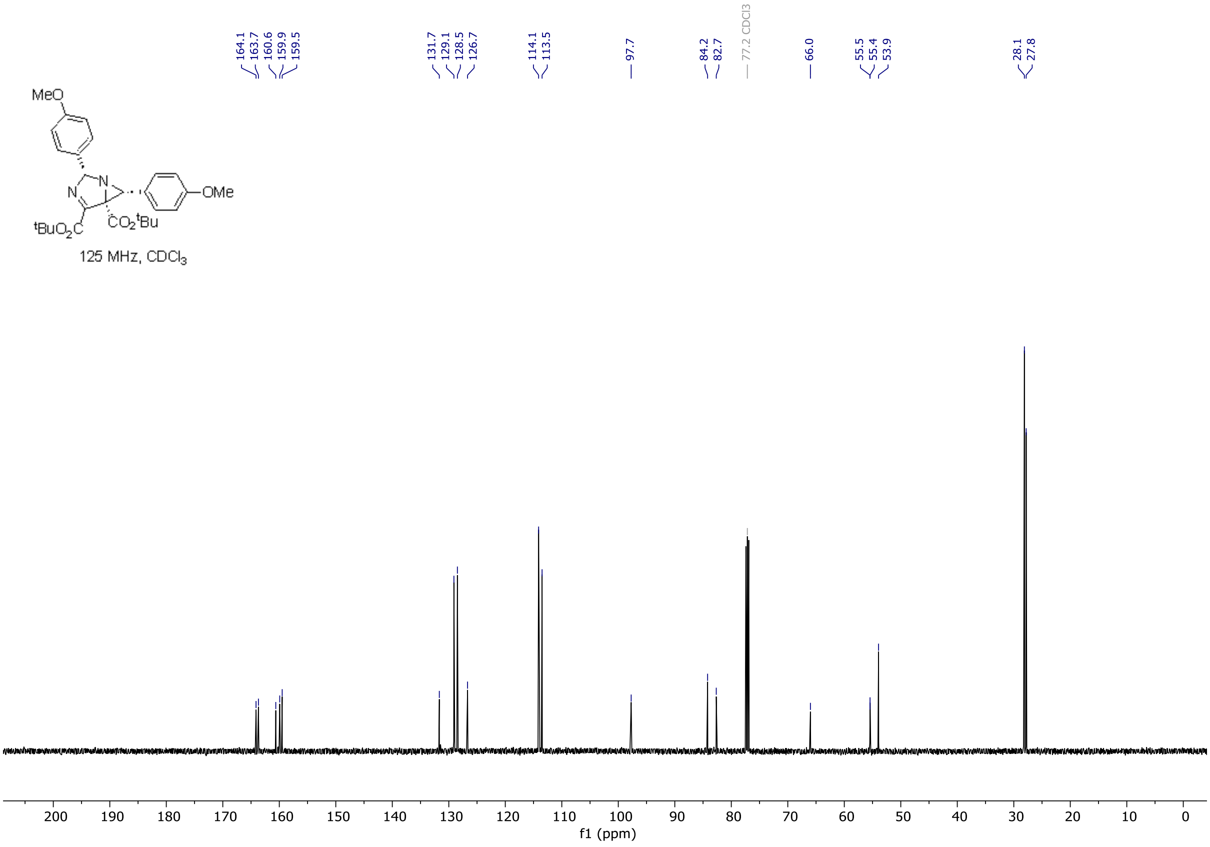

(+/-)Di-tert-pentyl (2S,5S,6R)-2,6-bis(4-fluorophenyl)-1,3-diazabicyclo[3.1.0]hex-3-ene-4,5-dicarboxylate (3n)

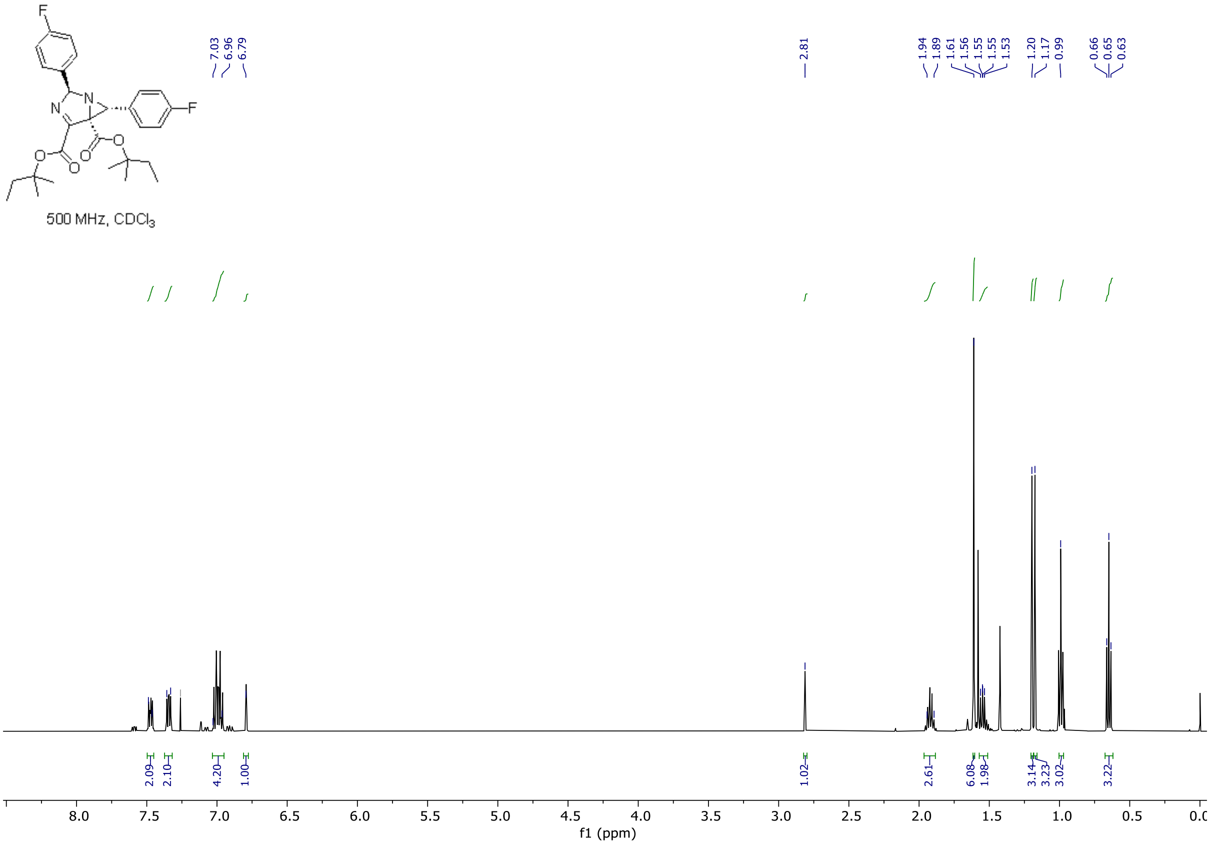

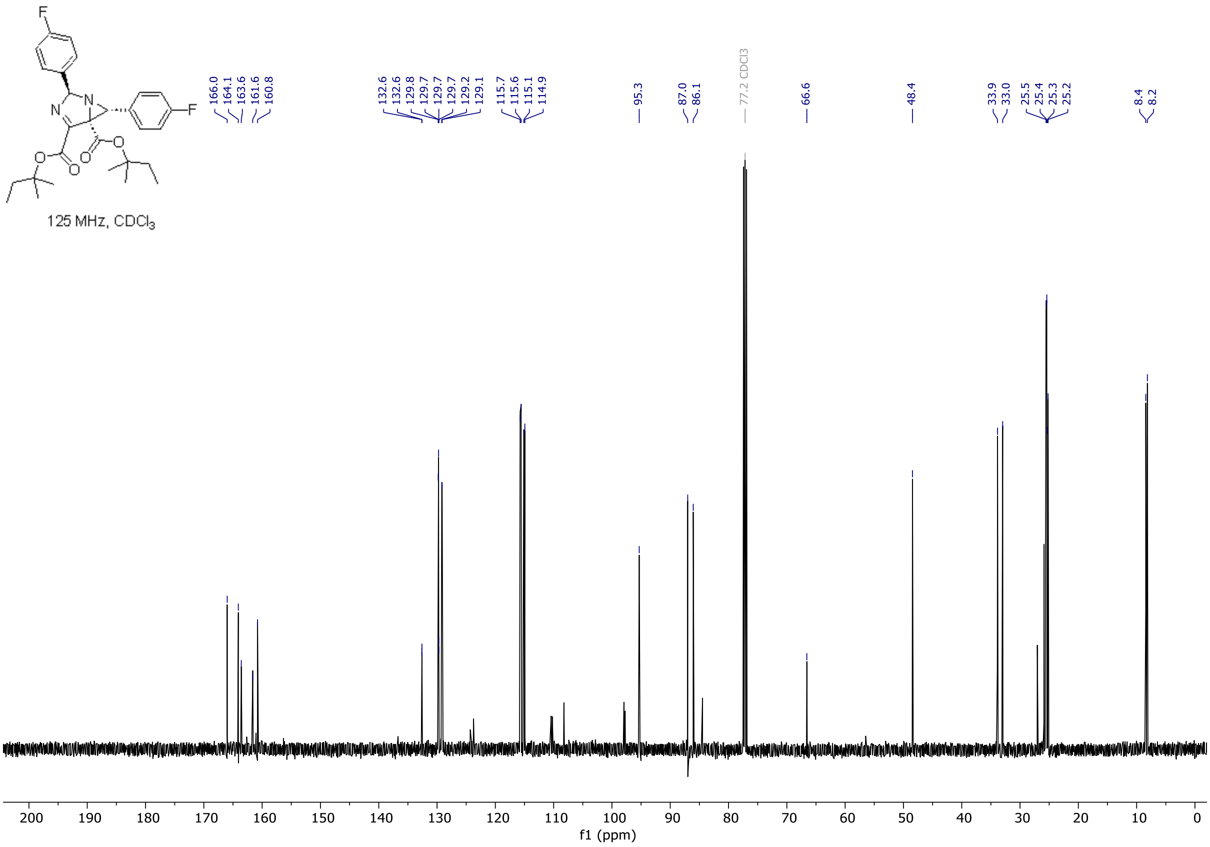

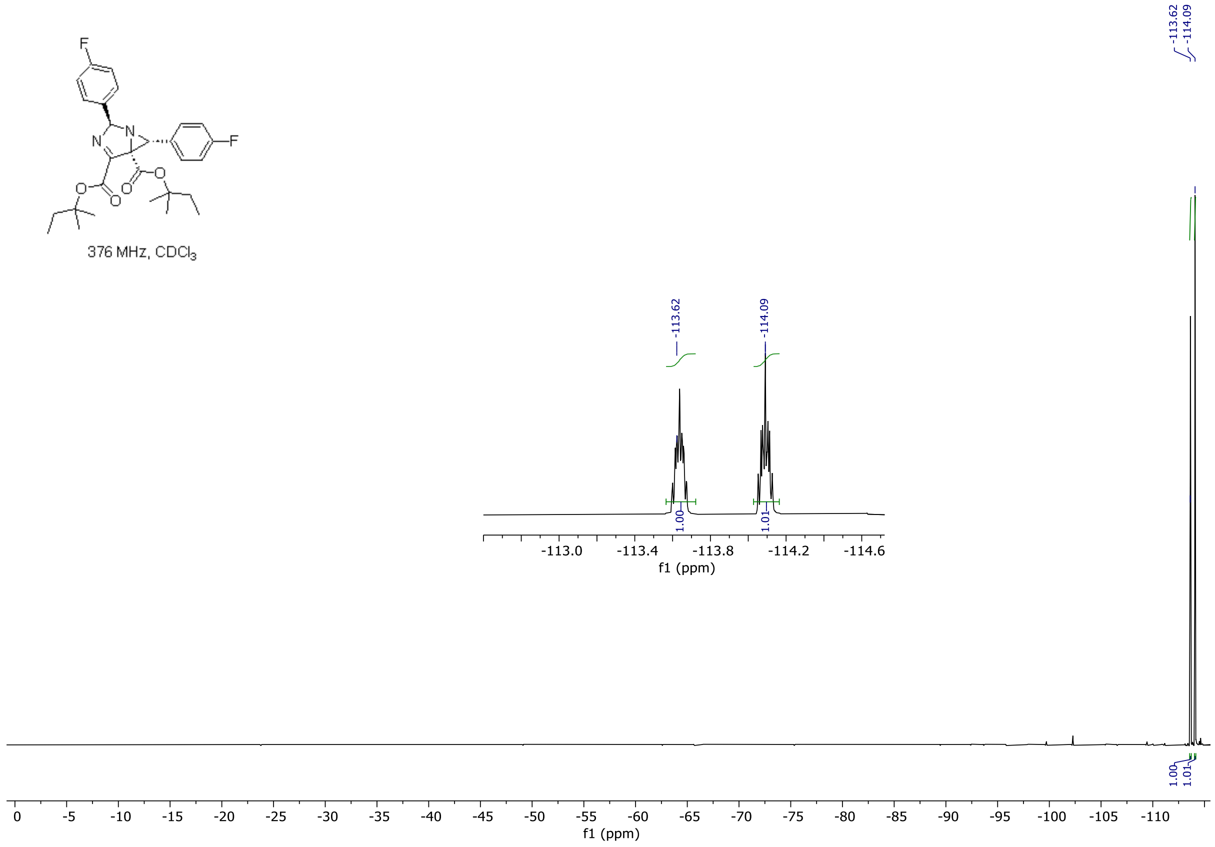
376 MHz, CDCl3
-113.62
-114.09
1.00
1.01
-113.0
-113.4
-113.8
-114.2
-114.6
f1 (ppm)

(+/-)Di-tert-pentyl(2R,5S,6R)-2,6-bis(4-fluorophenyl)-1,3-diazabicyclo[3.1.0]hex-3-ene-4,5-dicarboxylate (3n')

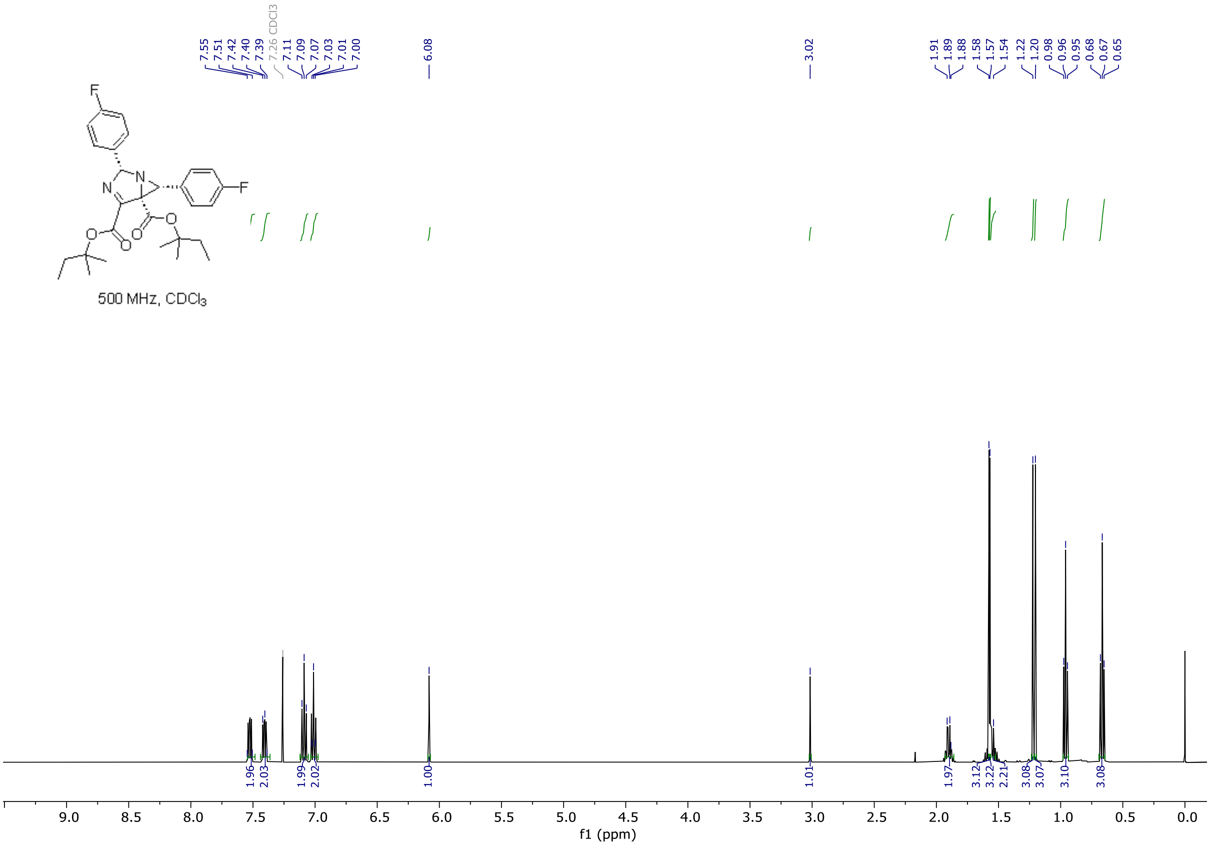

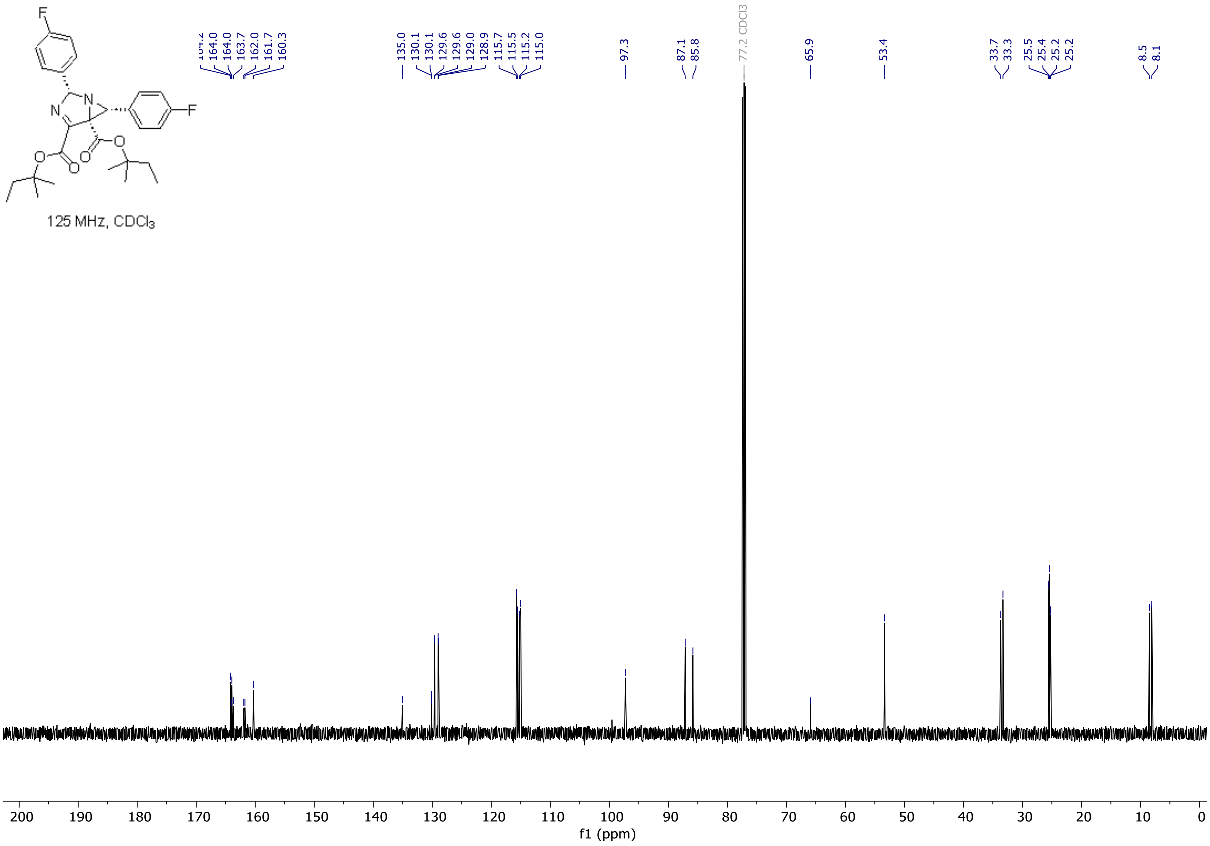

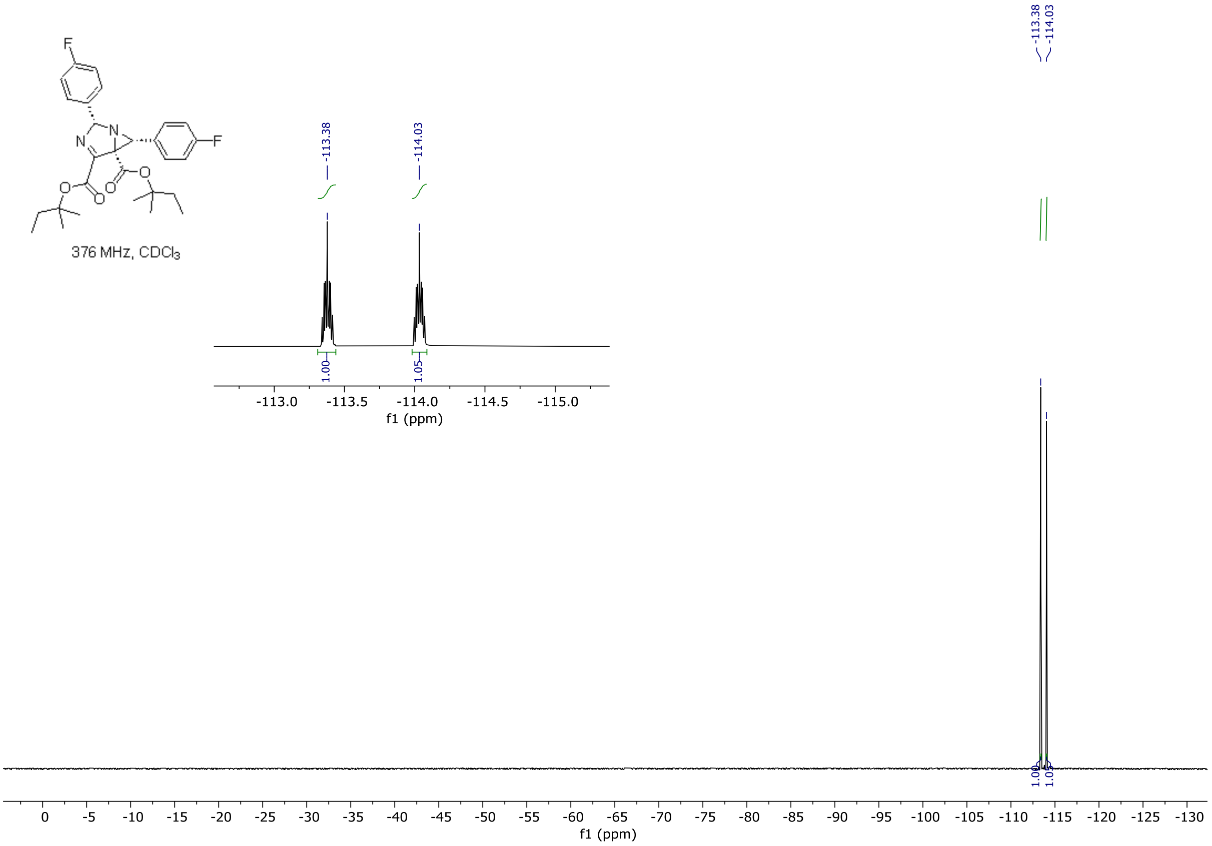

376 MHz, CDCl3
-113.38
-114.03
1.00
1.05
-113.0
-113.5
-114.0
-114.5
-115.0
f1 (ppm)
0
-5
-10
-15
-20
-25
-30
-35
-40
-45
-50
-55
-60
-65
-70
-75
-80
-85
-90
-95
-100
-105
-110
-115
-120
-125
-130
f1 (ppm)

(+/-)Methyl(2S,5S,6R)-2-(4-fluorophenyl)-5,6-diphenyl-1,3-diazabicyclo[3.1.0]hex-3-ene-4-carboxylate (5a)

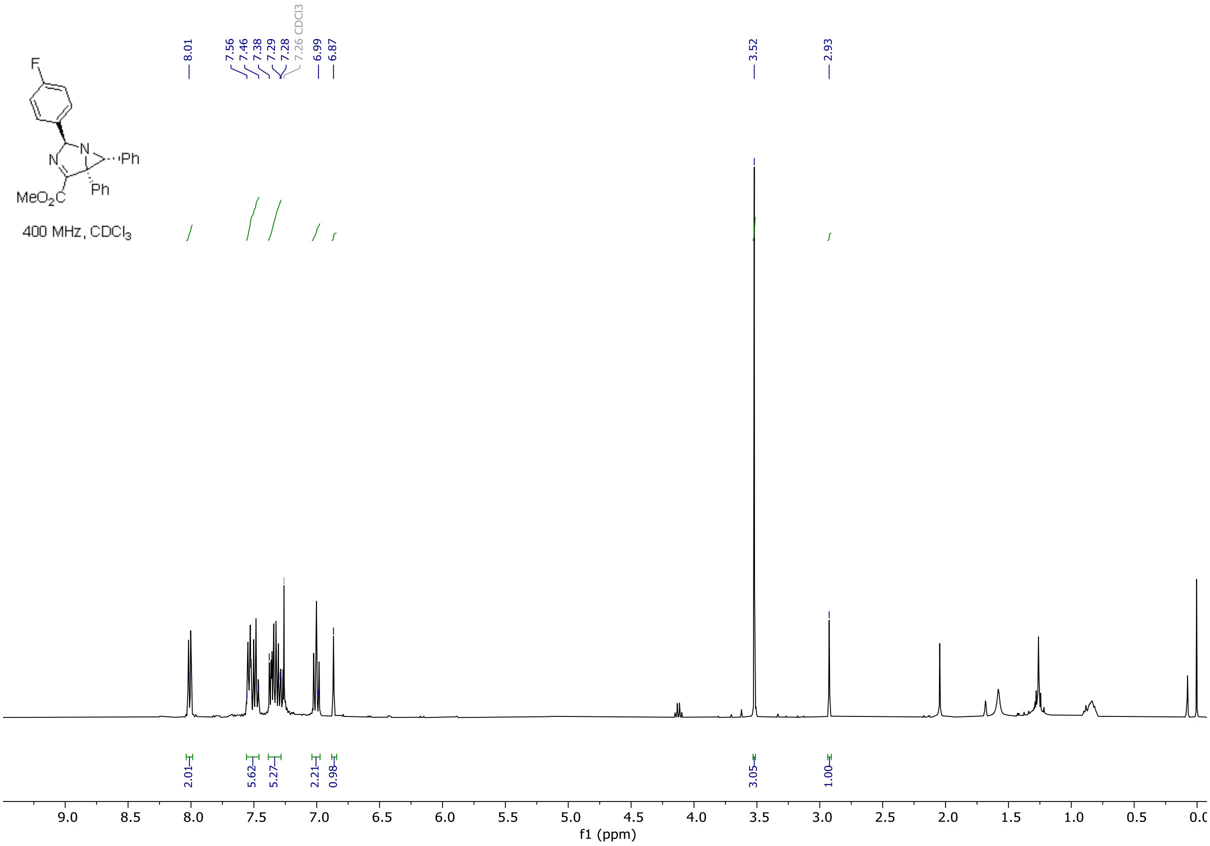

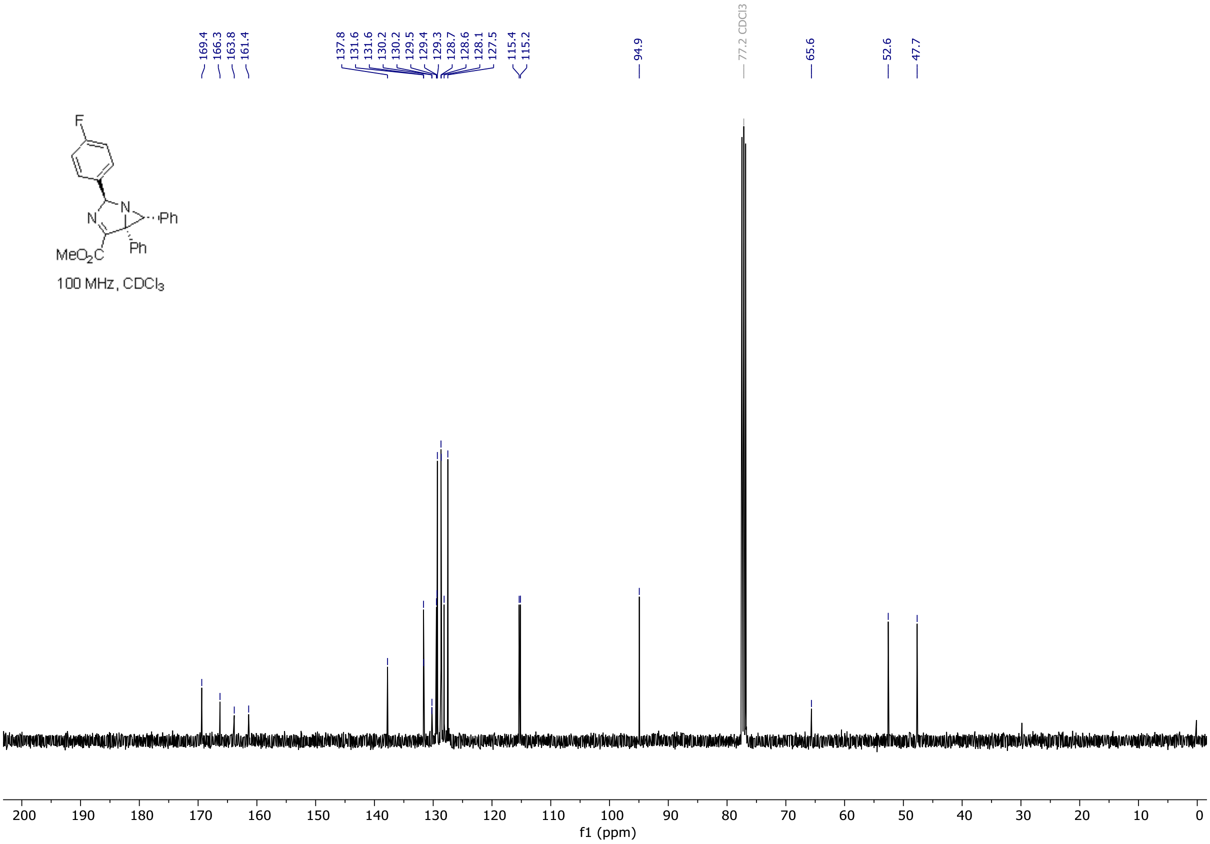

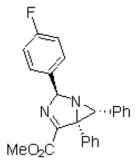

376 MHz, $CDCl_3$

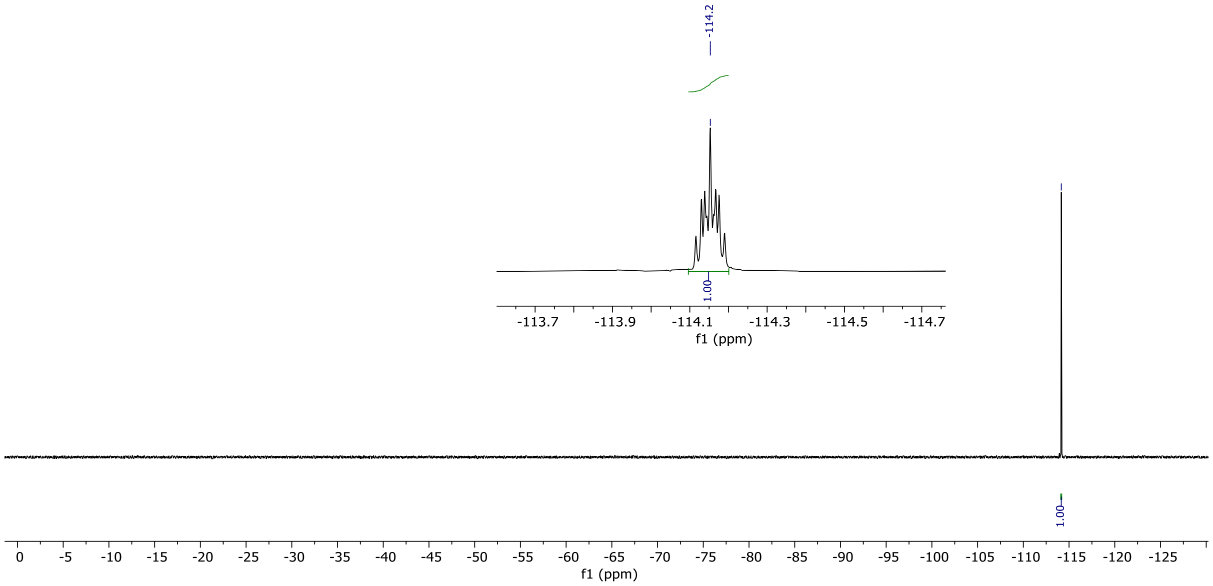

(+/-)Methyl2R,5S,6R)-2-(4-fluorophenyl)-5,6-diphenyl-1,3-diazabicyclo[3.1.0]hex-3-ene-4-carboxylate (5a')

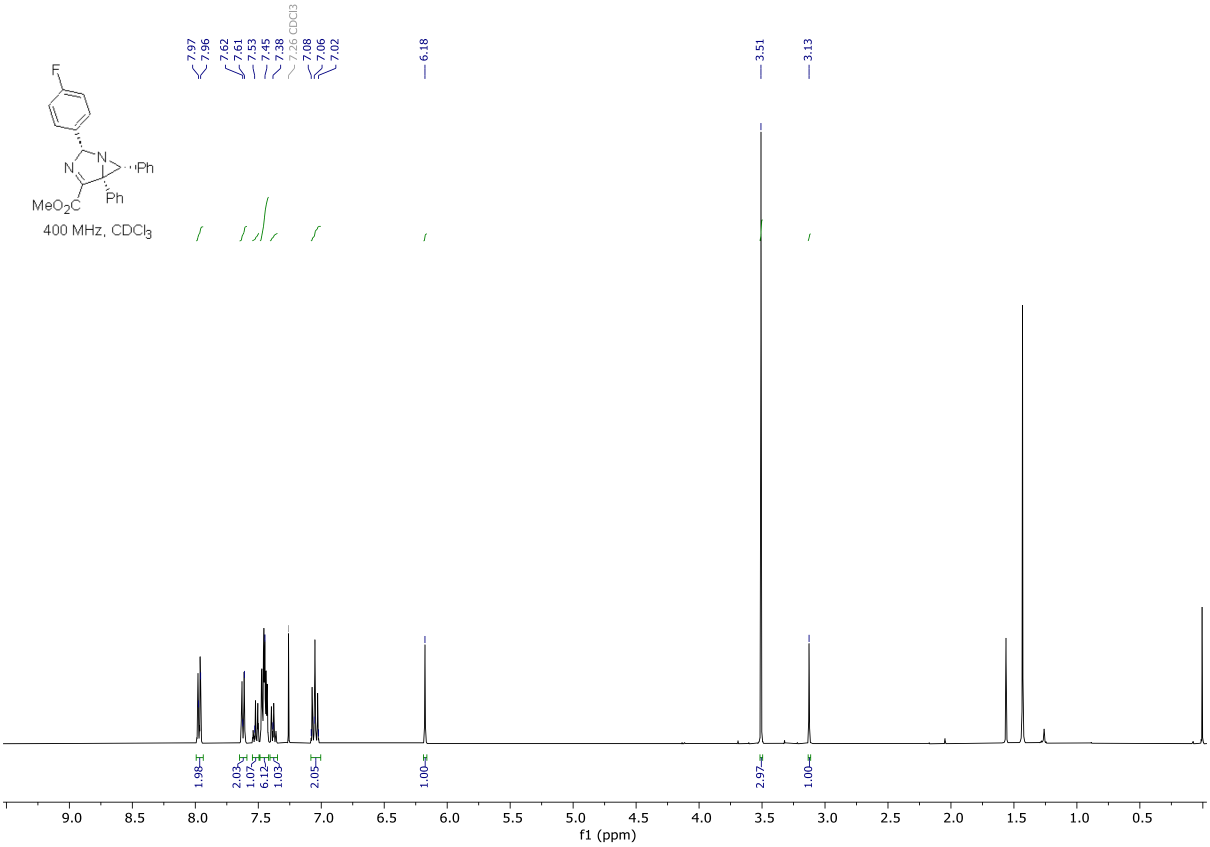

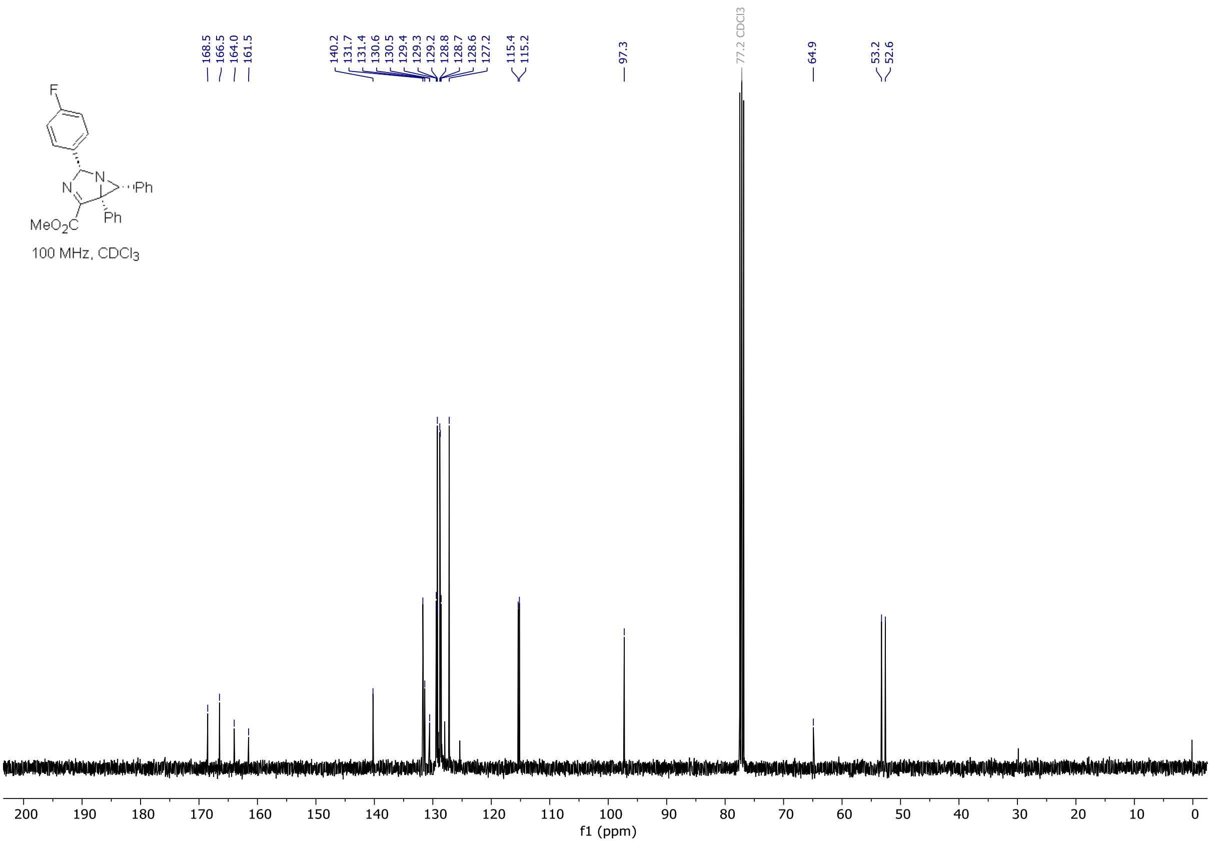

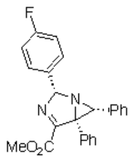

376 MHz, $CDCl_3$

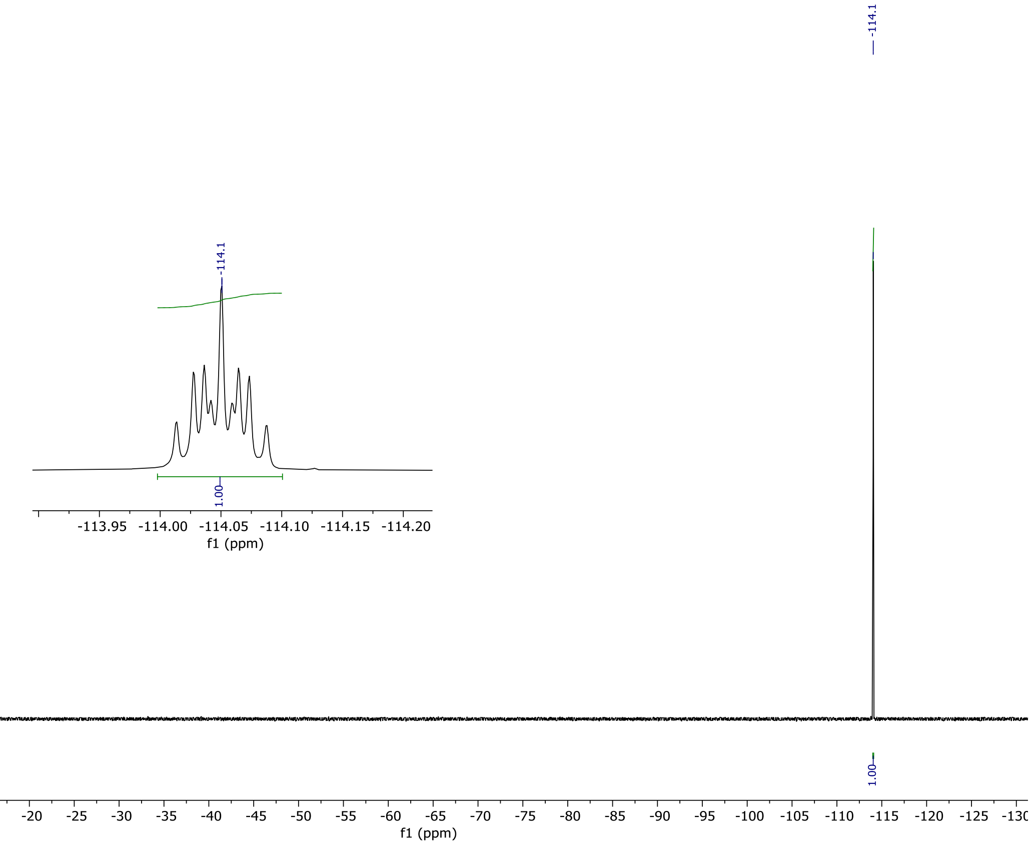

(+/-)Methyl(2S,5S,6R)-5,6-diphenyl-2-(p-tolyl)-1,3-diazabicyclo[3.1.0]hex-3-ene-4-carboxylate (5b)

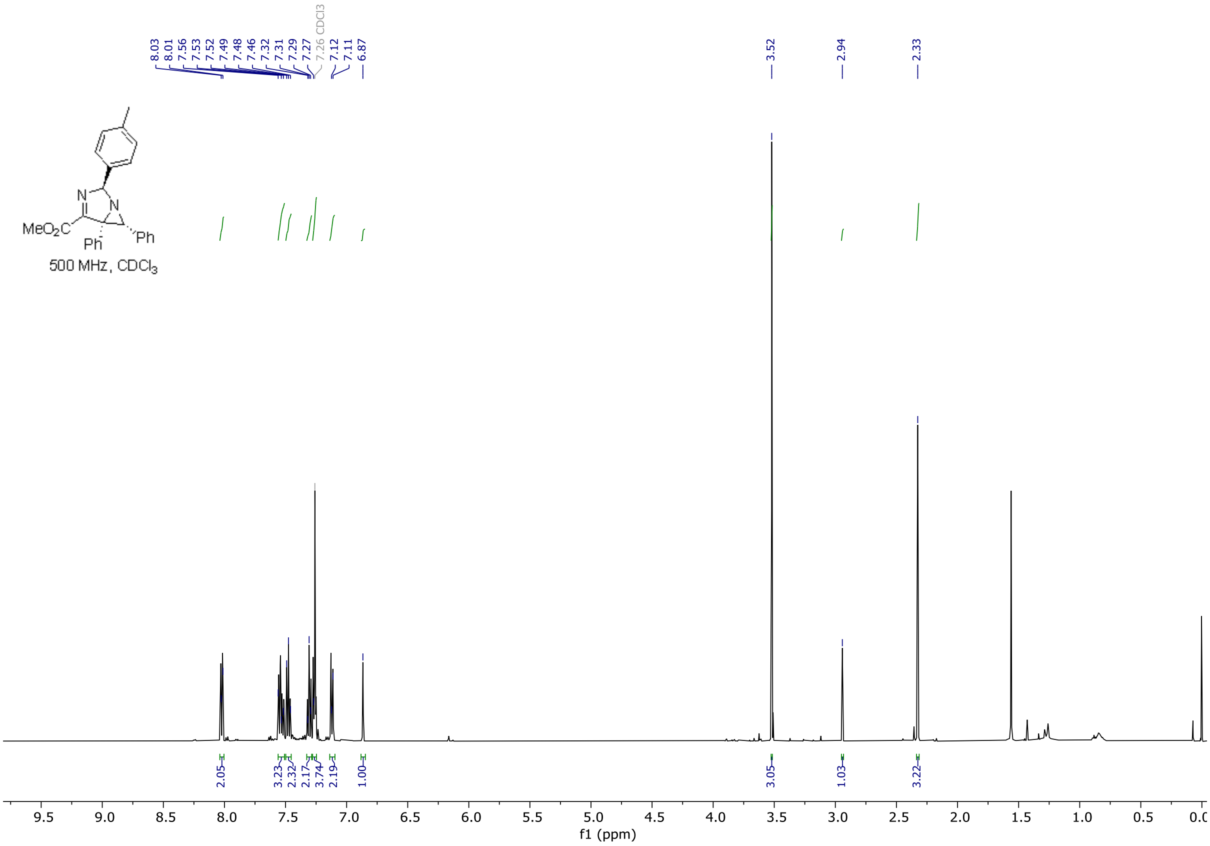

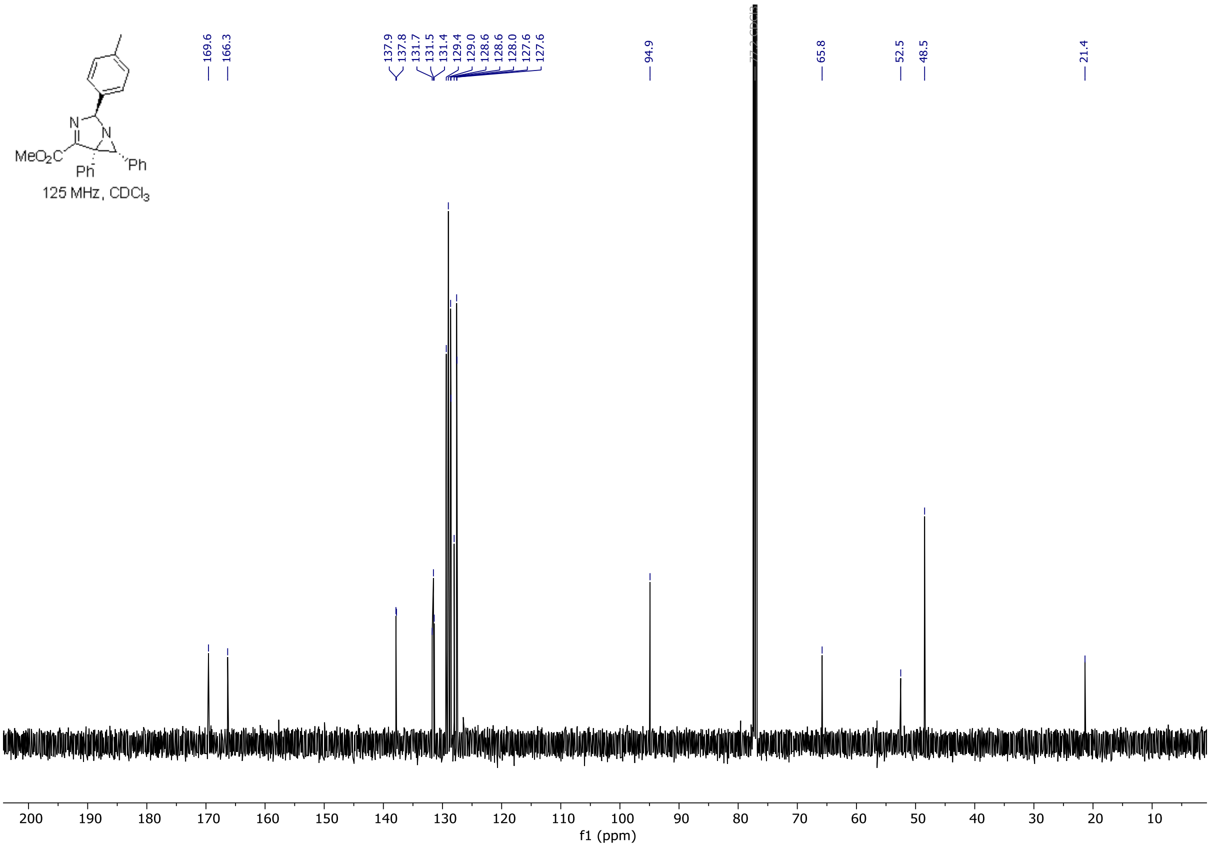

(+/-)Methyl(2R,5S,6R)-5,6-diphenyl-2-(p-tolyl)-1,3-diazabicyclo[3.1.0]hex-3-ene-4-carboxylate (5b’)

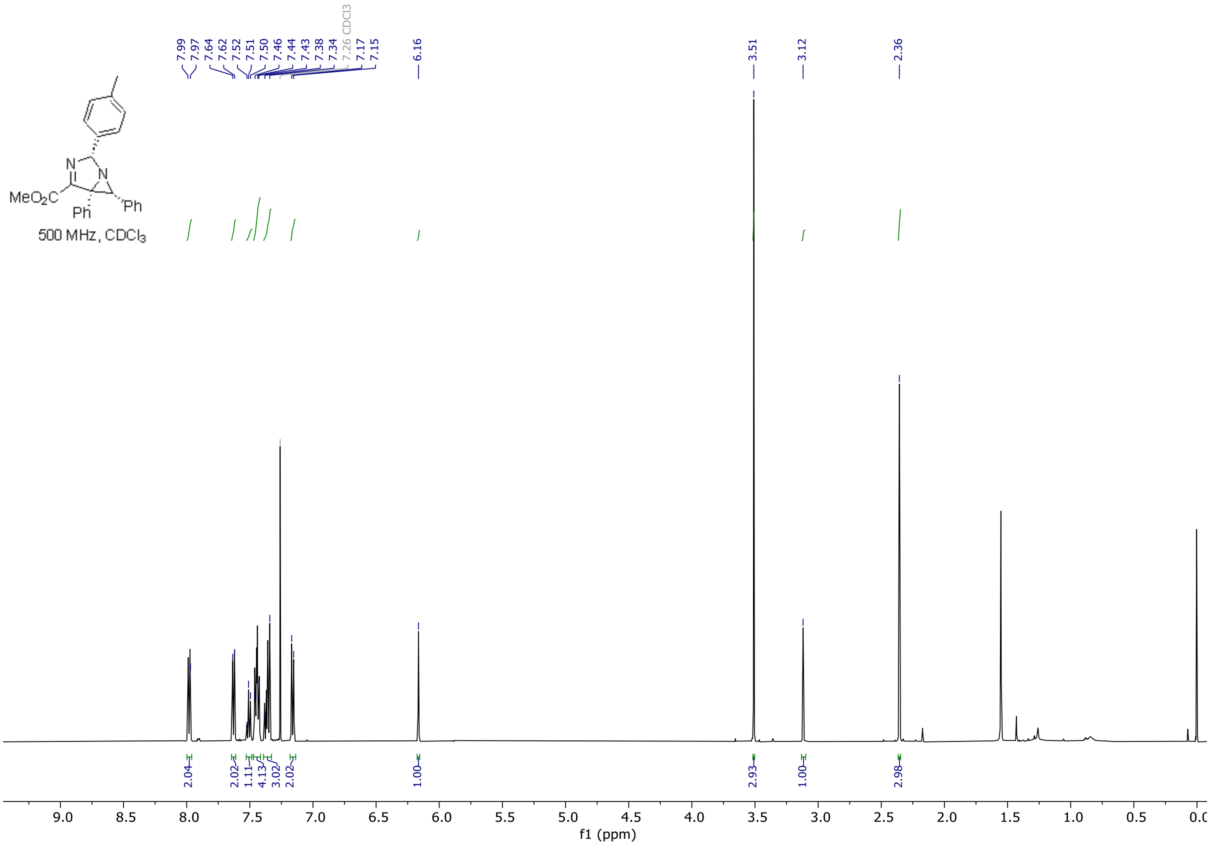

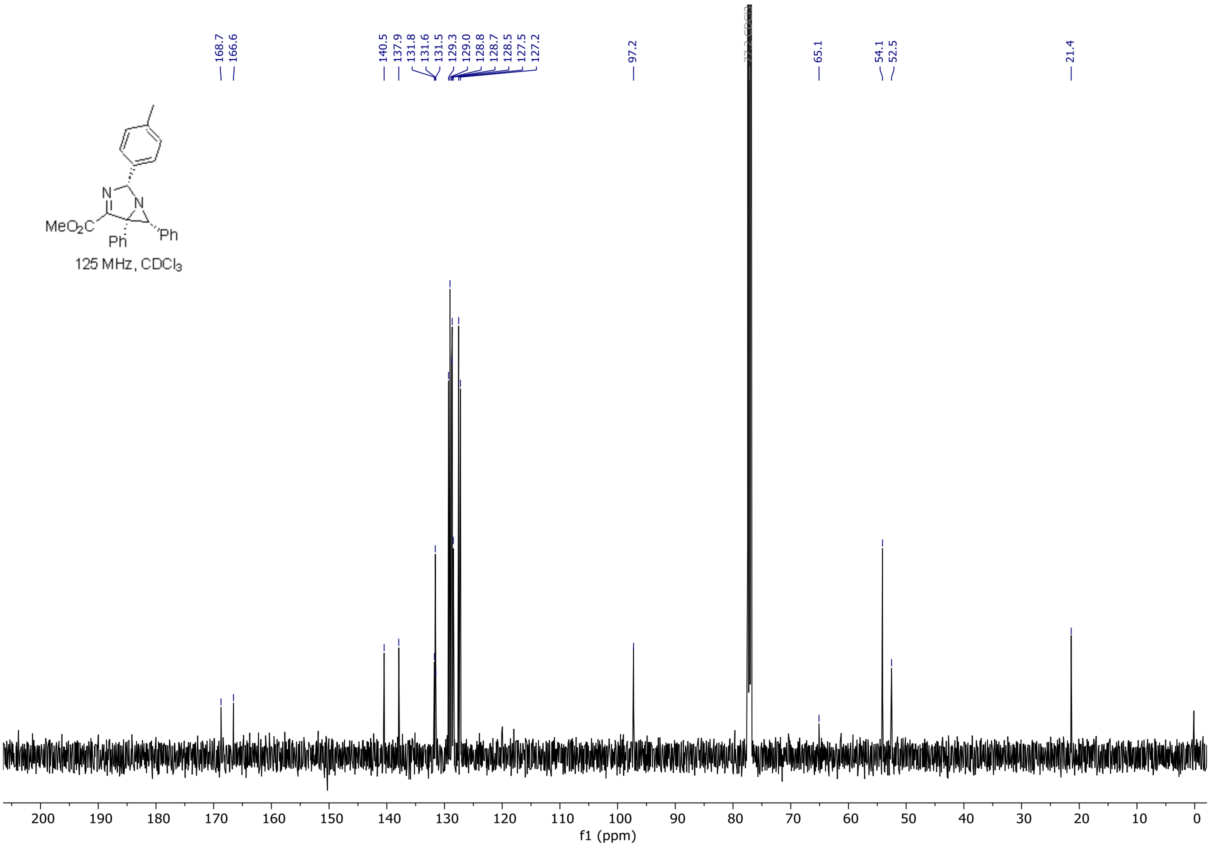

(+/-) (2R,5S,6R)-2,4,5,6-tetraphenyl-1,3-diazabicyclo[3.1.0]hex-3-ene (6)

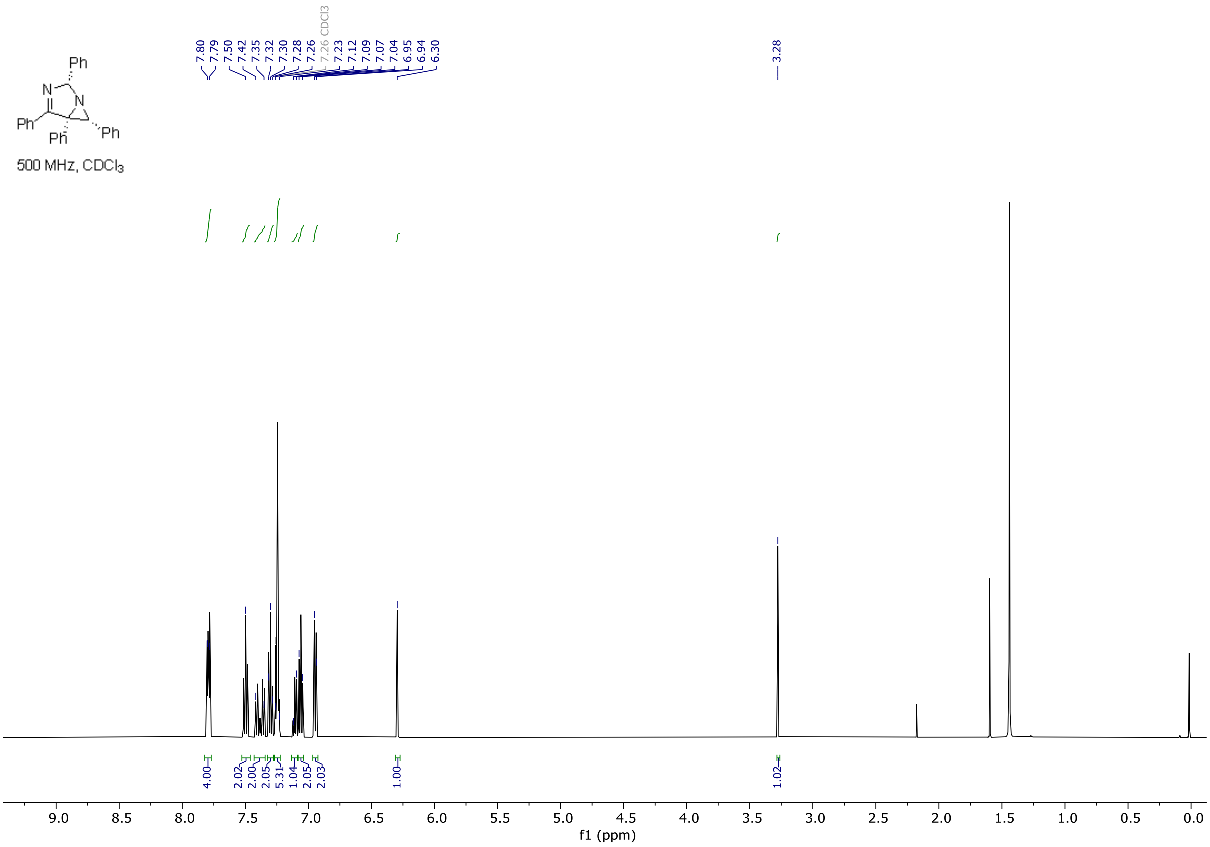

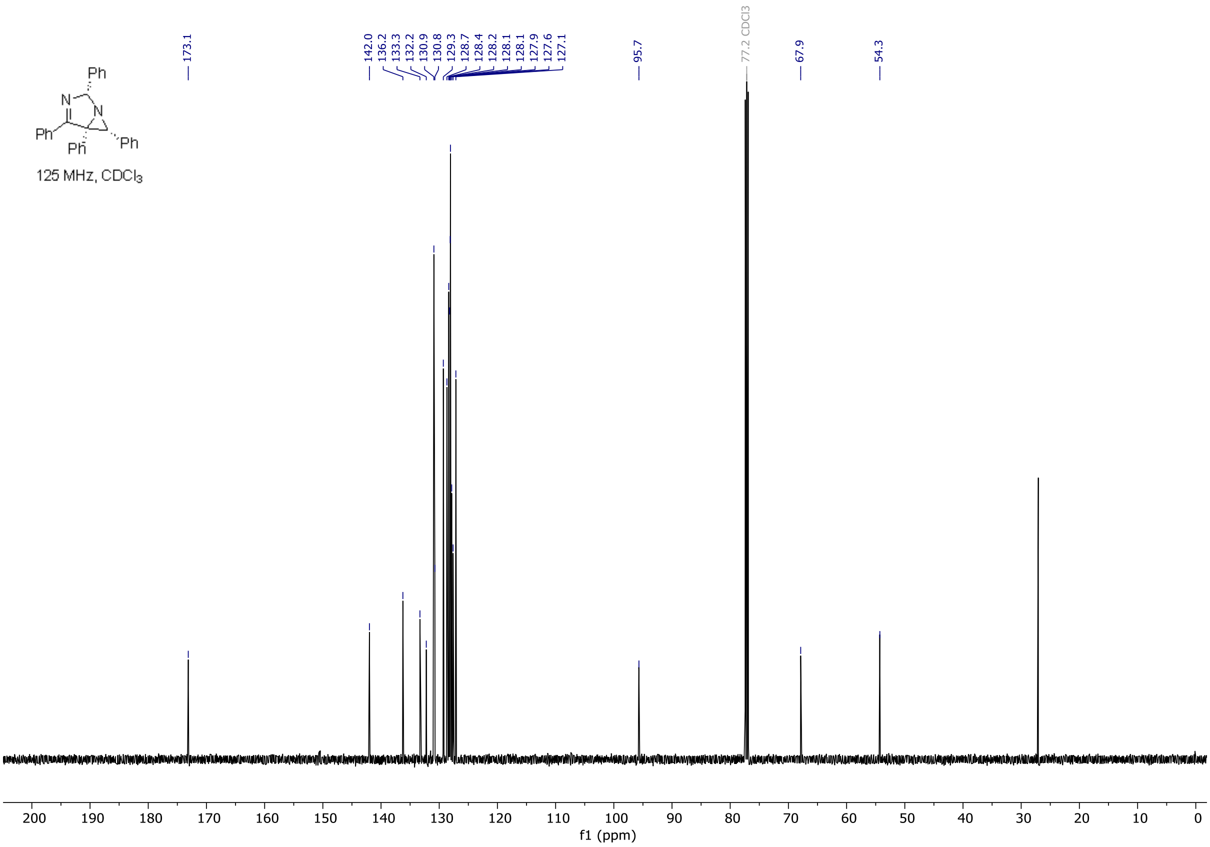

Dimethyl 2,6-diphenyl-1,6-dihydropyrimidine-4,5-dicarboxylate (6a)

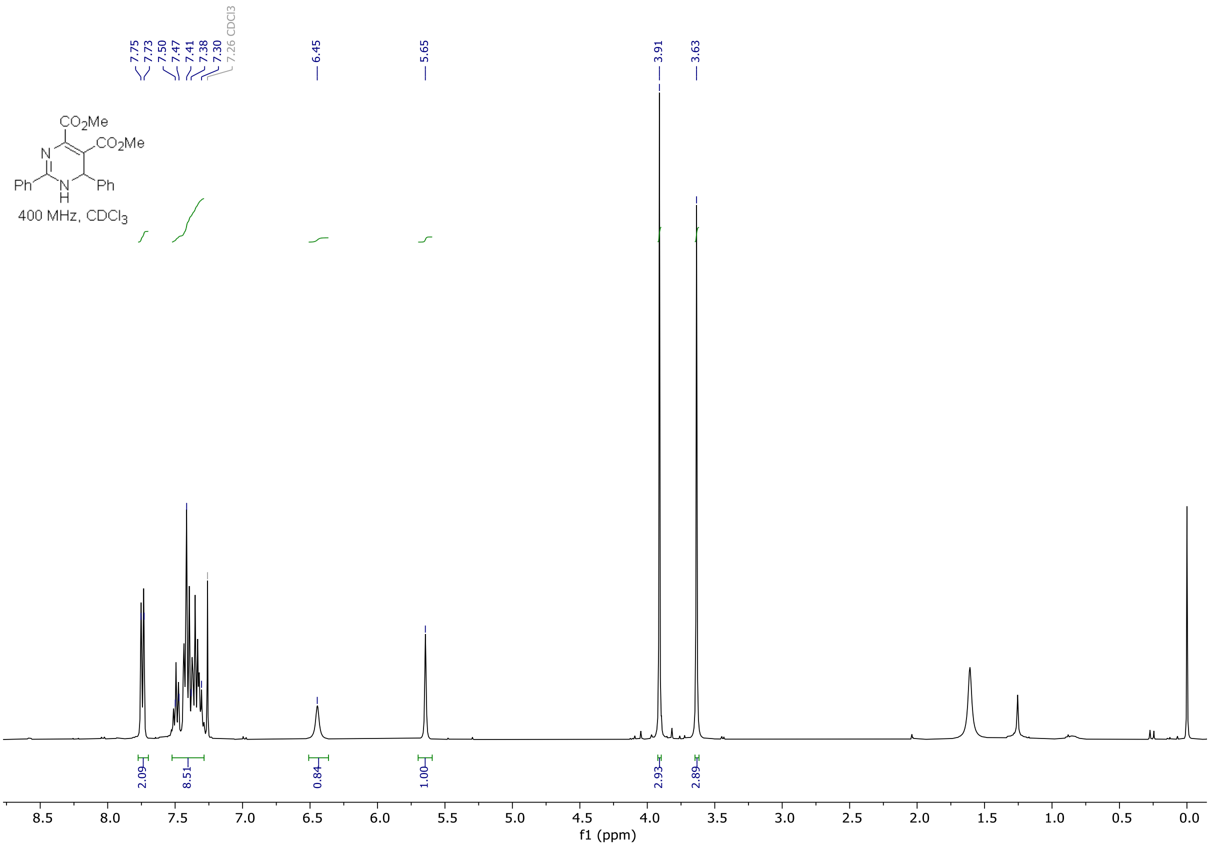

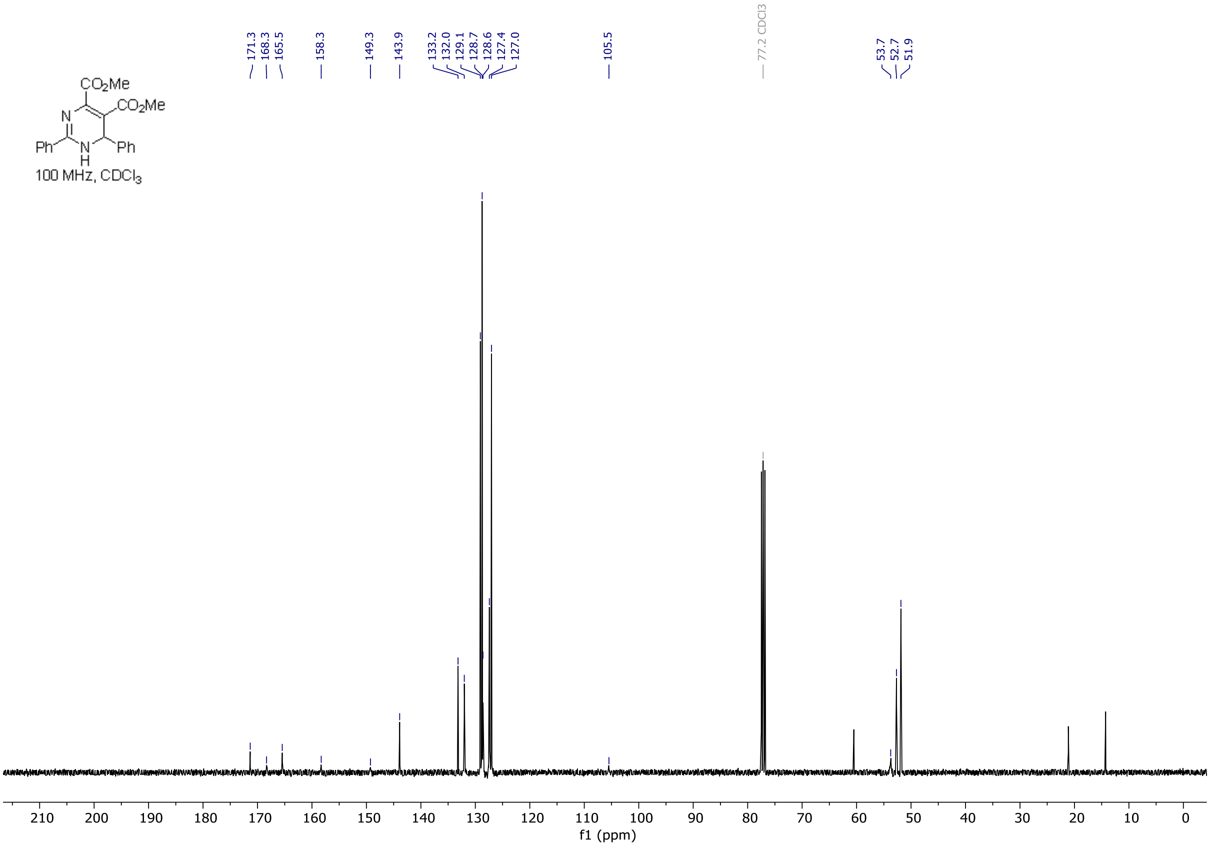

Dimethyl 2,6-bis(4-fluorophenyl)-1,6-dihydropyrimidine-4,5-dicarboxylate (6b)

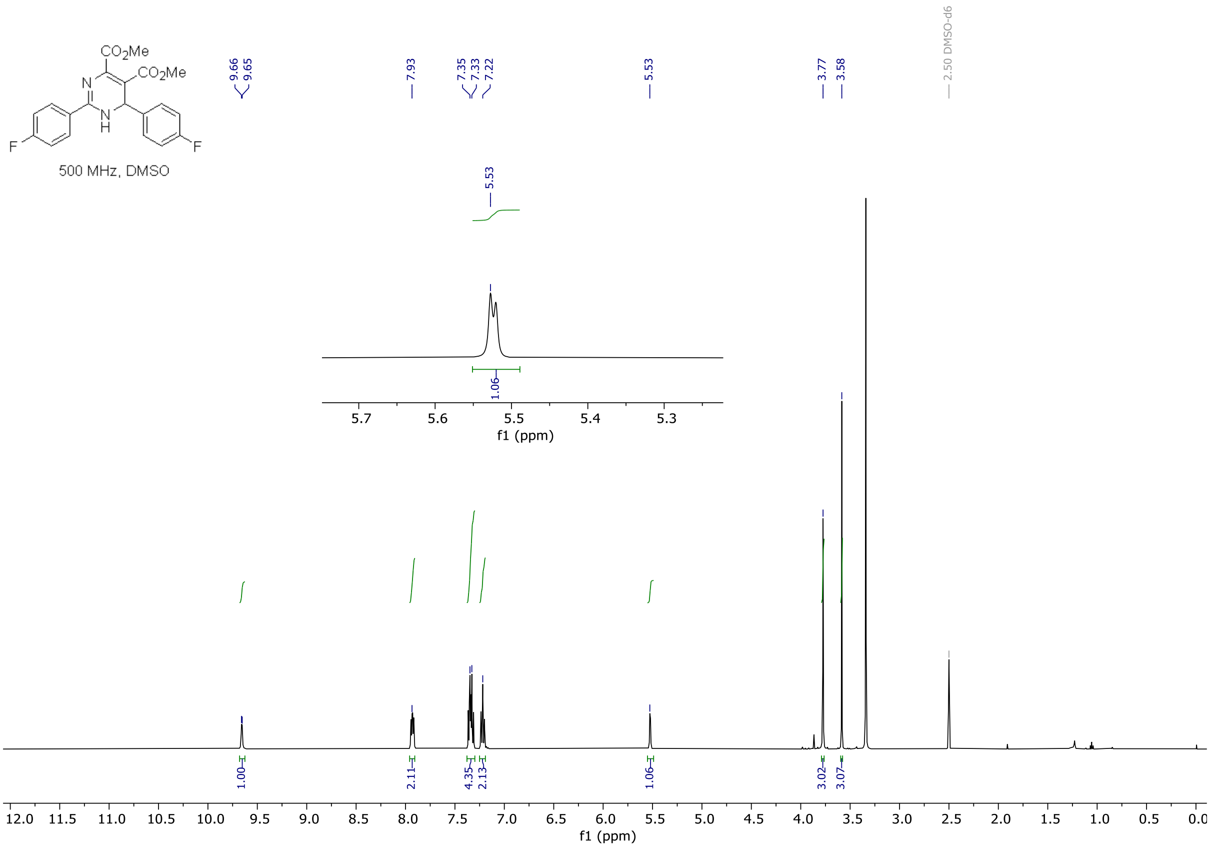

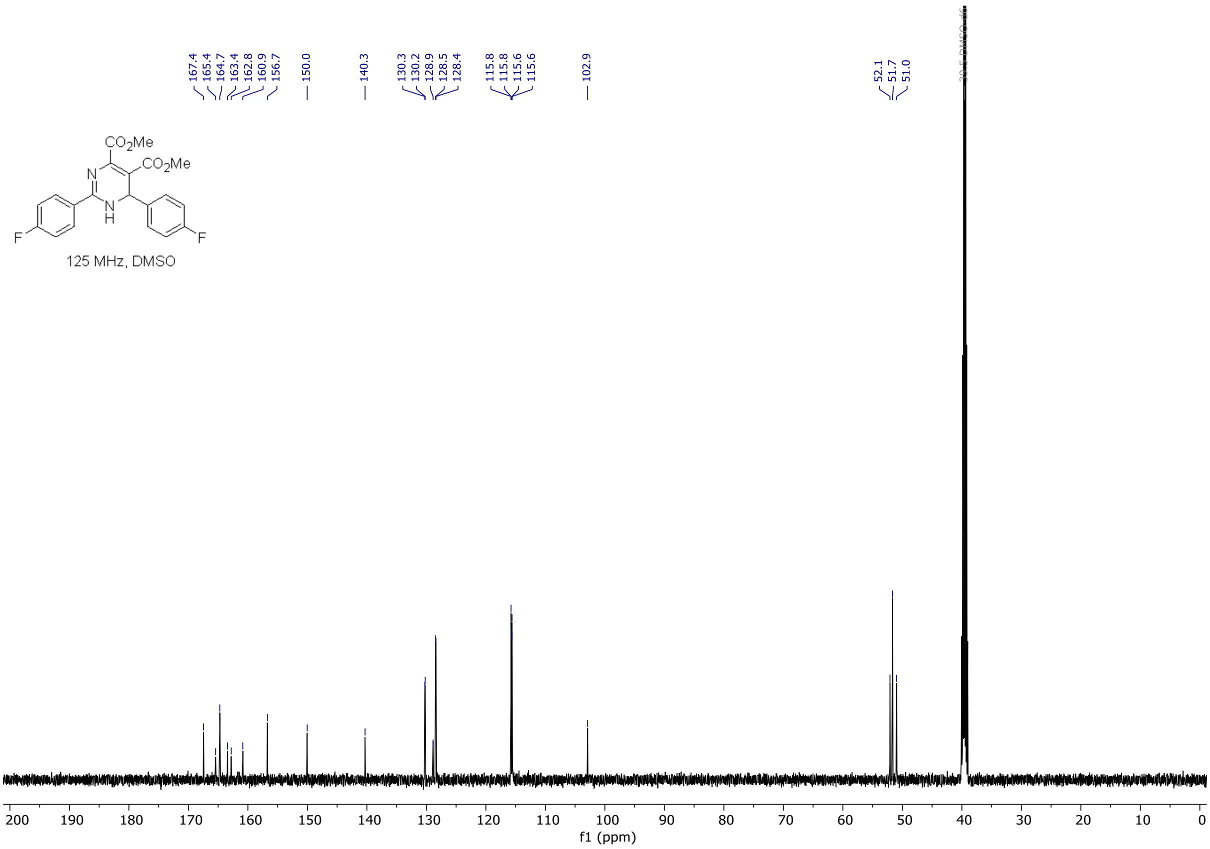

Dimethyl 2,6-bis(4-bromophenyl)-1,6-dihydropyrimidine-4,5-dicarboxylate (6c)

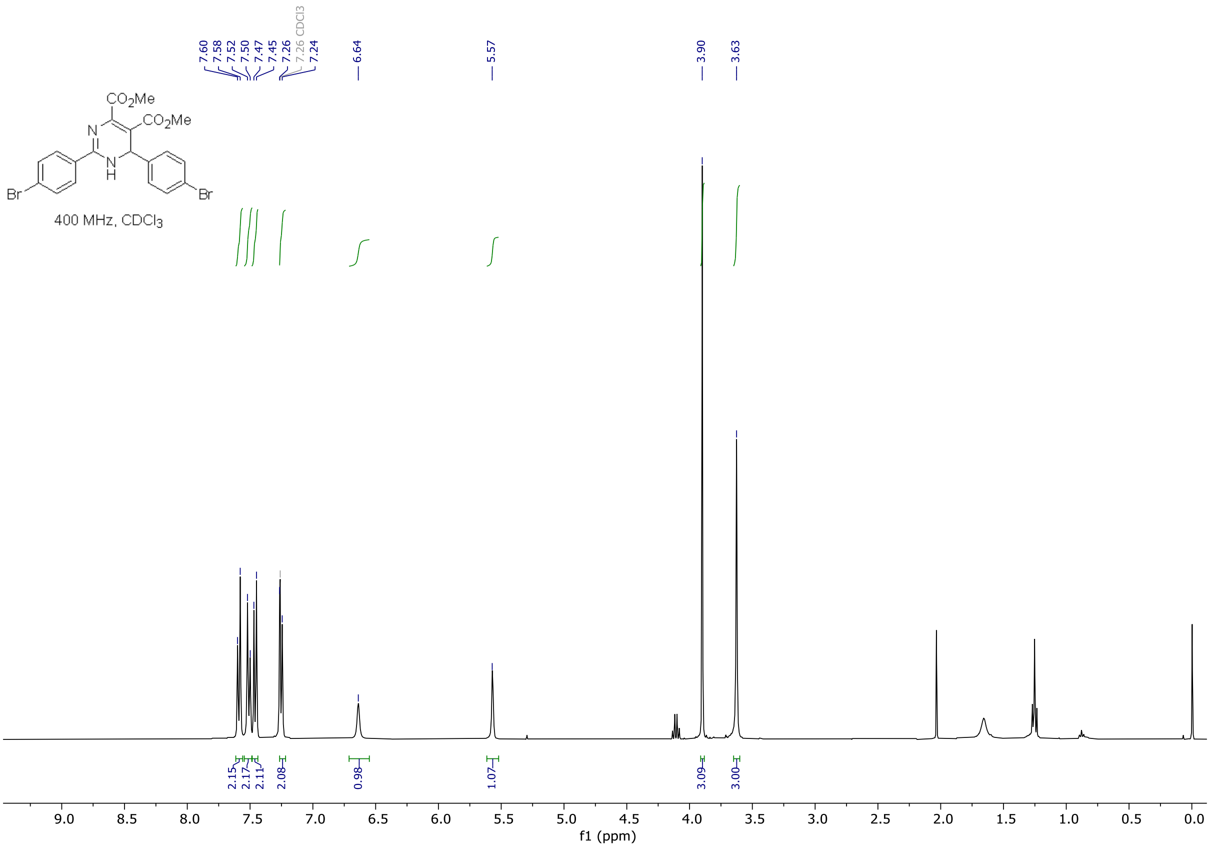

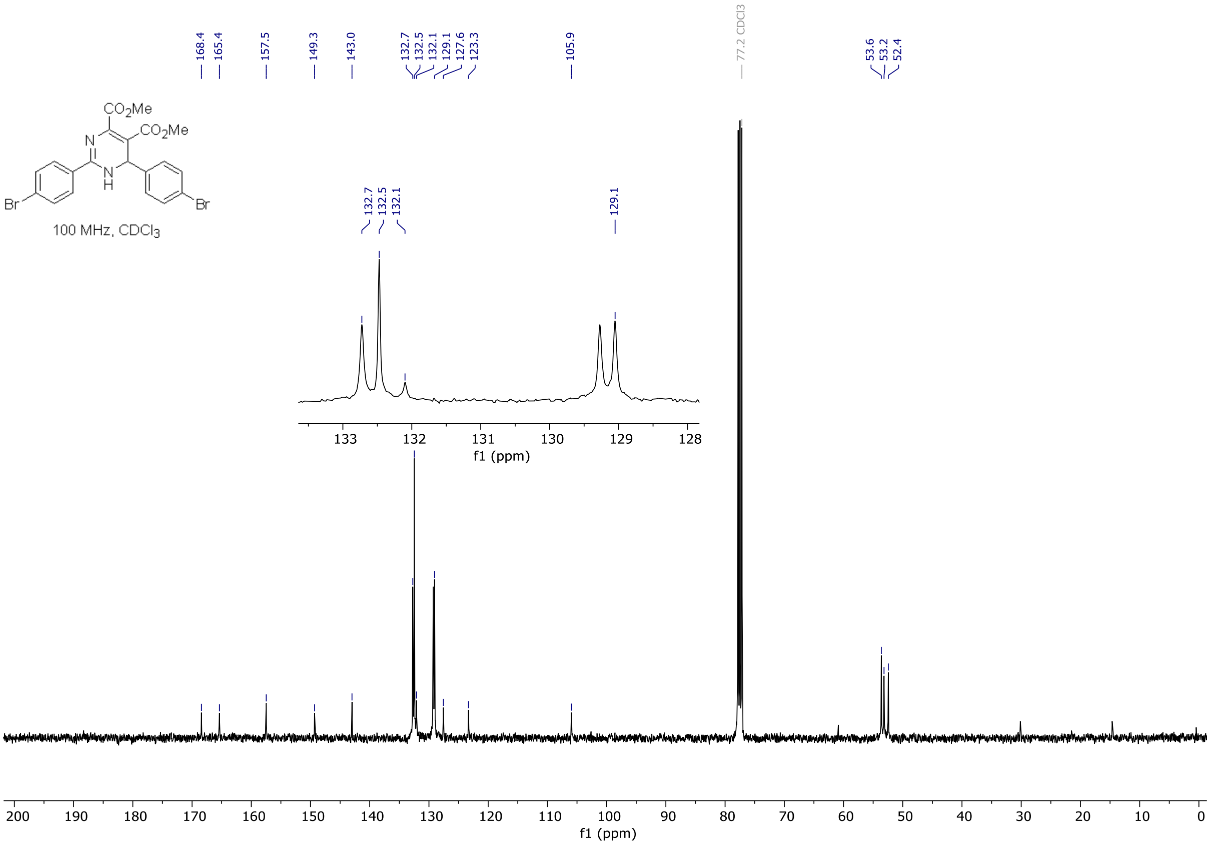

Dimethyl 2,6-di-p-tolyl-1,6-dihydropyrimidine-4,5-dicarboxylate (6d)

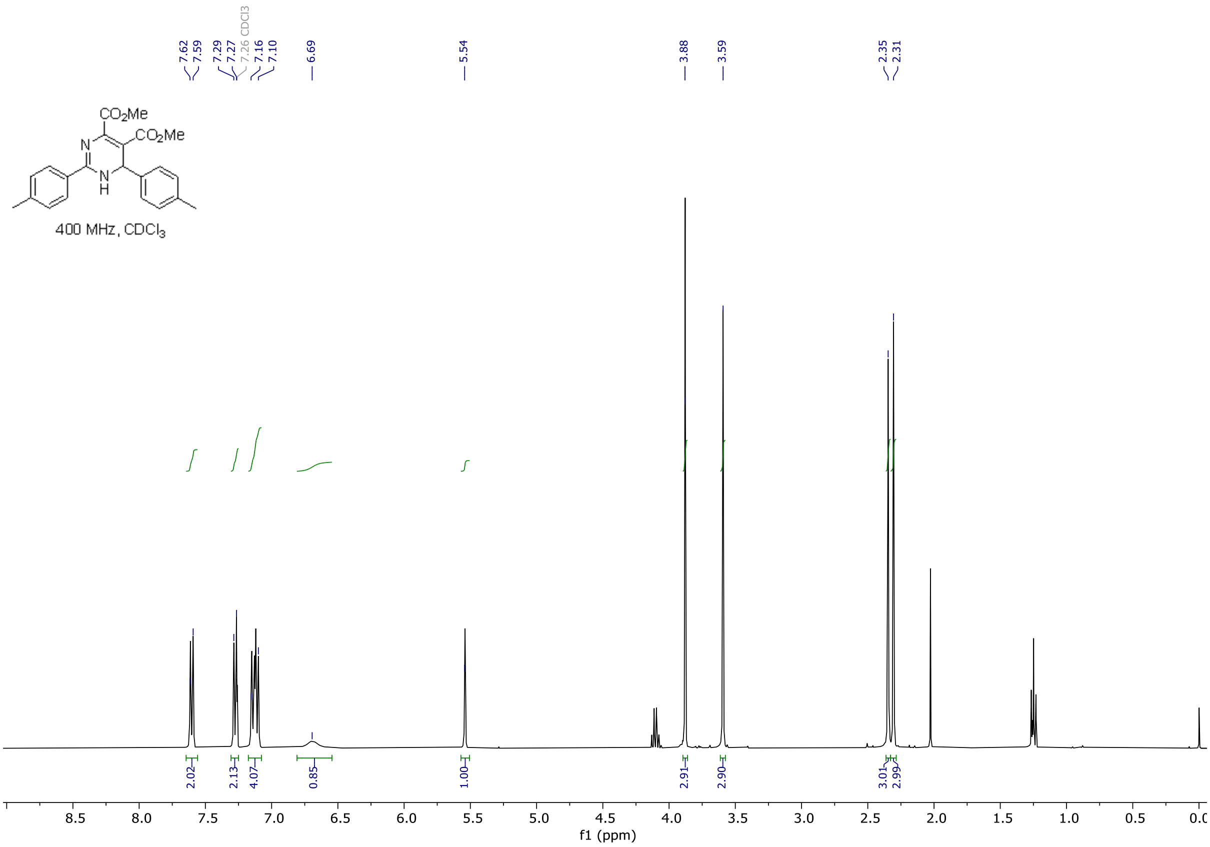

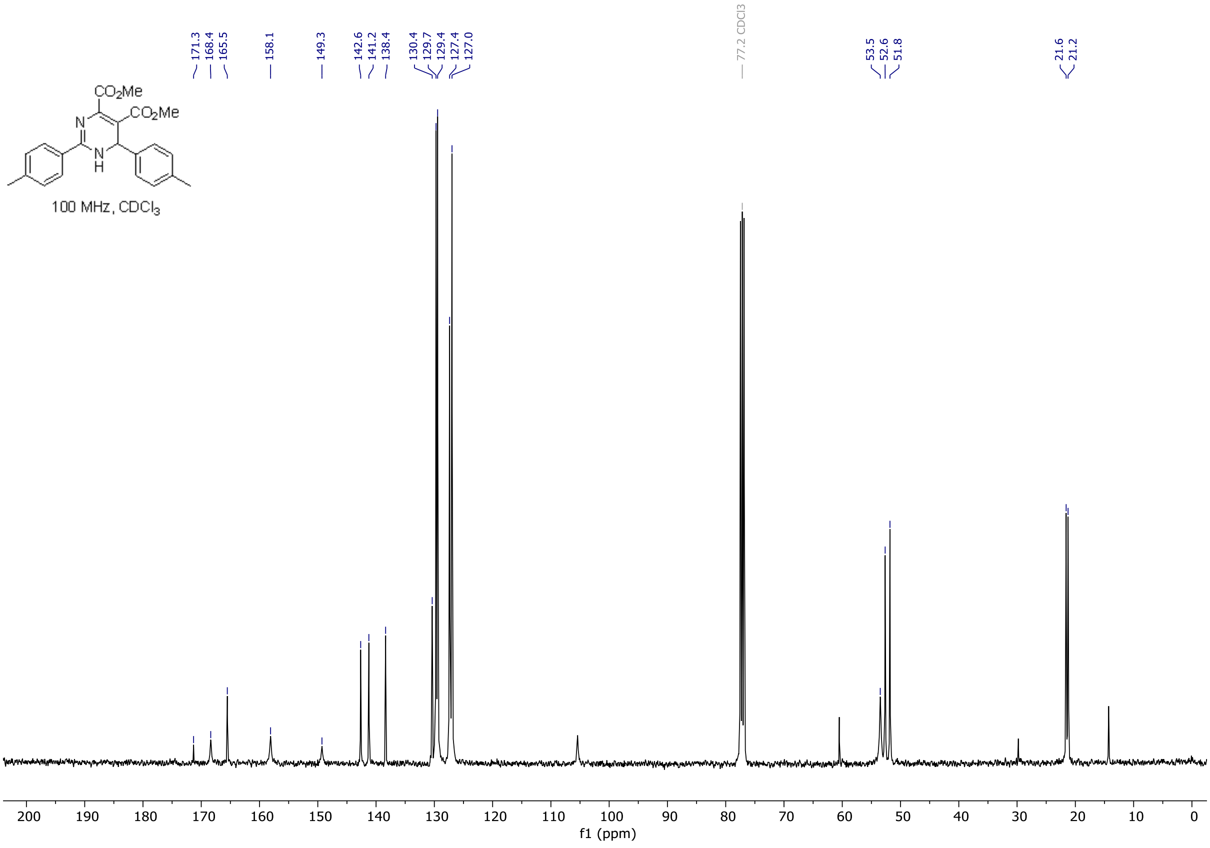

Dimethyl 2,6-bis(3-methoxyphenyl)-1,6-dihydropyrimidine-4,5-dicarboxylate (6e)

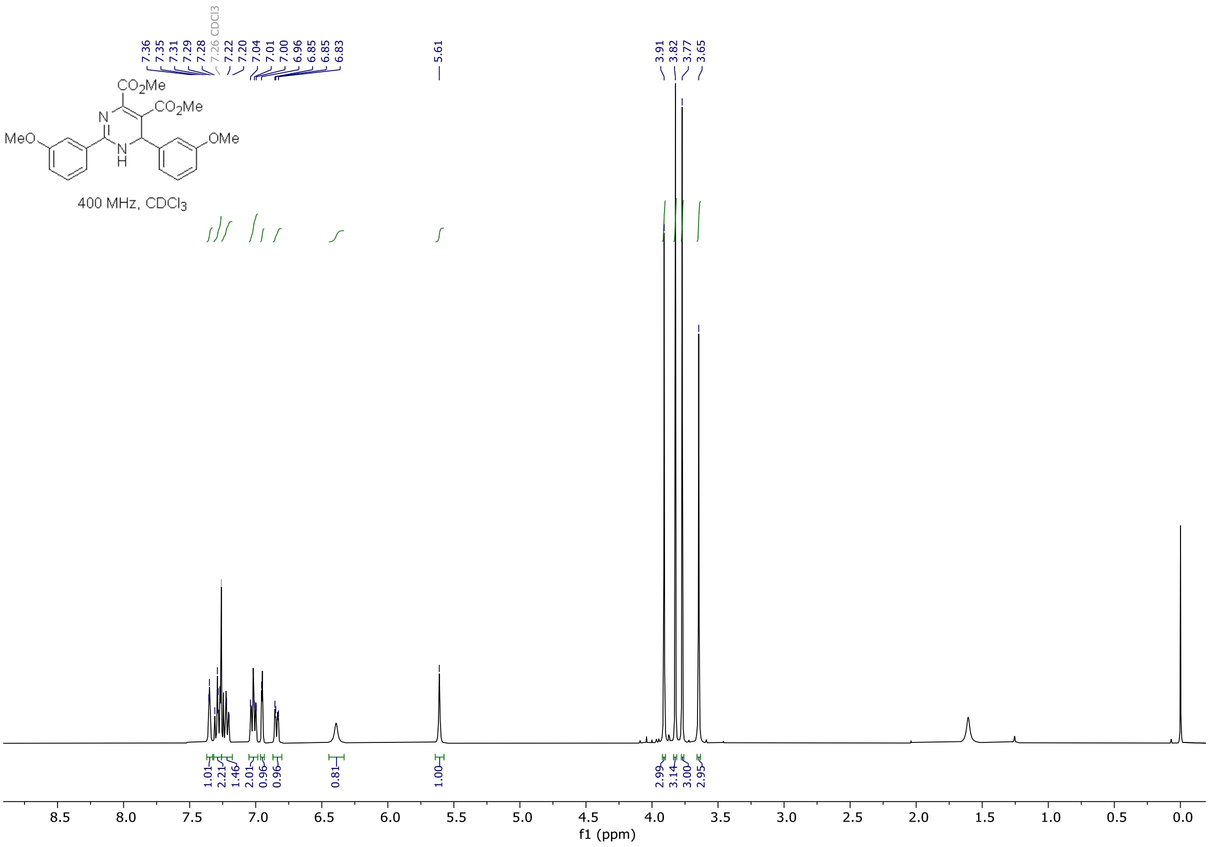

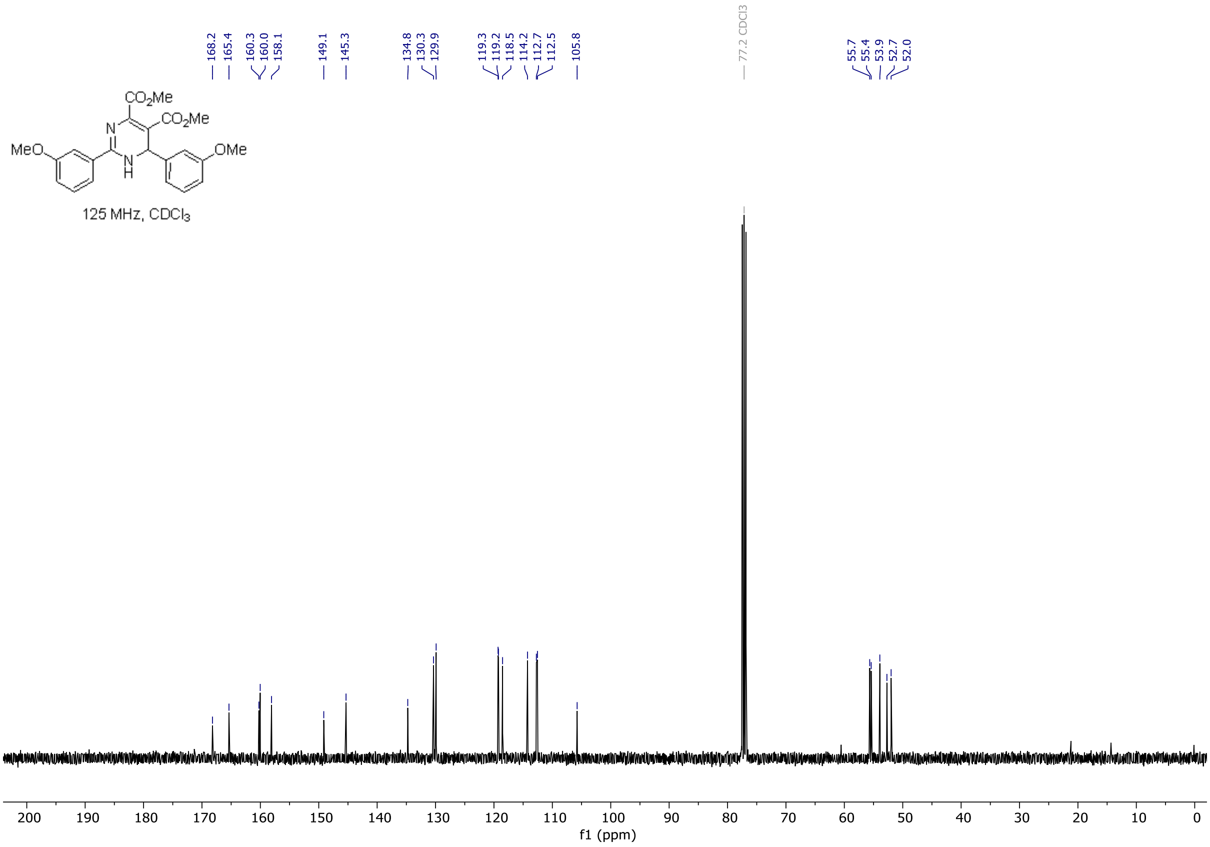

Dimethyl 2,6-bis(2,4-difluorophenyl)-1,6-dihydropyrimidine-4,5-dicarboxylate (6f)

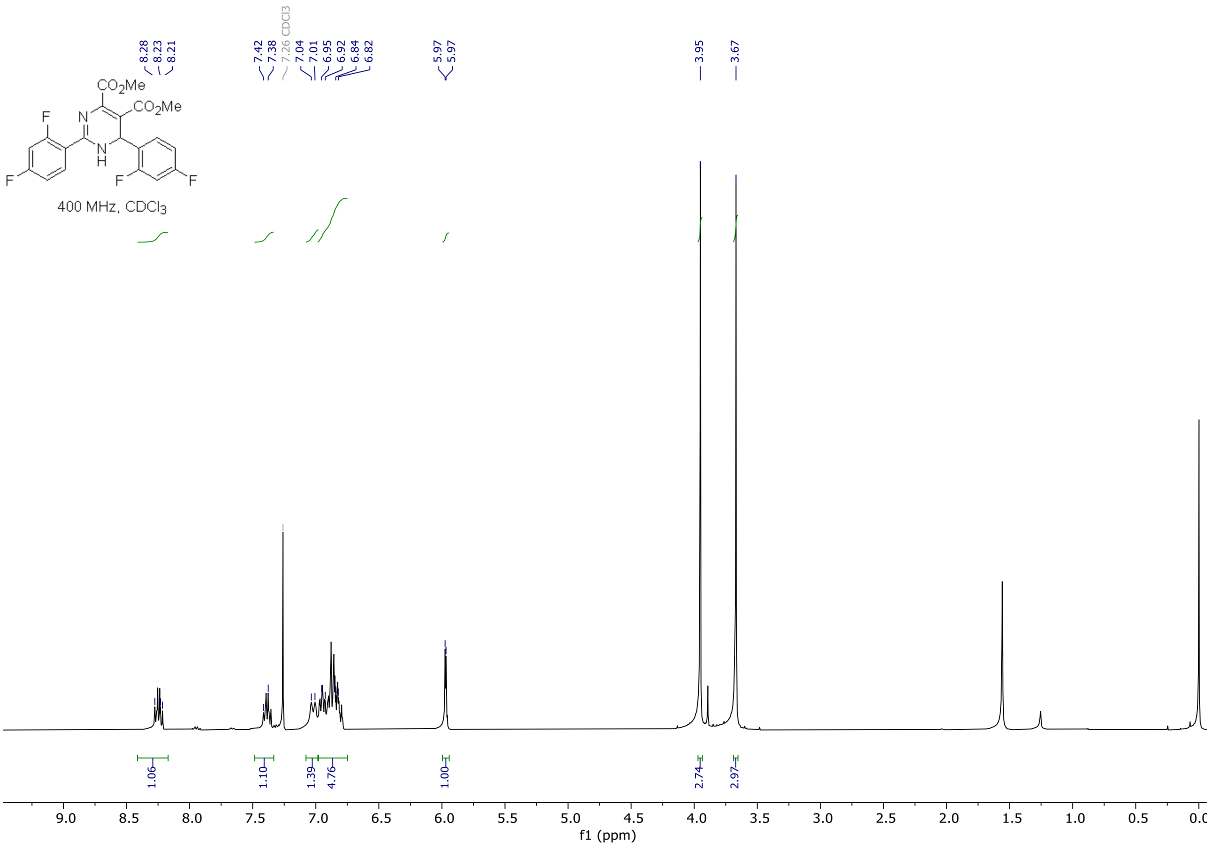

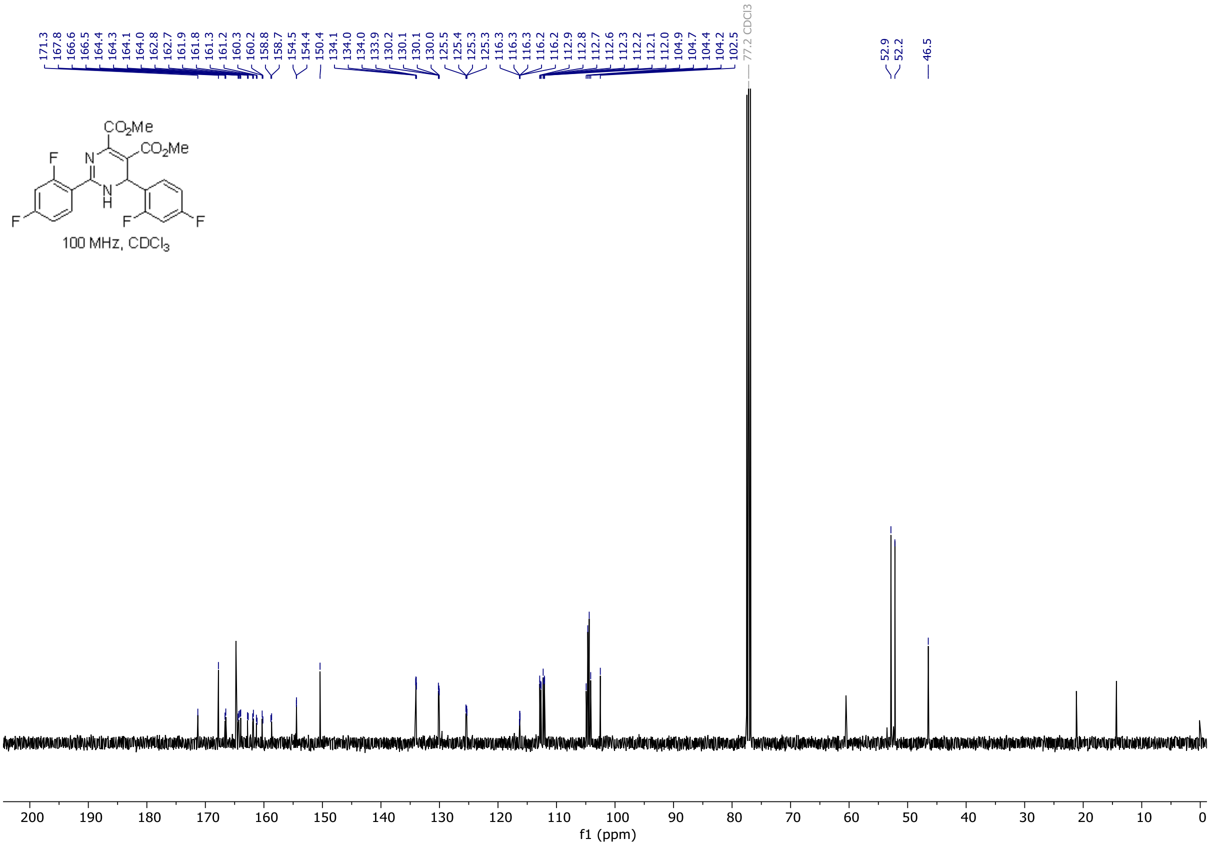

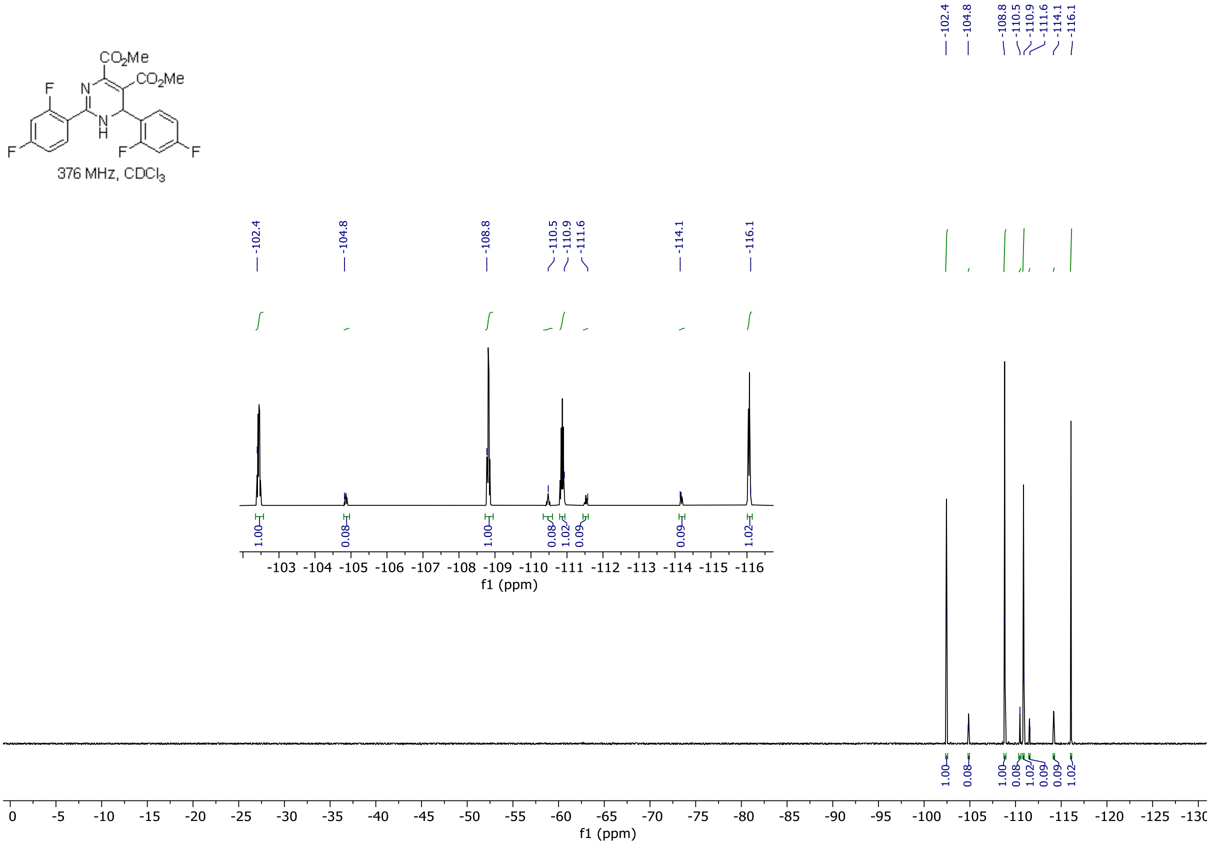
376 MHz, CDCl3
f1 (ppm)

Dimethyl 2,6-bis(2-(trifluoromethyl)phenyl)-1,6-dihydropyrimidine-4,5-dicarboxylate (6g)

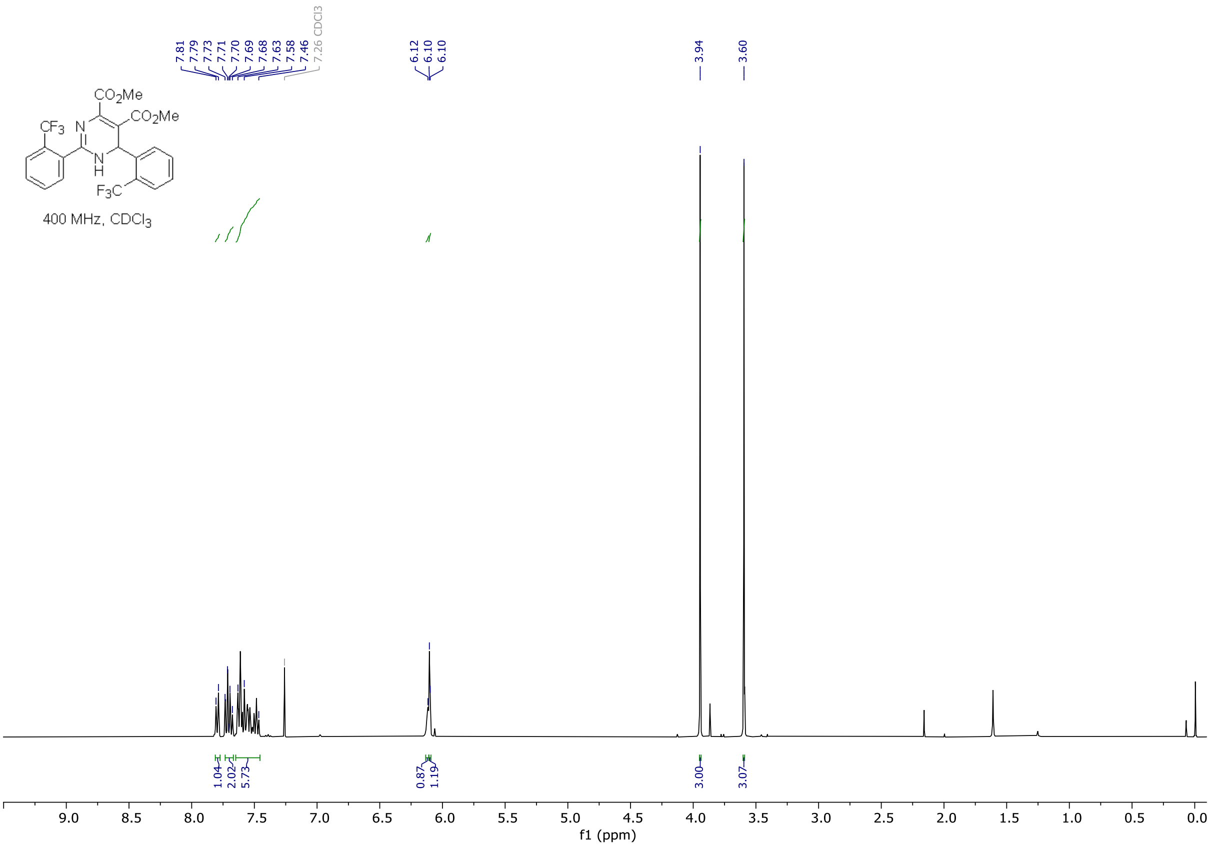

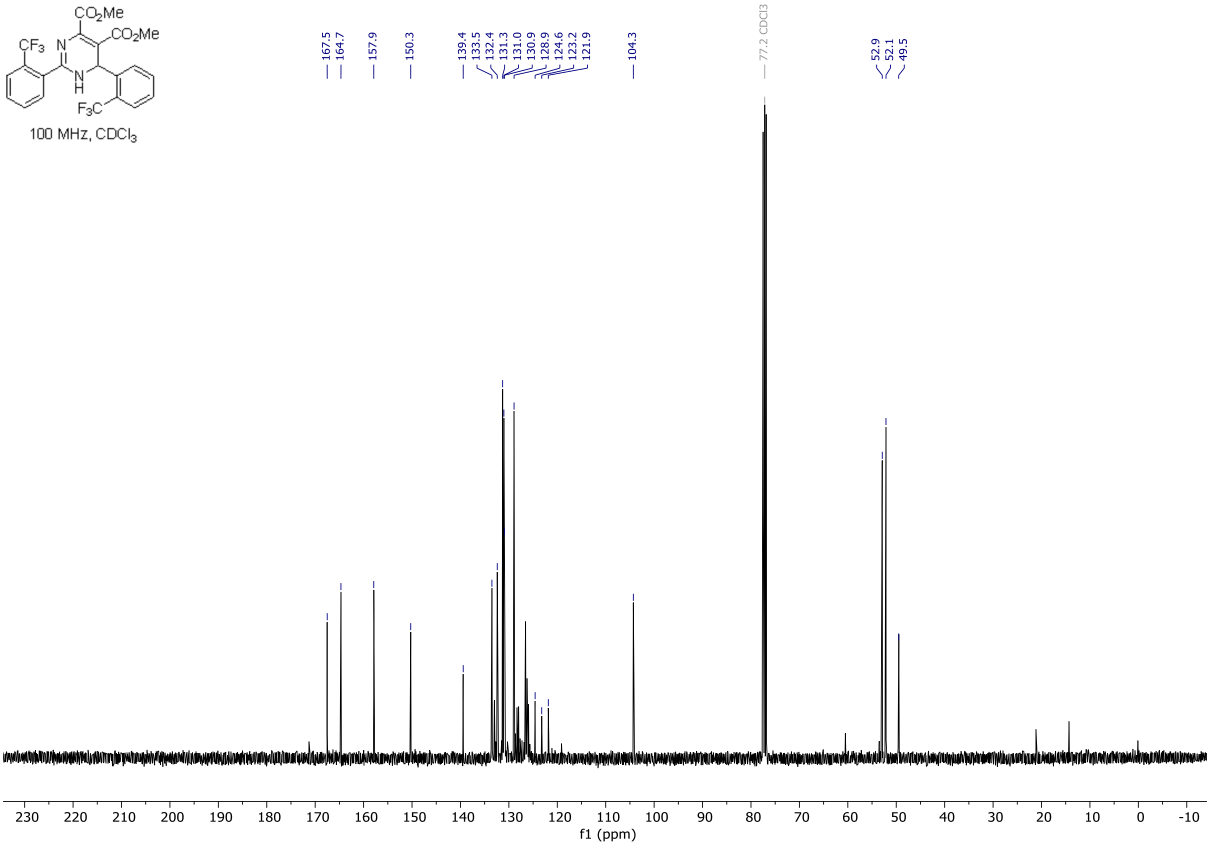

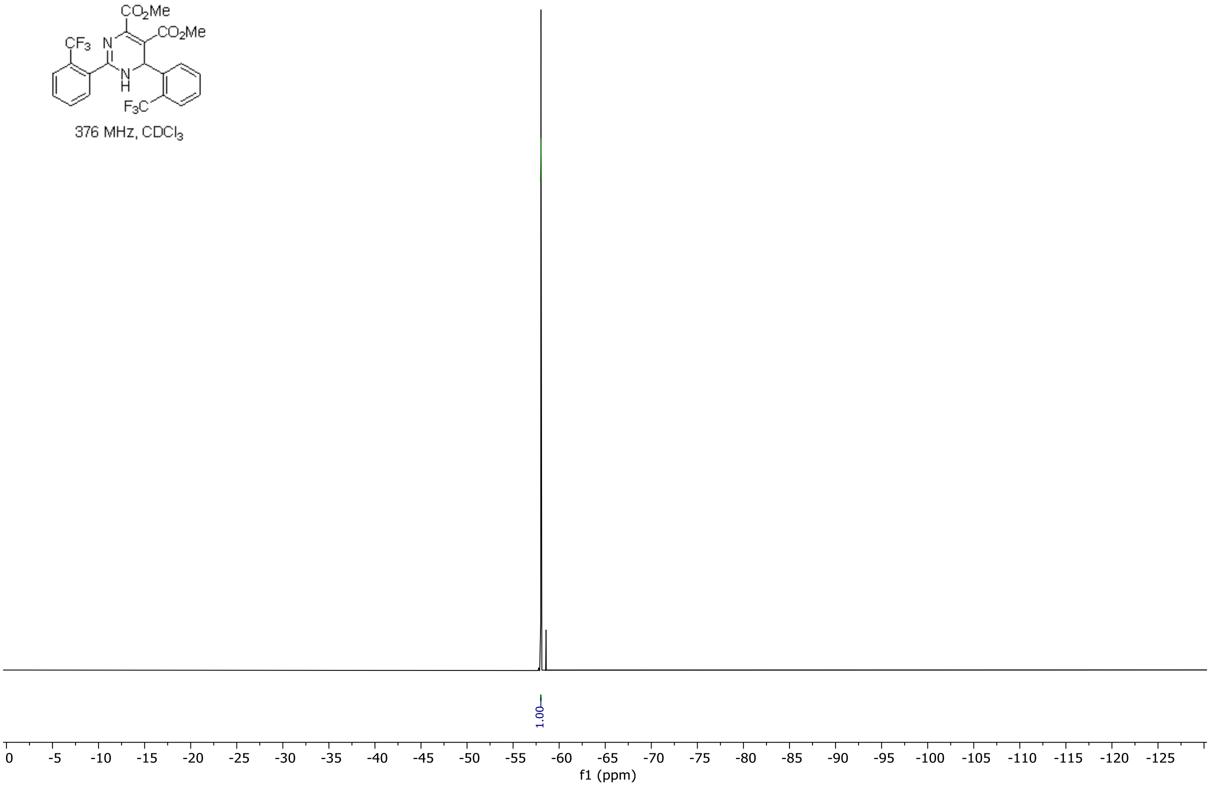
CO2Me
CF3
N
CO2Me
N
H
F3C
376 MHz, CDCl3
1.00
0 -5 -10 -15 -20 -25 -30 -35 -40 -45 -50 -55 -60 -65 -70 -75 -80 -85 -90 -95 -100 -105 -110 -115 -120 -125
f1 (ppm)

Di-tert-butyl 2,6-diphenyl-1,6-dihydropyrimidine-4,5-dicarboxylate (6h)

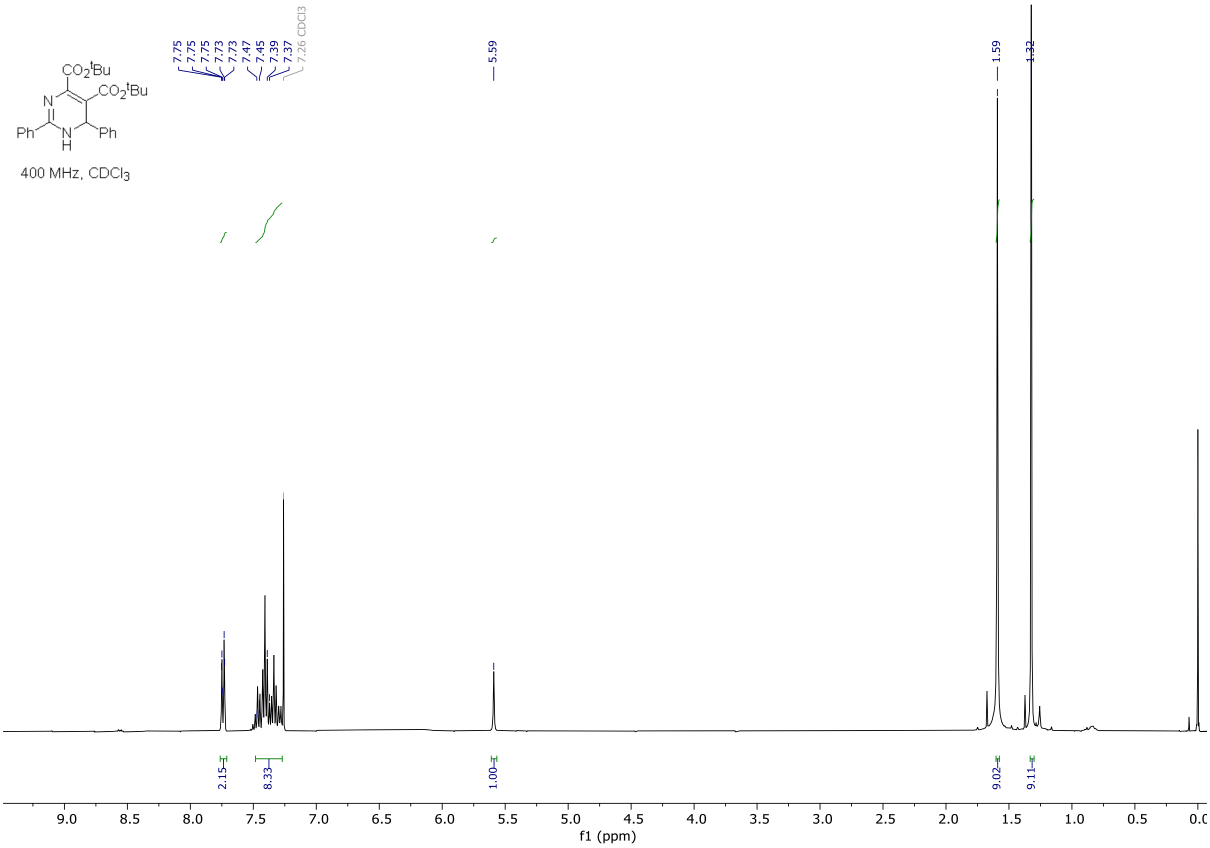

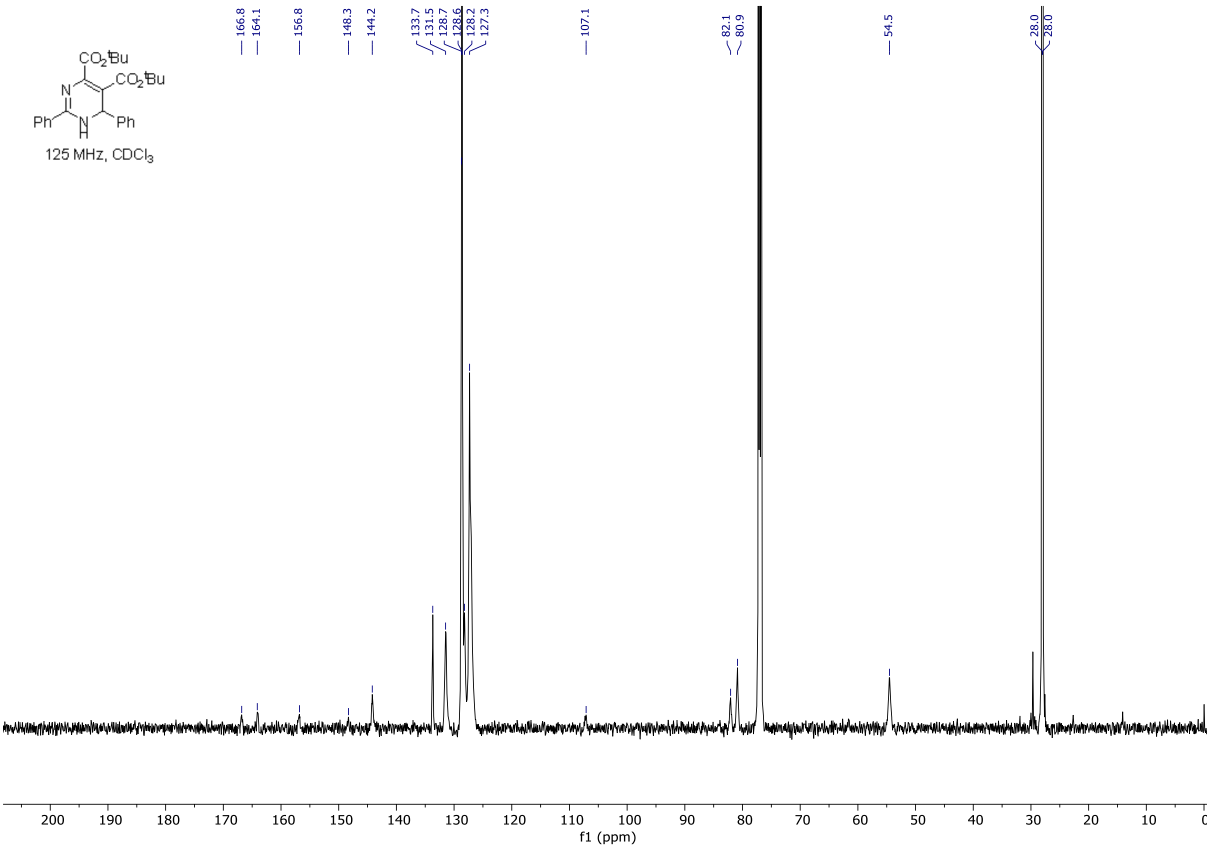

Di-tert-butyl 2,6-bis(4-fluorophenyl)-1,6-dihydropyrimidine-4,5-dicarboxylate (6i)

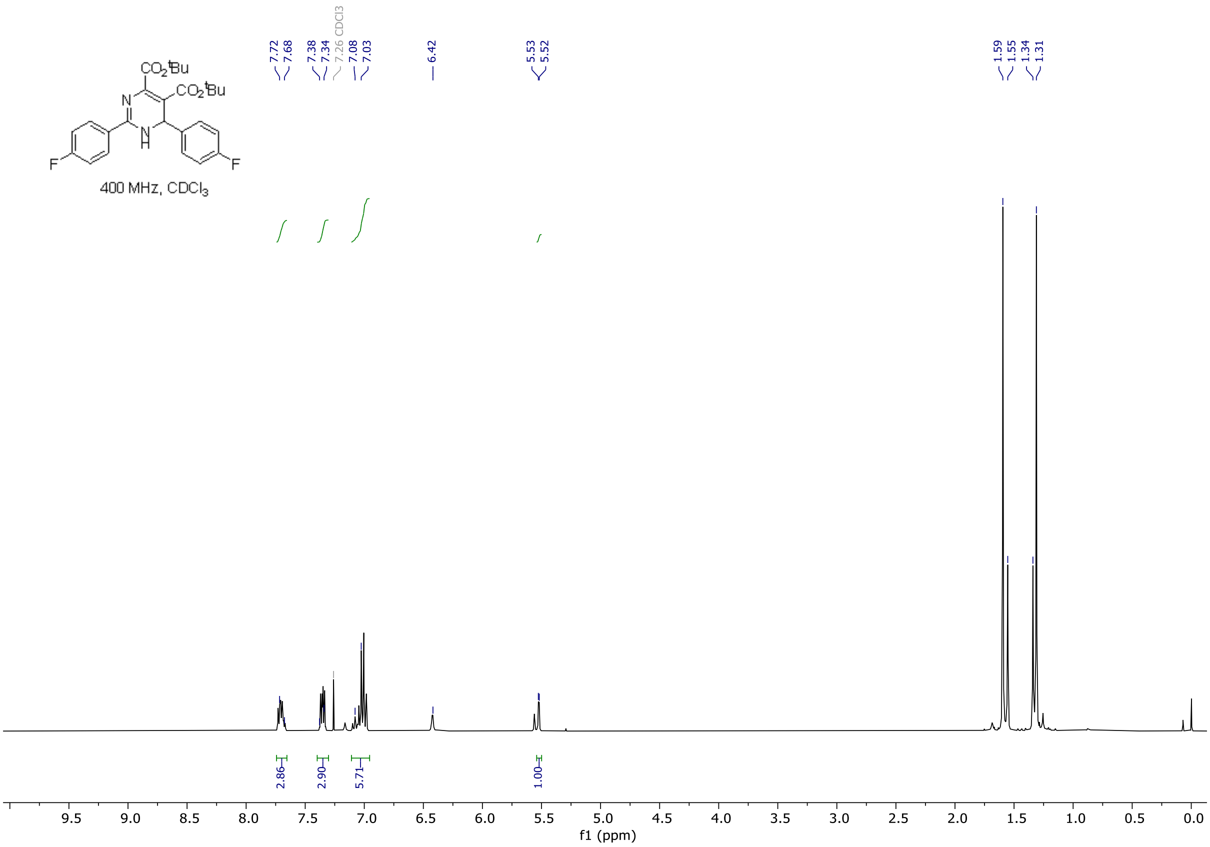

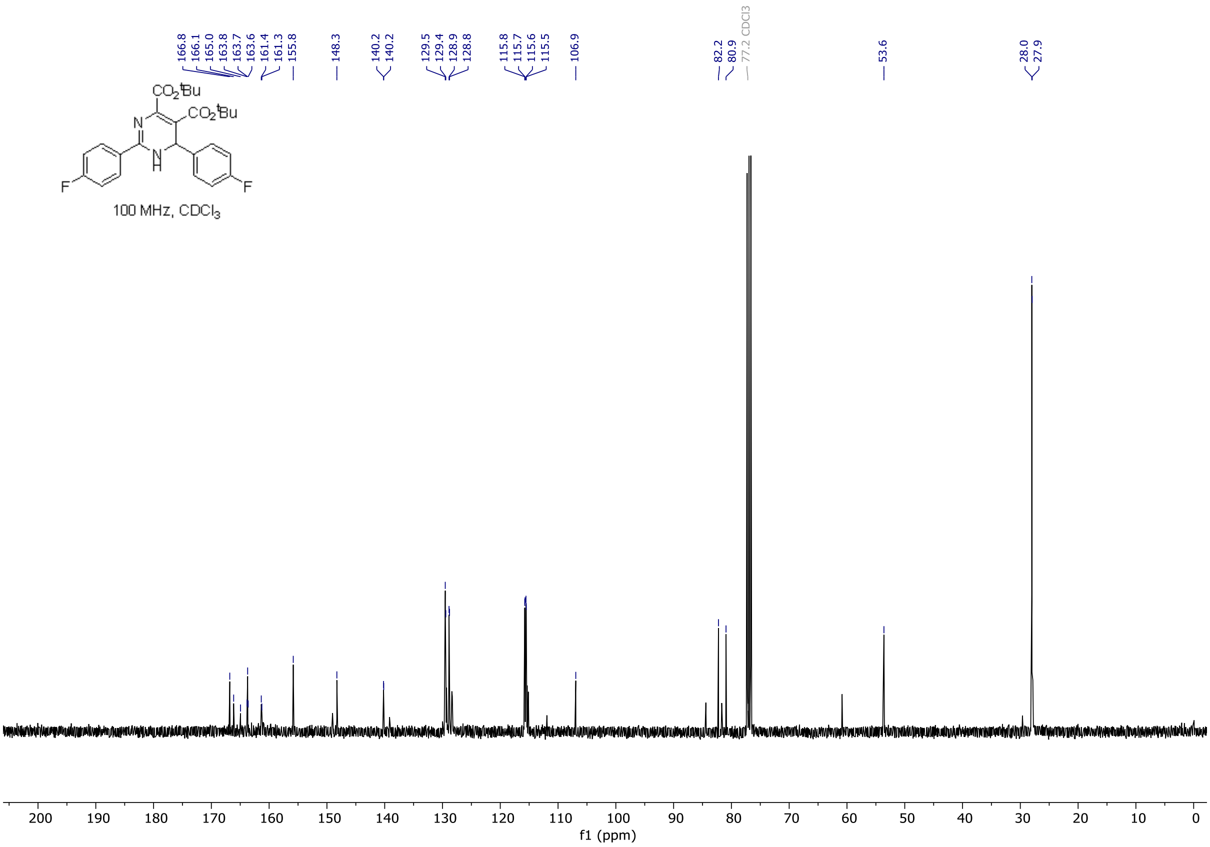

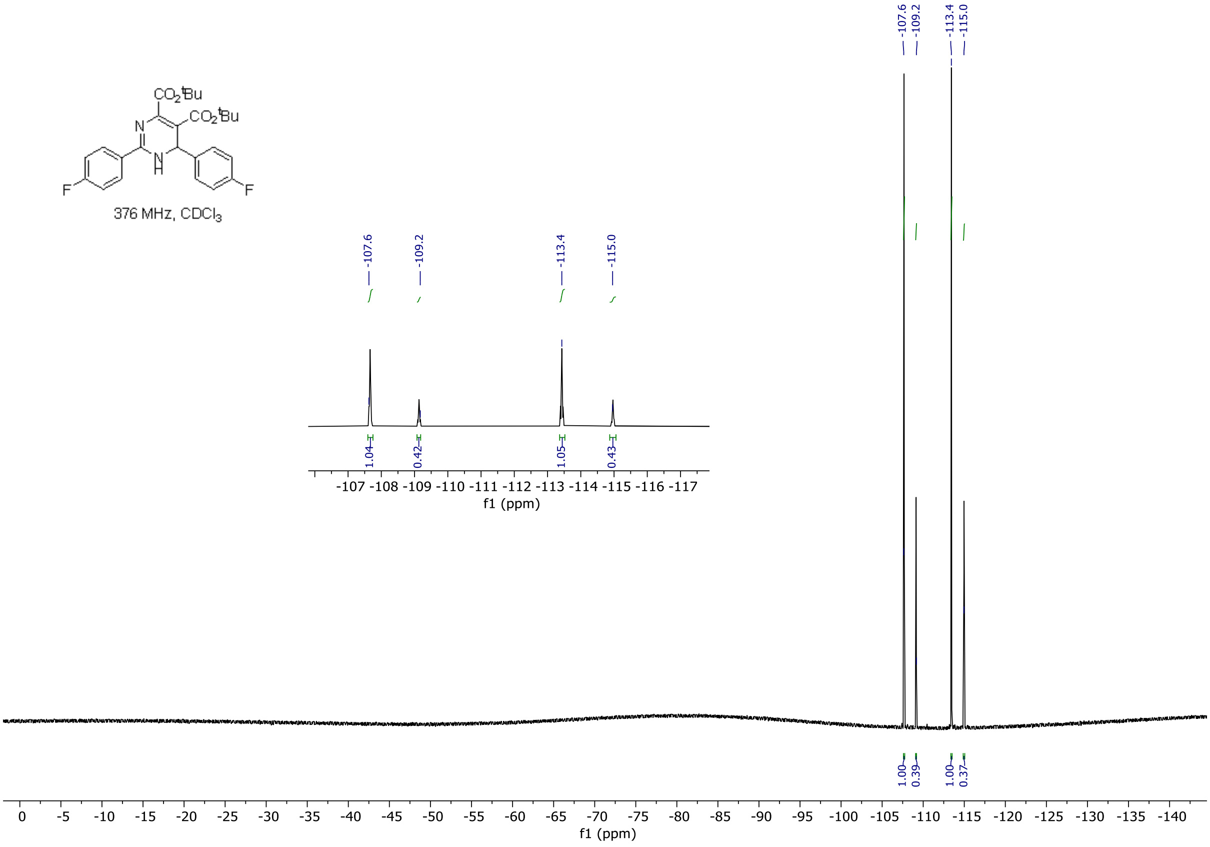

CO2tBu
CO2tBu
N
H
F
F
376 MHz, CDCl3
-107.6
-109.2
-113.4
-115.0
1.04
0.42
1.05
0.43
-107 -108 -109 -110 -111 -112 -113 -114 -115 -116 -117
f1 (ppm)
1.00
0.39
1.00
0.37
0 -5 -10 -15 -20 -25 -30 -35 -40 -45 -50 -55 -60 -65 -70 -75 -80 -85 -90 -95 -100 -105 -110 -115 -120 -125 -130 -135 -140
f1 (ppm)

Di-tert-butyl 2,6-bis(4-methoxyphenyl)-1,6-dihydropyrimidine-4,5-dicarboxylate (6j)

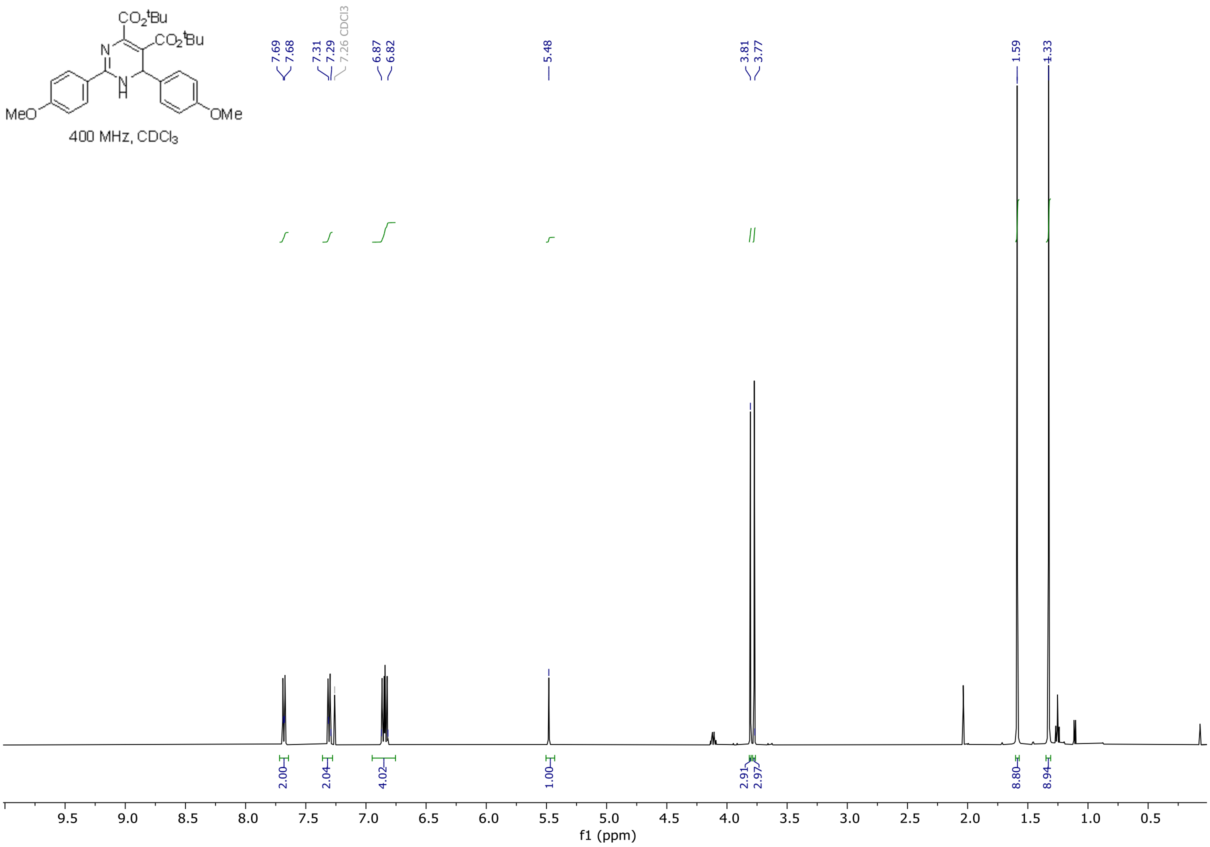

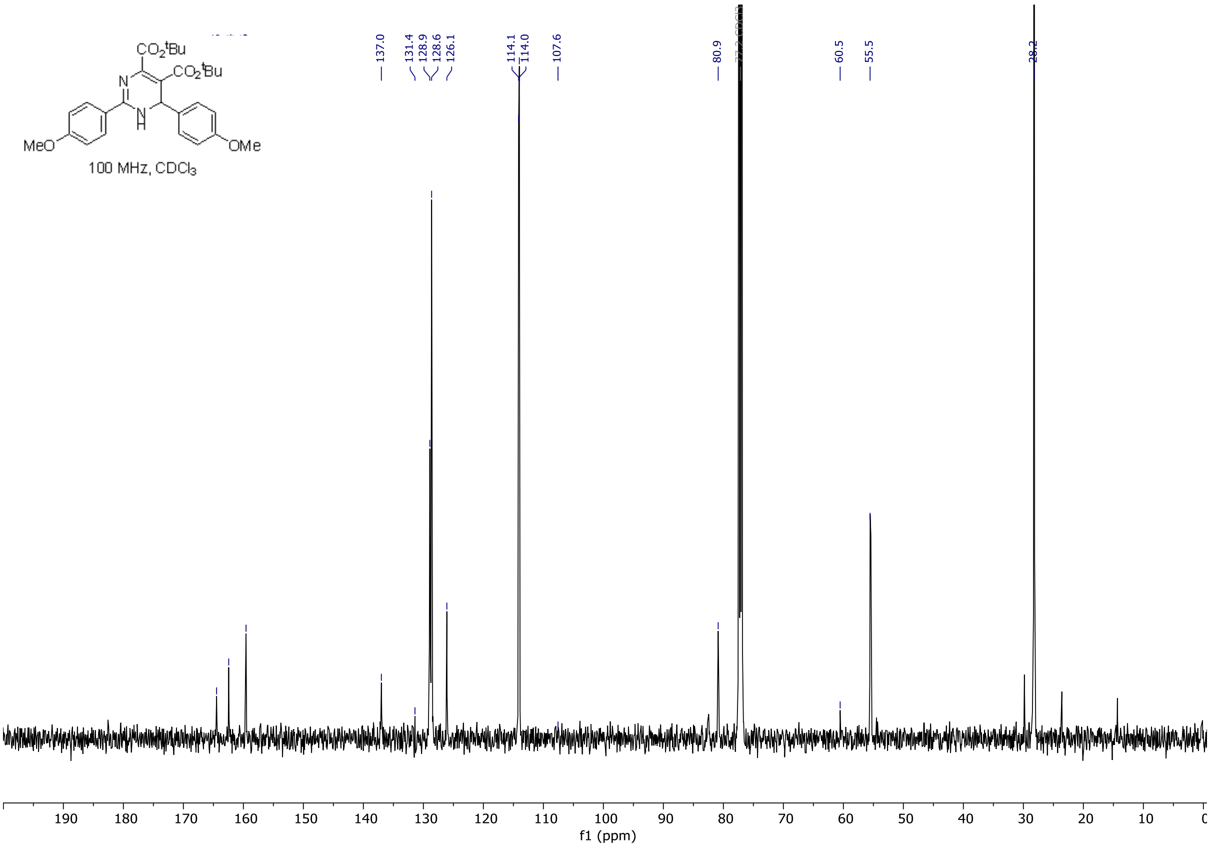

Di-tert-pentyl2,6-bis(4-fluorophenyl)-1,6-dihydropyrimidine-4,5-dicarboxylate(6k)

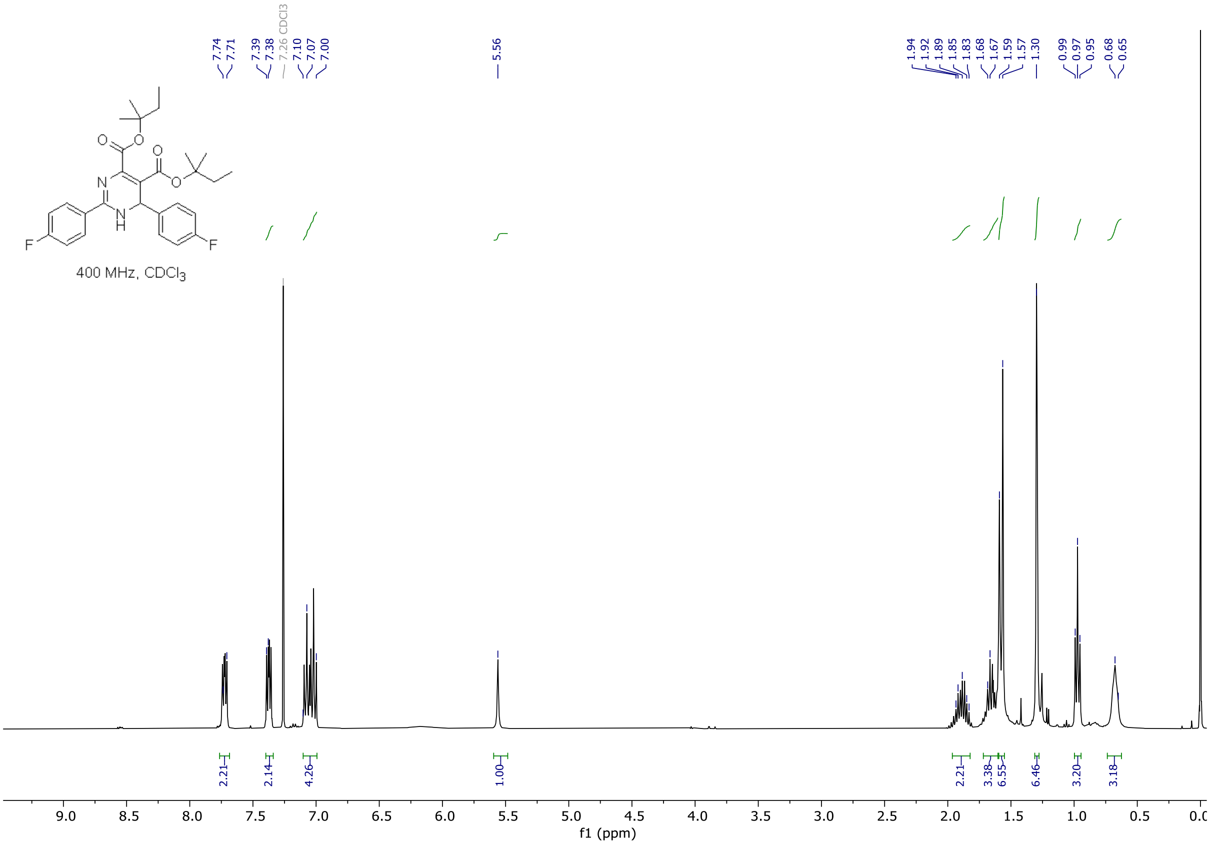

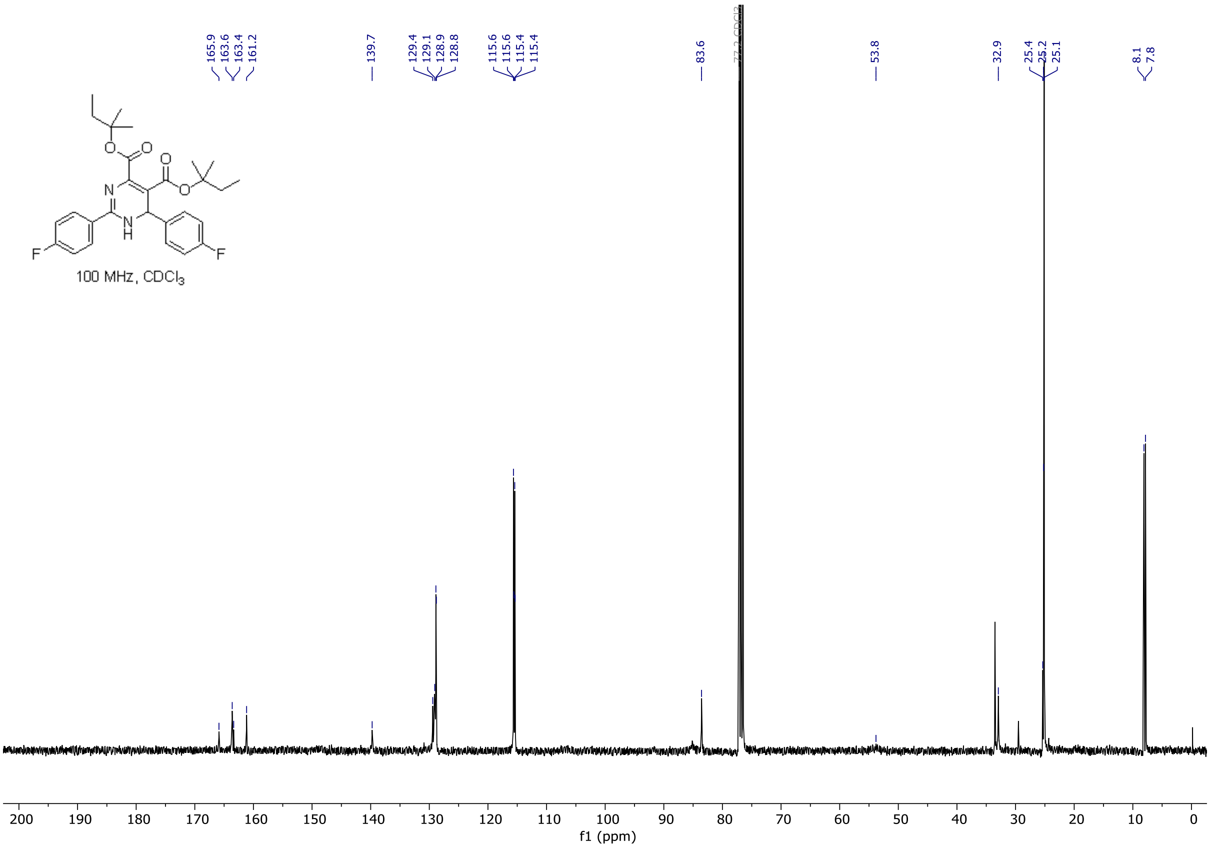

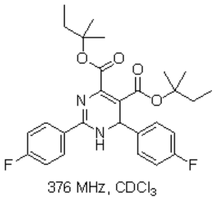
F
N
H
F
376 MHz, CDCl3

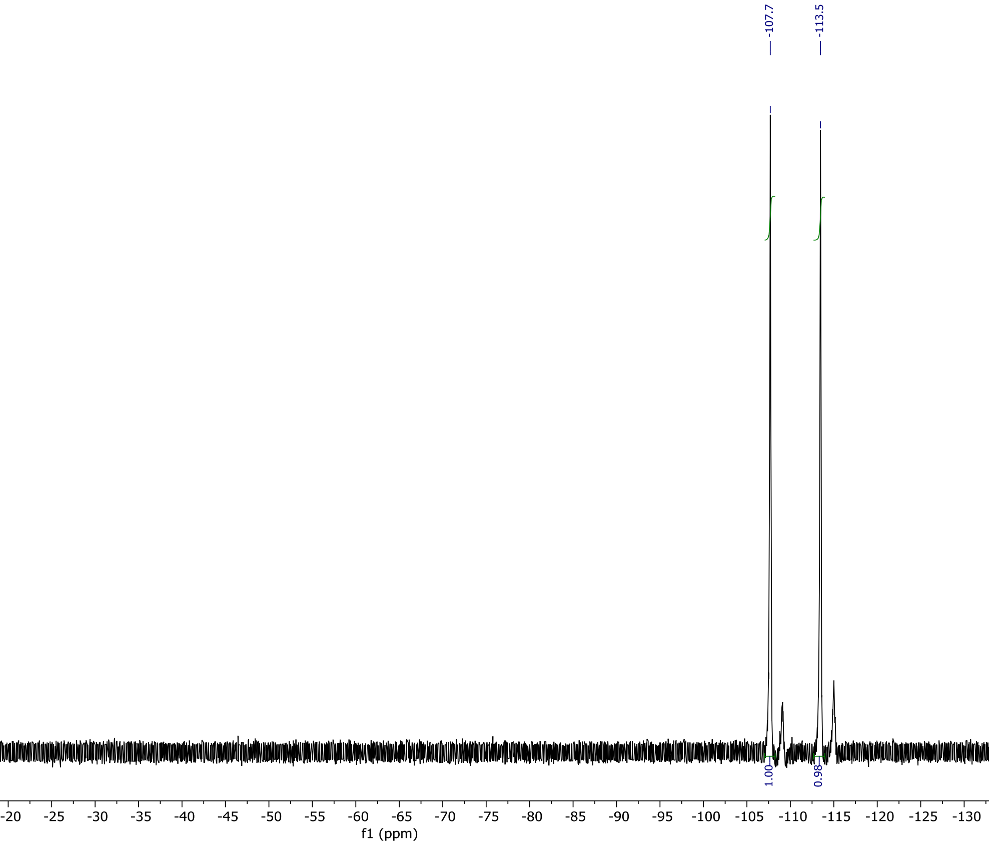
-107.7
-113.5
1.00
0.98
f1 (ppm)

Dimethyl 2,6-diphenylpyrimidine-4,5-dicarboxylate (7a)

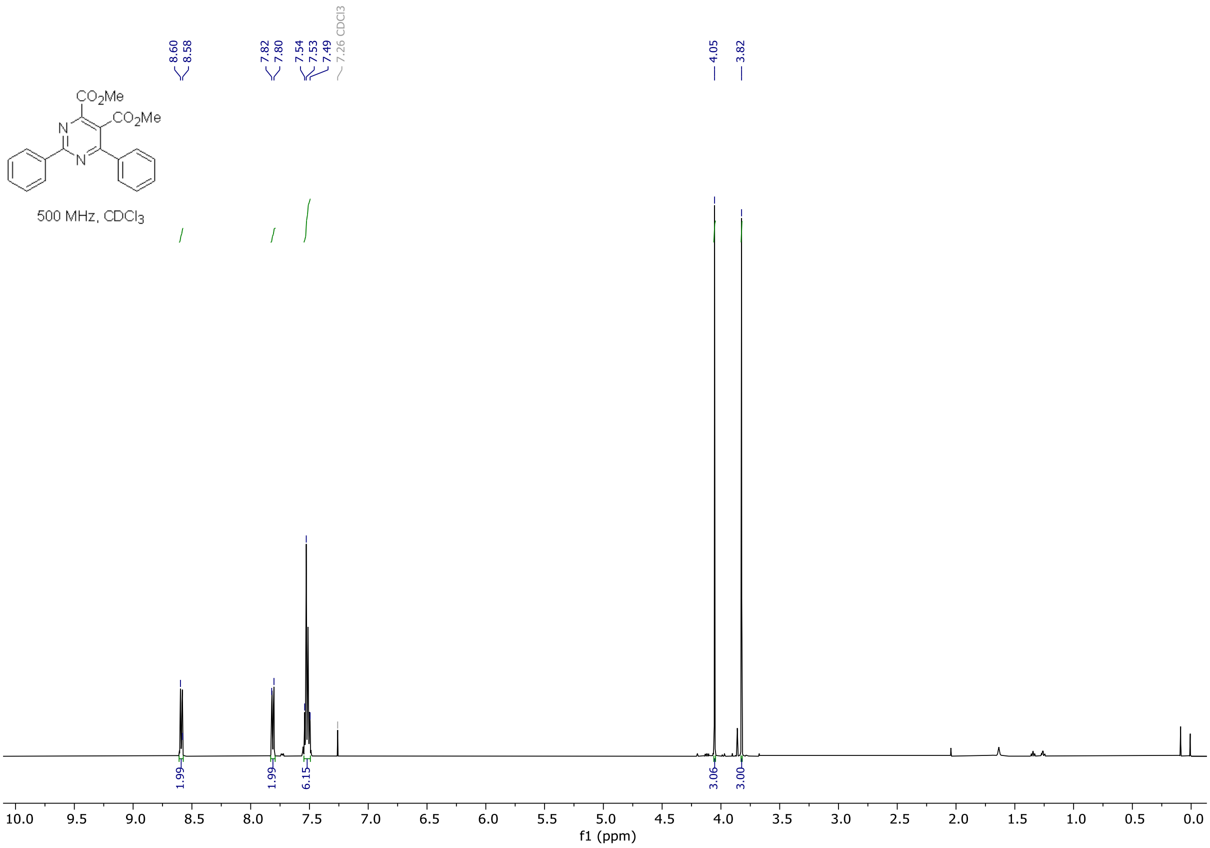

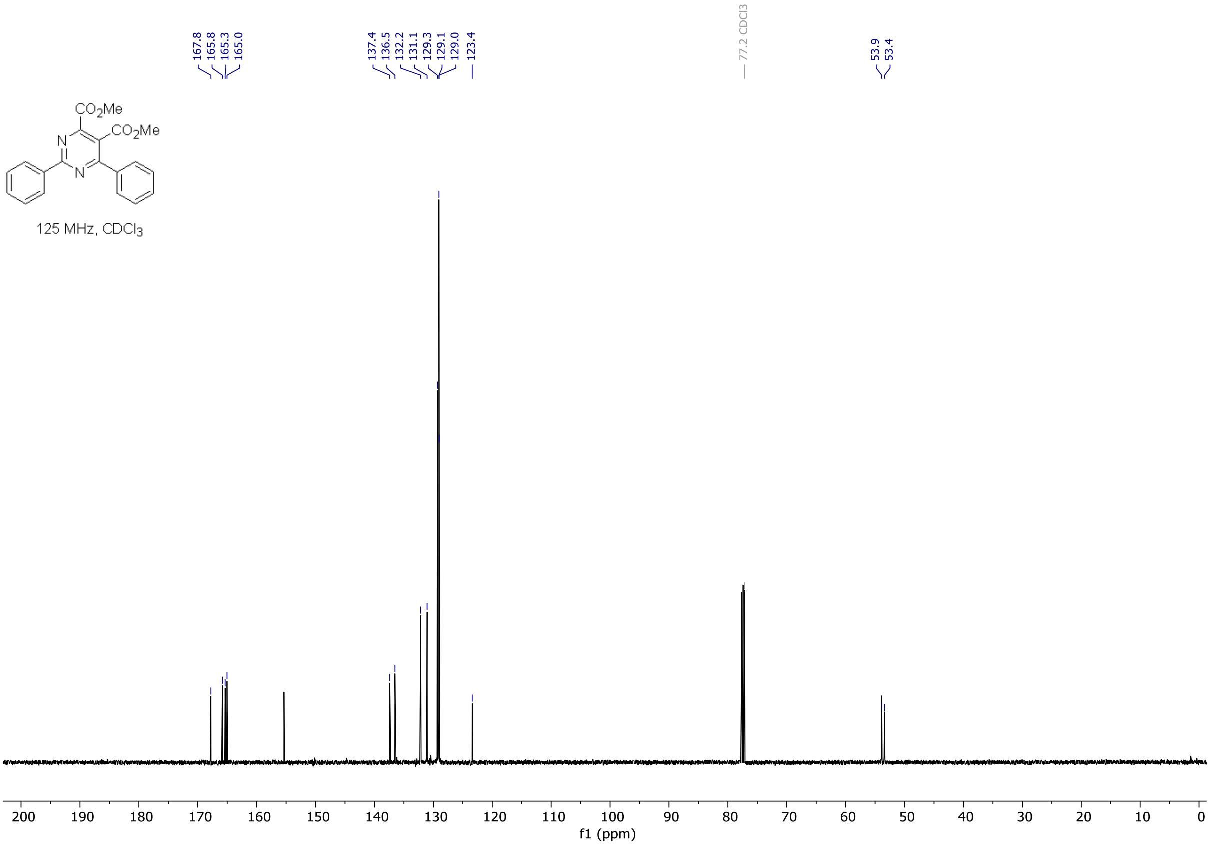

Dimethyl 2,6-bis(4-fluorophenyl)pyrimidine-4,5-dicarboxylate (7b)

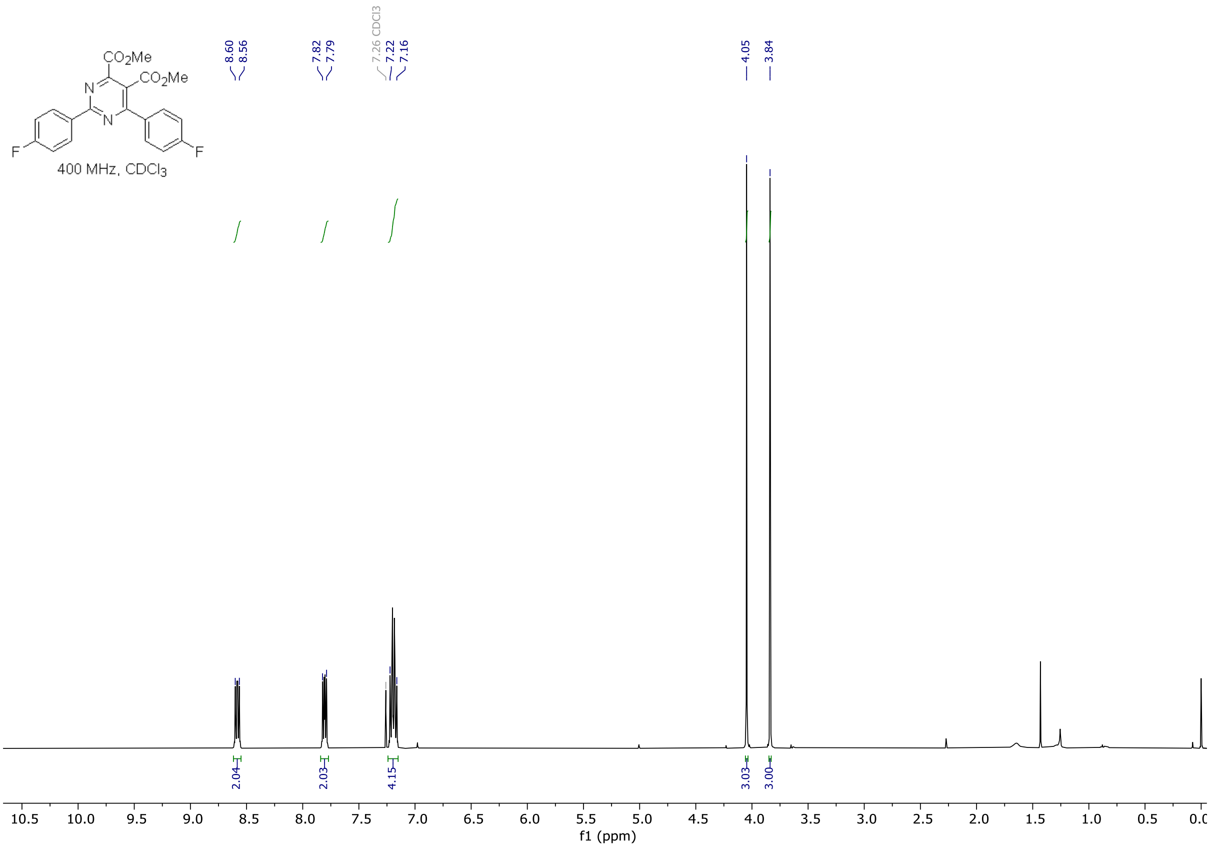

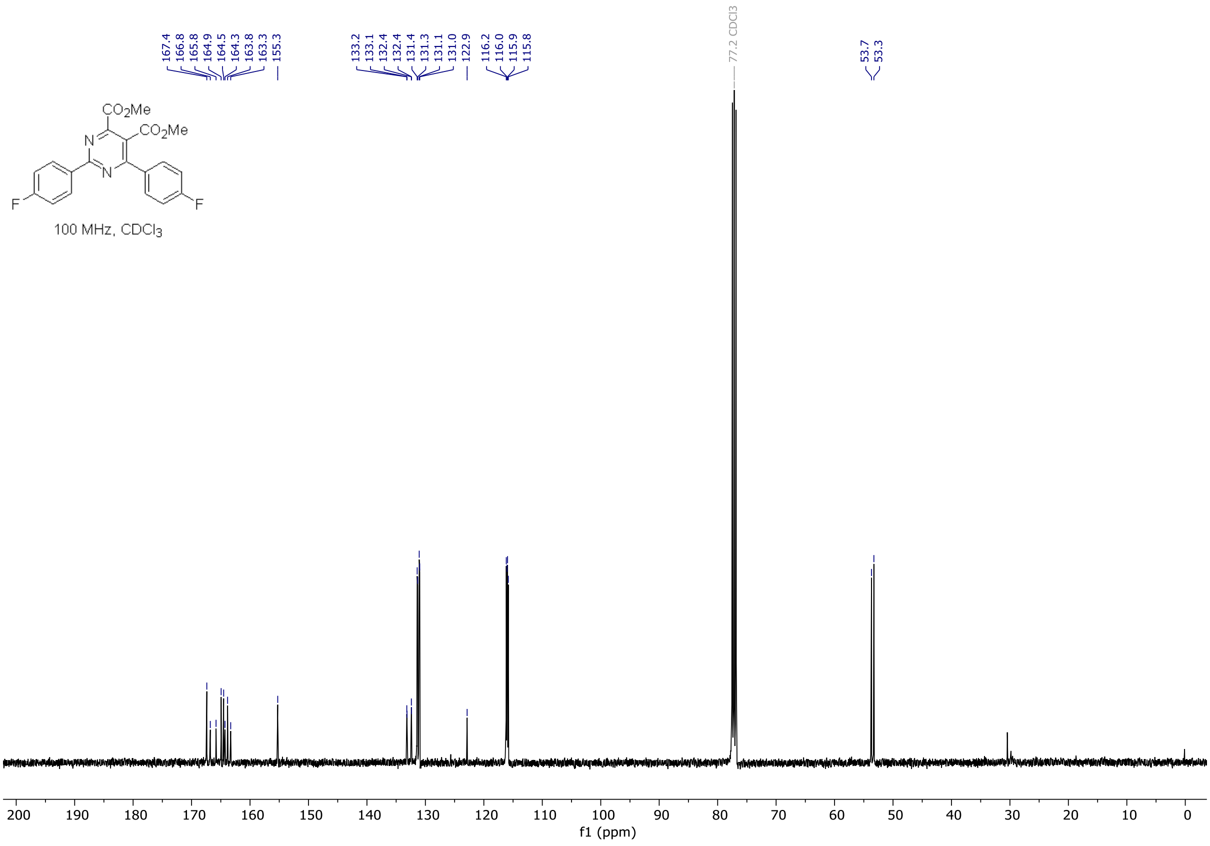

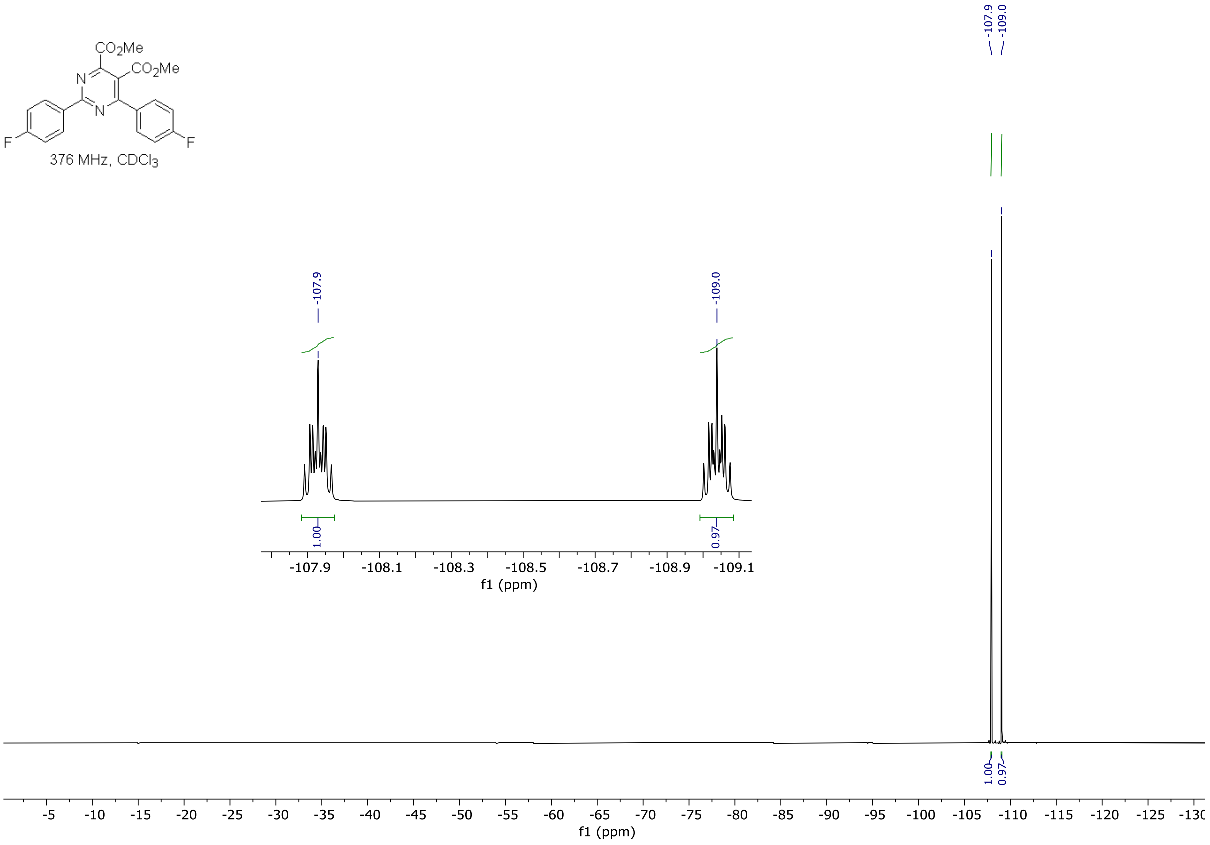

CO2Me
CO2Me
N
N
F
F
376 MHz, CDCl3
-107.9
-109.0
1.00
0.97
-107.9 -108.1 -108.3 -108.5 -108.7 -108.9 -109.1
f1 (ppm)
-5 -10 -15 -20 -25 -30 -35 -40 -45 -50 -55 -60 -65 -70 -75 -80 -85 -90 -95 -100 -105 -110 -115 -120 -125 -130
f1 (ppm)

Dimethyl 2,6-bis(4-bromophenyl)pyrimidine-4,5-dicarboxylate (7c)

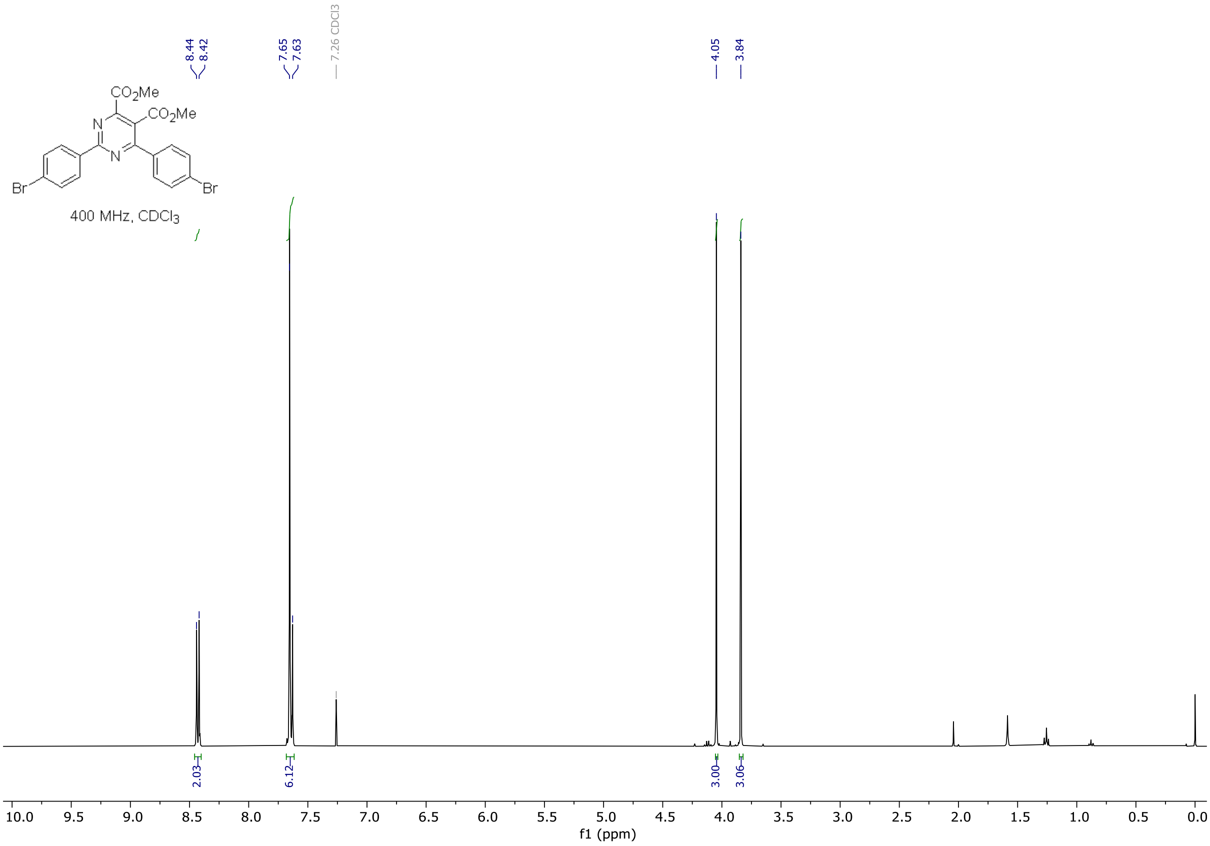

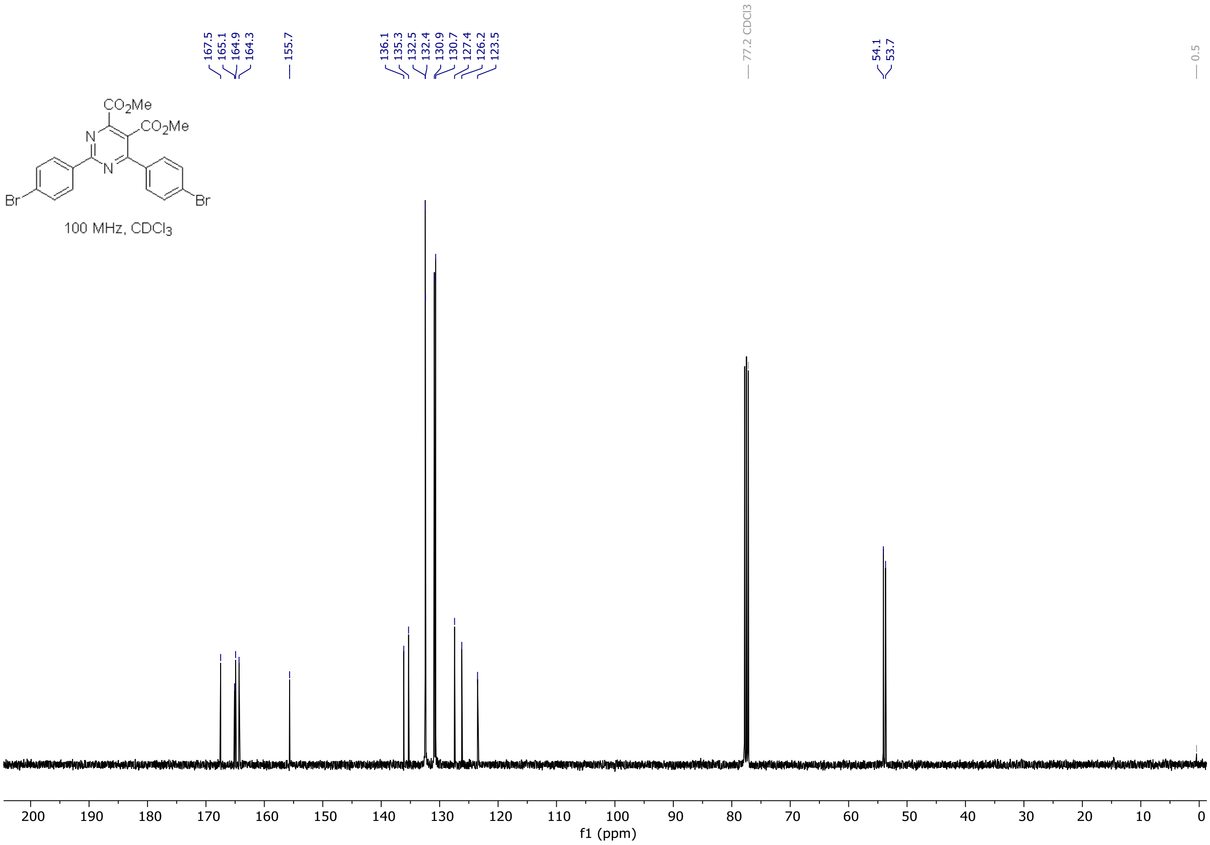

Dimethyl 2,6-di-p-tolylpyrimidine-4,5-dicarboxylate (7d)

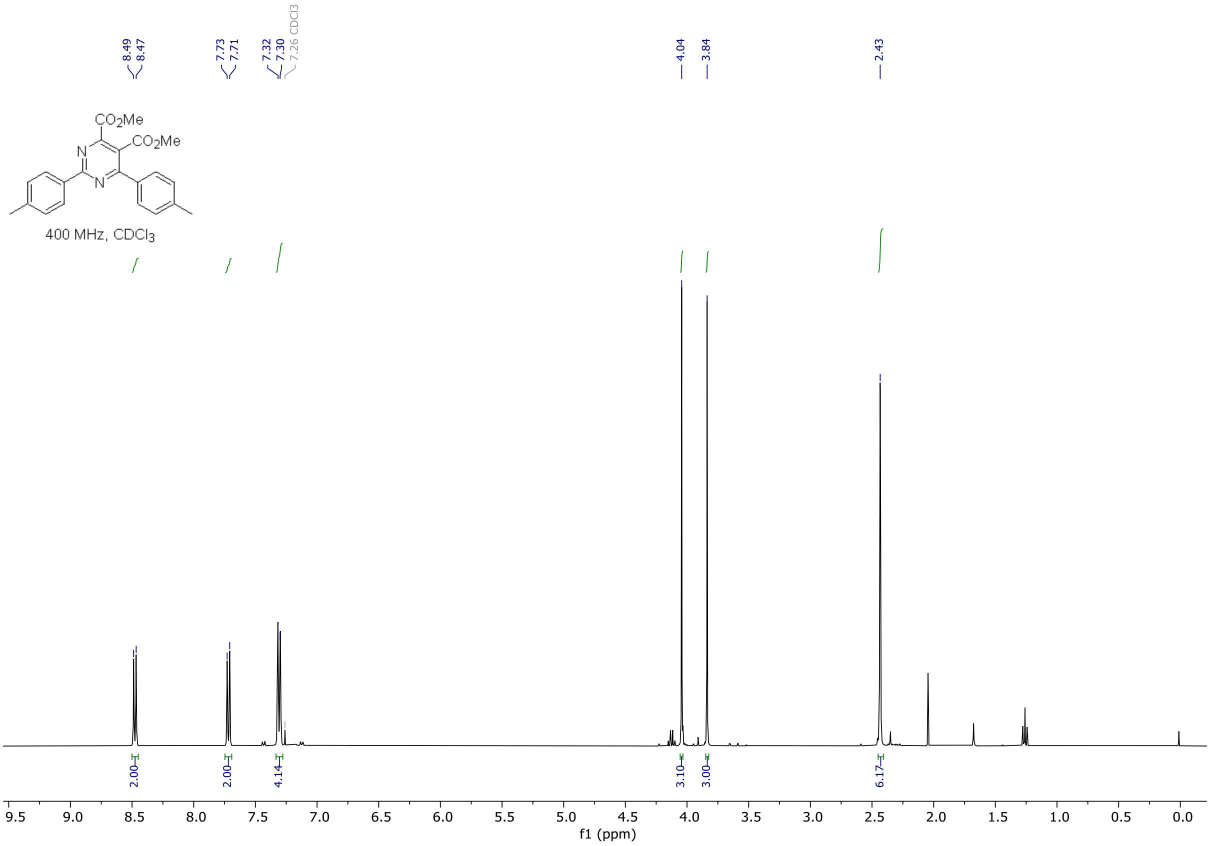

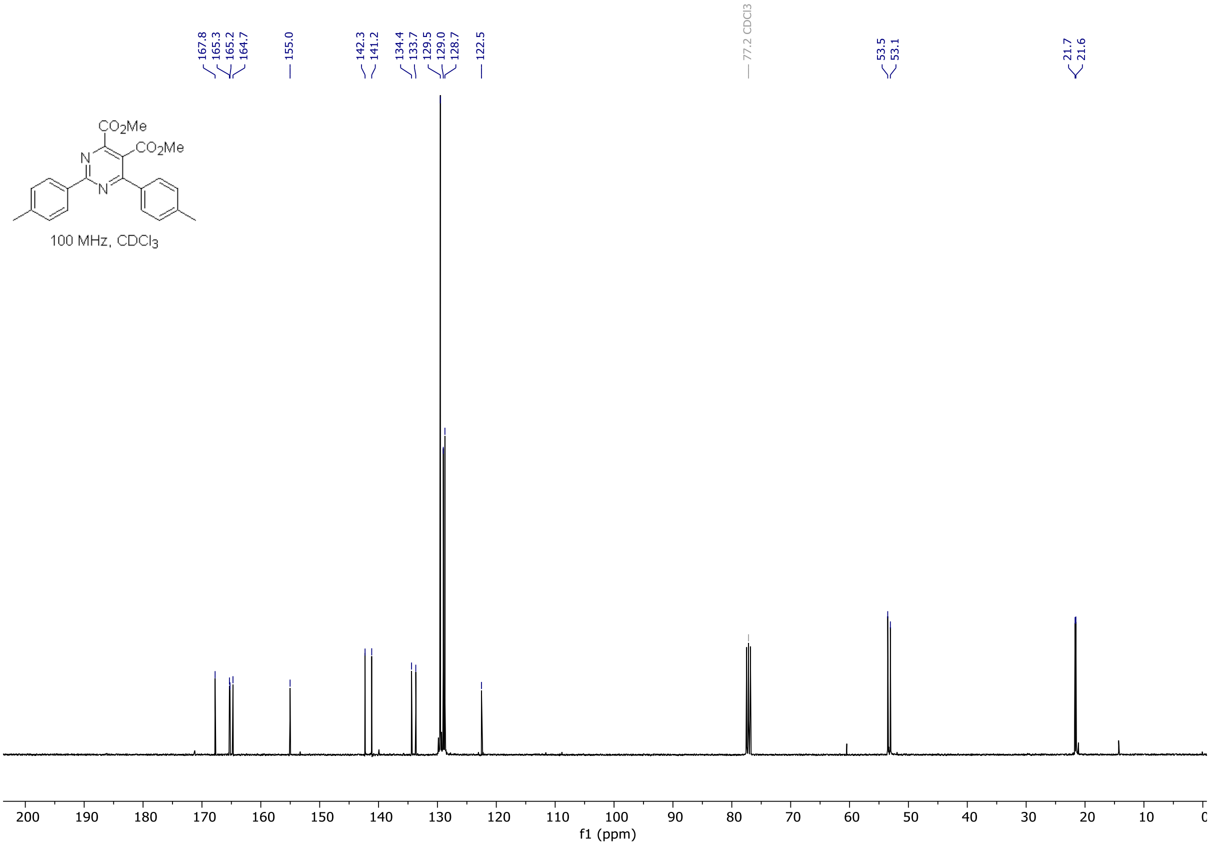

Dimethyl 6-(2-methoxyphenyl)-2-(3-methoxyphenyl)pyrimidine-4,5-dicarboxylate (7e)

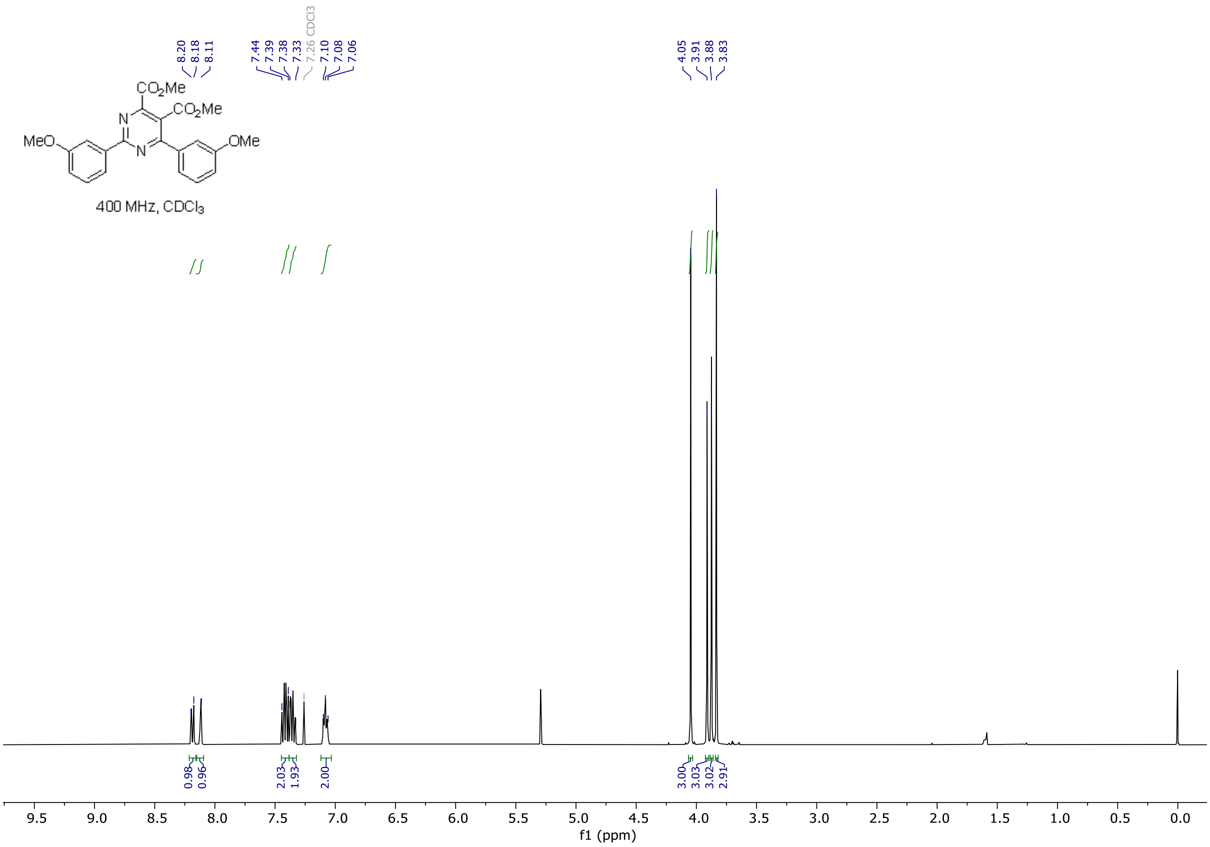

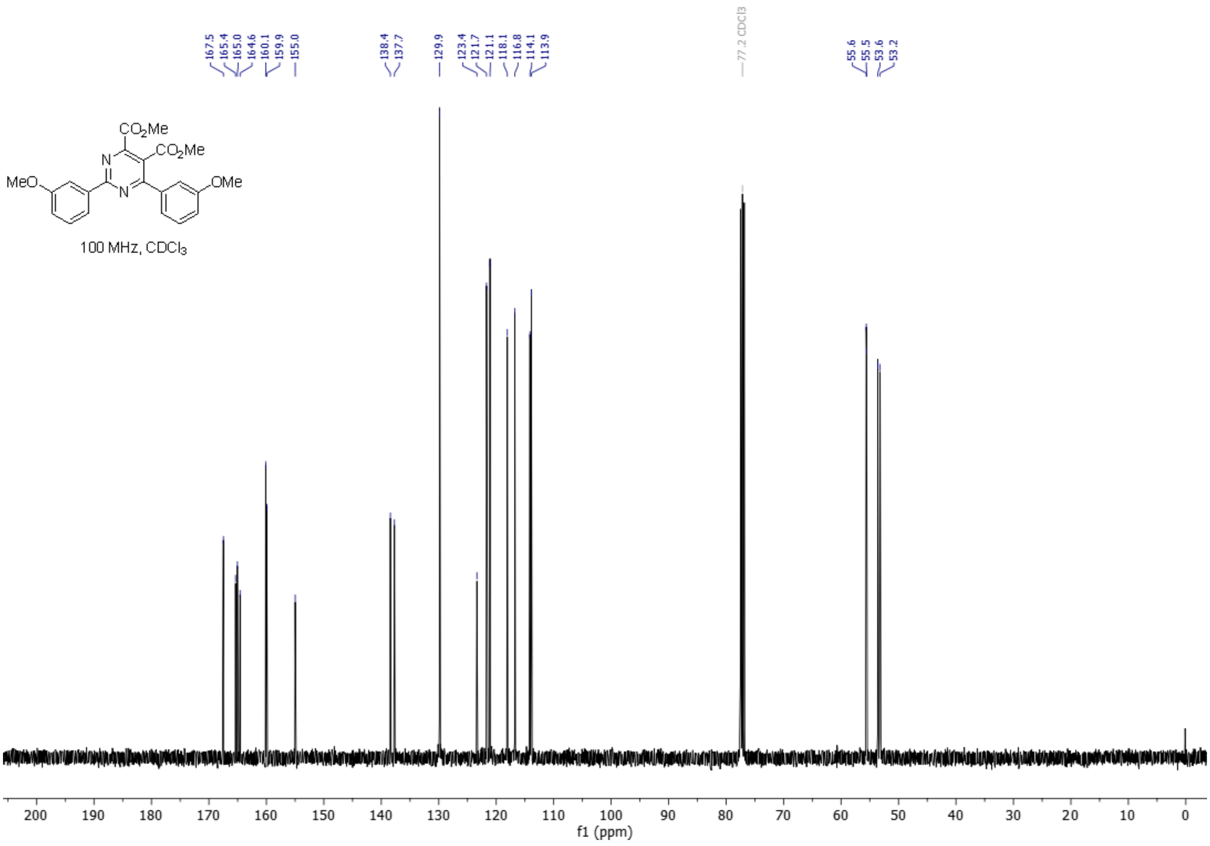

Dimethyl 2,6-bis(2,4-difluorophenyl)pyrimidine-4,5-dicarboxylate (7f)

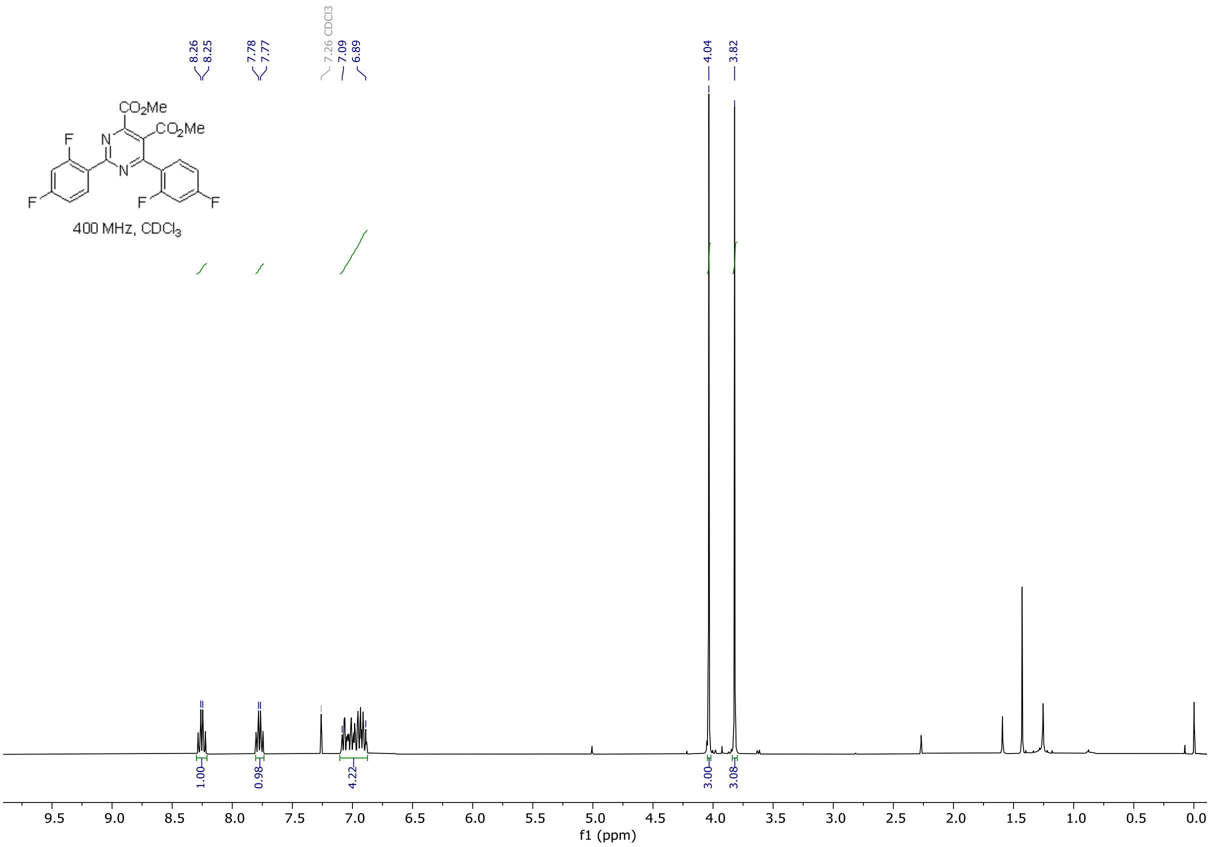

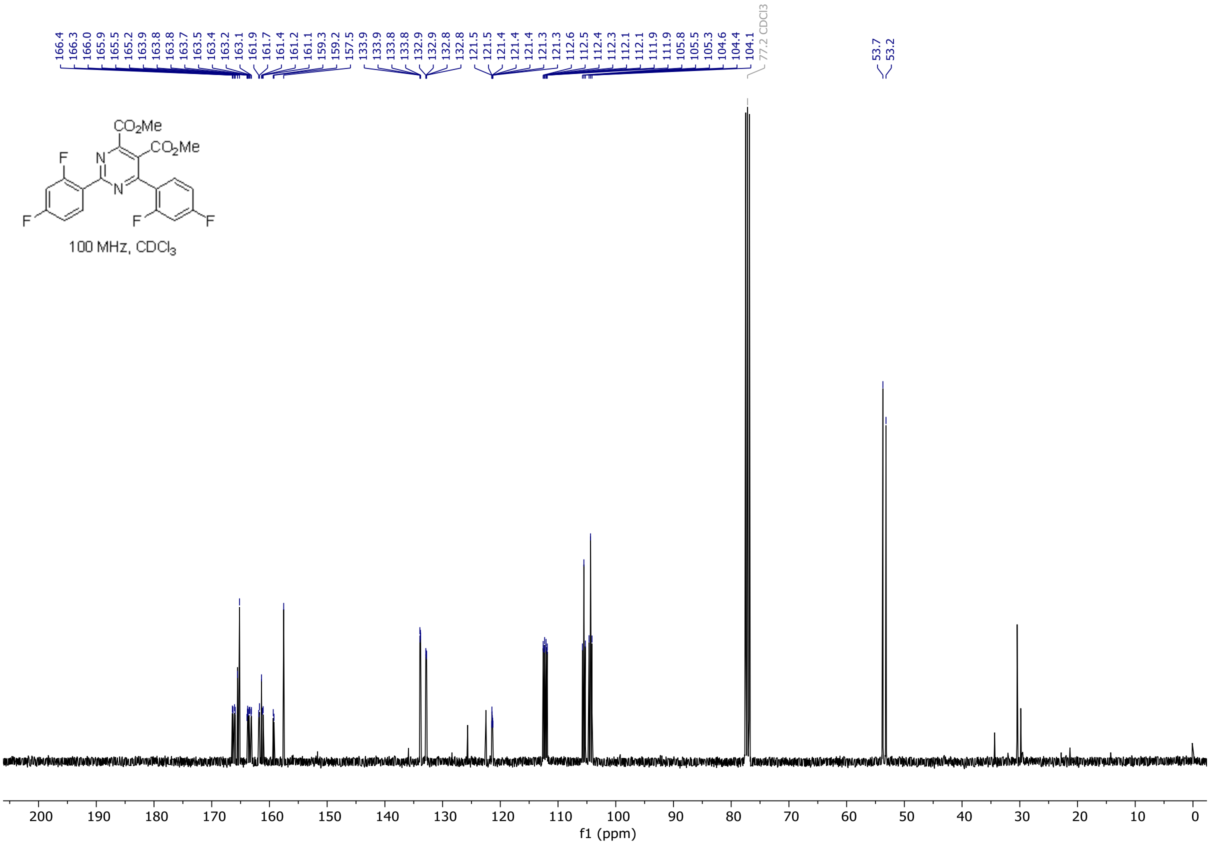

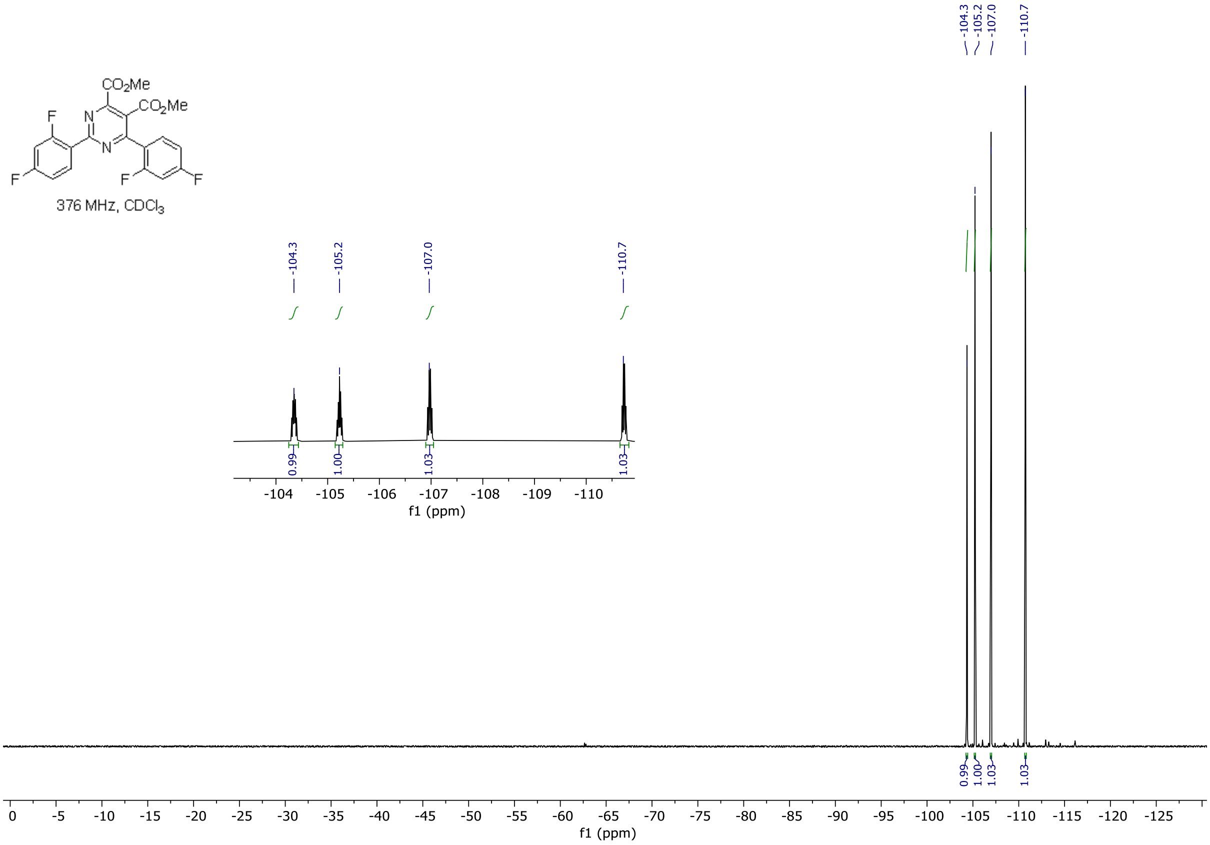
CO2Me
CO2Me
F
N
N
F
F
F
376 MHz, CDCl3
-104.3
-105.2
-107.0
-110.7
0.99
1.00
1.03
1.03
f1 (ppm)

Di-tert-butyl 2,6-diphenylpyrimidine-4,5-dicarboxylate (7g)

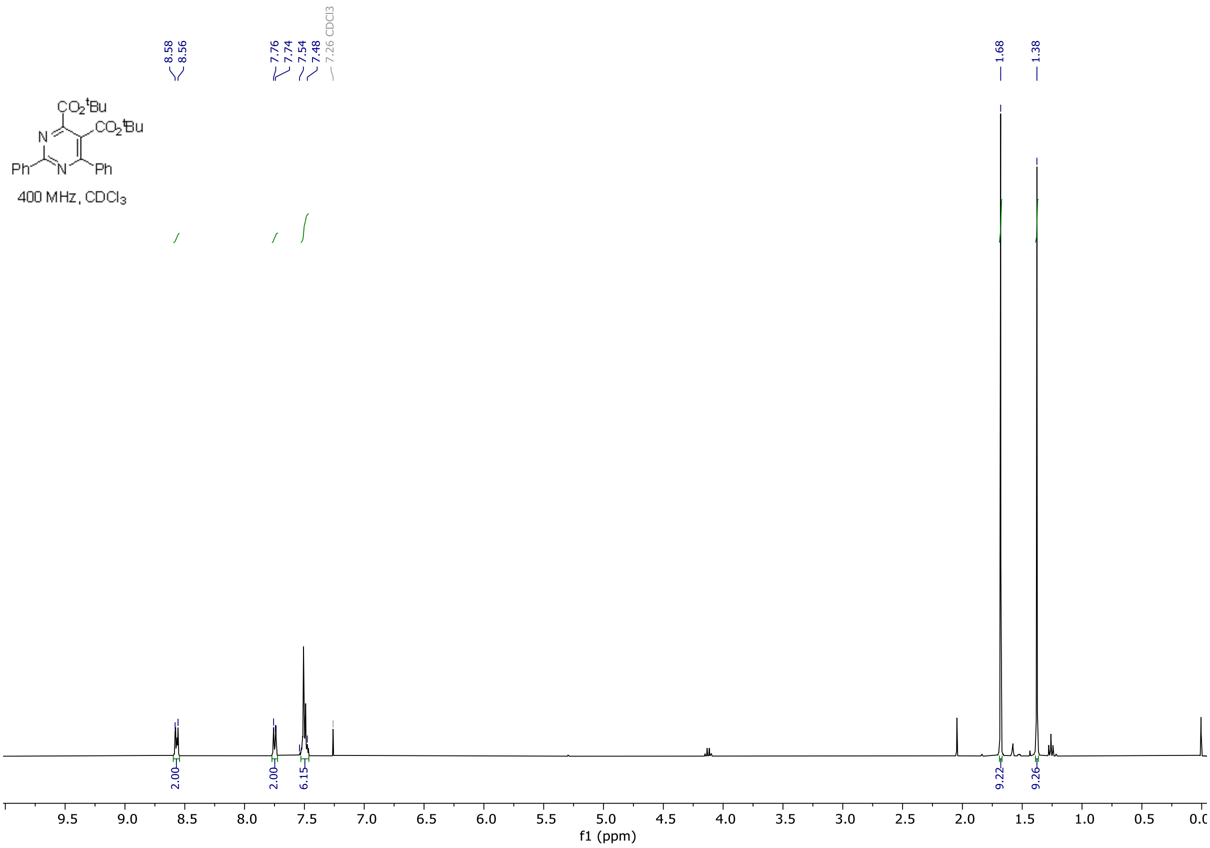

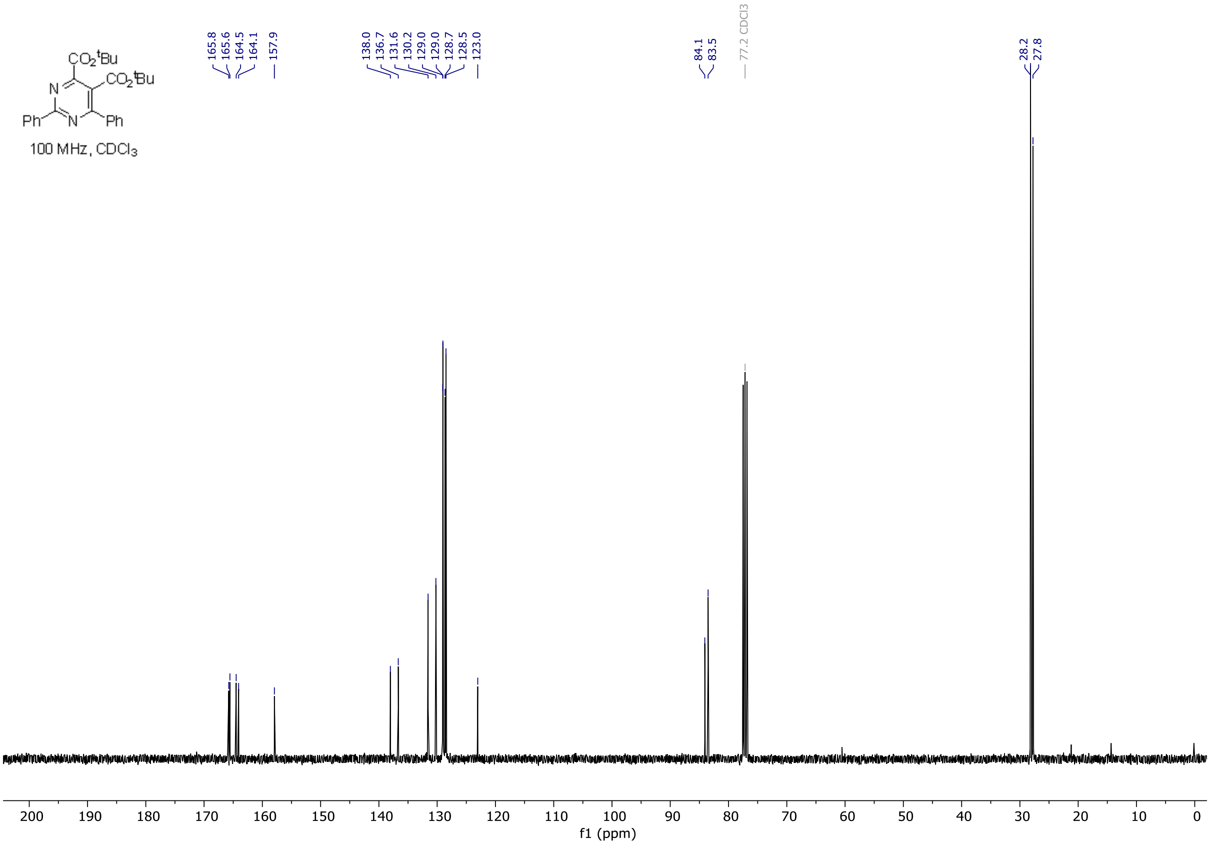

Di-tert-butyl 2,6-bis(4-methoxyphenyl)pyrimidine-4,5-dicarboxylate (7h)

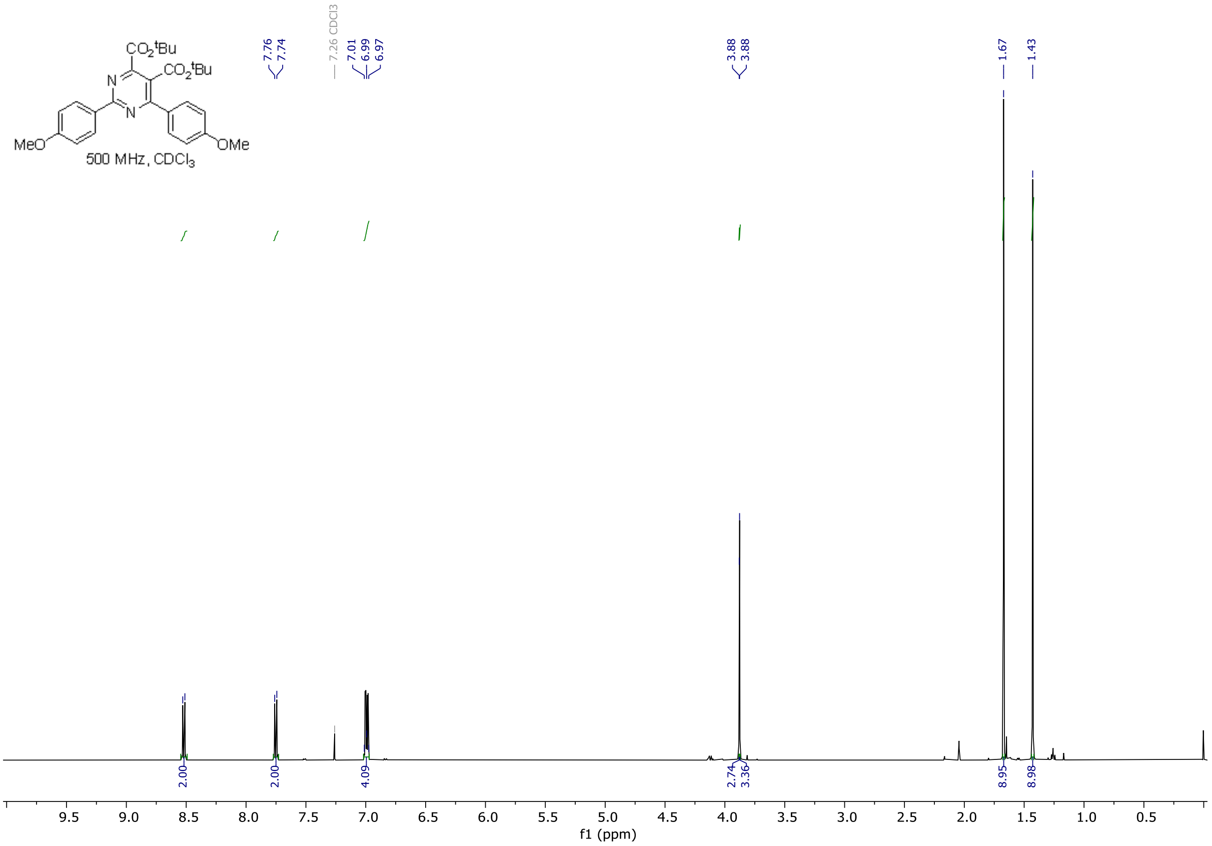

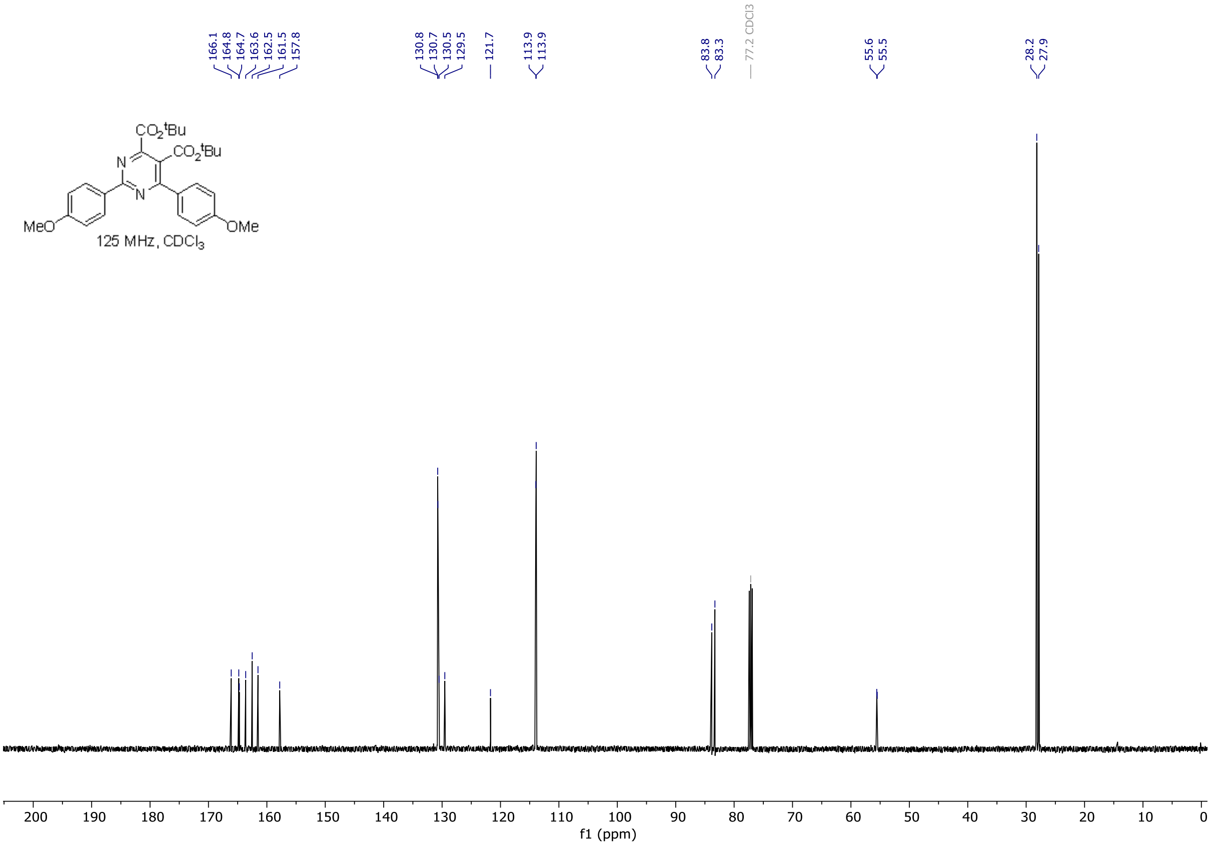

Di-tert-pentyl 2,6-bis(4-fluorophenyl)pyrimidine-4,5-dicarboxylate (7i)

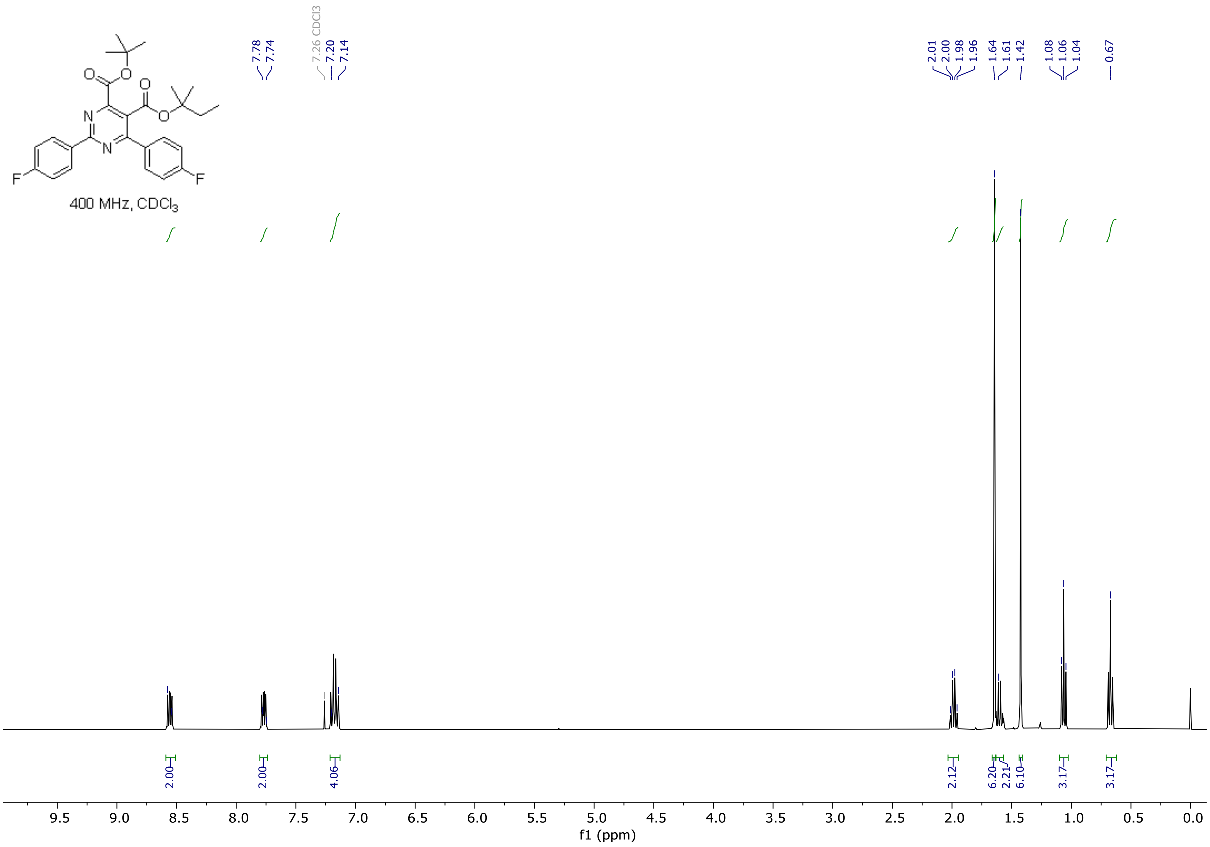

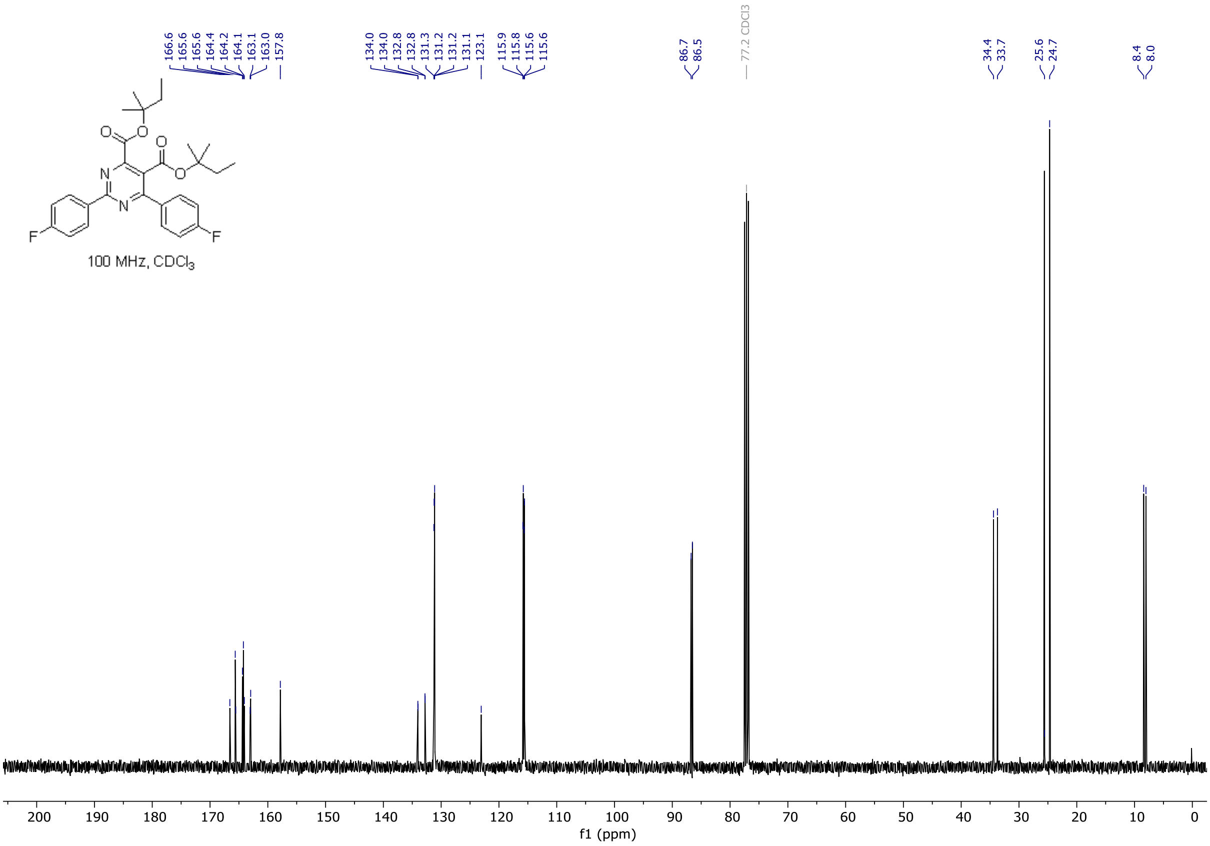

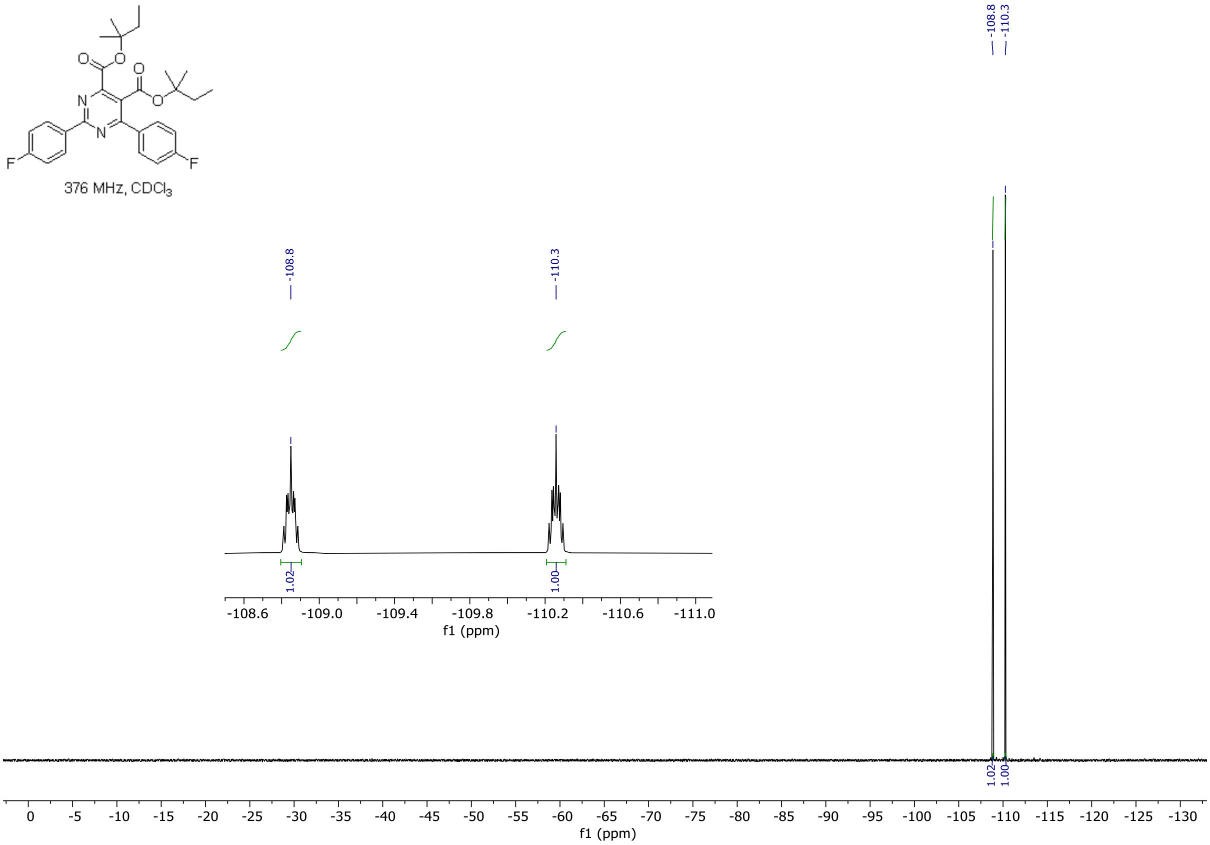
376 MHz, CDCl3
-108.8
-110.3
1.02
1.00
f1 (ppm)

Dimethyl 2,6-di-o-tolylpyrimidine-4,5-dicarboxylate (7j)

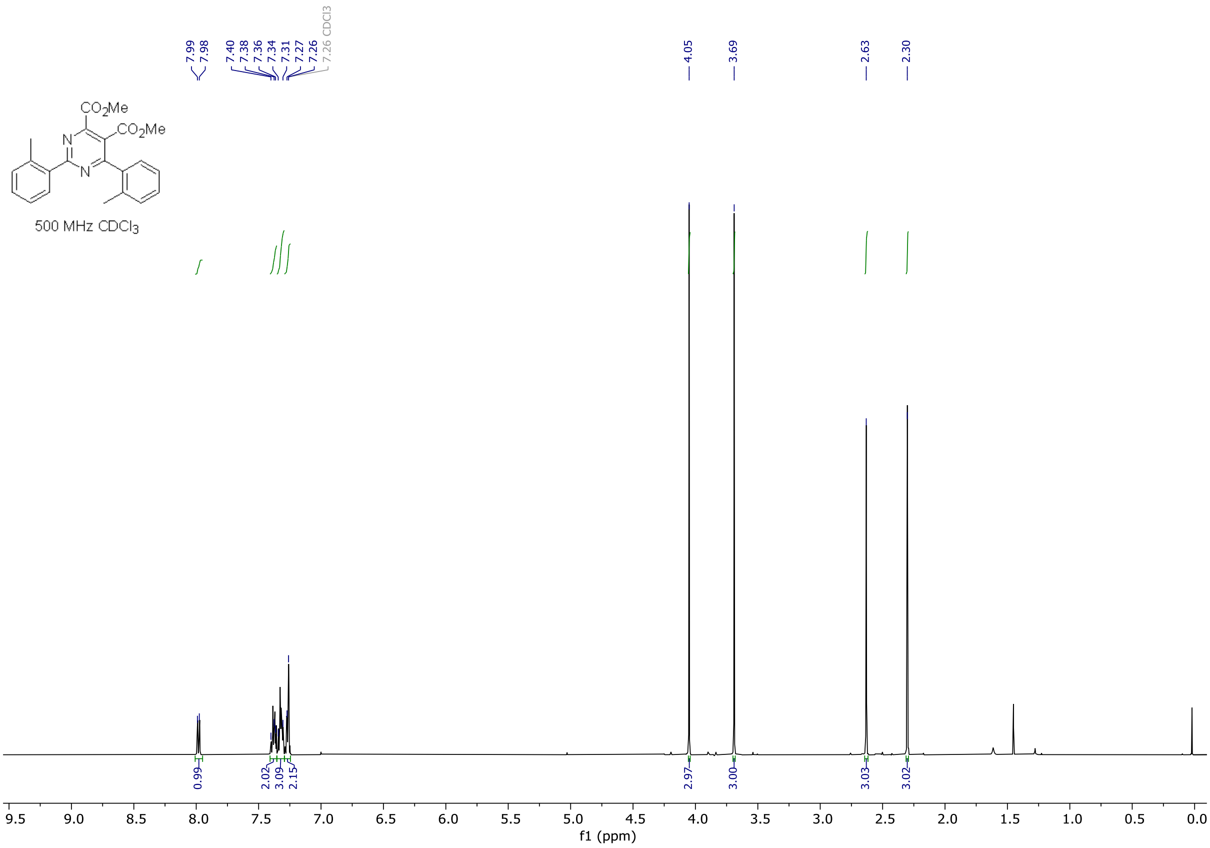

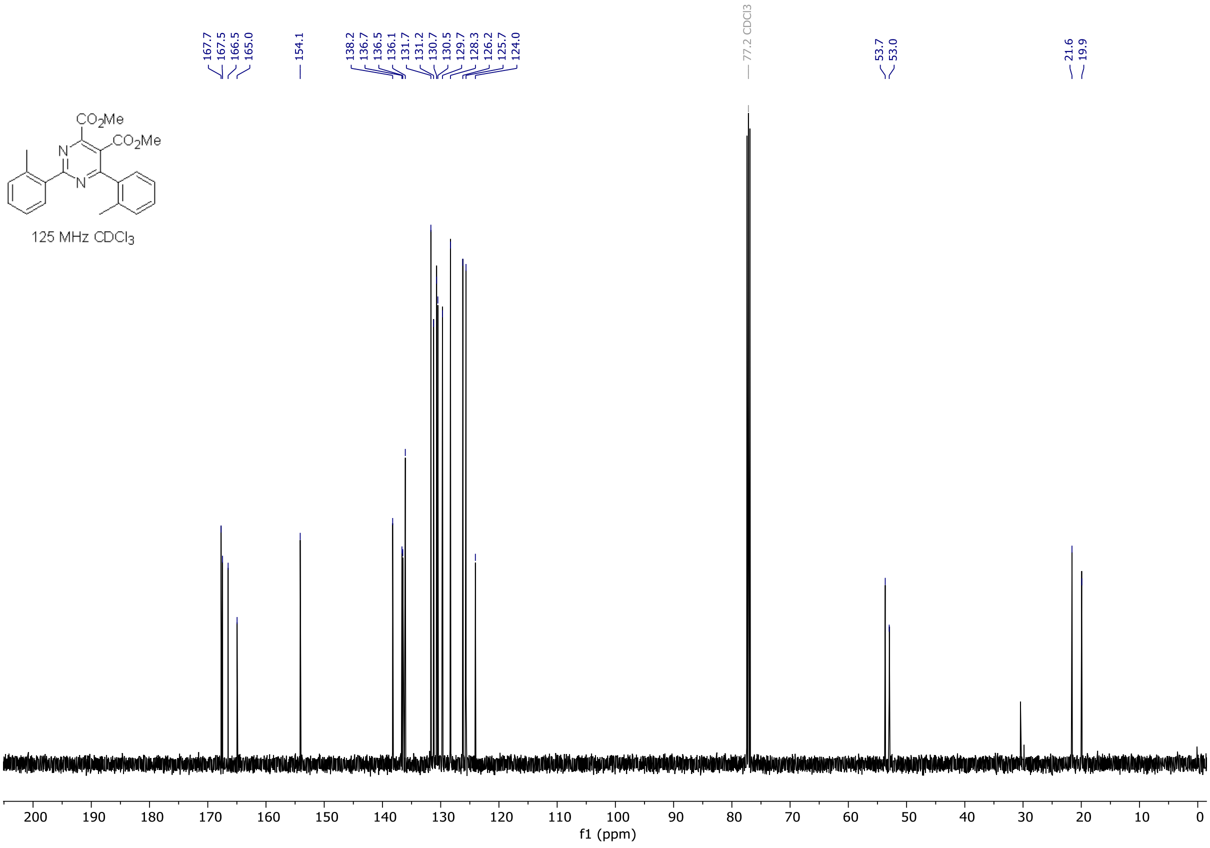

Di-tert-butyl 2,6-bis(4-fluorophenyl)pyrimidine-4,5-dicarboxylate (7k)

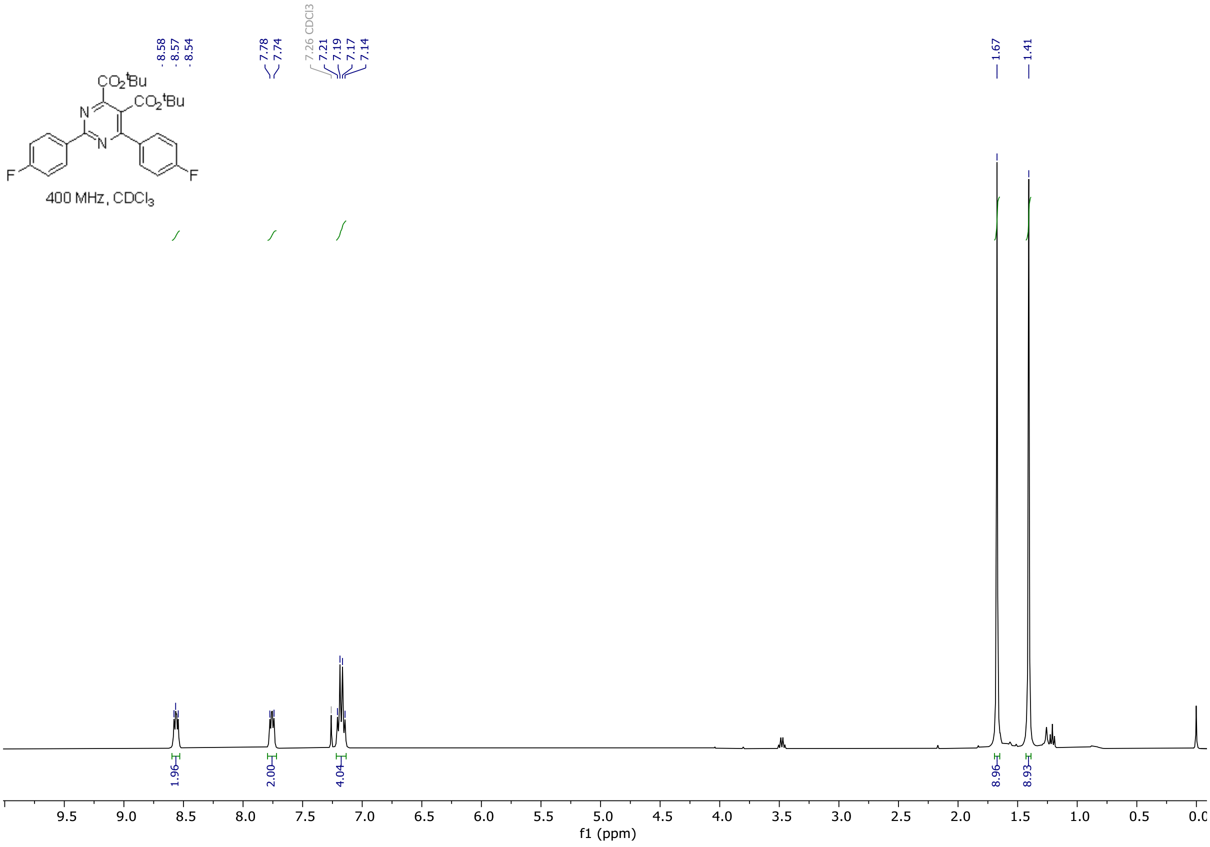

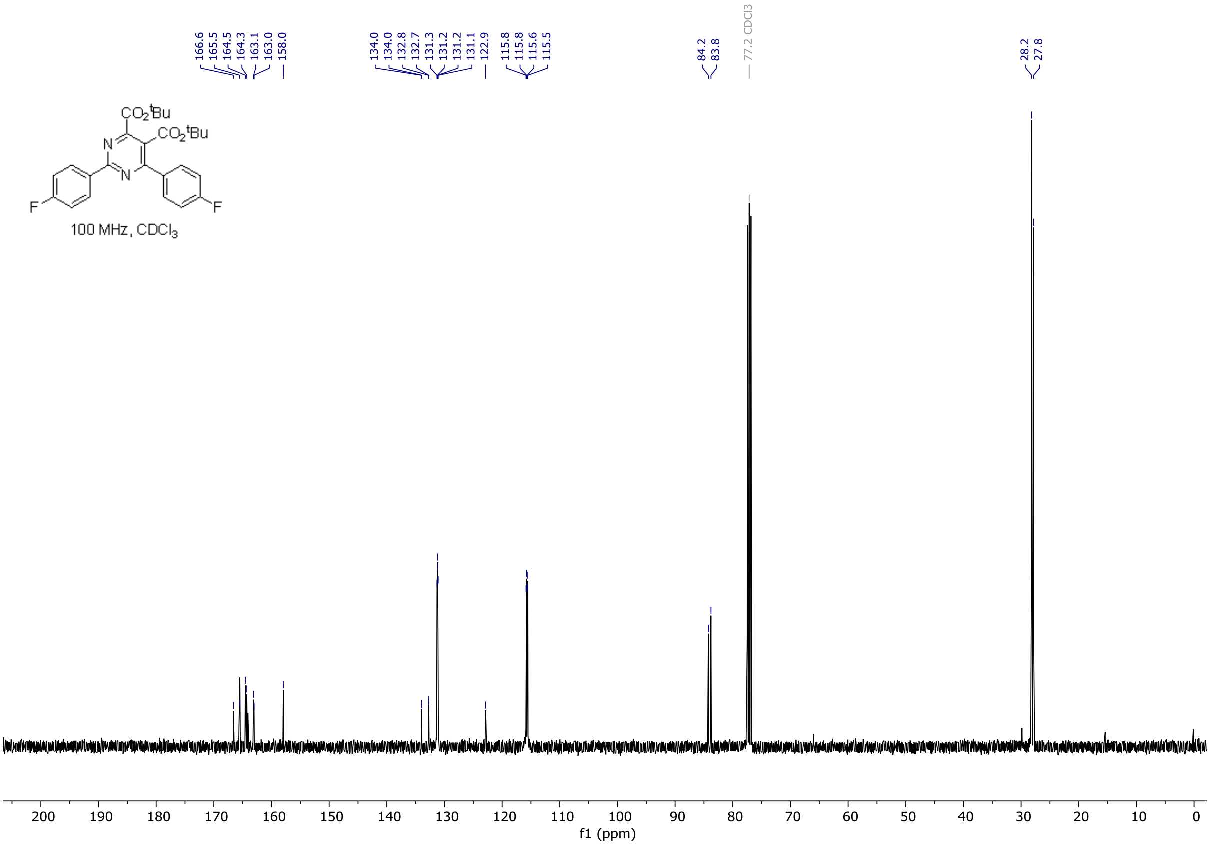

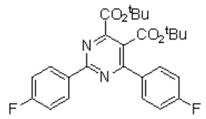

376 MHz, $CDCl_3$

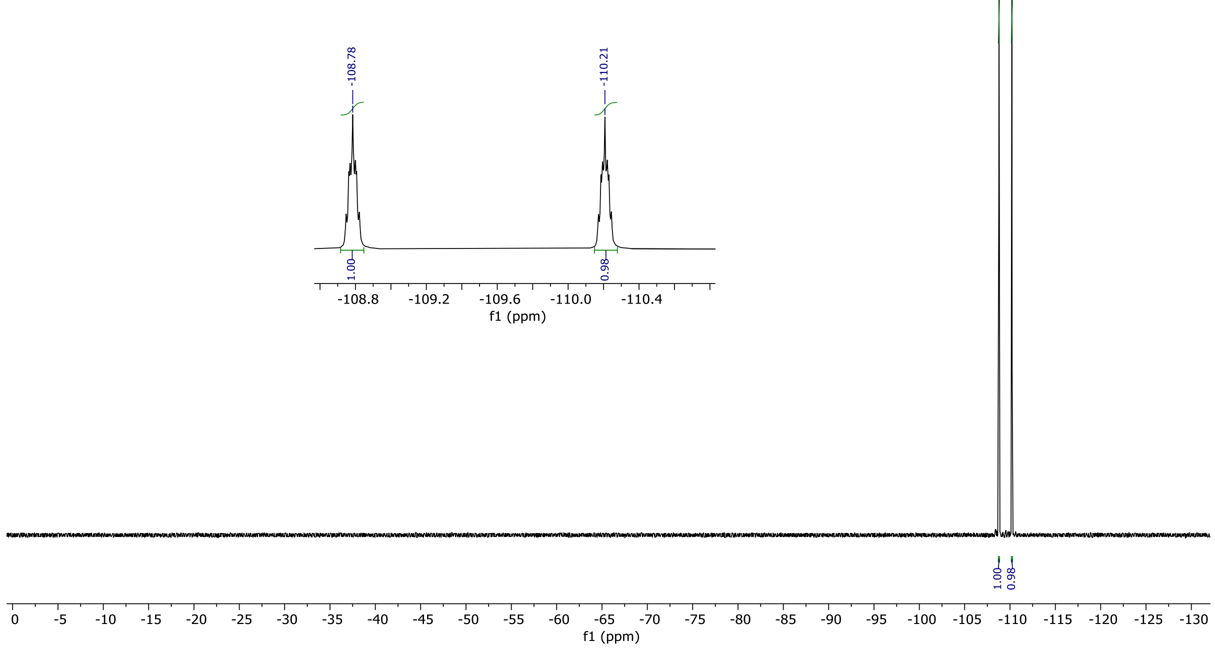

Supplement: Supplementary file 1 — Supporting Information [file CHEM-30-e202401491-s001.pdf]
